# Cell-active small molecule inhibitors validate the SNM1A DNA repair nuclease as a cancer target†

**DOI:** 10.1039/d4sc00367e

**Published:** 2024-04-30

**Authors:** Marcin Bielinski, Lucy R. Henderson, Yuliana Yosaatmadja, Lonnie P. Swift, Hannah T. Baddock, Matthew J. Bowen, Jürgen Brem, Philip S. Jones, Stuart P. McElroy, Angus Morrison, Michael Speake, Stan van Boeckel, Els van Doornmalen, Jan van Groningen, Helma van den Hurk, Opher Gileadi, Joseph A. Newman, Peter J. McHugh, Christopher J. Schofield

**Affiliations:** a Chemistry Research Laboratory, Department of Chemistry and the Ineos Oxford Institute for Antimicrobial Research, University of Oxford Mansfield Road Oxford OX1 3TA UK christopher.schofield@chem.ox.ac.uk; b Department of Oncology, MRC Weatherall Institute of Molecular Medicine, University of Oxford, John Radcliffe Hospital Oxford OX3 9DS UK peter.mchugh@imm.ox.ac.uk; c Centre for Medicines Discovery, NDM Research Building, University of Oxford Old Road Campus Research Building, Roosevelt Drive Oxford OX3 7DQ UK joseph.newman@cmd.ox.ac.uk; d University of Dundee, European Screening Centre Newhouse ML1 5UH UK; e Pivot Park Screening Centre 5349 AB Oss The Netherlands

## Abstract

The three human SNM1 metallo-β-lactamase fold nucleases (SNM1A–C) play key roles in DNA damage repair and in maintaining telomere integrity. Genetic studies indicate that they are attractive targets for cancer treatment and to potentiate chemo- and radiation-therapy. A high-throughput screen for SNM1A inhibitors identified diverse pharmacophores, some of which were shown by crystallography to coordinate to the di-metal ion centre at the SNM1A active site. Structure and turnover assay-guided optimization enabled the identification of potent quinazoline–hydroxamic acid containing inhibitors, which bind in a manner where the hydroxamic acid displaces the hydrolytic water and the quinazoline ring occupies a substrate nucleobase binding site. Cellular assays reveal that SNM1A inhibitors cause sensitisation to, and defects in the resolution of, cisplatin-induced DNA damage, validating the tractability of MBL fold nucleases as cancer drug targets.

## Abstract 2

SNM1 metallo-β-lactamase fold nucleases (human SNM1A–C) play roles in DNA damage repair and telomere maintenance. Screening and SAR studies lead to selective small molecules validating SNM1A as a cancer target.

## Graphical abstract

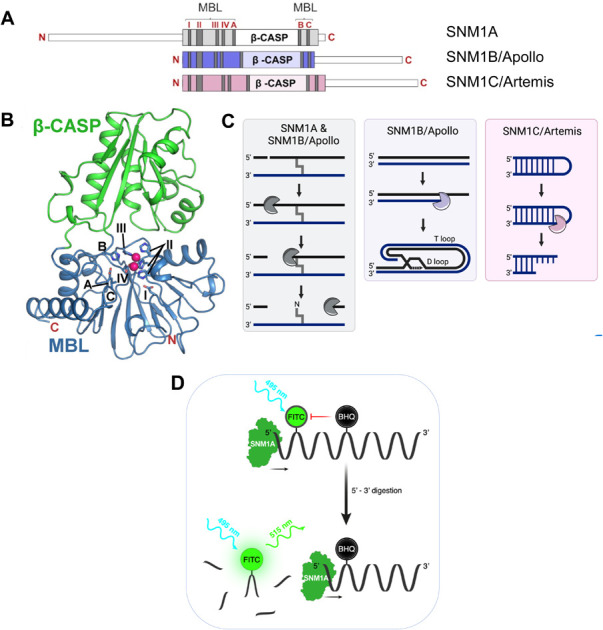

## Introduction

Cancer cells experience increased genomic stress over non-malignant cells, hence DNA repair is important in tumour propagation and some tumour cells are sensitive to the inhibition of pathways working to maintain genome stability.^1^ This principle has been exploited in medicine by poly-ADP-ribose polymerase (PARP) inhibitors that display a synthetic lethal interaction with cells defective in homologous recombination (HR).^2^ Two major classes of DNA damaging cancer treatments involve using drugs that induce DNA interstrand crosslinks (ICLs) and ionising radiation (and radiomimetic drugs) that induces chemically complex DNA double-strand breaks (DSBs). ICL-inducing chemotherapeutic drugs are widely employed, including in hard-to-treat cancers (pancreas, oesophageal, lung), but often with poor survival rates.^3,4^ By contrast, for reasons unclear, the combined use of ICL- and DSB-inducing treatments is often curative for testicular tumour treatment. Mutation and genome rearrangement frequencies also have an impact on immunotherapy, including immune checkpoint blockade.^5^ New ways to modulate tumour-DNA repair efficiency are thus of considerable interest.

**Fig. 1:**
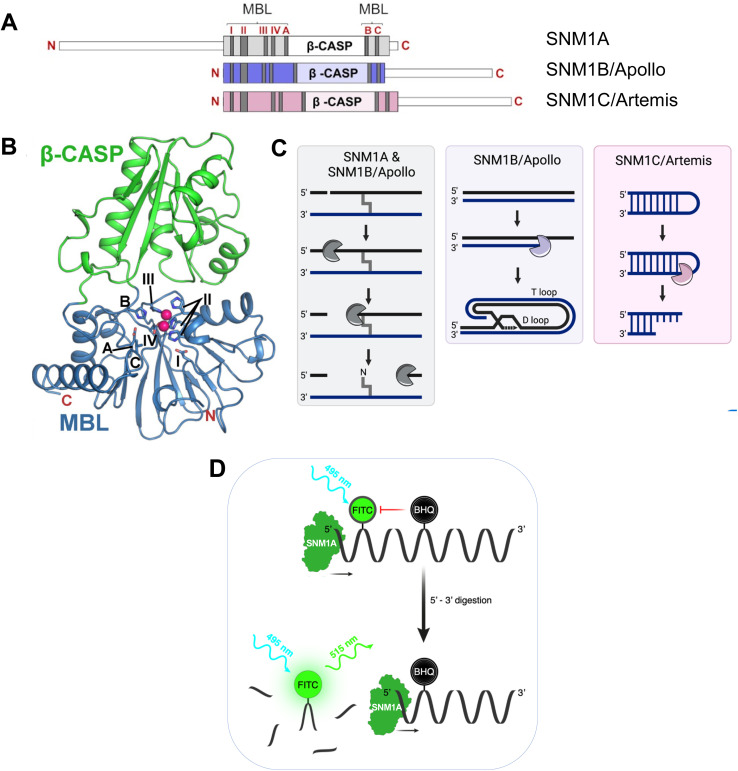
Overview of the domain organization, overall structure, key motifs, and molecular functions of the SNM1 nuclease family. (A) Domain organisation in human SNM1A, SNM1B and SNM1C showing MBL and β-CASP domains. The four highly conserved MBL fold motifs (1–4), are in red. Motif 1 = Asp; motif 2 = 3 His and 1 Asp (HxHxDH); motif 3 = His; and motif 4 = Asp. The β-CASP motifs are: motif A = Asp, motif B = His, motif C = Val. (B) Overall structure of the SNM1 family enzymes; MBL domain: blue: β-CASP domain: green. Metal ions in the active site are pink spheres and key residues forming MBL motifs are labelled and shown in stick format. (C) Schematic diagram of reactions catalysed by human SNM1 nucleases in DNA repair pathways and telomere processing. (D) Schematic of the fluorescence-based assay used in the high-throughput SNM1A inhibitor screen and hit validation: BHQ, black hole quencher-1; FITC, fluorescein isothiocyanate.

Potential DNA damage response (DDR) enzyme targets include the glycosylases, helicases, translocases, and nucleases that process damaged DNA.^1^ Three human ‘MBL-fold’ DNA repair factors, SNM1A, SNM1B (Apollo) and SNM1C (Artemis) are involved in repair of ICLs and DSBs. SNM1A–C have conserved β-CASP and metallo-β-lactamase (MBL) domains (Fig. 1A–C).^6–8^ The MBL fold is present in many hydrolases and other metallo-enzymes, including Ambler class B Zn(ii) dependent β-lactamases, where it has been shown that potent and selective *in vivo* inhibition can be achieved,^9,10^ including as a result of optimising hits arising from a high-throughput screen.^11^ Interestingly, given their central biological importance, there has been relatively little work on targeting MBL-fold nucleases, including SNM1A–C, as anti-cancer or other targets, with one exception being ongoing efforts to develop inhibitors of the mRNA MBL-fold processing Cleavage and Polyadenylation Specificity Factor (CPSF3) as a cancer target^12^ and as an antiparasitic agent.^13,14^

SNM1A–C are orthologous with a fungal ancestor, Pso2/Snm1, a key ICL repair factor in *Saccharomyces cerevisiae*.^15^ SNM1A/SNM1B are 5′–3′ exonucleases with the unusual capacity to hydrolyse DNA substrates containing lesions, including ICLs (Fig. 1C). SNM1A/SNM1B are both involved in ICL repair. SNM1B is also associated with repair of IR-induced DSB damage. SNM1A has a role in replication-coupled ICL repair,^16^ functioning with the endonuclease, XPF-ERCC1, to effectively “unhook” the ICL and allow downstream processing. SNM1B has a key role in telomere maintenance, mediated by its interaction with TRF2,^17,18^ where SNM1B resects the telomeric leading-strand to generate the 3′-overhang necessary for t-loop formation,^19^ which is required for telomere protection (Fig. 1C). Therapeutic targeting of SNM1B may thus induce senescence and/or loss of tumour cell viability due to telomere attrition. Very recently, a key role for SNM1A as an effector of the break-induced synthesis at telomeres has been unveiled, implying SNM1A is a potential target in tumours that depend upon telomere maintenance governed by the alternative lengthening of telomeres (ALT) pathway.^20^

SNM1C was first identified in studies of congenital radiosensitive severe acquired immune deficiency (RS-SCID).^21^ When bound to, and phosphorylated in its C-terminal region (which is distal to the MBL-β-CASP core fold) by DNA-dependent protein kinase (DNA-PK), SNM1C acquires endonuclease activity resulting in hydrolysis of hairpin-end intermediates generated during class-switch recombination and V(D)J recombination (Fig. 1C), a role consistent with the SCID phenotype caused by lack of SNM1C.^21^ SNM1C also has 5′–3′ exonuclease activity, though this is apparently weaker than that of SNM1A and SNM1B.^22^ It is likely that the radiosensitivity associated with SNM1C deficiency results from reduced capacity to process chemically damaged DNA termini at DSBs as part of the NHEJ repair pathway. SNM1C is thus a highly attractive target for sensitising cells to IR and radiomimetic drugs.

Early work to identify SNM1A–C inhibitors revealed moderate inhibition by some cephalosporin antibiotics providing an interesting link to the true bacterial MBLs.^23^ Squaramides and certain nucleoside analogues also inhibit SNM1A, however, with modest potency.^24,25^ Crystallographic analyses on SNM1A/SNM1B have revealed a 5′-phosphate binding pocket, which is absent in SNM1C, but which is critical for efficient SNM1A/SNM1B activity.^26^ Consistent with this observation, the addition of a group mimicking a 5′-phosphate was found to improve the potency of modified nucleoside SNM1A inhibitors.^27^

Here, we report how a high-throughput screen identified novel SNM1A inhibitor chemotypes. Optimisation enabled the identification of potent hydroxamic acid-based inhibitors, with candidates displaying selectivity for the individual SNM1 nucleases. The selective SNM1A inhibitors are cell-active, sensitise cancer cells to a key crosslinking anticancer drug (cisplatin), and lead to trapping of the SNM1A nuclease at sites of ICL-induced damage. The combined result provides strong evidence for the validity and tractability of SNM MBL-fold nucleases as cancer drug targets.

## Results

### A high-throughput screen identifies candidate SNM1A inhibitor pharmacophores

With the aim of identifying potent SNM1 family inhibitors, a European Lead Factory (ELF) public-private partnership high throughput screen of >302 000 compounds was performed against SNM1A in 384 well plate format over 3 days (mean *Z*′ > 0.74) (ESI Fig. 1†); the screen utilised an optimised version of a reported fluorescence-based assay.^23^ In brief, the fluorescence-based nuclease assay utilises a chemically modified 20-nucleotide ssDNA substrate with a fluorescein isothiocyanate (FITC) derived 5′-group and an internal black-hole-quencher-1 (BHQ-1), located eight nucleotides distal to the fluorescein group. In the intact substrate the BHQ-1 suppresses fluorescence, however, exonucleolytic digestion of the substrate from the 5′ end by SNM1A uncouples the BHQ-1 and FITC moieties (see Fig. 1D). Spectra were measured every 140 seconds with an increase in fluorescence indicating enzymatic activity by SNM1A (concentration: 0.1 nM). 7-Aminocephalosporanic acid (7-ACA) was used as a positive control (IC_50_ 12.2 mM). Overall, 19 structural clusters were identified of which 14 were singleton clusters. From the ELF screen, 24 candidate hits, with a range of chemotypes, with half maximal inhibitory concentration (IC_50_) values ranging from low micromolar to high nanomolar values were identified, providing a foundation for inhibitor development (ESI Fig. 1†)

We worked to obtain information on the binding modes of the hit compounds by soaking SNM1A crystals with them (1 mM final concentration), both in the presence and absence of added ZnCl_2_. Although the identity of the metal ion(s) used by SNM1s *in vivo* is uncertain, the available bio-chemical/physical evidence is that this is most likely Zn(ii). MBL fold enzymes normally have two metal ion binding sites (M1 and M2, Fig. 1C), although sometimes only one site is occupied or is required for catalysis.^28^ We, and others, have observed variations in SNM1 active site metal ion occupancy by crystallography, which may relate to differences in purification methods, in particular, when using Ni-affinity chromatographic (IMAC) purification. Indeed, structures of SNM1A–C enzymes are reported in both mono- and di-metal ion active site forms.^26,29–31^ When only a single metal ion is present it occupies the M1 site and is ligated by 4 protein residues (H732, H734, H737 and D815, in the case of SNM1A). The second metal ion site (M2) only has 3 protein residues as ligands (D736, H737 and D815 in the case of SNM1A); it may be that the M2 metal ion is bound less tightly and as such is not always carried through the purification procedures.

Because the relevant *in vivo* metalation status of the SNM1A used in the high-throughput screen cannot be unequivocally determined, soaking was performed under two conditions using the orthorhombic SNM1A crystal form. This crystal form grows from conditions containing malonate, imidazole and boric acid (MIB) buffer and manifests an active site where a malonate ion is bound to a single M1 site metal ion (presumed to be a nickel ion due to IMAC purification) (ESI Fig. 2†). To obtain a single metal malonate free form, crystals were resoaked in buffer lacking malonate (0.1 M HEPES pH 7.0, 30% PEG 1000) overnight. To obtain a two-metal ion form the same crystals were re-soaked in buffer lacking malonate, but with ZnCl_2_ (0.1 M HEPES pH 70, 30% (v/v) PEG 1000, 500 μM ZnCl_2_), a process enabling occupation of the M2 site with high occupancy (ESI Fig. 2†).

**Fig. 2:**
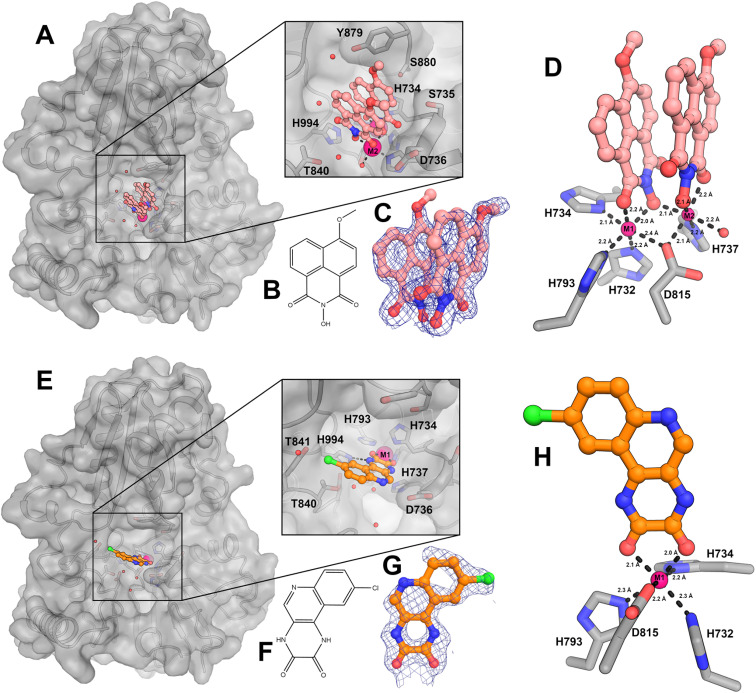
Structural characterization of the SNM1A binding mode of hit compounds 1 and 2 identified from the high throughput screen. (A) Surface representation from a crystal structure of SNM1A complexed with 1; the inset shows a close-up view with interacting residues labelled (inset). (B) Structure of 1. (C) 2Fo–1Fc electron density maps contoured at 1*σ* in the vicinity of 1. (D) Close-up view of the interaction of 1 with the metal ions in the active site. Metal ions are shown as pink spheres with key residues labelled and co-ordination distances shown. (E) Surface representation of SNM1A in complex with 2; the inset shows a close-up view with interacting residues labelled. (F) Structure of 2. (G) 2Fo–1Fc electron density maps contoured at 1*σ* in the vicinity of 2. (H) Close up view of the interaction 2 with the metal ion in the SNM1A active site. Metal ions are shown as pink spheres with key residues labelled and co-ordination distances shown.

Soaking of SNM1A crystals with the ELF hits for which inhibition was validated (see below), that is 1 [IC_50_ = 2.4 μM] and 2 [IC_50_ = 2.0 μM], was successful for 1 in the two-metal ion SNM1A form and 2 in the single metal ion SNM1A form (Fig. 2A–H). The electron density maps reveal both 1 and 2 bind directly to the metal ion(s) *via* oxygen atoms. Interestingly, 1 is an *N*-hydroxyimide, a pharmacophore that is known to inhibit nucleases and other metalloenzymes including the true bacterial MBLs.^32,33^

Two molecules of 1 were observed in the SNM1A active site, each positioned to coordinate to M1 or M2 *via* two oxygen ligands giving an octahedral arrangement (Fig. 2A and D); precedent for an MBL fold inhibitor binding mode involving two molecules comes from the observation of binding of two rhodanine derived inhibitor molecules at the active site of a true MBL.^34^ The molecule of 1 bound to the M1 site is positioned to hydrogen bond with the main chain amide of D736 and to interact with the side chains of S735, Y879 and S800 (Fig. 2A). The molecule of 1 bound to the M2 site is positioned to form a potential hydrogen bond with H994 and to make a water mediated contact with Y184 (Fig. 2A). The aromatic rings of both molecules of 1 stack with each other in a staggered arrangement, with the ring–ring distances between the two 1 rings being 3.2–3.5 Å (Fig. 2D).

The electron density map for the SNM1A: 1 complex revealed an apparent inconsistency between the ELF reported small molecule structure and the crystallographic data. The ELF report has the methoxy group of 1 at the *meta*-position, however, the electron density maps clearly imply that the methoxy substituent is in the *para*-position for both crystallographically observed molecules of 1 (Fig. 2C). Re-synthesis of each of 1, 2, and 3 revealed comparable levels of SNM1A inhibition as determined by the real-time fluorescence-based nuclease assay for each of the regioisomers with IC_50_ values of 2.4 μM, 2.0 μM, and 2.9 μM, respectively (ESI Fig. 3†).

**Fig. 3:**
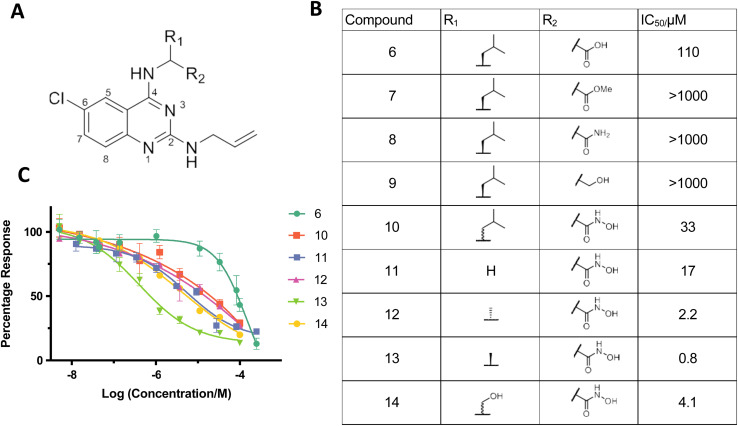
Identifying quinazoline–hydroxamic acids as a pharmacophore against SNM1A. (A) General structure of the K1 scaffold. (B) Table showing IC_50_ values of compounds 6–14 tested against SNM1A. (C) Effects of K1 derived compounds (6 and 10–14) on SNM1A exonuclease activity assessed using a real-time fluorescence-based nuclease assay, shown as mean ± SEM of four independent repeats.

The structures of 1 and 3 are closely related to *N*-hydroxyimides that are known to inhibit other nucleases, including XPF-ERCC1 and FEN1 (flap structure specific endonuclease 1).^35,36^ We also tested the inhibitory potential of a potent and *N*-hydroxyimide based FEN1 inhibitor against SNM1A, that is AZ1353160 (4). Whilst 4 showed some SNM1A inhibition, this was incomplete and an IC_50_ value could not be calculated from the fluorescence-based nuclease assay data. A structure of 4 complexed with SNM1A was solved to 1.7 Å resolution (ESI Fig. 4A–D†). As observed with 1, AZ1353160 (4) binds to the di-metal SNM1A form with two molecules of 4 at the active site, both of which are positioned to coordinate to the active site metal ions *via* oxygen atoms, in a similar manner to that observed for 1 (Fig. 3C). AZ1353160 (4) was obtained as a racemic mixture (ESI Fig. 4B†) and the best fit to the density was obtained with one (*R*)-enantiomer and one (*S*)-enantiomer, at the M1 and M2 sites, respectively (ESI Fig. 4C†). As for 1, the two 4 molecules stack in a staggered manner, with differences likely reflecting the benzo-dioxane substituent on the urea nitrogen of 4. The AZ1353160 (4) binding mode with SNM1A contrasts with that observed for it with FEN1,^36^ where a single inhibitor bridges the two magnesium ions in the FEN1 active site forming two contacts to each metal ion (ESI Fig. 5†).

As for binding of 1 at the M1 site, the one molecule of 2 observed at the active site was also observed to coordinate the SNM1A M1 metal ion form *via* its two carbonyl oxygens (metal–ligand distances: 2.0 and 2.1 Å) (Fig. 2E–H); its SNM1A binding mode is similar to that of the catechol group of ceftriaxone (5) which inhibits SNM1A and SNM1C with low μM potency^23,30^ (ESI Fig. 6†). A hydrogen bond is formed between H994 and the 2 diamide nitrogen (Fig. 2E) and the H_2_ chlorobenzene ring is close to the Y841 and T840 sidechains (Fig. 2E).

The obtained structures reveal both 1 and 2 (and, likely 3) bind directly to the active site metal ion(s) of SNM1A *via* two oxygen atoms and likely inhibit in a competitive manner with the DNA substrate, displacing the Zn(ii) ion complexed hydrolytic water/hydroxide that, in models of the reaction mechanism, is positioned for hydrolysis of the phosphodiester bond in the DNA backbone. Ceftriaxone (5) is an inhibitor of SNM1A/SNM1B, complexing the active site metal ion *via* its catechol group.^23^ We compared the inhibition by ceftriaxone (5) with that of 1, 2 and 3 and found each 1, 2 and 3 to be more potent and consistent inhibitors of SNM1A than ceftriaxone (5) (ESI Fig. 3†). We therefore employed 1 as a positive control in subsequent fluorescence-based and gel-based nuclease SNM1 inhibition assays. Although, we have not pursued this in our current work, the different observed binding modes for 1 and 2 suggest that it may be possible to identify inhibitors selective for binding to forms of SNM1A with one or two metal ions.

### Identification of hydroxamic acids as pharmacophore against SNM1A

The three rings of 1 and 3 likely contribute to their poor solubility and hence limit their usefulness as inhibitors. It is notable, however, that the SNM1A inhibitor 1, which we subsequently used as a positive control, also inhibits both SNM1B and SNM1C with IC_50_ values of 1.9 μM and 0.37 μM, respectively (ESI Fig. 7†), potentially enabling efforts to target these enzymes. However, given the poor solubility of 1/2 and the challenges we experienced in improving inhibition based on their structures, we did not develop further cyclic *N*-hydroxyimide based inhibitors, instead pursuing mechanistically related hits, in particular 6.

In the ELF screen a structurally similar quinazoline-based inhibitor, K1 (6) was identified as an SNM1A inhibitor, with an IC_50_ value of 17.4 μM. Resynthesised K1 (6), however, had a higher IC_50_ value for SNM1A (129 μM); despite being less potent than 1 and 2; K1 (6), however, possesses a heteroaromatic structure we considered more amenable to modification. We therefore developed a synthetic scheme (ESI Fig. 8†) suitable for the versatile synthesis of K1 (6) analogues.

We hypothesised that the carboxylic acid of K1 (6) may bind to one or both of the active-site metal ions. Indeed, when the carboxylic acid of 6 was replaced with normally less effective chelating groups, such as ester (7), amide (8) or hydroxyl (9) groups, there was a marked loss of SNM1A inhibition (Fig. 3B and C). Given the potency of both 1 and 3 as SNM1A inhibitors and the information about binding provided by the SNM1A: 1 crystal structures (Fig. 2), we substituted the carboxylic acid of K1 (6) with a hydroxamic acid (10), a change which resulted in a 3-fold increase in potency (Fig. 3B and C). Hydroxamic acid-based inhibitors containing different amino acid derivatives were then synthesised and tested against SNM1A (11–14); all showed significant improvement in potency compared to K1 (6). Compound 13 containing an l-alanine based sidechain was identified as the most potent inhibitor against SNM1A with an IC_50_ of 0.8 μM (Fig. 3B and C).

### Hydroxamic acids as inhibitors of SNM1A, SNM1B/Apollo and SNM1C/Artemis

**Fig. 4:**
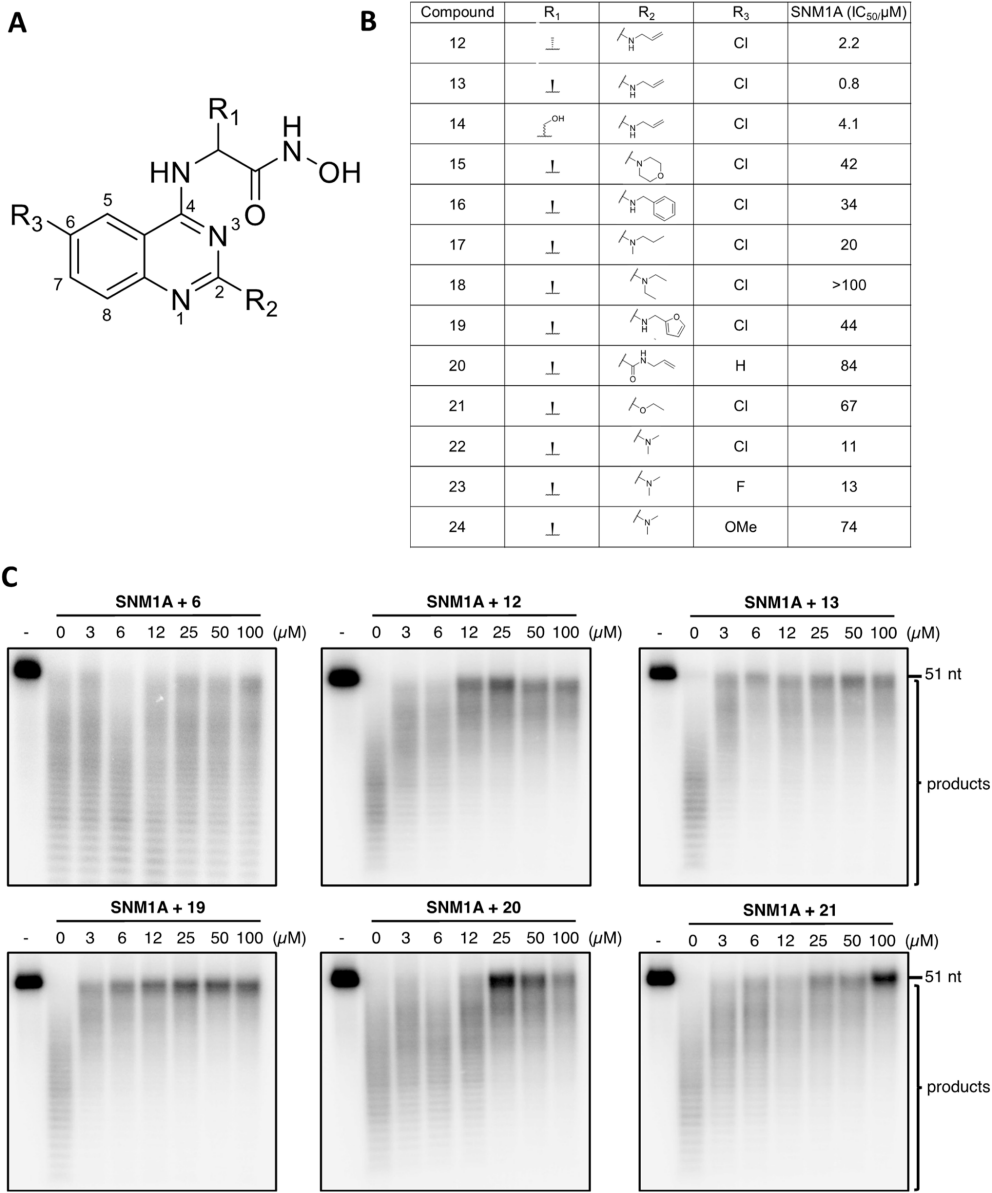
Testing quinazoline–hydroxamic acid derivatives against SNM1A. (A) The quinazoline–hydroxamic acid SNM1A inhibitor scaffold. (B) Table showing IC_50_ values of 12–24 tested against SNM1A. (C) Increasing concentrations (as indicated, in μM) of compounds incubated with 0.5 nM SNM1A (room temperature, 10 min), before initiating nuclease reaction by addition of ssDNA (37 °C, 20 min). Products were analysed on 20% denaturing SDS-PAGE gels. Gels are representatives from a minimum of three independent experimental repeats.

**Fig. 5:**
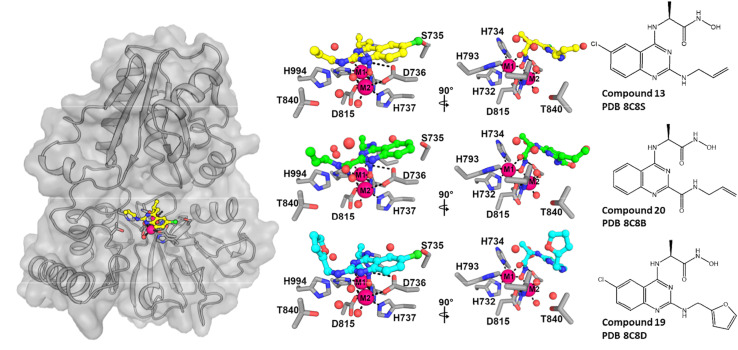
Views from crystal structures of quinazoline–hydroxamic acid-based inhibitors bound to SNM1A. The left-panel shows a surface view with representative hydroxamic inhibitors (13) bound to the active site. The central panel shows orthogonal views of the interaction between the hydroxamic acid inhibitors and the SNM1 active site. The right-hand panel shows the chemical structures of the compounds with corresponding PDB codes.

Following modifications of the amino acid side chain at the 4′-position, we attempted to optimise inhibitor 13, by modifying the side chain linked to the 2′-position of the quinazoline ring using a broad range of amine derivatives (Fig. 4). Alcohol and amides at the 2′-position were also tested as replacements of the amine side chain (Fig. 4). There is a potential interaction between the 6′-chloro group of compounds 13 & 19 and the side chain of Ser 375 (Fig. 5), although it is not clear if this interaction is a major contributor towards binding affinity due to the rather limited number of substitutions at this position in our synthesized compounds.

There is structural similarity between SNM1A, SNM1B, and SNM1C and major mechanistic features are likely conserved between the three nucleases. We therefore tested (13) and other hydroxamic acid analogues for inhibition of SNM1B and SNM1C. Due to differences in catalytic turnover between the three enzymes, different concentrations of SNM1B (1 nM) and SNM1C (2.5 nM) were used compared to that for SNM1A (0.5 nM) in the assays. Note that the substrate utilised for the high-throughput fluorescence based and subsequent SNM1A and SNM1B assays differed slightly for SNM1C reflecting its (primarily) endonucleolytic, rather than exonucleolytic, activity (ESI Fig. 9†).

Many of the hydroxamic acid compounds inhibit all three of SNM1A, SNM1B and SNM1C, though there were notable differences in potency (ESI Fig. 9†). The most potent inhibitor against SNM1A was 13 with an IC_50_ of 0.8 μM. In general inhibitors with a small sidechain at position 2′, such as with allylamine (12, 13 and 14) or dimethylamine (22 and 23) sidechains showed the best inhibition against SNM1A, while inhibitors with bulkier sidechains manifested a decrease in SNM1A potency. Interestingly introduction of an extra carbonyl group at position 2′ (as in amide 20), resulted in a 100-fold decrease in potency when compared to 13, while against SNM1B and SNM1C inhibitors 13 and 20 show inhibition with a similar range. Similarly, introduction of an ethoxy group at position 2′ caused a much steeper drop in potency against SNM1A, compared to SNM1B and SNM1C.

SNM1B and SNM1C can tolerate more bulky aromatic sidechains at position 2′, such as benzylamine (16) or furfurylamine (19) derivatives. 16 was the most potent identified SNM1C inhibitor with an IC_50_ of 1.1 μM; 19 was the most potent SNM1B inhibitor with an IC_50_ of 2.5 μM. In terms of selectivity, it is notable that 18, which has a diethylamine sidechain at the 2′ position, was only potent against SNM1C. Furthermore, based on results for compounds 22, 23 and 24, SNM1A and SNM1B likely do not tolerate groups larger than a halogen atom at the 6′ position, because the introduction of a methoxy group resulted in significant drop in potency (24), while for SNM1C all three compounds (22, 23 and 24) were similarly potent.

The overall results identify quinazoline–hydroxamic acids as broadly effective inhibitors of the human SNM1 family of nucleases. Importantly, the SAR studies show how small changes in active site binding elements can make large differences in the relative potency *versus* SNM1A/B/C, likely due to subtle differences in the precise active site architectures of the three enzymes.

### Biochemical analyses validating inhibition of SNM1A

To validate the inhibition of SNM1A, SNM1B, and SNM1C observed in the fluorescence-based screening assays, we then performed gel-based assays using 3′ terminally-labelled oligonucleotide substrates that enable both quantitative and qualitative (visual) assessment of the pattern of inhibition, for a subset of compounds. Importantly, whereas the fluorescence-based assay measures a single hydrolytic event at the substrate terminus, the gel-based nuclease assay informs on the pattern of digestion of the entire 51-mer substrate DNA over time. The gel-based assay results (Fig. 4 and ESI 10†) clearly show 19 inhibits each of SNM1A–C. 12, 13 and 20 exhibit good inhibition of SNM1A and SNM1B, whereas 21, which is a poor inhibitor of SNM1A, clearly inhibits the nuclease activity of SNM1C, with complete inhibition of digestion being observed at ∼6 μM of 21 (ESI Fig. 11A and B†). Interestingly, while the gel-based assay pattern of inhibition for most compounds correlated well with the data obtained for the fluorescence-based assay, compound 19 consistently exhibited relatively more potent inhibition of SNM1A in gel-based assays. We estimated the IC_50_ (obtained by direct quantification of ratio between intact substrate and digestion product) to be approximately 8 μM, as opposed to 44 μM in the fluorescence assay (ESI Fig. 10C†); the reason for this discrepancy is unclear, but nonetheless underscores the importance of direct visualisation of qualitative and quantitative effects of the inhibitors on nuclease reactions.

### Structural studies reveal mode of inhibition by quinazoline–hydroxamic acids

We used our high-resolution orthorhombic crystals of the SNM1A nuclease domain (aa 676–1040) as a soaking platform for inhibitor development involving analogues of 6. As was the case for the initial ELF hit compound soaking, crystals were back-soaked to remove malonate ions with or without 500 μM ZnCl_2_ to obtain the mono- and di-metal forms suitable for soaking different classes of inhibitors. We obtained structures for SNM1A complexed with three derivatives of 6 (13, 20, and 19) at resolutions ranging from 1.5 to 1.8 Å (Fig. 5A and ESI 9†). All of these compounds bind to SNM1A with very similar orientations. Their quinazoline ring is positioned to stack with D736, in an equivalent position to the adenine moiety in the SNM1B nucleotide complex.^26^ The hydroxamate group binds to both metal ions with its carbonyl oxygen coordinating with M1 and with its hydroxyl group bridging M1 and M2, the latter likely in a manner similar to the nucleophilic water. A hydrogen bond is formed between one of the carboxylate oxygens and D736 and the nitrogen of the hydroxamate moiety with all three compounds (Fig. 5B).

The R1 methyl group (Fig. 4) of the inhibitors is directed towards the side chains of H734 and S880. Two of the inhibitors (13 and 19) have a chlorine atom at the R3 position, which is positioned close (∼3.2 Å) to the side chain of S735 (Fig. 5B and C). The binding modes of the R2 substituents are more diverse, but all of them occupy a similar position close to K883, Y841 and G963. These three residues form the binding pocket of the 5′ phosphate of the substrate, which has been shown to be a key determinant of DNA binding and exonuclease activity for SNM1B.^26^ With all three inhibitors, the R2 groups are positioned in an equivalent position to the ribose ring of the nucleotide in the SNM1B nucleotide complex, but do not extend into the phosphate binding pocket itself (Fig. 5D). In the SNM1A complex with 19, weak electron density corresponding to apparent binding of a second molecule of 19 was observed; the second molecule of 19 is located within stacking distance of the first molecule and is positioned to form additional contacts with a symmetry related molecule in the crystal. Although intriguing given the observations with 1 and 2 (Fig. 2), this interaction is likely at least in part due to crystal lattice interactions.

### Cell-based assays reveal sensitization to ICL-inducing drugs

**Fig. 6:**
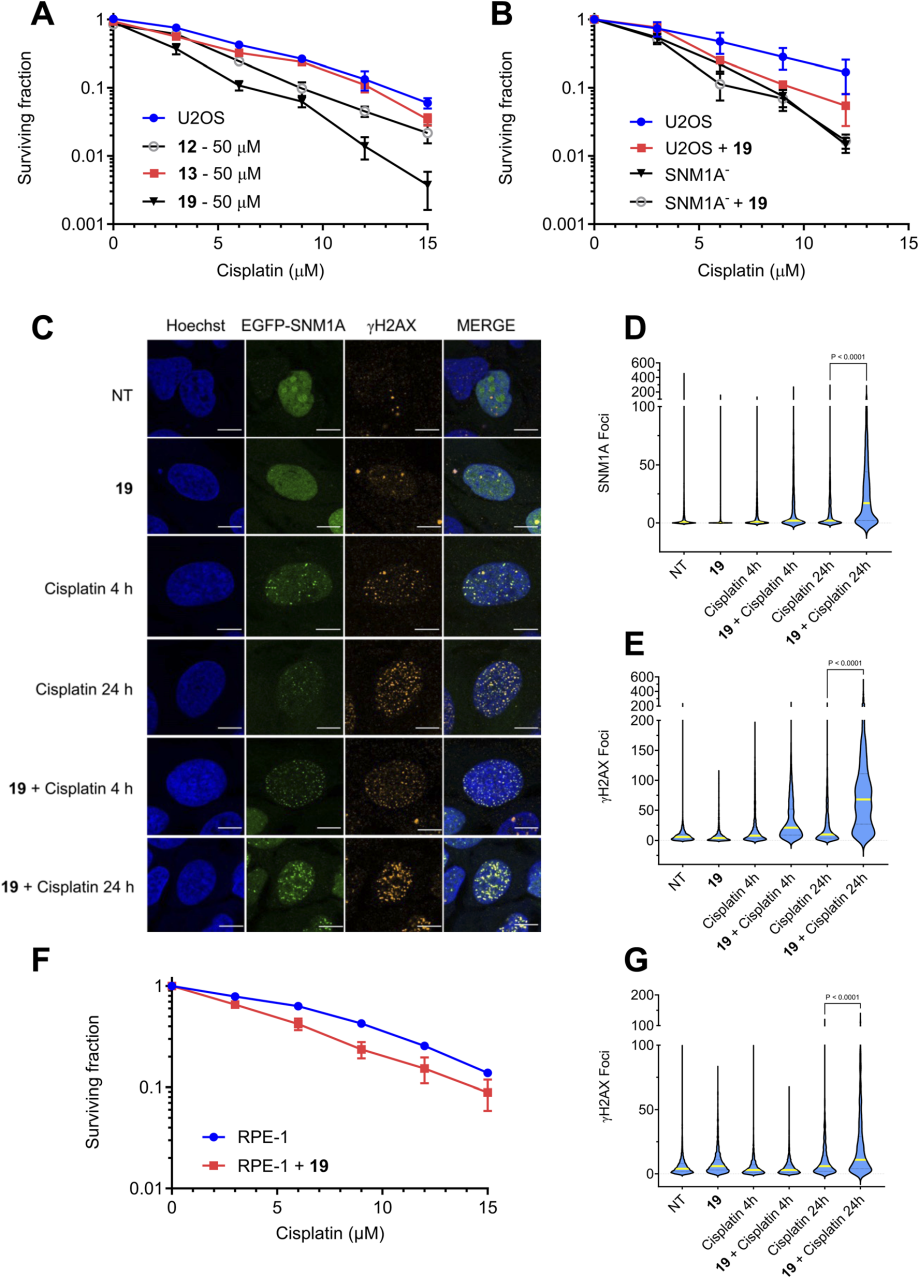
SNM1A inhibitor 19 sensitises cancer cells to cisplatin and delays resolution of cisplatin-induced DNA damage (A). Clonogenic survival assay showing the impact of 20 hours pre-treatment of U2OS (osteosarcoma) cells with 50 μM 12, 13, or 19 on the sensitivity a range of cisplatin concentrations. Data are the average of three repeats, error bars show the standard error of the mean. (B) Effect of 50 μM pretreatment with 19 on the cisplatin sensitivity of SNM1A^−^ cells generated in U2OS background in a clonogenic survival assay (the data for the parental U2OS cells used in panel A. is shown again to allow comparison of sensitivity of the different cell lines). Data are the average of three repeats, error bars show the standard error of the mean. (C) Representative images of U2OS cells stably expressing EGFP-SNM1A and treated with 50 μM cisplatin, 19 (50 μM) or a combination of these treatments. Cisplatin treatment induces γH2AX and EGFP-SNM1A foci in cells after 4 hours, that are resolved within 24 hours. Treatment with 19 delays the resolution of these foci at 24 hours (scale bar 10 μm). (D and E) Violin plots showing the distribution of foci counts for the experiments in C. (data represents three biological repeats counting >500 cells per repeat). (F) Effect of 19 (50 μM) on the sensitivity of RPE-1 cells to cisplatin in a clonogenic survival assay. (G) Violin plots showing the distribution of γH2AX foci counts in RPE-1 cells treated with 50 μM cisplatin, in the presence and absence of 50 μM 19. Data represents three biological repeats counting >500 cells per repeat.

A central aim of our SNM1 inhibitor development programme is to identify compounds that inhibit the cellular activity of SNM1-MBL fold proteins. Since SNM1A plays a key role in mediating the repair response to cisplatin induced DNA crosslinks, we investigated whether our SNM1A inhibitors potentiate cisplatin sensitivity, using three compounds selected from our *in vitro* analyses, that is 12, 13 and 19. None of these compounds were potently cytotoxic as single agents (ESI Fig. 12†), under the tested conditions, although some cell death was observed with 19 at 100 μM. Accordingly, for combination studies with cisplatin a fixed dose of all three compounds of 50 μM was selected, which in each case produced ≤10% cell death. The combination of these compounds with cisplatin (treatment 0–15 μM cisplatin) revealed potentiation of cisplatin cytotoxicity for 12 and 19, with the most striking interaction being apparent for 19 (which reaches statistical significance) (Fig. 6A). In order to investigate whether the sensitisation of U2OS cells reflects on-target effects of 19, we employed U2OS cells that have been gene edited to disrupt SNM1A, and where no SNM1A protein can be detected. While U2OS wild-type cells are sensitised to cisplatin treatment by 19, SNM1A^−^ cells were similarly sensitive to cisplatin treatment in the presence or absence of 19 (Fig. 6B). By contrast to wild-type U2OS cells (Fig. 6B), this suggests epistasis between cisplatin sensitivity in SNM1A^−^ cells in the presence and absence of inhibitor 19.

To explore this point more directly, we investigated whether U2OS cells treated with cisplatin and 19 exhibit the hallmarks of defects in SNM1A-mediated repair. To this end, cells stably expressing N-terminally EGFP-tagged SNM1A (EGFP-SNM1A) were treated with 50 μM cisplatin alone, 19 (50 μM), or with a combination of both compounds. It is known that on treatment of cells with DNA crosslinking agents, SNM1A forms subnuclear foci that are associated with ongoing sites of DNA repair.^37^ Treatment with 50 μM 19 alone did not induce EGFP-SNM1A repair-associated foci, or foci of a common marker, *i.e.* a phosphorylated form of histone variant H2AX (γH2AX) used to mark sites of DNA breakage and repair (Fig. 6C, quantified in Fig. 6D and E). As previously established, cisplatin treatment efficiently induces co-localisation of EGFP-SNM1A foci and γH2AX foci within 4 hours following treatment, marking sites of cisplatin damage and its repair (Fig. 6C–E). These largely resolved 24 hours post-treatment, again consistent with previous studies.^16,37^ Co-treatment of cells with 19 and cisplatin saw a robust induction of SNM1A foci, consistent with the SNM1A activity being dispensable for its localisation to sites of crosslink repair, and γH2AX foci formation (Fig. 6C–E). In the presence of 19 both classes of colocalising foci persisted at 24 hours. This clear persistence of repair intermediates and trapping of EGFP-SNM1A at the sites of these repair intermediate implies that 19 engages with SNM1A in a cellular context, delaying the completion of cisplatin crosslink repair, which, in turn, results in the sensitisation of cells to cisplatin.

Finally, we explored the response of genetically stable and karyotypically normal immortalised (non-cancer) cells to 19, in the presence and absence of cisplatin. As for the U2OS cells, RPE-1 cells (hTert-immortalised retinal pigment epithelial cells) treated with 19 alone exhibited a mild toxicity only at the highest dose employed (100 μM), where a negligible reduction in survival was observed at 50 μM (ESI Fig. 12†). For RPE-1 cells treated with cisplatin, the inclusion of 50 μM 19 did not substantially increase their dose-dependent sensitivity of cisplatin (Fig. 6F), compared to the vehicle-alone control. Moreover, while the number of γH2AX foci persisting in RPE-1 cells 24 hours after 50 mM cisplatin treatment was elevated in the presence of 50 μM 19 relative to the vehicle-alone control (Fig. 6G), this increase was less dramatic than that observed with U2OS cells. The average number of γH2AX foci in cisplatin treated U2OS at 24 hours increases 4-fold following pretreatment with 19 (21.5 ± 0.5 to 81.6 ± 1.7 foci). By contrast in RPE-1 cells, the average γH2AX foci count only increased 2-fold under the same conditions (10.4 ± 0.3 to 21.2 ± 0.7 foci). Together, these observations imply a cancer cell-selective increase in cisplatin sensitivity associated with DNA damage persistence might be achievable through inhibition of SNM1A by small molecule inhibitors.

## Discussion

Nucleases play essential roles in all life forms and are vital tools in the manipulations of molecular biology, *e.g.* CRISPR mediated gene-editing. It is therefore striking that drugs to target nucleases have not been pursued more extensively. The lack of work on inhibiting nucleases contrasts with many other enzyme superfamilies, including other hydrolytic enzymes such as proteases and related enzymes including β-lactamases. The reasons for the apparent paucity of work on the modulation of nuclease activity are not clear, but may reflect the normally tight regulation of nuclease activity in cells, difficulties in preparing some nucleases and/or appropriate substrates, and challenges in obtaining selective inhibitors. In the case of the Zn(ii) dependent bacterial ‘true’ MBLs (that hydrolyze β-lactam antibiotics) extensive studies from multiple groups have demonstrated their viability as drug targets, with highly potent and selective inhibitors having been developed, including those resulting from hits identified from high throughput screening.^11^ By contrast, there are few studies on inhibition of the structurally and mechanistically related MBL fold nucleases.^12–14^

The results presented here demonstrate the viability of developing highly potent and selective inhibitors of the human SNM1A–C nucleases which play vital roles in DDR, and which are targets for cancer treatment. To identify scaffolds suitable for SNM1 inhibition, we carried out a HTS employing a fluorescence-based assay^38^ and the ELF compound collection. This approach has previously delivered new types of inhibitors for the clinically relevant family of di Zn(ii) ion dependent B1 subfamily of the true MBL NDM-1 and related B1 subfamily MBLs;^11^ MBL inhibitor types identified include the indole carboxylates, which bind to the active site Zn(ii) ions in a manner that stabilises, but which does not displace the hydrolytic water, which (at least in the resting enzyme) bridges between the two Zn(ii) ions; the indole carboxylate scaffold, however, does not inhibit the SNM1 nucleases.^11^ The ELF screen identified several SNM1A hit pharmacophores that we considered suitable for further exploration as SNM1A inhibitors, including by structural studies involving both the mono- and di Zn(ii) forms of SNM1A, which were used because the precise nature of the metal ions at the SNM1 active sites *in vivo* is unknown.

Initially, we focused on the cyclic hydroxyimide hits 1–3 as SNM1A inhibitors, in part because related compounds have been shown to inhibit the nuclease FEN1 (flap structure specific endonuclease 1), which like SNM1A is also involved in DNA damage repair pathways.^35,36^ The *N*-hydroxyimides 1–3 were shown by crystallography to bind to the active site metal ions in a manner that displaces the hydrolytic water/hydroxide and which will compete with the substrate (at least in the active site region). Although, there is likely scope for future development of cyclic *N*-hydroxyimides as SNM1 inhibitors, the insolubility of the tested compounds coupled with the mechanistically interesting, but complicating, crystallographic observation that, at least in cases, two molecules of the cyclic *N*-hydroxyimide can bind at the active site, prompted us to explore other pharmacophores from the ELF screen, in particular quinazoline-containing inhibitors (6, and related compounds), where 6 was a moderately potent inhibitor, but which appeared more amenable to SAR studies than the cyclic *N*-hydroxyimides. Based on analysis of the SNM1A crystal structures obtained with 1–3, we substituted the C2 carboxylic acid of the hit quinazoline inhibitors with a hydroxamic acid, a known pharmacophore for the true MBLs,^39^ a modification that led to increased potency of SNM1A inhibition. Subsequent SAR studies involving modifications at the C4 quinazoline position enabled the identification of potent SNM1A inhibitors, as shown by both fluorescence and gel-based assays (Fig. 4B). Interestingly a combination of a thioxodihydroquinazolinone and cisplatin has been reported to work synergistically in cisplatin resistant cells and shows promising results in a mouse model.^40,41^ Although, the thioxodihydroquinazolinones are structurally distinct from the quinazoline-containing inhibitors reported here, it cannot be ruled out that they, or metabolites of them, act as SNM1 inhibitors *in vivo*.

Crystallographic analyses on three of the quinazoline–hydroxamate inhibitors (13, 19 and 20) reveal that they bind in similar manner to β-lactam antibiotics binding to true MBLs, with the quinazoline ring binding in a manner equivalent to that of an adenine ring observed in an SNM1B nucleotide complex structure^26^ (ESI Fig. 13†). The quinazoline C4 linked hydroxamate is positioned to interact with the M1 and M2 metal ions of SNM1A, with its carbonyl oxygen coordinating to M1 and its hydroxyl/hydroxide group occupying the M1 : M2 bridging position which is occupied by the hydrolytic water/hydroxide during catalysis.

Hydroxamic acids have been developed for clinical use as histone deacetylase inhibitors,^42^ though such compounds also have potential to inhibit other human metallo-enzymes^43^ and to act as non-selective metal ion chelators. Hence appropriate derivatisation of them is required to enable selectivity, which in the case of SNM1 inhibitors includes against other human MBL fold nucleases such as CPSF73.^28^ Although there is clearly scope for further optimisation of the inhibitors reported here (*e.g.* cyclic hydroxamic acids and use of other Zn(ii) chelating pharmacophores), the results comparing inhibition of all three of SNM1A, B and C are interesting with respect to selectivity. Whilst many of the hydroxamic acids inhibited all three human SNM1 nucleases with comparable efficiency, the results clearly indicate developing inhibitors selective for the individual isoforms should be possible, including *via* modification of the quinazoline 2′ and 6′ positions. Thus, SNM1B and SNM1C can tolerate larger groups at the 2′ position than SNM1A and SNM1A and SNM1B are less tolerant of substitution at the 6′ position than is SNM1C.

We tested selected compounds for evidence that they can inhibit SNM1A in cells. It is well-established that SNM1A is a key mediator of DNA crosslink repair, and that SNM1A deficient cells are sensitive to drugs that induce DNA crosslinks, such as cisplatin.^16,37^ We observed cancer cell sensitisation when treatment with cisplatin treatment was combined with 19. A chemical-genetic approach, employing matched, engineered SNM1A^−^ cells demonstrated that the sensitisation of U2OS cells is dependent on the presence of SNM1A, implying that the observed sensitisation is (at least in part) due to on-target engagement by 19. To more directly examine this, we employed imaging approaches, the results of which revealed that EGFP-SNM1A is efficiently recruited to sites cisplatin of damage and repair as marked by γH2AX foci. Importantly, upon co-administration of cisplatin and 19, SNM1A persisted at such sites, indicating that repair is compromised. The recruitment of SNM1A to crosslinks is known to be independent of its catalytic activity and to be mediated by interaction of SNM1A with PCNA (proliferating cell nuclear antigen) *via* the conserved SNM1A PIP box motif and with the ubiquitin-modified form of PCNA (PCNA^Ub^) mediated by a ubiquitin-binding zinc finger in the N-terminus of SNM1A. Together, our cellular data indicate that 19 likely competes with repair substrates for SNM1A, stalling the step in crosslink repair that is mediated by SNM1A as evidenced by persistence of SNM1A at sites of cisplatin damage that are also marked by the DNA repair intermediate marker, γH2AX.

Interestingly, compared with U2OS cells an immortalised (non-cancer) cell line (RPE-1) exhibited a less dramatic senstisation to cisplatin in combination with 19 treatment. Moreover, a concomitantly lower fold increase in persistent γH2AX foci was observed. This observation suggests that when additional DNA damaging genomic stress is simultaneously administered pharmacologically, tumour-derived cells might be more vulnerable to SNM1A loss than non-cancer cells. The reasons for this require further investigation with one possibility being additive effects of SNM1A inhibition and underlying defects in the machinery maintaining genome stability in cancer cells, and/or the presence of more robust cell cycle checkpoints in non-cancer cells preventing unrestrained entry into S-phase in the presence of cisplatin damage.

## Conclusion

The combined SAR results from fluorescence and gel-based biochemical assays coupled with cellular studies demonstrate the viability of selective inhibition of SNM1A and, by implication, SNM1B/C in a cellular context. Cellular assays reveal that SNM1A inhibitors cause sensitisation to, and defects in the resolution of, cisplatin-induced DNA damage. Future structure guided work can build on the results presented here to develop inhibitors suitable for investigating the validity of SNM1A as a target for cancer treatment. Importantly, the results demonstrate that it should be possible to employ structural data to develop SNM1 isoform selective inhibitors.

## Methods

### Purification of SNM1A, SNM1B, SNM1C

DNA encoding for the MBL and β-CASP domains of SNM1A (aa 676–1040) was cloned into the baculovirus transfer vector pFB-LIC-Bse; for the MBL and β-CASP domains of SNM1B (aa 1–335) into baculoviral expression vectors pFB-CT10HF-LIC (GenBank EF199842); and for the MBL and β-CASP domains of SNM1C (aa 1–362) into the baculovirus expression vector pBF-6HZB (GenBank™ accession number KP233213.1). Baculovirus generation was performed as described.^44^ Recombinant proteins were produced by infecting Sf9 cells at 2 × 10^6^ cells per mL with 3.0 mL L^−1^ of P2 virus for SNM1A and SNM1B and 1.5 mL L^−1^ of P2 virus for SNM1C respectively. Infected Sf9 cells were harvested 70 h after infection by centrifugation (900×*g*, 20 min). The cell pellets were resuspended in 30 mL L^−1^ lysis buffer (50 mM HEPES pH 7.5, 500 mM NaCl, 10 mM imidazole, 5% (v/v) glycerol and 1 mM tris(2- carboxyethyl)phosphine (TCEP), rapidly frozen in liquid nitrogen, then stored at −80 °C. Purifications of recombinant SNM1A, SNM1B and SNM1C were performed as described.^26,29,30^

### Generation of 3′-radiolabelled substrates

3′-Radiolabelled substrates were made by labelling 10 pmol of single-stranded DNA (Eurofins MWG Operon, Germany) with 3.3 pmol of α-^32^P-dATP (PerkinElmer) by incubation with terminal deoxynucleotidyl transferase (TdT, 20 U; ThermoFisher Scientific), at 37 °C for 1 hour, before being passed through a P6 Micro Bio-Spin chromatography column (BioRad), as reported. See ESI Table 1† for oligonucleotide sequences used in this work.

### Gel-based nuclease assays

Standard exonuclease assays were carried out in a 10 μL final volume containing 20 mM HEPES-KOH, pH 7.5, 50 mM KCl, 0.5 mM TCEP, 10 mM MgCl_2_, 0.05% (v/v) Triton X-100, 5% (v/v) glycerol, and SNM1 proteins as indicated. Inhibitors were dissolved in DMSO and diluted immediately prior to the reaction in the above buffer. Inhibitors were serially diluted two-fold from 100 μM. The DMSO concentration was kept constant at 0.1% (v/v) in the final reaction mixture. Inhibitors were incubated with the indicated SNM1 enzyme for 10 minutes at room temperature; reactions were initiated by addition of 10 nM DNA substrate and incubated at 37 °C for 20 min. Reactions were quenched by addition of 5 μL stop solution (95% formamide, 10 mM EDTA, 0.25% (v/v) xylene cyanol, 0.25% (v/v) bromophenol blue) and heated at 95 °C for 3 min. Reactions were analysed by 20% denaturing polyacrylamide gel electrophoresis solution of 19 : 1 acrylamide:bis-acrylamide, BioRad and 7 M urea (Sigma Aldrich) in 1 × TBE (Tris-borate EDTA) buffer. Electrophoresis was carried out at 525 V for 75 minutes; gels were subsequently fixed for 60 minutes in a 50% (v/v) methanol, 10% (v/v) acetic acid solution, and dried at 80 °C for two hours under vacuum. Dried gels were exposed to a Kodak Phosphor imager screen and scanned using a Typhoon 9500 instrument (GE Healthcare), scanned images were quantified using ImageJ.

### Fluorescence-based nuclease digestion assays

Fluorescence-based nuclease digestion SNM1A–C assays were performed as described.^30,45^ In the case of SNM1A and SNM1B, a 20-mer oligonucleotide with a 5′ FITC-conjugated-T and an internal BHQ-1 (black hole quencher) conjugated-T, eight nucleotides distal (3′) to the fluor was utilised; whereas for SNM1C a 20-mer oligonucleotide with a 5′ FITC-conjugated-T and a 3′ BHQ-1(black hole quencher) was used to capture all possible endonucleolytic incisions. Reactions (25 μL) were performed in 384 well microplates in nuclease buffer (20 mM HEPES-KOH pH 7.5, 50 mM KCl, 10 mM MgCl2, 0.5 mM TCEP, 0.05% (v/v) Triton-X, 0.1 mg mL^−1^ BSA, 5% (v/v) glycerol) with SNM1A/B/C (0.1 nM SNM1A, 0.5 nM SNM1B and 1 nM SNM1C) as indicated. Proteins were incubated for 10 min at RT with the stated compound where applicable, before the reaction was initiated with the addition of 10 nM DNA substrate. Fluorescence was measured using a PHERAstar FSX fluorescent plate reader in fluorescent top read mode using excitation at 495 nm, emission at 525 nm, and cutoff at 151 nm. Fluorescence spectra were measured every 140 s, for 35 min, at 37 °C. The fluorescence intensity of each well was plotted against compound concentration and fitted using a log (inhibitor) response curve using GraphPad Prism software (GraphPad Software, Inc., La Jolla, CA, USA).

### Crystallization and structure determination

SNM1A crystallization was performed by vapour diffusion in sitting drops at 4 °C. A protein solution at 9–10 mg mL^−1^ was mixed at with an equal volume crystallization solution containing 30% (v/v) PEG 1000, 0.1 M MIB pH 6.0 (MIB is sodium malonate dibasic monohydrate, imidazole, boric acid). The crystals contained malonate bound to the active site (Fig. S1†). For compound soaking experiments, crystals were first incubated overnight in malonate-free liquor (30% (V/V) PEG, 0.1 M HEPES, pH 7.0) with and without addition of ZnCl_2_ (500 μM). European Lead Factory (ELF) derived compounds for crystal soaking studies were dispensed using an Echo dispenser at 10% v/v from 10 mM stocks at the X Chem facility at Diamond Light Source. Crystals were harvested after 1–4 hours soaking, then flash-cooled in liquid N_2_ without addition of a cryoprotectant. In house synthesized compounds were soaked overnight at a final concentration of approximately 10 mM before harvesting and cryo-cooling. Data were collected at Diamond Light Source beamlines I04-1 (Fragments), I04 and I03. Crystallography statistics are provided in ESI Table 2.†

### Generation of SNM1A knock out cell lines

Genome editing technologies were employed to make stable deletion–disruptions in *DCLRE1A*, the gene encoding for SNM1A, in U2OS osteosarcoma cells. These have previously been described in detail in Swift *et al.*, bioXriv, 2022, doi.org/10.1101/2022.07.21.500940. These U2OS cells contain a 22-nucleotide deletion in exon 1 of DCLRE1A, producing a frameshift and premature stop codon distal from the deletion site; no SNM1A protein was detected by immunoblotting in these cells. RPE-1 cells were kindly provided by Andrew Blackford (University of Oxford).

### Clonogenic survival assays

Clonogenic assays were performed in 10 cm tissue culture dishes (for RPE-1 cells, U2OS cells and derived clones). Cells (1000 per dish) were seeded in complete media (10 mL) and allowed to attach overnight, then treated with cisplatin or cisplatin plus inhibitor for the times stated. Following treatments, cells were allowed to grow and form colonies for 10 days. Colonies formed were stained with Coomassie R250 (Sigma) and counted on a COLCount Colony Counter (Oxford Optronix). All experiments represent the mean (±SEM) of at least three biological repeats of duplicate dishes/flasks for each treatment.

### Microscopic analysis of damage-induced subnuclear foci

To assess the number of nuclear foci following cisplatin/inhibitor treatments, cells were plated in glass bottom dishes (as above) and allowed to attach. Following treatment, cells were fixed with 4% (v/v) formaldehyde in PBS, blocked with immunofluoresence (IF) blocking buffer (5% horse serum, 1% saponin in PBS) for 1 h, then incubated with primary antibodies at the desired concentrations in IF blocking buffer (overnight, 4 °C). After washing cells three times with PBS, cells were incubated with secondary antibodies for 2–4 hours in IF blocking buffer, washed a further 3 times with PBS then stained with Hoechst 333 258 (1 μg mL^−1^ in PBS) for 30 minutes before a further three washes (all washes and incubations were performed at room temperature, unless otherwise stated).

Confocal images were obtained with a Plan APO 63X 1.40NA oil immersion objective, a pinhole setting of 1 AU, bandpass emission settings of 410–468 nm for Hoechst, 490–544 nm for EGFP, 579–624 nm for RFP or Alexa 568, and 633–695 nm for Alexa 647, a projected pixel dimension of around 110 nm × 110 nm, a pixel dwell time of 1.35 μs, and with a line averaging setting of 2. In order to ensure sufficient cell numbers (*N* > 300), images were acquired in a tiled 5 × 5 format corresponding to an image area of about 0.65 mm × 0.65 mm. Images were imported into ImageJ and foci were counted using a macro adjusting for staining levels between experiments. Anti-γH2AX antibodies were a mouse monoclonal (Millipore; JBW301).

## Data availability

X-ray structures been deposited in the PDB database (https://www.rcsb.org/) with codes 8C8S, 8C8D, 8C8B, 8GC9, 8CF0, 8CEW and also 6 previously deposited structures from the PDB database with codes 5FV7, 5NZW, 5Q7C, 7A1F, 7APV and 4HL2.

## Author contributions

PSJ, SPM, AM, MS, SvB, EvD, JvD and HvdH performed the high-throughput screen and initial validation of hits. M. Bielinski, LRH, YY, HTB, LPS, M. Bowen, JB and JN performed all other experiments. OG, PM and CJS designed the project, supervised the experiments and wrote the paper with inputs from all authors.

## Conflicts of interest

The authors declare no competing interests.

## Supplementary Material

SC-015-D4SC00367E-s001
