# Supplementary material for: Cell-active small molecule inhibitors validate the SNM1A DNA repair nuclease as a cancer target
Source: Chem Sci. 2024 Apr 30;15(21):8227–41. doi: 10.1039/d4sc00367e (PMC11134331; doi:10.1039/d4sc00367e)
Supplement: SC-015-D4SC00367E-s001 [file SC-015-D4SC00367E-s001.pdf]

Electronic Supplementary Material (ESI) for Chemical Science.
This journal is © The Royal Society of Chemistry 2024

# Supplementary Information

# Cell-active small molecule inhibitors validate the SNM1A DNA repair nuclease as a cancer target

Marcin Bielinski[1,†], Lucy R. Henderson[2,†], Yuliana Yosaatmadja[3,¶], Lonnie P. Swift[2], Hannah T. Baddock[2, ‡], Matthew J. Bowen[1], Jürgen Brem[1], Philip S. Jones[4,#], Stuart P. McElroy[4,#], Angus Morrison[4,#], Michael Speake[4,#], Stan van Boeckel[5], Els van Doornmalen[5], Jan van Groningen[5], Helma van den Hurk[5], Opher Gileadi[3, ††], Joseph A. Newman[3*], Peter J. McHugh[2*], and Christopher J. Schofield[1*].

* To whom correspondence should be addressed:

email: christopher.schofield@chem.ox.ac.uk;

email: peter.mchugh@imm.ox.ac.uk;

email: joseph.newman@cmd.ox.ac.uk.

[1] Chemistry Research Laboratory, Department of Chemistry and the Ineos Oxford Institute for Antimicrobial Research, University of Oxford, Mansfield Road, Oxford OX1 3TA, United Kingdom.

[2] Department of Oncology, MRC Weatherall Institute of Molecular Medicine, University of Oxford, John Radcliffe Hospital, Oxford OX3 9DS, United Kingdom.

[3] Centre for Medicines Discovery, NDM Research Building, University of Oxford, Old Road Campus Research Building, Roosevelt Drive, Oxford OX3 7DQ, United Kingdom.

[4] University of Dundee, European Screening Centre, Newhouse, ML1 5UH, United Kingdom.

[5] Pivot Park Screening Centre, 5349 AB Oss, the Netherlands.

[†]The contributions of these authors should be considered equal.

[¶] Current address: School of Biological Sciences, Building 110, 3A Symonds Street, Auckland 1010, New Zealand.

[‡]Current Address: Calico Life Sciences, 1170 Veterans Blvd, South San Francisco, CA 94080 USA.

[†]Current address: SGC Karolinska, Center for Molecular Medicine (CMM), Karolinska University Hospital, 171 76 Stockholm, Sweden.

[#]Present Address: BioAscent Discovery, Newhouse, ML1 5UH, United Kingdom

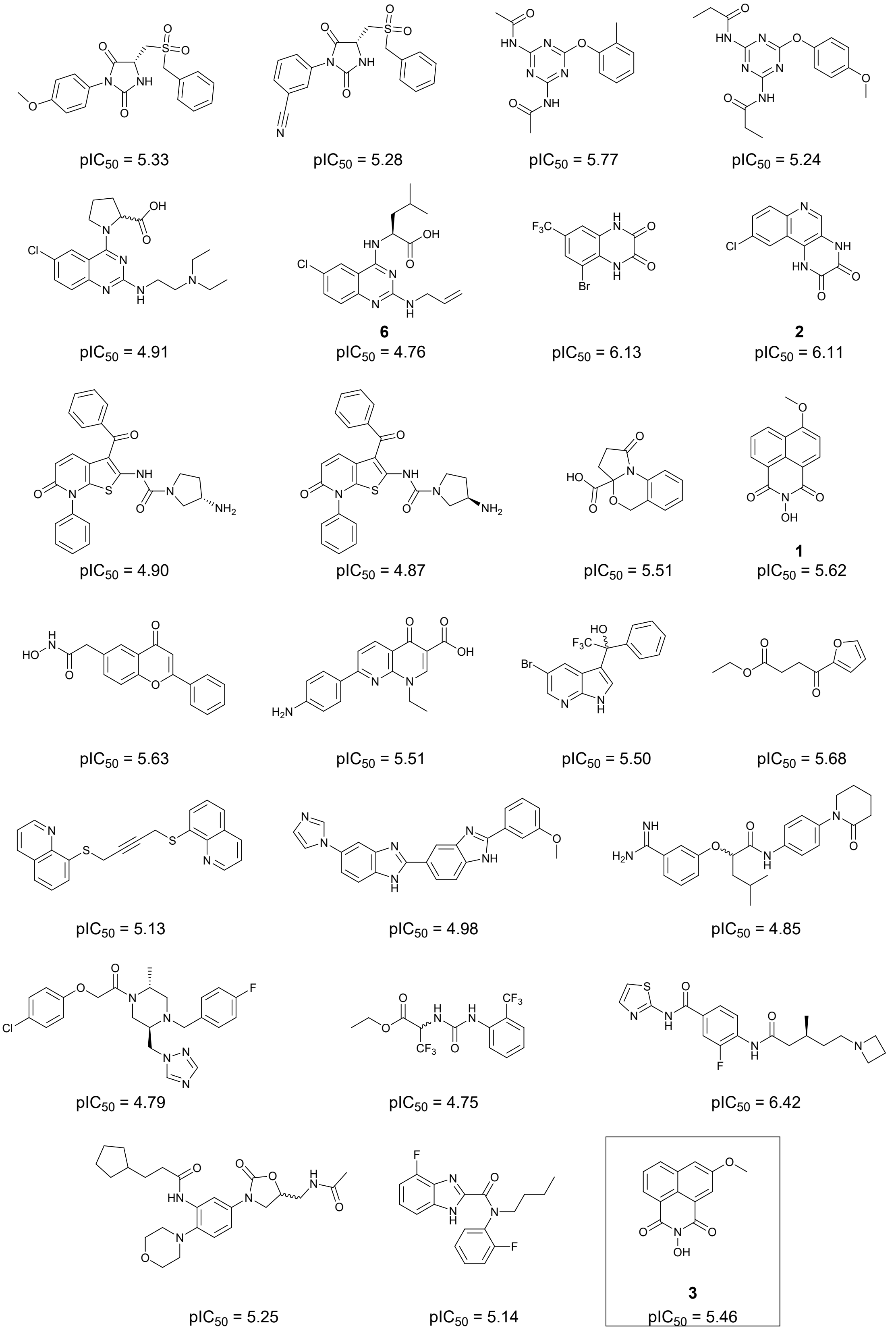


**Suppl. Figure 1:** Results of the high throughput screen for SNM1A inhibitors. The 24 hit molecules were selected from a European Lead Factory (ELF) public-private partnership high-throughput screen of >302, 000 compounds in 384 well plate format over 3 days using the fluorescence based described in the Methods. 7-Aminocephalopsoranic acid was used as a positive control ($IC_{50}$ 12.2 µM). Day 1: 60143 compounds screened in 47 plates, mean Z' 0.747 (0.630 – 0.820; Day 2: 111154 compounds screened in 87 plates, mean Z' 0.80 (0.85 – 0.91. Day 3: 131448 compounds screened in 87 plates, mean Z' 0.86 (0.73 – 0.90. In total, 824 compounds manifested a Z score <-4. Full details will be published elsewhere. Overall, 19 structural clusters were identified of which 14 were singleton clusters. Note, the ELF reported identified the structure of **1** as that shown in the figure but the correct structure of the screened compound is likely **3**. (see main text for details). Note although **6** (K1) is not a potent SNM1A inhibitor hydroxamic acid derivatives of it are more potent (see Figure 3 and main text for details).

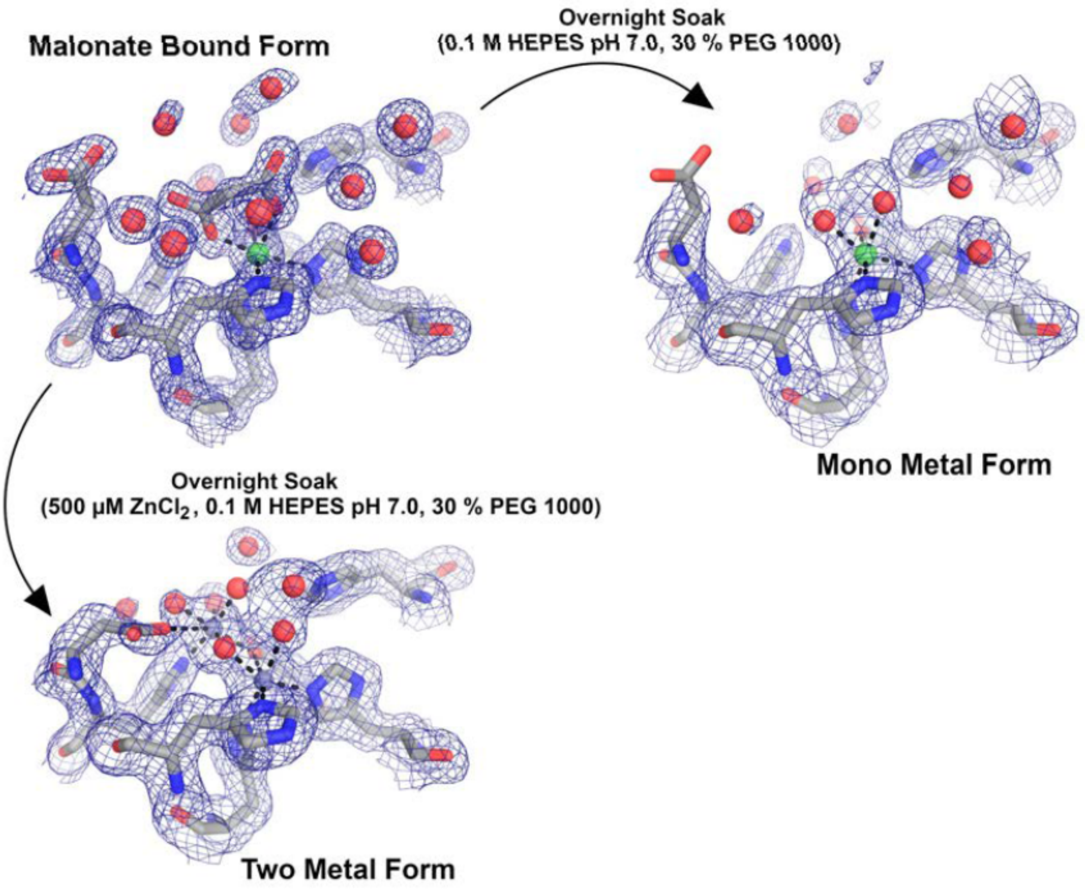


**Suppl. Figure 2.** Electron density maps (2Fo-1Fc contoured at 1σ) showing the active site environment of SNM1A prior to soaking (top left malonate form; PDB 5Q7C) and after soaking in the absence or presence of $ZnCl_2$ (top right and bottom left respectively) to produce crystals with one and two metal ions in the active site suitable for compound soaking experiments.

**A**

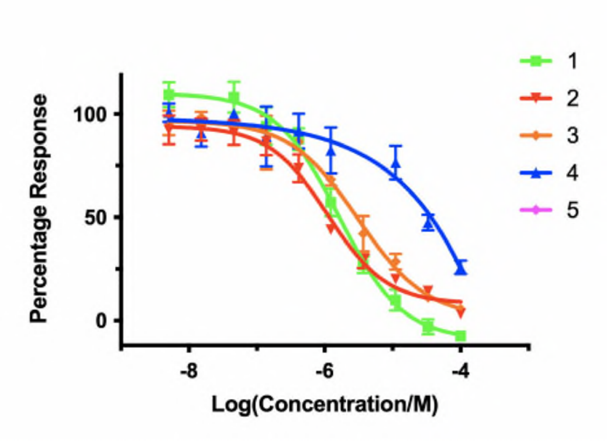


**B**

| Compound | Structure | IC$_{50}$ / µM |
|---|---|---|
| **1** | 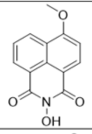 | 2.4 |
| **2** | 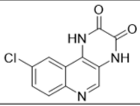 | 2.0 |
| **3** | 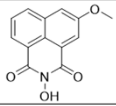 | 2.9 |
| **4** | 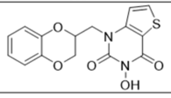 | n.d. |
| **5** | 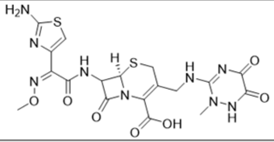 | 32 |

**Suppl. Figure 3.** (**A**) Effects of compounds **1-5** on the exonuclease activity of SNM1A measured *via* a real-time fluorescence-based nuclease assay, shown as mean ± SEM of four independent repeats. (**B**) Table showing IC$_{50}$ values of compounds **1-5** tested against SNM1A.

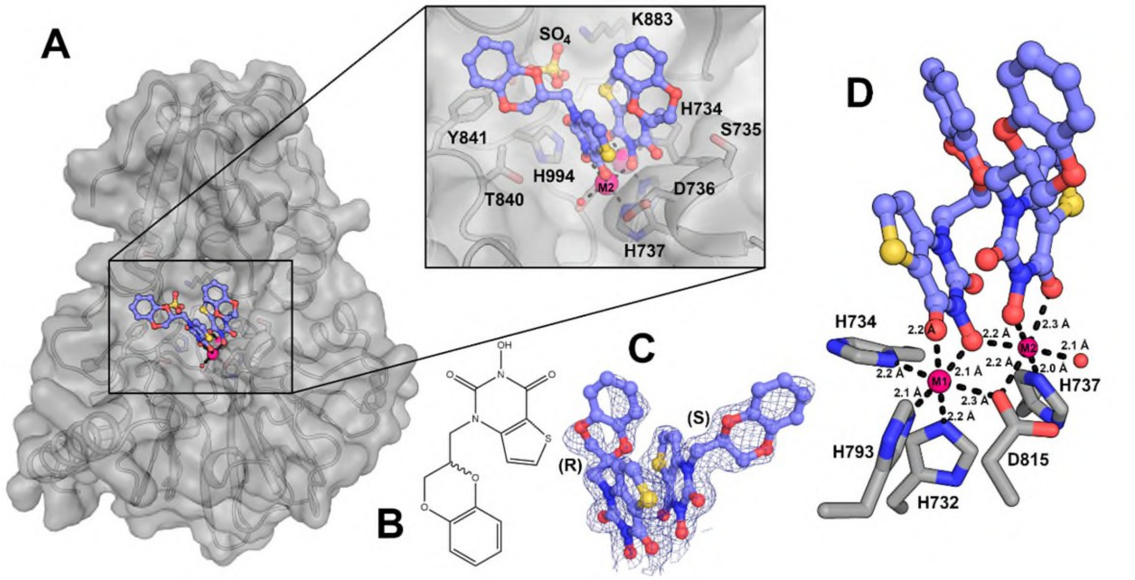


**Suppl. Fig. 4.** Structural characterization of AZ1353160 (**4**) binding to SNM1A. (**A**) Surface representation of SNM1A in complex with AZ1353160 (PDB 8GC9) (**4**); the inset shows a closeup view with interacting residues labelled. (**B**) Structure of AZ1353160 (**4**). (**C**) 2Fo-1Fc electron density maps contoured at 1σ in the vicinity of AZ1353160 (**4**) with the stereochemistry of two enantiomers labelled. (**D**) Close-up view of the interaction of AZ1353160 (**4**) with the metal ions in the SNM1A active site. Metal ions are shown as pink spheres with key residues labelled and coordination distances shown.

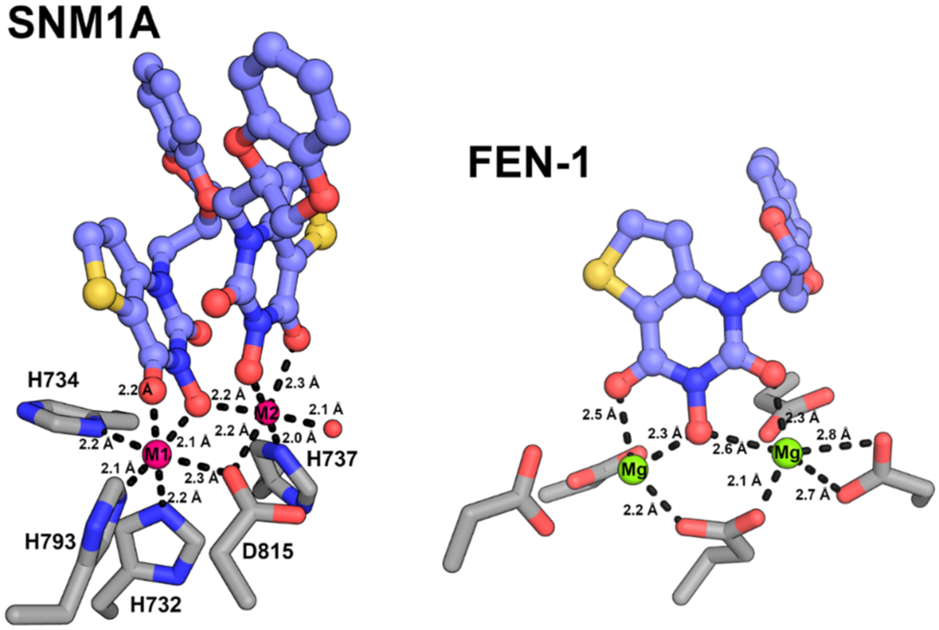


**Suppl. Fig. 5.** Comparison of the mode of metal ion coordination modes observed for AZ1353160 with SNM1A (on the left, PDB 8GC9) and FEN1 (on the right, PDB 5FV7). Metal ligand interactions are shown as black dashed lines with interaction distances labelled (two magnesium ions are present at the FEN1 active site).

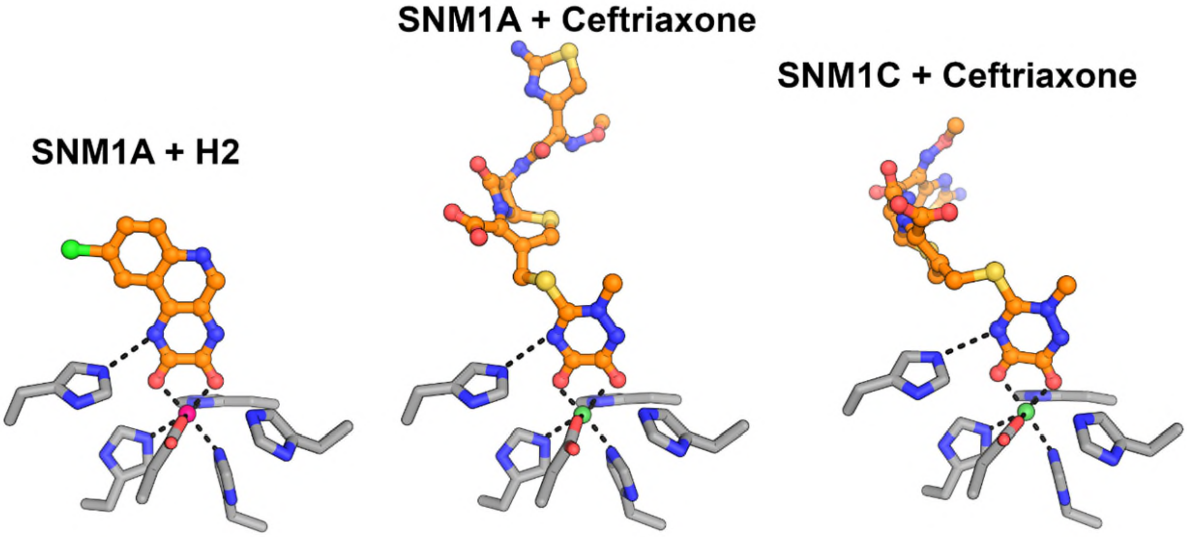


**Suppl. Figure 6.** Comparison of the mode of interaction of compound **2** (on the left in complex with SNM1A, PDB 8CF0) with the catechol moiety in ceftriaxone in complex with SNM1A (centre, PDB 5NZW) and SNM1C (right, PDB 7APV).

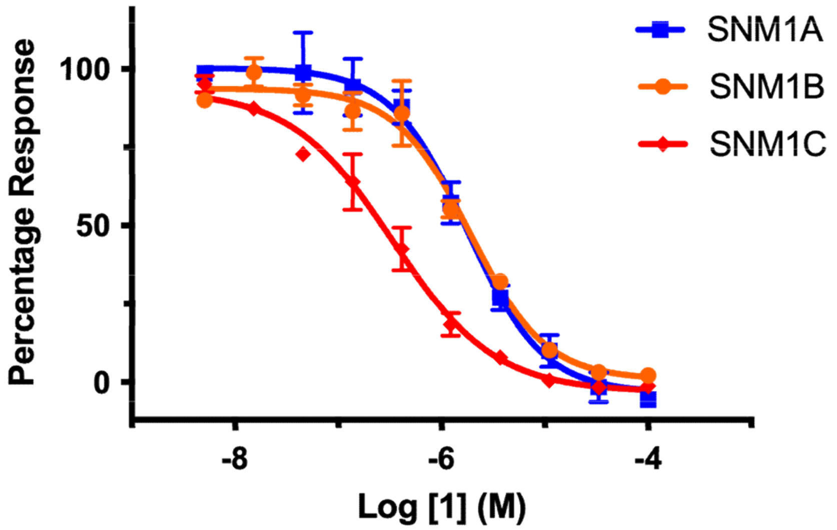


| Enzyme | $IC_{50}/\mu M$ |
|---|---|
| SNM1A | 2.4 |
| SNM1B | 1.9 |
| SNM1C | 0.37 |

**Suppl. Fig. 7.** Comparison of the effects of compound **1** on SNM1A, SNM1B and SNM1C's nuclease activity measured *via* a real-time fluorescence-based nuclease assay, shown as mean ± SEM of four independent repeats.

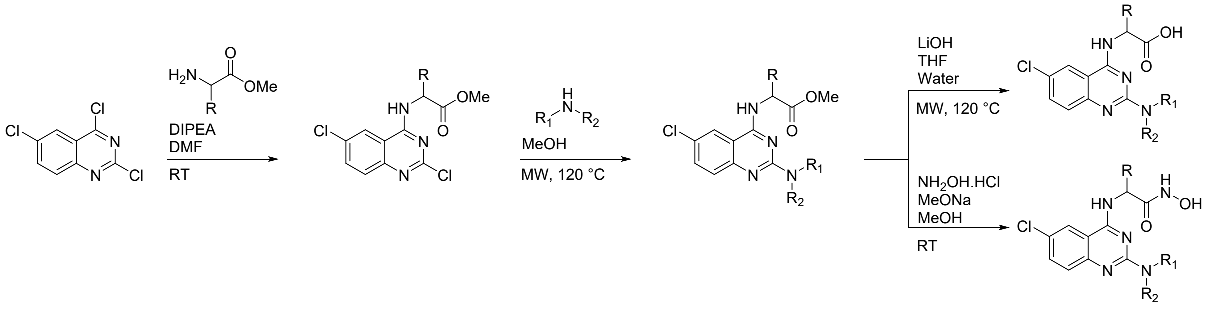


**Suppl. Fig. 8**. for synthesis of inhibitor **6** and its analogues.

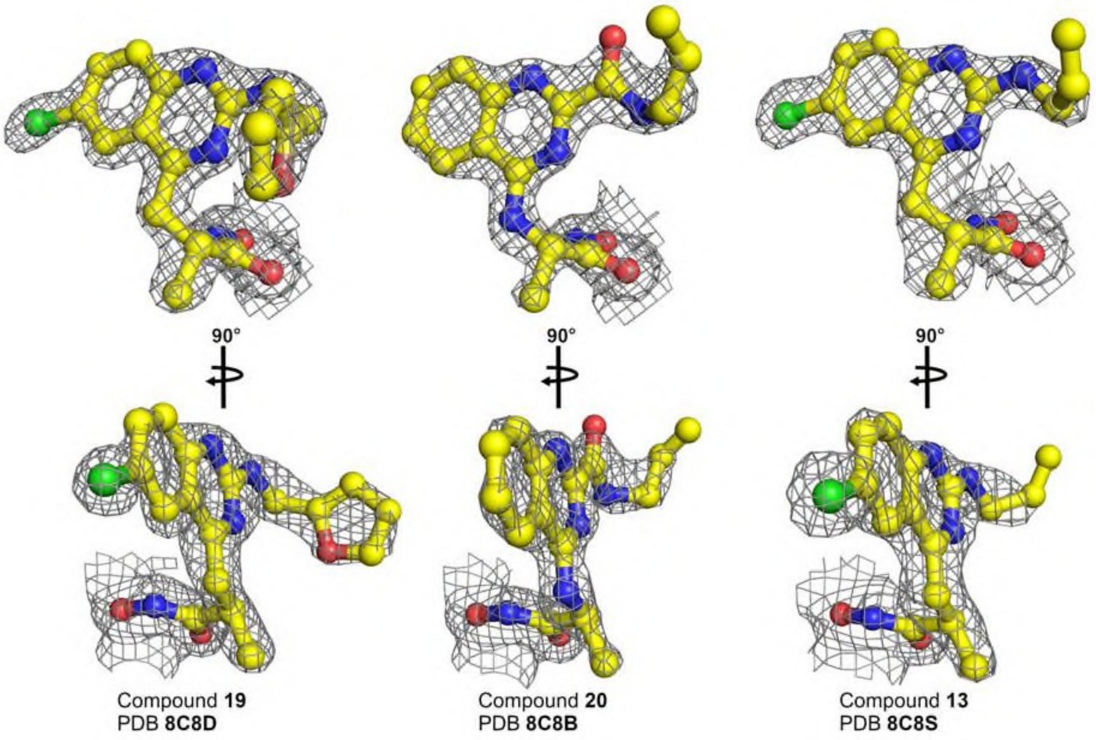


**Suppl. Figure 9**. 2Fo-1Fc Electron density maps for the 3 hydroxamic acid inhibitors (**19, 20, 13;** PDB 8C8D, 8C8B and 8C8S, respectively) complexed with SNM1A. Maps are contoured at 1 σ.

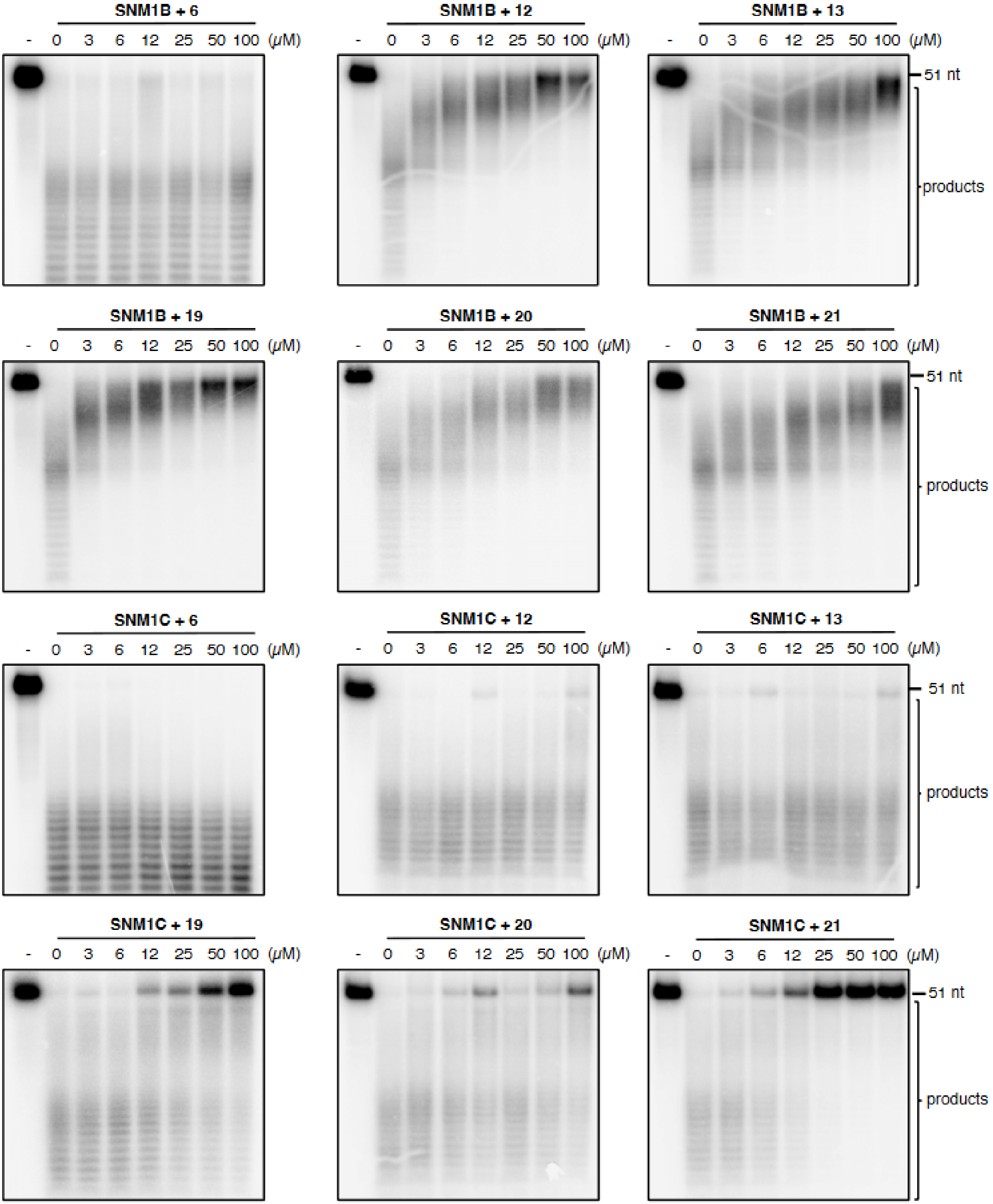


**Suppl. Figure 10.** Increasing concentrations (as indicated, in µM) of compounds incubated with 1 nM SNM1B or 2.5 nM SNM1C (room temperature, 10 min), before initiating nuclease reaction by addition of ssDNA (37 °C, 20 min). Products were analysed by 20% denaturing PAGE. Gels are representative versions from a minimum of three independent experimental repeats.

**A**

| Compound | $R_1$ | $R_2$ | $R_3$ | SNM1B ($IC_{50}$/μM) | SNM1C ($IC_{50}$/μM) |
|---|---|---|---|---|---|
| 12 | | | Cl | 96 | 4.3 |
| 13 | | | Cl | 19 | 1.2 |
| 14 | | | Cl | 24 | 5.7 |
| 15 | | | Cl | 40 | 17 |
| 16 | | | Cl | 11 | 1.1 |
| 17 | | | Cl | 18 | 25 |
| 18 | | | Cl | >100 | 16 |
| 19 | | | Cl | 2.5 | 2.3 |
| 20 | | | H | 22 | 1.8 |
| 21 | | | Cl | 13 | 5.6 |
| 22 | | | Cl | 9.8 | 35 |
| 23 | | | F | 23 | 21 |
| 24 | | | OMe | >100 | 37 |

**B**

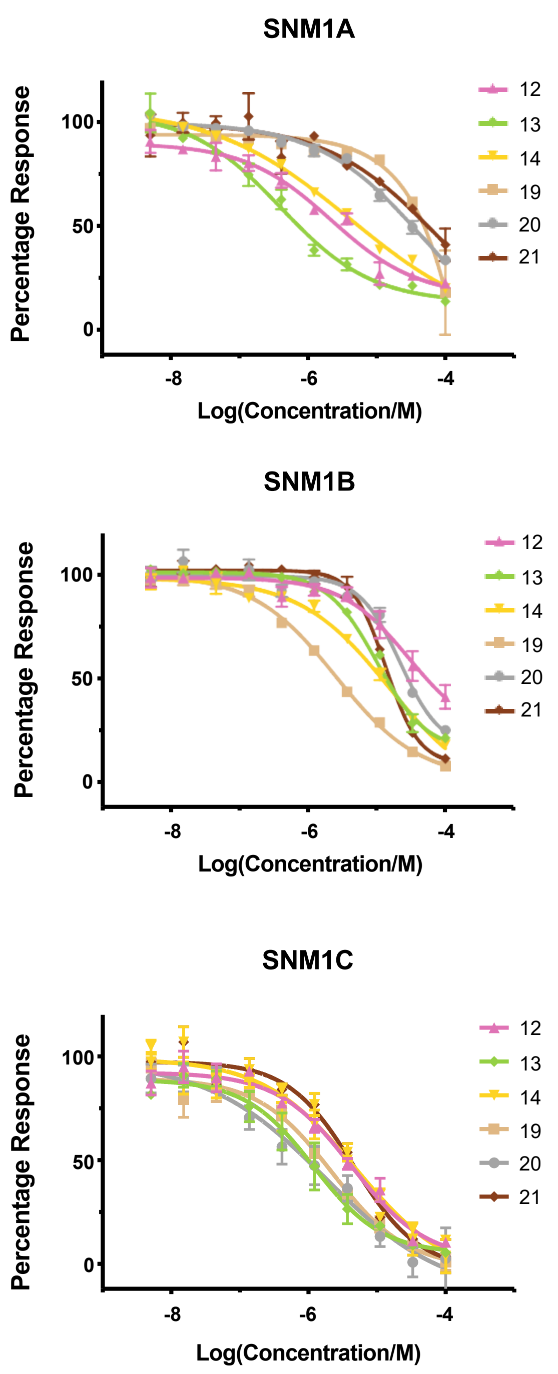


**C**

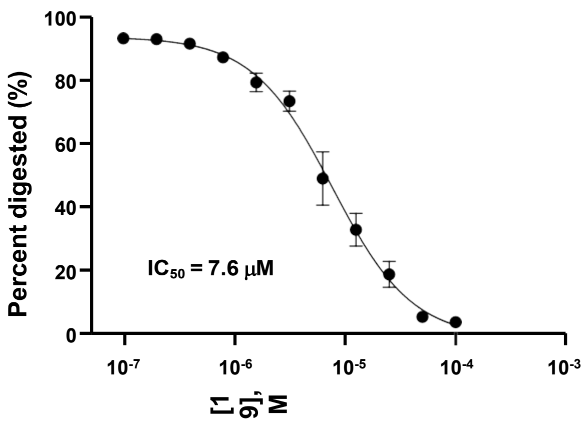


**Suppl. Figure 11. (A)** Table showing $IC_{50}$ values of compounds **12-24** tested against SNM1B and SNM1C. **(B)** Effects of compounds **12, 13, 14, 19, 20, 21** on SNM1A, SNM1B and SNM1C's nuclease activity measured *via* a real-time fluorescence-based nuclease assay, shown as mean ± SEM of four independent repeats. **(C)** $IC_{50}$ of compound **19** estimated from densitometric analysis of gel based assays. The data is derived from three independent experiments and error bars are SEM.

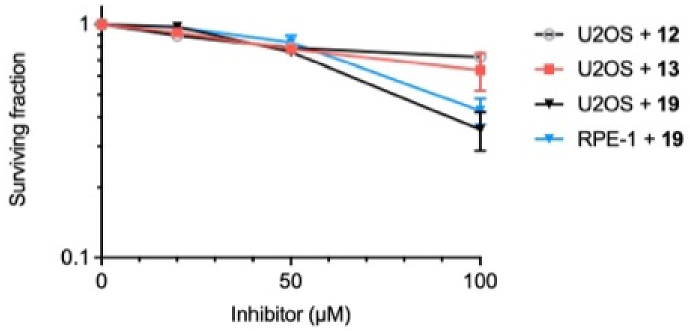


**Suppl. Figure 12.** Clonogenic survival assay to investigate optimal concentrations for cellular treatment with inhibitors 12, 13 and 19 in at the stated concentrations U2OS and 19 in RPE-1 cells. Data are the average of three repeats, error bars show the standard error of the mean.

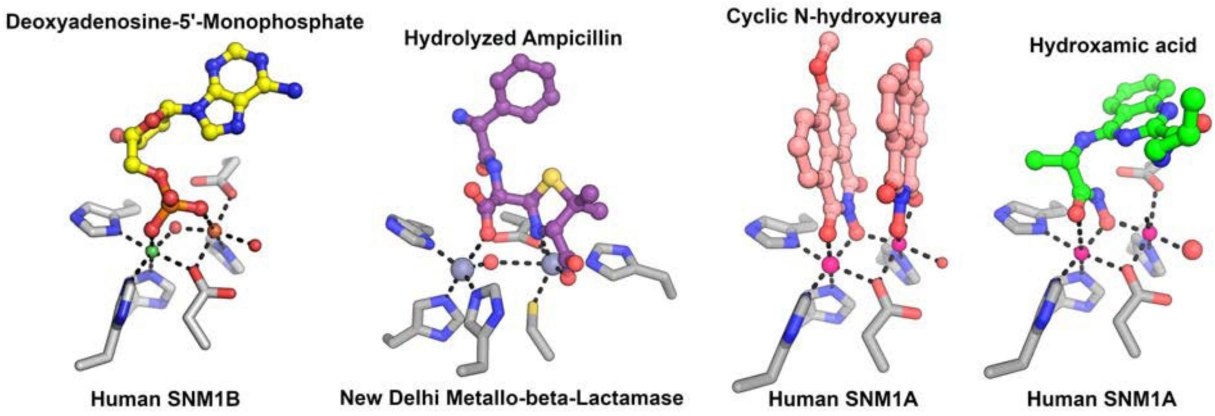


**Suppl. Figure 13.** Comparison of modes of interaction of cyclic-N-hydroxyurea (**1** pink, 8CEW) and hydroxamic acid (**20** green, PDB 8C8B) pharmacophores with a "true MBL" binding to a β-lactam antibiotic (purple, 4HL2) and a SNM1B nucleotide complex (yellow, PDB 7A1F). Structures of the small molecules are shown in the top panel

| Data collection and refinement statistics | | | | | | |
|---|---|---|---|---|---|---|
| | **SNM1A + Compd. 13** | **SNM1A + Compd. 19** | **SNM1A + Compd. 20** | **SNM1A + AZ1353160** | **SNM1A + Compd. 2** | **SNM1A + Compd. 1** |
| Space group | P212121 | P212121 | P212121 | P212121 | P212121 | P212121 |
| Cell dimensions, *a,b,c* (Å) | 51.68, 57.32, 113.73 | 51.58, 57.01, 113.42 | 51.69, 57.25, 113.52 | 51.54, 57.19, 113.95 | 51.31, 56.21, 112.95 | 51.59, 56.50, 113.95 |
| Angles α,β,γ (°) | 90, 90, 90 | 90, 90, 90 | 90, 90, 90 | 90, 90, 90 | 90, 90, 90 | 90, 90, 90 |
| Wavelength (Å) | 0.976 | 0.976 | 0.976 | 0.976 | 0.976 | 0.976 |
| Resolution (Å) | 40.3-1.80 (1.84-1.80) | 38.2-1.46 (1.50-1.46) | 36.3 – 1.46 (1.49-1.46) | 57.2 – 1.68 (1.72-1.68) | 56.48-1.76 (1.81-1.76) | 36.13-1.53 (1.57-1.53) |
| $R_{merge}$ | 0.143 (2.758) | 0.067 (4.14) | 0.066 (3.247) | 0.173 (8.04) | 0.113 (0.770) | 0.090 (0.985) |
| I/σI | 10.5 (1.1) | 15.2 (0.6) | 19.4 (0.7) | 13.6 (1.4) | 7.3 (1.9) | 9.8 (1.3) |
| CC1/2 | 0.998 (0.462) | 0.999 (0.32) | 0.999 (0.30) | 0.999 (0.708) | 0.994 (0.489) | 0.996 (0.609) |
| Completeness % | 100 (100) | 100 (99.9) | 97.8 (94.2) | 100 (100) | 99.9 (100) | 99.9 (99.7) |
| Multiplicity | 13.5 (12.9) | 13.4 (12.4) | 13.6 (13.0) | 25.4 (25.1) | 6.3 (5.8) | 6.0 (4.4) |
| No. Unique reflections | 32114 (1897) | 58935 (4271) | 57993 (2744) | 39243 (2843) | 33172 (2402) | 51062 (3716) |
| Resolution | 40.3 - 1.80 | 38.2 – 1.46 | 36.3 – 1.46 | 51.0 – 1.68 | 56.48 – 1.76 | 36.13 – 1.53 |
| $R_{work}/R_{free}$ (%) | 21.2/24.6 | 21.1/23.3 | 19.4/21.7 | 22.3/23.1 | 22.1/25.6 | 19.75/22.36 |
| No. atoms | | | | | | |
| Protein | 2701 | 2869 | 2730 | 2554 | 2726 | 2730 |
| Solvent | 101 | 112 | 251 | 142 | 249 | 255 |
| Ligand/ion | 26 | 54 | 27 | 53 | 22 | 42 |
| Average B factors ($Å^2$) | | | | | | |
| All atoms | 34 | 36 | 36 | 40 | 30 | 32 |
| Protein | 34 | 36 | 36 | 40 | 29 | 31 |
| Solvent | 32 | 34 | 41 | 41 | 35 | 39 |
| Ligand/ion | 41 | 54 | 41 | 44 | 39 | 35 |
| Wilson B | 28 | 26 | 25 | 27 | 25 | 23 |
| R.M.S. deviations | | | | | | |
| Bond lengths (Å) | 0.006 | 0.004 | 0.005 | 0.003 | 0.002 | 0.003 |
| Bond angles (°) | 0.826 | 0.701 | 0.740 | 0.639 | 0.543 | 0.629 |
| Ramachandran plot | | | | | | |
| Favoured (%) | 97.35 | 97.91 | 97.94 | 96 | 98 | 98 |
| Allowed (%) | 2.36 | 1.79 | 2.06 | 3 | 2 | 2 |
| PDB ID | 8C8S | 8C8D | 8C8B | 8GC9 | 8CF0 | 8CEW |

**Suppl. Table 1.** Data collection and refinement statistics.

| Name | Sequence | Use |
|---|---|---|
| **DNA1** | 5′ P-A[FITC]TA ATT TGA [BHQ]TCA TCT ATT AT- OH 3′ | Real-time fluorescence assays with SNM1A and SNM1B. |
| **DNA2** | 5′ OH-[FITC]TAA TTA ATA ATA GAT CAC CT[BHQ]-OH 3′ | Real-time fluorescence assays with SNM1C. |
| **DNA3** | 5′ P-ATA AAT ATT TTT TAT TAA TAA TAG ATC ACC TTT CTT TCT CTT CTC CCC TT - OH 3′ | Radioactive gel-based assays with SNM1A, SNM1B and SNM1C |

**Suppl. Table 2**. Details of oligonucleotides used in this study.

## Chemical Synthesis

### General Methods

Commercial reagents were from Sigma-Aldrich, Acros Organics, Fluka, Fluorochem, Abcr, and Fisher Scientific and were used without purification. Microwave reactions were performed using an Initiator 2 microwave reactor (Biotage). Flash column chromatography was performed using a Biotage Isolera automated flash column chromatography platform using Biotage Sfär or Biotage SNAP Ultra columns. Semi-preparative RP HPLC was performed using a Shimadzu LC-20AR machine equipped with an ACE 5 AQ (250 × 21.2mm) HPLC column utilising the following solvents: MilliQ $H_2O$ + 0.1% (v/v, solvent A) TFA, MeCN + 0.1% (v/v, solvent B) TFA.

IR spectra were recorded using a Bruker Tensor 27 FT-IR spectrometer either as a solid or a thin film. Selected characteristic peaks are reported in $cm^{-1}$. NMR were recorded using Bruker Avance spectrometers in the deuterated solvent stated. Assignments given correspond with the numbering systems drawn; where assignments are not provided, it was not possible to assign the resonances due to overlapping signals. Low-resolution mass spectra were recorded using an Agilent Technologies 1260 Infinity LC-MS system equipped with a 6120 Quadrupole mass spectrometer. High-resolution mass spectra (HRMS) were recorded in HPLC grade methanol using electrospray ionisation (ESI+/-) on a Bruker APEX III FT-ICR mass spectrometer. Optical rotations were measured using a Perkin-Elmer polarimeter at 25 °C and $[\alpha]^{25}$ values are given in $10^{-1}$ deg $cm^2$ $g^{-1}$.

### Procedures and Characterisation

### General Procedure A

To a solution of 2,4,6-trichloroquinazoline (31) (1 equiv.) and the requisite amino acid derivative (1.2-2 equiv.) in anhydrous DMF (5 mL) at 0 °C, anhydrous diisopropylamine (DIPEA) (2 equiv.) was added dropwise. The reaction mixture was stirred for 16 h at room temperature under an inert atmosphere, then diluted with cold $H_2O$ (75 mL). The resultant precipitate was collected by filtration and dried under a vacuum.

### General Procedure B

To a solution of the requisite 2-chloroquinazoline derivative in MeOH (4 mL), allylamine (1 mL) was added. The reaction mixture was stirred for 20 min at 120 °C in a microwave reactor, then concentrated *in vacuo*.

### General Procedure C

To a solution of the requisite ester (1 equiv.) in a mixture of THF/MeOH (1:1, 3 ml), 2.5 M $LiOH_{(aq)}$ (5 equiv.) was added. The reaction mixture was stirred for 10 min at 100 °C in a microwave reactor. The reaction was then neutralised using 1 M HCl $_{(aq)}$ (5 equiv.), then concentrated *in vacuo*.

### General Procedure D

To a solution of the requisite ester (1 equiv.) and $NH_2OH\cdot HCl$ (3 equiv.) in MeOH (5 mL), a solution of NaOMe 25 wt. % in MeOH (10 equiv.) was added dropwise. The reaction mixture was stirred for 2-5 h, with reaction progress being observed by TLC The reaction mixture was acidified to pH 2 with 4 M HCl in 1,4-dioxane and concentrated *in vacuo*.

**Scheme S-1. Synthesis of compound 1.**

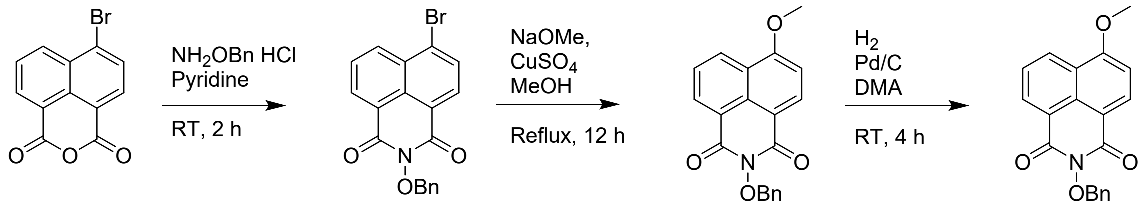


25 26 1

**2-Benzyloxy-6-bromo-benzo[de]isoquinoline-1,3-dione (25)**

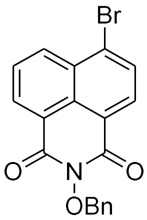


*O*-Benzylhydroxylamine hydrochloride (346 mg, 2.16 mmol) was added to a solution of 6- bromo-benzo[de]isochromene-1,3-dione (300 mg, 1.08 mmol) in anhydrous pyridine (10 ml). The reaction mixture was stirred under reflux for 2 h under an inert atmosphere. It was then cooled to room temperature, concentrated *in vacuo,* and EtOH (20 mL) was added to a solid residue. The precipitate was collected by filtration, washed with cold EtOH, then dried to give 2-benzyloxy-6-bromo-benzo[de]isoquinoline-1,3-dione (**25**) (395 mg, 1.03 mmol, 96%) as an orange solid.

IR $\nu_{max}/cm^{-1}$ (solid): 1719 and 1677 (C=O).

$^{1}H$ NMR (400 MHz, $CDCl_3$) δ 8.70 (dd, *J* = 7.3, 1.2 Hz, 1H), 8.62 (dd, *J* = 8.5, 1.2 Hz, 1H), 8.45 (d, *J* = 7.9 Hz, 1H), 8.07 (d, *J* = 7.9 Hz, 1H), 7.88 (dd, *J* = 8.5, 7.3 Hz, 1H), 7.72 – 7.63 (m, 2H), 7.45 – 7.33 (m, 3H), 5.27 (s, 2H).

$^{13}C$ NMR (101 MHz, $CDCl_3$) δ 160.6, 134.1, 134.1, 132.6, 131.7, 131.5, 131.2, 131.0, 130.1, 129.3, 128.7, 128.4, 123.4, 122.5, 78.8.

LRMS: *m/z*(%)= 382.2 (100), 384.2 (98) $[M + H]^+$; HRMS: calcd. $C_{19}H_{13}O_3N^{79}Br$ $[M + H]^+$: 382.0073; observed: 382.0070.

**2-Benzyloxy-6-methoxy-benzo[de]isoquinoline-1,3-dione (26)**

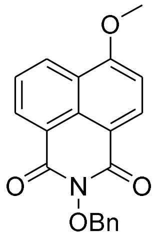


NaOMe 25 wt. % in MeOH (0.27 mL, 1.43mmol) was added to a solution of 2-benzyloxy-6- bromo-benzo[de]isoquinoline-1,3-dione **(**25) (360 mg, 0.94 mmol) and $CuSO_4$ (10 mg, 0.06 mmol) in anhydrous MeOH (8 mL). The reaction mixture was stirred under reflux for 12 h under an inert atmosphere, then cooled to room temperature. The precipitate was collected by filtration, washed with $H_2O$, then dried to give 2-benzyloxy-6-methoxy- benzo[de]isoquinoline-1,3-dione (**26**) (286 mg, 0.86 mmol, 90%) as a yellow solid.

IR $\nu_{max}/cm^{-1}$(solid): 1770 and 1743 (C=O).

$^{1}H$ NMR (400 MHz, $CDCl_3$) δ 8.63 (dd, *J* = 7.3, 1.2 Hz, 1H), 8.61 – 8.56 (m, 2H), 7.77 – 7.65 (m, 3H), 7.47 – 7.34 (m, 3H), 7.06 (d, *J* = 8.3 Hz, 1H), 5.26 (s, 2H), 4.13 (s, 3H).

$^{13}C$ NMR (101 MHz, $CDCl_3$) δ 161.5, 161.5, 161.0, 134.4, 134.1, 132.1, 130.1, 129.4, 129.2, 128.8, 128.6, 126.2, 123.9, 122.7, 115.2, 105.6, 78.6, 56.5.

LRMS: *m/z*(%)= 334.2 (100) [M + H]$^+$; HRMS: calcd. $C_{20}H_{16}O_4N$ [M + H]$^+$: 334.1074; observed: 334.1075.

**2-Hydroxy-6-methoxy-benzo[de]isoquinoline-1,3-dione (1)**

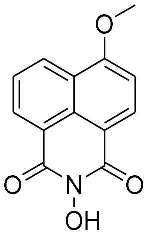

Palladium on carbon (35 mg, 10 wt. %) was added to a solution of 2-benzyloxy-6-methoxy-benzo[de]isoquinoline-1,3-dione (26) (275 mg, 0.83 mmol) in DMA (10 mL),. The reaction mixture was stirred for 4 h at room temperature under a hydrogen atmosphere. The reaction was filtered through Celite®, washed with DMA, then concentrated *in vacuo*. The solid residue was washed with MeOH, then dried to give 2-hydroxy-6-methoxy- benzo[de]isoquinoline-1,3-dione (**1**) (194 mg, 0.80 mmol, 96%) as a yellow solid.

IR $\nu_{max}$/cm$^{-1}$(solid): 1703 and 1650 (C=O).

$^{1}H$ NMR (400 MHz, DMSO-$d_6$) δ 10.63 (s, 1H), 8.50 (ddd, *J* = 8.5, 7.8, 1.2 Hz, 2H), 8.45 (d, *J* = 8.3 Hz, 1H), 7.80 (dd, *J* = 8.4, 7.3 Hz, 1H), 7.31 (d, *J* = 8.4 Hz, 1H), 4.12 (s, 3H).

$^{13}C$ NMR (101 MHz, DMSO-$d_6$) δ 160.9, 160.5, 160.5, 133.4, 131.1, 128.5, 127.4, 126.5, 122.9, 122.2, 114.4, 106.4, 56.7.

LRMS: *m/z*(%)= 244.1 (100) [M + H]$^+$; HRMS: calcd. $C_{13}H_9O_4N^{23}Na$ [M + Na]$^+$: 266.04238; observed: 266.04261.

**Scheme S-2. Synthesis of Compound 2.**

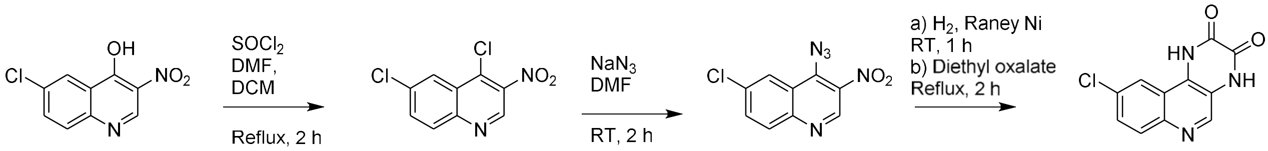


27 28 2

**4,6-Dichloro-3-nitroquinoline (27)**

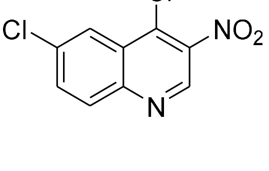

Anhydrous DMF (3 drops) and $SOCl_2$ (80 µL, 1.1 mmol) were added to a solution of 6-chloro- 3-nitroquinolin-4-ol (100 mg, 0.45 mmol) in anhydrous DCM (2 mL). The reaction mixture was stirred under

reflux for 2 h under an inert atmosphere. It was then cooled, quenched with sat. $NaHCO_{3(aq)}$ (10 mL) and extracted with DCM (2 × 10 mL). The combined organic layers were washed with brine (20 mL), dried over $MgSO_4$, then concentrated *in vacuo*. The product was purified using flash column chromatography (2-10% EtOAc in cyclohexane) to give 4,6-dichloro-3-nitroquinoline (**27**) (86mg, 0.35 mmol, 78%) as a yellow solid.

IR $\nu_{max}/cm^{-1}$(thin film): 1528 and 1334 (N-O).

$^1H$ NMR (400 MHz, $CDCl_3$) δ 9.23 (s, 1H), 8.40 (dd, *J* = 2.3, 0.5 Hz, 1H), 8.16 (d, *J* = 9.1 Hz, 1H), 7.88 (dd, *J* = 9.0, 2.3 Hz, 1H).

$^{13}C$ NMR (101 MHz, $CDCl_3$) δ 147.6, 144.6, 136.3, 135.4, 134.1, 131.9, 126.4, 124.8.

LRMS: *m/z*(%)= 243.0 (100), 245 (62) $[M + H]^+$.

**4-Azido-6-chloro-3-nitroquinoline (28)**

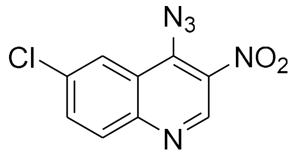


$NaN_3$ (46 mg, 1.1 mmol) was added to a solution of 4,6-dichloro-3-nitroquinoline (**27**) (86 mg, 0.35 mmol) in anhydrous DMF (2 mL). The reaction mixture was stirred for 2 h at room temperature and concentrated *in vacuo*. The solid residue was dissolved in DCM (10 mL), washed with $H_2O$ (10 mL) and brine (2 × 10 mL), and dried over $MgSO_4$. The solution was concentrated *in vacuo* to give 4-azido-6-chloro-3-nitroquinoline (**28**) (75 mg, 0.30 mmol, 86%) as a brown solid.

$^1H$ NMR (400 MHz, $CDCl_3$) δ 9.31 (s, 1H), 8.38 (dd, *J* = 2.3, 0.5 Hz, 1H), 8.12 – 7.97 (m, 1H), 7.82 (dd, *J* = 9.0, 2.3 Hz, 1H).

$^{13}C$ NMR (101 MHz, $CDCl_3$) δ 148.2, 146.0, 139.8, 135.1, 134.3, 131.4, 123.7, 122.8.

IR $\nu_{max}/cm^{-1}$(thin film): 2125 (N=N=N), 1566 (N-O) and 1360 (N-O). LRMS: *m/z*(%)=

250.0 (100), 252.0 (31) $[M + H]^+$.

**9-Chloro-1,4-dihydropyrazino[2,3-c]quinoline-2,3-dione (2)**

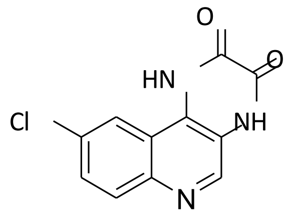


Raney nickel 50% slurry in $H_2O$ (10 mg) was added to a degassed solution of 4-azido-6-chloro-3-nitroquinoline (**28**) (40 mg, 0.16 mmol) in MeOH (5 mL). The reaction mixture was stirred at room temperature under a $H_2$ atmosphere for 1 h. The reaction mixture was filtered through Celite®, then concentrated *in vacuo* to afford 6-chloroquinoline-3,4-diamine as a brown oil, which was used directly for

next step without further purification. A mixture of 6-chloroquinoline-3,4-diamine in diethyl oxalate (1.5 mL) was stirred under reflux for 2 h. The reaction mixture was then cooled to 0 °C. The precipitate was collected by filtration, washed with MeOH and DCM, then dried under vacuum to give 9-chloro-1,4-dihydropyrazino[2,3-c]quinoline-2,3-dione (**2**) (14 mg, 0.06 mmol, 35% over 2 steps) as a brown solid.

IR $\nu_{max}/cm^{-1}$(solid): 1687 and 1659 (C=O).
$^{1}$H NMR (400 MHz, DMSO-$d_6$) δ 12.43 (s, 2H), 8.97 – 8.45 (m, 2H), 7.96 (d, *J* = 8.9 Hz, 1H), 7.66 (dd, *J* = 8.9, 2.3 Hz, 1H).

$^{13}$C NMR (101 MHz, DMSO-$d_6$) δ 157.0, 155.0, 142.5, 140.4, 131.6, 131.4, 128.4, 125.7, 120.5, 119.1, 117.9.

LRMS: *m/z*(%) 248.0 (100), 250.0 (32) [M + H]$^+$; HRMS: calcd. $C_{11}H_7O_2N_3{}^{35}Cl$ [M + H]$^+$: 248.02216; observed: 248.02213.

**Scheme S-3. Synthesis of 2-hydroxy-5-methoxy-benzo[de]isoquinoline-1,3-dione (3)**

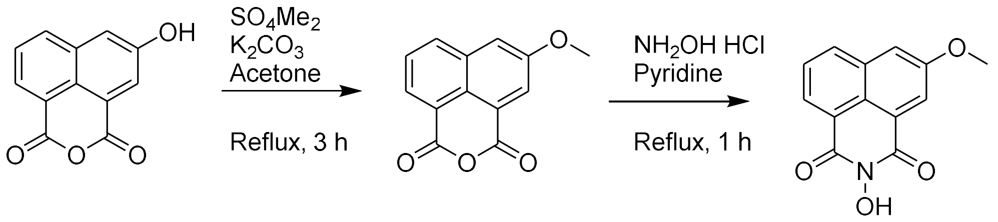


**29** **3**

**5-Methoxy-benzo[de]isochromene-1,3-dione (29)**

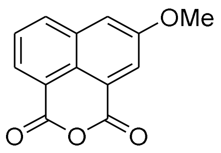


$SO_4Me_2$ (0.8 mL, 0.93 mmol) and $K_2CO_3$ (200 mg, 1.45 mmol) were added to a solution of 5- hydroxy-benzo[de]isochromene-1,3-dione (100 mg. 0.46 mmol) in anhydrous acetone (5 mL). The reaction mixture was stirred under reflux for 3 h under an inert atmosphere, then concentrated *in vacuo*. 1 M $HCl_{(aq)}$ (20 mL) was then added, the precipitate was filtered, washed with $H_2O$, then dried to give 5-methoxy-benzo[de]isochromene-1,3-dione **(29)** (78 mg, 0.34 mmol, 73%) as a cream solid.

IR $\nu_{max}/cm^{-1}$(solid): 1774 and 1739 (C=O).

$^{1}$H NMR (400 MHz, DMSO-$d_6$) δ 8.41 (dd, *J* = 8.3, 1.1 Hz, 1H), 8.34 (dd, *J* = 7.3, 1.1 Hz, 1H), 8.05 (d, *J* = 2.5 Hz, 1H), 8.01 (d, *J* = 2.6 Hz, 1H), 7.85 (dd, *J* = 8.3, 7.3 Hz, 1H), 4.00 (s, 3H).

$^{13}$C NMR (101 MHz, DMSO-$d_6$) δ 160.6, 160.3, 157.8, 134.0, 133.2, 129.7, 128.0, 125.1, 123.2, 120.5, 118.9, 114.3, 56.1.

LRMS: *m/z*(%)= 229.1 (100) [M + H]$^+$; HRMS: calcd. $C_{13}H_9O_4$ [M + H]$^+$: 229.0495; observed: 229.0498.

**2-Hydroxy-5-methoxy-benzo[de]isoquinoline-1,3-dione (3)**

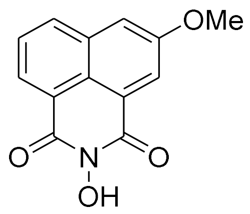


$NH_2OH{\cdot}HCl$ (45 mg, 0.66 mmol) was added to a solution of 5-methoxy- benzo[de]isochromene-1,3-dione (**29**) (78 mg, 0.34 mmol) in anhydrous pyridine (3 mL). The reaction mixture was stirred under reflux for 1 h under an inert atmosphere. The reaction mixture was then diluted with cold $H_2O$ (75 mL). The precipitate was collected by filtration, washed with $H_2O$, then dried to give 2-hydroxy-5-methoxy-benzo[de]isoquinoline-1,3-dione (**3**) (81 mg, 0.33, 97%) as a yellow solid.

Melting Point (from EtOH): 233-236°C. IR $\nu_{max}/cm^{-1}$(solid): 1682 and 1628 (C=O).
$^1H$ NMR (400 MHz, DMSO-$d_6$) δ 10.72 (s, 1H), 8.32 (s, 1H), 8.30 (s, 1H), 8.01 (d, *J* = 2.3 Hz, 1H), 7.89 (d, *J* = 2.6 Hz, 1H), 7.80 (t, *J* = 7.8 Hz, 1H), 3.97 (s, 3H).

$^{13}C$ NMR (101 MHz, DMSO-$d_6$) δ 161.4, 160.9, 158.2, 133.7, 133.7, 128.7, 128.2, 124.3, 122.6, 122.2, 122.1, 113.9, 56.5.

LRMS: *m/z*(%)= 244.1 (100) $[M + H]^+$; HRMS: calcd. $C_{13}H_{10}O_4N$ $[M + H]^+$: 244.0604; observed: 244.0606.

**Scheme S-4. synthesis of intermediate 31**

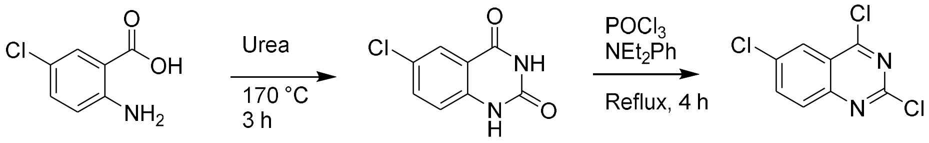


**30** **31**

**6-Chloroquinazoline-2,4-dione (30)**

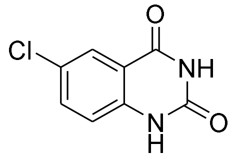


A mixture of 2-amino-5-chlorobenzoic acid (10 g, 58 mmol) and urea (35 g, 580 mmol) were stirred for 3 h at 170 °C. Then the reaction mixture was cooled to 100 °C and H2O (50 mL) was added. The mixture was stirred for a further 10 min and cooled to 0 °C. The precipitate was collected by filtration then washed with cold H2O. The precipitate was added to 0.5 M NaOH(aq) (100 mL) and stirred under reflux for 10 min. The mixture was then cooled at 0 °C for 1 h and the precipitate was collected by filtration. The precipitate was washed with cold H2O and dried under a vacuum. 6-Chloroquinazoline-2,4-dione (**30**) (8.9 g, 45 mmol, 77%) was obtained as a white solid.

$^1H$ NMR (400 MHz, DMSO-$d_6$) δ 11.45 (s, 1H), 11.28 (s, 1H), 7.82 (d, *J* = 2.5 Hz, 1H), 7.69 (dd, *J* = 8.7, 2.5 Hz, 1H), 7.19 (d, *J* = 8.8 Hz, 1H).

13C NMR (101 MHz, DMSO) δ 162.3, 150.5, 140.2, 135.3, 126.8, 126.4, 118.0, 116.3.

LRMS: *m/z*(%)= 197.0 (100), 199.0 (35) $[M + H]^+$.

**2,4,6-Trichloroquinazoline (31)**

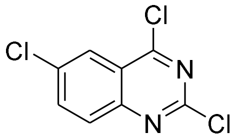

Diethylaniline (13.5 mL, 90 mmol) was added to a solution of 6-chloroquinazoline-2,4-dione

(**30**) (8.9 g, 45 mmol) in $POCl_3$ (26 mL, 270 mmol). The reaction mixture was stirred under reflux for 4 h. The mixture was then diluted with $Et_2O$ (200 mL), poured slowly to cold $H_2O$ (200 mL) and extracted with $Et_2O$ (3 × 200 mL). The combined organic layers were washed with brine (300 mL) and dried over $NaSO_4$. The product was filtered through a pad of silica in DCM, then concentrated *in vacuo* to give 2,4,6-trichloroquinazoline (**31**) (10.1 g, 43 mmol, 90%) as a yellow solid.

$^1$H NMR (400 MHz, $CDCl_3$) δ 8.24 (dd, *J* = 2.2, 0.7 Hz, 1H), 7.96 (dd, *J* = 9.0, 0.7 Hz, 1H), 7.92 (dd, *J* = 9.0, 2.1

Hz, 1H).13C NMR (101 MHz, $CDCl_3$) δ 163.0, 155.4, 150.8, 137.1, 135.3, 129.7, 124.9, 123.0.

6.1% wt. DCM as measured by NMR.

LRMS: *m/z*(%)= 233.0 (100), 235.0 (86), 237.0 (26) $[M + H]^+$.

**Scheme S-5. Synthesis of compounds 7-9**

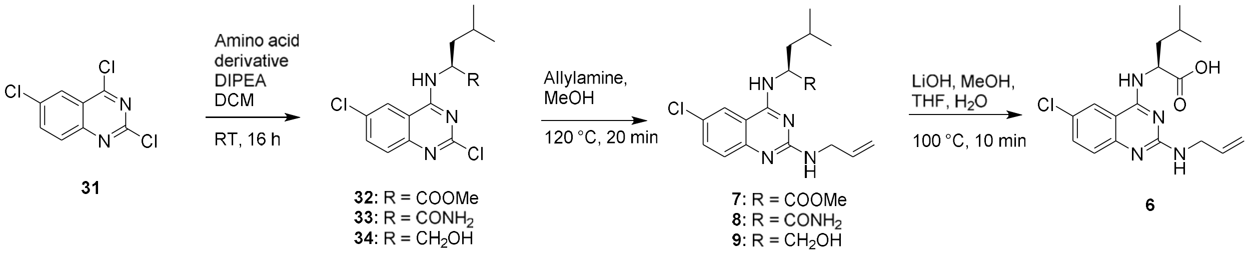


**Methyl (2,6-dichloroquinazolin-4-yl)-*L*-leucinate (32)**

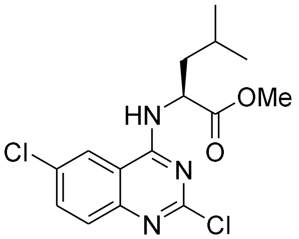

Methyl (2,6-dichloroquinazolin-4-yl)-*L*-leucinate (**32**) was prepared using General Procedure A, with 2,4,6-trichloroquinazoline (**31**) (300 mg, 1.28 mmol) and *L*-leucine methyl ester hydrochloride (320 mg, 1.76 mmol). Methyl (2,6-dichloroquinazolin-4-yl)-*L*-leucinate (**32**) (391 mg, 1.14 mmol, 89%) was obtained as a pale yellow solid.

IR $\nu_{max}$/cm$^{-1}$(thin film): 1724 (C=O).

$^1$H NMR (400 MHz, $CDCl_3$) δ 7.63 (d, *J* = 2.2 Hz, 1H,), 7.56 (dd, *J* = 8.9, 2.1 Hz, 1H), 7.51 (d, *J* = 8.9 Hz, 1H), 6.93 (d, *J* = 7.7 Hz, 1H), 5.04 (ddd, *J* = 9.9, 7.6, 3.9 Hz, 1H), 3.89 (s, 3H), 1.90 – 1.72 (m, 3H), 1.00 (dd, *J* = 7.6, 6.1 Hz, 6H).

13C NMR (101 MHz, $CDCl_3$) δ 175.4, 159.9, 157.5, 149.1, 134.3, 131.8, 129.2, 120.6, 113.6, 53.0, 52.9, 41.2, 25.2, 23.1, 22.0.

LRMS: *m/z*(%)= 342.1 (100), 344.1 (64) [M + H]$^+$; HRMS: calcd. $C_{15}H_{18}O_2N_3{}^{35}Cl$ [M + H]$^+$: 342.0771; observed: 342.0770.

**Methyl (2-allylamino-6-chloroquinazolin-4-yl)-*L*-leucinate (7)**

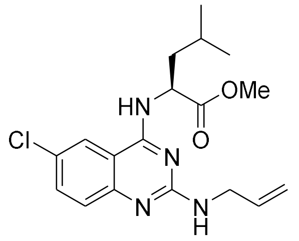

Methyl (2-allylamino-6-chloroquinazolin-4-yl)-*L*-leucinate (**7**) was prepared using General Procedure B, with methyl (2,6-dichloroquinazolin-4-yl)-*L*-leucinate **(32)** (290 mg, 0.87 mmol). The product was purified using flash column chromatography (10−50% EtOAc in cyclohexane) to give methyl (2-allylamino-6-chloroquinazolin-4-yl)-*L*-leucinate (**7**) (146 mg, 0.40 mmol, 47%) as a yellow solid.

IR $\nu_{max}/cm^{-1}$(thin film): 1726 (C=O).

$^1H$ NMR (400 MHz, $CDCl_3$) δ 7.50 (d, *J* = 2.3 Hz, 1H), 7.41 (dd, *J* = 8.9, 2.2 Hz, 1H), 7.30 (d, *J* = 8.9 Hz, 1H), 6.07 (s, 1H), 5.97 (ddt, *J* = 17.2, 10.6, 5.5 Hz, 1H), 5.25 (dd, *J* = 17.2, 1.7 Hz, 1H), 5.12 (dd, *J* = 10.2, 1.5 Hz, 1H), 4.97 – 4.87 (m, 1H), 4.10 (app. ddt, *J* = 5.7, 4.1, 1.6 Hz, 2H), 3.78 (s, 3H), 1.98 – 1.71 (m, 3H), 1.00 (d, *J* = 6.1 Hz, 3H), 0.97 (d, *J* = 5.9 Hz, 3H).

$^{13}C$ NMR (101 MHz, $CDCl_3$) δ 174.4, 159.0, 158.8, 150.5, 135.5, 133.1, 126.9, 125.7, 120.3, 115.3, 111.1, 77.2, 76.8, 76.5, 52.2, 52.1, 43.8, 41.2, 24.8, 22.7, 21.9.

LRMS: *m/z*(%)= 363.2 (100), 365.2 (36) [M + H]$^+$; HRMS: calcd. $C_{18}H_{24}O_2N_4{}^{35}Cl$ [M + H]$^+$: 363.15823; observed 363.15891.

$[a]\frac{25}{D}$ = −8.1 (c = 1 in $CHCl_3$).

**(2-Allylamino-6-chloroquinazolin-4-yl)-*L*-leucine (6)**

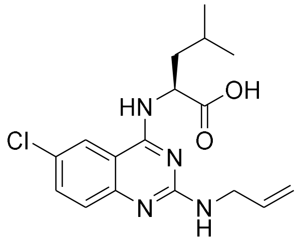

(2-Allylamino-6-chloroquinazolin-4-yl)-*L*-leucine (**6**) was prepared using General Procedure C, with methyl (2-allylamino-6-chloroquinazolin-4-yl)-*L*-leucinate (**7**) (60 mg, 0.17 mmol). Purification using flash column chromatography (0−10% MeOH with 1% (v/v) formic acid in EtOAc) gave (2-allylamino-6-chloroquinazolin-4-yl)-*L*-leucine (**6**) (34 mg, 0.10 mmol, 59%) as a white solid.

IR $\nu_{max}/cm^{-1}$(solid): 3038 (O-H) and 1666 (C=O).

$^1H$ NMR (500 MHz, DMSO-$d_6$ + Hydrochloric acid-*d* solution) δ 8.76 (d, *J* = 2.2 Hz, 1H), 7.80 (dd, *J* = 8.9, 2.3 Hz, 1H), 7.49 (d, *J* = 8.9 Hz, 1H), 5.80 (ddt, *J* = 15.9, 10.3, 5.3 Hz, 1H) 5.18 (d, *J* = 17.2 Hz, 1H), 5.06 (d, *J* = 10.4 Hz, 1H), 4.64 (dd, *J* = 10.9, 4.1 Hz, 1H), 3.96 (d, *J* = 5.4 Hz, 2H), 1.99 – 1.92 (m, 1H), 1.72 – 1.53 (m, 2H), 0.86 (d, *J* = 6.2 Hz, 3H), 0.81 (d, *J* = 6.1 Hz, 3H).

$^{13}C$ NMR (126 MHz, DMSO-$d_6$ + hydrochloric acid-*d* solution) δ 173.0, 163.4, 159.7, 152.7, 138.1, 136.0, 134.2, 128.8, 124.6, 119.1, 116.9, 110.7, 53.7, 43.3, 24.9, 23.2, 21.5.

LRMS: *m/z*(%)= 349.2 (100), 351.2 (33) [M + H]$^+$; HRMS: calcd. $C_{17}H_{22}O_2N_4{}^{35}Cl$ [M + H]$^+$: 349.14267; observed: 349.14258.

[a]$_D^{25}$ = −7.3 (c = 0.3 in MeOH:1M $HCl_{(aq)}$ (9:1)).

**(*S*)-2-(2,6-Dichloroquinazolin-4-yl)amino-4-methylpentanamide (33)**

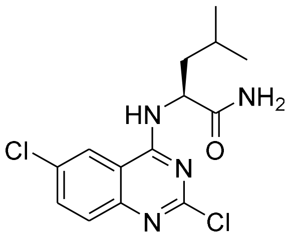

(*S*)-2-(2,6-Dichloroquinazolin-4-yl)amino-4-methylpentanamide (**33**) was prepared using General Procedure A, with 2,4,6-trichloroquinazoline (**31**) (300 mg, 1.28 mmol) and *L*- leucine amide hydrochloride (320 mg, 2.46 mmol). (*S*)-2-(2,6-Dichloroquinazolin-4-yl)amino- 4-methylpentanamide (**33**) (394 mg, 1.21 mmol, 94%) was obtained as a pale yellow solid.

IR $\nu_{max}$/cm$^{-1}$(solid): 1688 (C=O).

$^1H$ NMR (400 MHz, DMSO-$d_6$) δ 8.68 (d, *J* = 7.9 Hz, 1H), 8.65 (d, *J* = 2.3 Hz, 1H), 7.83 (dd, *J* = 8.9, 2.3 Hz, 1H), 7.64 (d, *J* = 8.9 Hz, 1H), 7.61 (s, 1H), 7.08 (s, 1H), 4.71 (ddd, *J* = 11.4, 7.8, 3.8 Hz, 1H), 1.84 (ddd, *J* = 12.7, 11.2, 3.8 Hz, 1H), 1.78 – 1.52 (m, 1H), 0.92 (d, *J* = 6.3 Hz, 3H), 0.87 (d, *J* = 6.3 Hz, 3H).

$^{13}C$ NMR (101 MHz, DMSO-$d_6$) δ 173.6, 160.5, 157.0, 149.0, 134.0, 130.1, 128.6, 123.2, 114.6, 53.1, 40.2, 40.1, 40.0, 39.9, 39.7, 39.5, 39.3, 39.1, 38.9, 24.5, 23.2, 21.1.

LRMS: *m/z*(%)= 327.1 (100), 329.1 (66) [M + H]$^+$; HRMS: calcd. $C_{14}H_{17}ON$ $^{35}Cl_2$ [M$_4$+ H]$^+$: 327.0774; observed: 327.0774.

**(*S*)-2-(2-Allylamino-6-chloroquinazolin-4-yl)amino-4-methylpentanamide (8)**

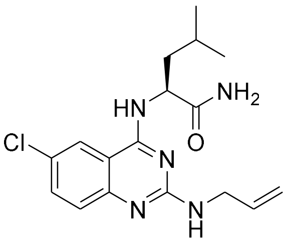

(*S*)-2-(2-Allylamino-6-chloroquinazolin-4-yl)amino-4-methylpentanamide (**8**) was prepared using General Procedure B, with (*S*)-2-(2,6-Dichloroquinazolin-4-yl)amino-4- methylpentanamide (**33**) (370

mg, 1.13 mmol). Purification using flash column chromatography (0–10% MeOH in DCM) gave (*S*)-2-(2-allylamino-6-chloroquinazolin-4- yl)amino-4-methylpentanamide (**8**) (97 mg, 0.28 mmol, 25%) as a white solid.

IR $\nu_{max}$/cm$^{-1}$(solid): 1688 (C=O).

$^{1}$H NMR (400 MHz, DMSO-$d_6$) δ 8.30 (d, *J* = 2.4 Hz, 1H), 7.81 (d, *J* = 7.8 Hz, 1H), 7.47 (dd, *J* = 8.9, 2.4 Hz, 1H), 7.32 (s, 1H), 7.22 (d, *J* = 8.9 Hz, 1H), 6.95 (s, 1H), 6.75 (s, 1H), 5.90 (ddd, *J* = 15.9, 10.4, 5.1 Hz, 1H), 5.16 (dd, *J* = 17.1, 1.8 Hz, 1H), 5.01 (dd, *J* = 10.2, 1.7 Hz, 1H), 4.68 (ddt, *J* = 10.6, 7.7, 4.3 Hz, 1H), 3.94 (dt, *J* = 5.8, 1.6 Hz, 1H), 1.89 – 1.54 (m, 2H), 0.92 (d, *J* = 6.3 Hz, 3H), 0.86 (d, *J* = 6.3 Hz, 3H).

$^{13}$C NMR (101 MHz, DMSO-$d_6$) δ 174.6, 159.3, 136.7, 132.4, 122.7, 114.7, 52.6, 43.1, 40.2, 40.1, 40.0, 39.9, 39.7, 39.5, 39.3, 39.1, 38.9, 24.5, 23.2, 21.4.

LRMS: *m/z*(%)= 348.2 (100), 348.2 (34) [M + H]$^+$; HRMS: calcd. $C_{18}H_{25}ON_5{}^{35}Cl$ [M + H]$^+$: 348.15875; observed: 348.15856.

$[a]\frac{25}{D}$ = +14.5 (c = 1 in MeOH:DMF (9:1)).

**(*S*)-2-(2,6-Dichloroquinazolin-4-yl)amino-4-methylpentan-1-ol (34)**

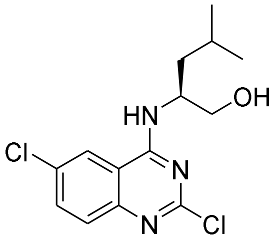


(*S*)-2-(2,6-Dichloroquinazolin-4-yl)amino-4-methylpentan-1-ol (**34**) was prepared using General Procedure A, with 2,4,6-trichloroquinazoline (**31**) (300 mg, 1.28 mmol) and *L*- Leucinol (270 µL, 2.12 mmol) to give (*S*)-2-(2,6-dichloroquinazolin-4-yl)amino-4- methylpentan-1-ol (**34**) (328 mg, 1.05 mmol, 80%) as a pale yellow solid.

IR $\nu_{max}$/cm$^{-1}$(solid): 1733 and 1664 (C=O).

$^{1}$H NMR (400 MHz, DMSO-$d_6$) δ 8.55 (d, *J* = 2.3 Hz, 1H), 8.34 (d, *J* = 8.4 Hz, 1H), 7.80 (dd, *J* = 8.9, 2.3 Hz, 1H), 7.62 (d, *J* = 8.9 Hz, 1H), 4.83 (t, *J* = 5.8 Hz, 1H), 4.45 (ddd, *J* = 9.8, 8.2, 4.2 Hz, 0H), 3.48 (td, *J* = 5.8, 3.4 Hz, 2H), 1.76 – 1.51 (m, 2H), 1.43 (tt, *J* = 9.3, 4.6 Hz, 1H), 0.90 (d, *J* = 3.6 Hz, 3H), 0.88 (d, *J* = 3.7 Hz, 3H).

$^{13}$C NMR (101 MHz, DMSO-$d_6$) δ 160.5, 157.4, 149.1, 133.8, 130.0, 128.7, 122.8, 114.5, 63.2, 51.3, 40.1, 40.0, 39.9, 39.8, 39.7, 39.6, 39.5, 39.3, 39.1, 38.9, 24.4, 23.4, 21.9.

LRMS: *m/z*(%)= 314.1 (100), 316.1 (69) [M + H]$^+$; HRMS: calcd. $C_{14}H_{18}ON_3{}^{35}Cl_2$ [M + H]$^+$: 314.0821; observed: 314.0821.

### (*S*)-2-(2-Allylamino-6-chloroquinazolin-4-yl)amino-4-methylpentan-1-ol (9)

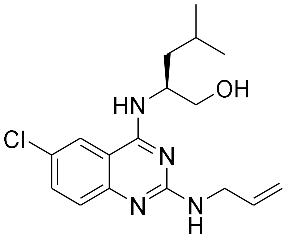

(*S*)-2-(2-Allylamino-6-chloroquinazolin-4-yl)amino-4-methylpentan-1-ol (**9**) was prepared using General Procedure B, with (*S*)-2-(2,6-dichloroquinazolin-4-yl)amino-4-methylpentan-1- ol (**34**) (270 mg, 0.85 mmol). Purification using flash column chromatography (0–10% MeOH with 1% (v/v) formic acid in EtOAc) gave (*S*)-2-(2-allylamino-6-chloroquinazolin-4-yl)amino- 4-methylpentan-1-ol (**9**) (122 mg, 0.36 mmol, 42%) as a white solid**.**

IR $\nu_{max}$/cm$^{-1}$(solid): 3650 (O-H).

$^1$H NMR (500 MHz, DMSO-$d_6$ + formic acid-*d* ) δ 8.30 (s, 1H), 7.66 – 7.48 (m, 2H), 7.27 (d, *J* = 8.9 Hz, 1H), 5.91 (ddt, *J* = 15.9, 10.2, 5.2 Hz, 1H), 5.16 (dd, *J* = 17.2, 1.9 Hz, 1H), 5.04 (dd, *J* = 10.3, 1.8 Hz, 1H), 4.46 (dd, *J* = 9.2, 4.7 Hz, 1H), 3.96 (q, *J* = 5.9 Hz, 2H), 3.47 (d, *J* = 5.8 Hz, 1H), 1.61 (dddd, *J* = 25.6, 18.3, 8.3, 5.0 Hz, 2H), 1.42 (ddd, *J* = 17.4, 8.5, 4.1 Hz, 1H), 0.90 (d, *J* = 6.4 Hz, 3H), 0.87 (d, *J* = 6.5 Hz, 3H).

$^{13}$C NMR (126 MHz, DMSO-$d_6$ + formic acid-*d*) δ 159.7, 136.6, 133.4, 125.0, 123.2, 115.4, 112.1, 63.8, 55.4, 51.1, 49.1, 43.5, 40.5, 40.3, 40.2, 40.1, 40.0, 39.8, 39.6, 39.5, 26.8, 24.9, 23.8, 22.5.

LRMS: *m/z*(%)= 335.2 (100), 337.2 (34) [M + H]$^+$; HRMS: calcd. $C_{17}H_{24}ON$ $^{35}$Cl [M + H]$^+$: 335.16296 ; observed: 335.16332.

[a]$_D^{25}$ = −23.4 (c = 1 in MeOH:DMF (9:1)).

### Scheme S-6. Synthesis of compounds 10-14

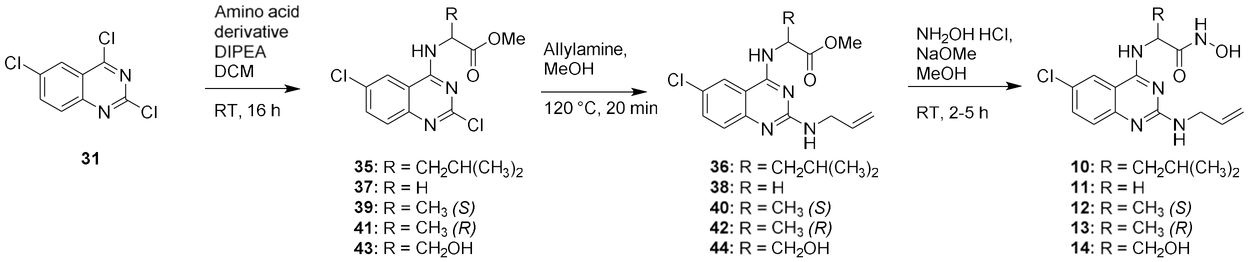


### Methyl (2,6-dichloroquinazolin-4-yl)leucinate (35)

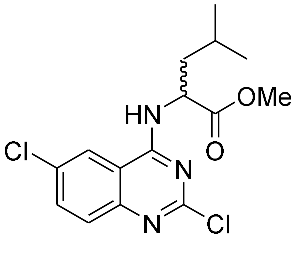

Methyl (2,6-dichloroquinazolin-4-yl)leucinate (**35**) was prepared using General Procedure A, with 2,4,6-trichloroquinazoline (**31**) (500 mg, 1.28 mmol) and *DL*-leucine methyl ester hydrochloride (584 mg, 1.92

mmol). Methyl (2,6-dichloroquinazolin-4-yl)-*L*-leucinate (**35**) (593 mg, 1.12 mmol, 82%) was obtained as a pale yellow solid.

$^{1}$H NMR (400 MHz, $CDCl_3$) δ 7.61 (d, *J* = 2.1 Hz, 1H), 7.53 (dt, *J* = 8.9, 1.9 Hz, 1H), 7.48 (dd, *J* = 9.0, 2.3 Hz, 1H), 7.15 – 7.05 (m, 1H), 5.17 – 4.88 (m, 1H), 3.90 (s, 3H), 1.86 – 1.73 (m, 3H), 1.01 (d, *J* = 6.2 Hz, 2H), 0.98 (d, *J* = 6.0 Hz, 1H).

$^{13}$C NMR (101 MHz, $CDCl_3$) δ 176.1, 160.0, 157.4, 148.8, 134.1, 131.7, 128.9, 120.6, 113.4, 77.5, 77.2, 76.8, 53.1, 40.9, 25.2, 23.1, 21.8.

LRMS: *m/z*(%)= 342.1 (100), 344.1 (63) $[M + H]^+$.

**Methyl (2-allylamino-6-chloroquinazolin-4-yl)leucinate (36)**

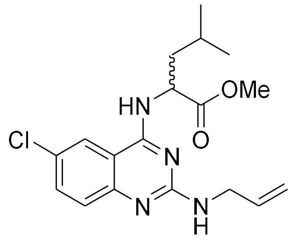


Methyl (2-allylamino-6-chloroquinazolin-4-yl)leucinate (**36**) was prepared using General Procedure B, with methyl (2,6-dichloroquinazolin-4-yl)leucinate (**35**) (300 mg, 0.90 mmol). Purification using flash column chromatography (10−50% EtOAc in cyclohexane) gave methyl (2-allylamino-6-chloroquinazolin-4-yl)leucinate (**36**) (166 mg, 0.47 mmol 52 %) as a pale yellow solid.

$^{1}$H NMR (400 MHz, $CDCl_3$) δ 7.48 (dd, *J* = 2.4, 1.3 Hz, 1H), 7.40 – 7.34 (m, 1H), 7.29 – 7.21 (m, 1H), 6.34 (s, 1H), 5.96 (ddt, *J* = 17.2, 10.6, 5.5 Hz, 1H), 5.25 (dd, *J* = 17.2, 1.7 Hz, 1H), 5.15 – 5.06 (m, 1H), 4.90 (q, *J* = 7.4 Hz, 1H), 4.09 (tt, *J* = 5.7, 1.6 Hz, 3H), 3.80 (s, 3H), 1.83 – 1.70 (m, 3H), 1.00 (d, *J* = 6.1 Hz, 3H), 0.96 (d, *J* = 6.0 Hz, 3H).

$^{13}$C NMR (101 MHz, $CDCl_3$) δ 175.2, 159.2, 159.0, 150.3, 135.8, 133.3, 126.8, 126.1, 120.7, 115.7, 111.3, 77.5, 77.2, 76.8, 52.6, 44.1, 41.3, 25.1, 23.0, 22.1.

LRMS: *m/z*(%)= 363.2 (100), 365.2 (36) $[M + H]^+$.

**2-(2-allylamino-6-chloroquinazolin-4-yl)amino-*N*-hydroxy-4-methylpentanamide (10)**

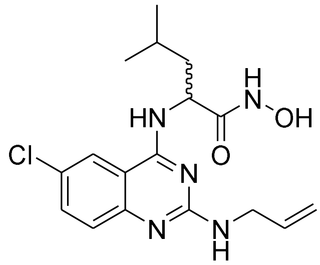


2-(2-allylamino-6-chloroquinazolin-4-yl)amino-*N*-hydroxy-4-methylpentanamide (**10**) was prepared using General Procedure D, with methyl (2-allylamino-6-chloroquinazolin-4- yl)leucinate (**36**) (160 mg, 0.44 mmol). Purification using flash column chromatography (2−10% MeOH in DCM) gave 2-(2-allylamino-6-chloroquinazolin-4-yl)amino-*N*-hydroxy-4- methylpentanamide (**10**) (62 mg, 0.17 mmol, 39%) as a white solid.

IR $v_{max}/cm^{-1}$(solid): 3303 (O-H) and 1629 (C=O).$^{1}$H NMR (500 MHz, DMSO-$d_6$) δ 10.58 (s, 1H), 8.84 (s, 1H), 8.35

(d, $J$ = 2.5 Hz, 1H), 7.95 (s,
1H), 7.50 (dd, $J$ = 8.9, 2.4 Hz, 1H), 7.26 (d, $J$ = 8.9 Hz, 1H), 6.80 (s, 1H), 5.95 (d, $J$ = 15.5 Hz, 1H), 5.20 (dd, $J$ = 17.2, 1.9 Hz, 1H), 5.05 (d, $J$ = 10.2 Hz, 1H), 4.68 (s, 1H), 4.07 – 3.87 (m, 2H), 1.80 (d, $J$ = 8.7 Hz, 1H), 1.72 – 1.55 (m, 2H), 0.93 (d, $J$ = 6.4 Hz, 3H), 0.86 (d, $J$ = 6.3 Hz, 3H)

$^{13}$C NMR (126 MHz, DMSO-$d_6$) δ 169.1, 159.1, 158.9, 150.1, 136.4, 135.2, 132.6, 126.5, 123.8, 122.9, 114.9, 111.9, 50.7, 43.1, 40.0, 39.9, 39.7, 39.5, 39.4, 39.2, 39.0, 24.4, 22.9, 21.6.

LRMS: $m/z$(%)= 364.2 (100), 366.2 (35) [M + H]$^+$; HRMS: calcd. $C_{17}H_{23}O_2N_5{}^{35}Cl$ [M + H]$^+$: 364.1535 ; observed: 364.1537.

IR $\nu_{max}$/cm$^{-1}$(solid): 3303 (O-H) and 1629 (C=O).

**Methyl (2,6-dichloroquinazolin-4-yl-glycinate (37)**

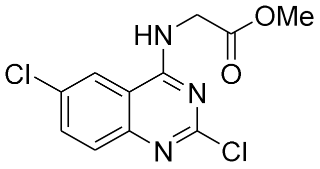

Methyl (2,6-dichloroquinazolin-4-yl-glycinate (**37**) was prepared using General Procedure A, with 2,4,6-trichloroquinazoline (**31**) (500 mg, 2.1 mmol) and glycine methyl ester hydrochloride (400 mg, 3.2 mmol). Methyl (2,6-dichloroquinazolin-4-yl)-glycinate (**37**) (589 mg, 2.0 mmol , 96%) was obtained as a pale pink solid.

IR $\nu_{max}$/cm$^{-1}$(thin film): 1730 (C=O).

$^1$H NMR (400 MHz, DMSO-$d_6$) δ 9.33 (t, $J$ = 5.8 Hz, 1H), 8.45 (d, $J$ = 2.3 Hz, 1H), 7.87 (dd, $J$ = 8.9, 2.3 Hz, 1H), 7.69 (d, $J$ = 8.9 Hz, 1H), 4.28 (d, $J$ = 5.8 Hz, 2H), 3.68 (s, 3H).

$^{13}$C NMR (101 MHz, DMSO-$d_6$) δ 170.2, 161.0, 157.3, 149.4, 134.8, 131.0, 129.4, 123.0, 114.8, 52.5, 42.9.

LRMS: $m/z$(%)= 286.1 (100), 288.1 (63) [M + H]$^+$; HRMS: calcd. $C_{11}H_{10}O_2N_3{}^{35}Cl_2$ [M + H]$^+$: 286.0145; observed: 286.0146.

IR $\nu_{max}$/cm$^{-1}$(thin film): 1730 (C=O).

**Methyl (2-allylamino-6-chloroquinazolin-4-yl)glycinate (38)**

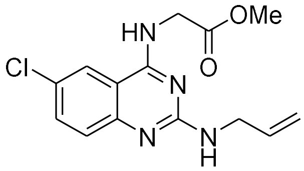

Methyl (2-allylamino-6-chloroquinazolin-4-yl)-glycinate (**38**) was prepare using General Procedure B, with methyl (2,6-dichloroquinazolin-4-yl-glycinate (**37**) (300 mg, 1.05 mmol). The product was purified using flash column chromatography (40−80% EtOAc in cyclohexane) to give methyl (2-allylamino-6-chloroquinazolin-4-yl)glycinate (**38**) (93 mg, 0.30 mmol, 29%) as a white solid.

IR $\nu_{max}$/cm$^{-1}$(thin film): 1726 (C=O).

$^1$H NMR (400 MHz, $CDCl_3$) δ 7.56 (d, *J* = 0.9 Hz, 0H), 7.54 (d, *J* = 2.3 Hz, 1H), 7.45 (dd, *J* = 8.9, 2.3 Hz, 1H), 7.35 (d, *J* = 8.9 Hz, 1H), 6.19 (s, 1H), 5.97 (ddt, *J* = 17.2, 10.6, 5.5 Hz, 1H), 5.26 (dd, *J* = 17.2, 1.6 Hz, 1H), 5.12 (dd, *J* = 10.2, 1.5 Hz, 1H), 5.08 (s, 1H), 4.32 (d, *J* = 4.9 Hz, 2H), 4.11 (ddd, *J* = 5.7, 4.1, 1.6 Hz, 2H), 3.83 (s, 3H).

$^{13}$C NMR (101 MHz, $CDCl_3$) δ 171.2, 159.1, 135.7, 133.5, 129.2, 126.3, 120.7, 117.4, 115.8, 77.5, 77.2, 76.8, 52.7, 44.1, 43.0.

LRMS: *m/z*(%)= 307.1 (100), 309.1 (34) [M + H]$^+$; HRMS: calcd. $C_{14}H_{16}O_2N_4{}^{35}Cl$ [M + H]$^+$: 307.0956 ; observed: 307.0958.

**2-(2-Allylamino-6-chloroquinazolin-4-yl)amino-*N*-hydroxyethanamide (11)**

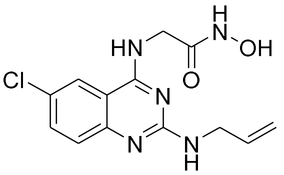


2-(2-Allylamino-6-chloroquinazolin-4-yl)amino-*N*-hydroxyethanamide (**11**) was prepared using General Procedure D, with methyl (2-allylamino-6-chloroquinazolin-4-yl)glycinate (**38**) (68 mg, 0.22 mmol). The product was purified using flash column chromatography (3−15% MeOH + 0.5% (v/v) AcOH in DCM) to give 2-(2-Allylamino-6-chloroquinazolin-4-yl)amino-*N*- hydroxyethanamide (**11**) (24 mg, 0.08 mmol 35%) as a white solid. ~4% wt. acetic acid impurity was present as judged by $^1$H NMR, likely due to salt formation.

IR $\nu_{max}$/cm$^{-1}$(solid): 3330 (O-H) and 1640 (C=O).

$^1$H NMR (500 MHz, DMSO-$d_6$) δ 10.63 (s, 1H), 9.02 (s, 1H), 8.44 (d, *J* = 30.7 Hz, 1H), 8.20 (s, 1H), 7.57 (s, 1H), 7.33 (d, *J* = 8.9 Hz, 1H), 6.95 (d, *J* = 9.1 Hz, 1H), 5.91 (ddt, *J* = 16.0, 11.0, 5.4 Hz, 1H), 5.33 – 5.13 (m, 1H), 5.05 (dq, *J* = 10.4, 1.7 Hz, 1H), 4.02 (d, *J* = 5.2 Hz, 2H), 3.99 – 3.93 (m, 2H).

$^{13}$C NMR (126 MHz, DMSO-$d_6$) δ 165.7, 159.4, 135.9, 133.4, 126.1, 124.9, 122.9, 115.2, 112.2, 43.1, 42.0, 40.0, 39.9, 39.7, 39.5, 39.4, 39.2, 39.0.

LRMS: *m/z*(%)= 308.1 (100), 310.1 (36) [M + H]$^+$; HRMS: calcd. $C_{13}H_{15}O_2N_5{}^{35}Cl$ [M + H]$^+$: 308.0909 ; observed: 308.0909.

**Methyl (2,6-dichloroquinazolin-4-yl)-D-alaninate (39)**

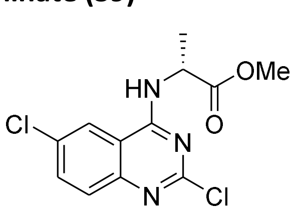


Methyl (2,6-dichloroquinazolin-4-yl)-*D*-alaninate (**39**) was prepared using General Procedure A, with 2,4,6-trichloroquinazoline (**31**) (800 mg, 3.4 mmol) and *D*-alanine methyl ester hydrochloride (575 mg, 4.1 mmol). Methyl (2,6-dichloroquinazolin-4-yl)-*D*-alaninate (**39**) (624 mg, 2.1 mmol, 61%) was obtained as a yellow solid.

IR $\nu_{max}$/cm$^{-1}$(thin film): 1718 (C=O).

$^1$H NMR (400 MHz, $CDCl_3$) δ 7.69 (dd, *J* = 1.9, 0.9 Hz, 1H), 7.63 – 7.58 (m, 2H), 6.87 (d, *J* = 7.0 Hz, 1H), 5.20 – 4.80 (m, 1H), 3.88 (s, 3H), 1.61 (d, *J* = 7.2 Hz, 3H).

$^{13}$C NMR (101 MHz, $CDCl_3$) δ 174.5, 159.3, 157.6, 149.3, 134.4, 132.0, 129.4, 120.7, 113.8,

53.2, 49.9, 18.1.

LRMS: *m/z*(%)= 300.1 (100), 302.1 (65) [M + H]$^+$.

**Methyl (2-allylamino-6-chloroquinazolin-4-yl)-*D*-alaninate (40)**

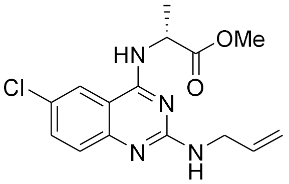


Methyl (2-allylamino-6-chloroquinazolin-4-yl)-*D*-alaninate (**40**) was prepared using General Procedure B, with methyl (2,6-dichloroquinazolin-4-yl)-*D*-alaninate (**39**) (270 mg, 0.88 mmol). The product was purified using flash column chromatography (40−80% EtOAc in cyclohexane) to give methyl (2-allylamino-6-chloroquinazolin-4-yl)-*D*-alaninate (**40**) (106 mg, 0.33 mmol, 37%) as a yellow solid.

IR $\nu_{max}$/cm$^{-1}$(thin film): 1727 (C=O).

$^1$H NMR (400 MHz, $CDCl_3$) δ 7.53 (d, *J* = 2.3 Hz, 1H), 7.43 (dd, *J* = 9.0, 2.3 Hz, 1H), 7.32 (d, *J* = 9.0 Hz, 1H), 6.18 (d, *J* = 6.7 Hz, 1H), 6.03 – 5.85 (m, 1H), 5.32 – 5.18 (m, 1H), 5.13 – 5.07 (m, 1H), 5.05 (s, 1H), 4.92 – 4.80 (m, 1H), 4.14 – 4.02 (m, 2H), 3.80 (s, 3H), 1.58 (d, *J* = 7.2 Hz, 3H).

$^{13}$C NMR (101 MHz, $CDCl_3$) δ 174.5, 159.3, 158.7, 150.8, 135.8, 133.4, 127.2, 126.1, 120.7, 115.6, 52.7, 49.7, 44.1, 18.2.

LRMS: *m/z*(%)= 321.2 (100), 323.2 (34) [M + H]$^+$.

**(*R*)-2-(2-Allylamino-6-chloroquinazolin-4-yl)amino-*N*-hydroxypropanamide (12)**

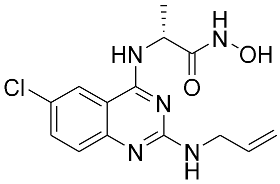


*R*)-2-(2-allylamino-6-chloroquinazolin-4-yl)amino-*N*-hydroxypropanamide (**12**) was prepared using General Procedure D, with methyl (2-allylamino-6-chloroquinazolin-4-yl)-*D*-alaninate (**40**) (90 mg, 0.25 mmol). The product was purified using flash column chromatography (1−10% MeOH in DCM + 0.5% (v/v) formic acid) to give (*S*)-2-(2-Allylamino- 6-chloroquinazolin-4-yl)amino-*N*-hydroxypropanamide (**12**) (72 mg, 0.22 mmol, 80%) as a white solid.

IR $\nu_{max}$/cm$^{-1}$(solid): 3383 (O-H) and 1652 (C=O).

$^1$H NMR (600 MHz, DMSO-*d$_6$*) δ 10.56 (s, 1H), 8.80 (s, 1H), 8.36 – 8.28 (m, 1H), 8.00 (s, 1H), 7.60 – 7.50 (m, 1H), 7.33 – 7.22 (m, 1H), 6.83 (s, 1H), 5.97 – 5.81 (m, 1H), 5.19 (dt, J = 17.2, 1.8 Hz, 1H), 5.05 (dt, J = 10.2, 1.8 Hz, 1H), 4.62 (p, J = 7.1 Hz, 1H), 3.97 (s, 2H), 1.41 (d, J = 7.1 Hz, 3H).

$^{13}$C NMR (151 MHz, DMSO-*d$_6$*) δ 169.4, 158.8, 158.5, 149.4, 136.3, 132.7, 124.0, 122.9, 115.0, 43.1, 17.7.

LRMS: *m/z*(%)= 321.1 (100), 322.1 (36) [M + H]$^+$.

$[a]\frac{25}{D}$ = −2.1 (c = 0.5 in MeOH).

**Methyl (2,6-dichloroquinazolin-4-yl-L-alaninate) (41)**

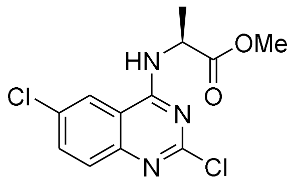


Methyl (2,6-dichloroquinazolin-4-yl-*L*-alaninate) (**41**) was prepared using General Procedure A, with 2,4,6-trichloroquinazoline (**31**) (500 mg, 2.1 mmol) and *L*-alanine methyl ester hydrochloride (450 mg, 3.2 mmol). Methyl (2,6-dichloroquinazolin-4-yl)-*L*-alaninate (**41**) (605 mg, 2.0 mmol, 95%) was obtained as a yellow solid.

IR $\nu_{max}$/cm$^{-1}$(thin film): 1719 (C=O).

$^{1}$H NMR (400 MHz, $CDCl_3$) δ 7.65 (dd, *J* = 2.1, 0.7 Hz, 1H), 7.57 (dd, *J* = 8.9, 2.1 Hz, 1H), 7.53 (dd, *J* = 8.9, 0.6 Hz, 1H), 7.02 (d, *J* = 7.1 Hz, 1H), 5.02 (p, *J* = 7.2 Hz, 1H), 3.90 (s, 3H), 1.61 (d, *J* = 7.2 Hz, 3H).

$^{13}$C NMR (101 MHz, $CDCl_3$) δ 174.8, 159.2, 157.4, 149.1, 134.2, 131.8, 129.1, 120.5, 113.6, 53.0, 49.8, 17.8.

LRMS: *m/z*(%)= 300.1 (100), 302.1 (63) [M + H]$^+$; HRMS: calcd. $C_{12}H_{12}O_2N_3{}^{35}Cl_2$ [M + H]$^+$: 300.0301; observed: 300.0303.

**Methyl (2-allylamino-6-chloroquinazolin-4-yl)-*L*-alaninate (42)**

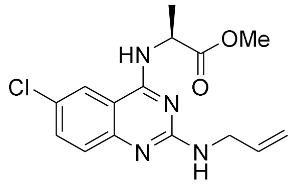


Methyl (2-allylamino-6-chloroquinazolin-4-yl)-L-alaninate (**42**) was prepared using General Procedure B, using methyl (2,6-dichloroquinazolin-4-yl-*L*-alaninate) (**41**) (300 mg, 1.00 mmol). The product was purified using flash column chromatography (40−80% EtOAc in cyclohexane) to give methyl (2-allylamino-6-chloroquinazolin-4-yl)-*L*-alaninate (**42**) (193 mg, 0.60 mmol, 60%) as a yellow solid.

IR $\nu_{max}$/cm$^{-1}$(thin film): 1722 (C=O).

$^{1}$H NMR (400 MHz, $CDCl_3$) δ 7.53 (d, *J* = 2.2 Hz, 1H), 7.44 (dd, *J* = 8.9, 2.3 Hz, 1H), 7.33 (d, *J* = 8.9 Hz, 1H), 6.14 (d, *J* = 6.7 Hz, 1H), 6.05 – 5.90 (m, 1H), 5.25 (dd, *J* = 17.1, 1.6 Hz, 1H), 5.12 (dd, *J* = 10.3, 1.6 Hz, 1H), 5.06 (s, 1H), 4.86 (p, *J* = 7.0 Hz, 1H), 4.10 (tt, *J* = 5.8, 1.7 Hz, 2H), 3.80 (s, 3H), 1.57 (d, *J* = 7.1 Hz, 3H).

$^{13}$C NMR (101 MHz, $CDCl_3$) δ 174.5, 159.3, 158.7, 150.8, 135.8, 133.4, 127.2, 126.1, 120.7, 115.6, 52.7, 49.7, 44.1, 18.2.

LRMS: *m/z*(%)= 321.2 (100), 322.1 (34) [M + H]$^+$; HRMS: calcd. $C_{15}H_{18}O_2N_4{}^{35}Cl$ [M + H]$^+$: 321.1113;

observed: 321.1113.

**(*S*)-2-(2-Allylamino-6-chloroquinazolin-4-yl)amino-*N*-hydroxypropanamide (13)**

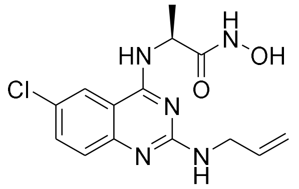

(*S*)-2-(2-Allylamino-6-chloroquinazolin-4-yl)amino-*N*-hydroxypropanamide (**13**) was prepared using General Procedure D, with methyl (2-allylamino-6-chloroquinazolin-4-yl)-*L*-

alaninate (**42**) (190 mg, 0.59 mmol). The product was purified using flash column chromatography (2−15% MeOH in DCM) to give (*S*)-2-(2-Allylamino-6-chloroquinazolin-4- yl)amino-*N*-hydroxypropanamide (**13**) (137 mg, 0.43 mmol, 72%) as a white solid.

IR $ν_{max}$/cm$^{-1}$(solid): 1656 (C=O).

$^1$H NMR (400 MHz, DMSO-*d*$_6$) δ 10.55 (s, 1H), 8.86 (s, 1H), 8.30 (d, *J* = 2.3 Hz, 1H), 7.97 (s, 1H), 7.48 (dd, *J* = 8.9, 2.4 Hz, 1H), 7.23 (d, *J* = 8.9 Hz, 1H), 6.79 (s, 1H), 5.92 (td, *J* = 10.9, 5.0 Hz, 1H), 5.18 (dd, *J* = 17.3, 1.9 Hz, 1H), 5.03 (d, *J* = 10.8 Hz, 1H), 4.62 (p, *J* = 6.6 Hz, 1H), 4.16 – 3.81 (m, 2H), 1.41 (d, *J* = 7.1 Hz, 3H).

$^{13}$C NMR (101 MHz, DMSO-*d*$_6$) δ 169.5, 159.0, 158.8, 152.0, 150.5, 136.5, 132.5, 123.7, 122.8, 114.8, 43.1, 17.7.

LRMS: *m/z*(%)= 322.2 (100), 324.1 (39) [M + H]$^+$; HRMS: calcd. $C_{14}H_{17}O_2N_5{}^{35}Cl$ [M + H]$^+$: 322.1065 ; observed: 322.1066.

$[a]\frac{25}{D}$ = +3.1 (c = 0.5 in MeOH).

**Methyl (2,6-dichloroquinazolin-4-yl)-serinate (43)**

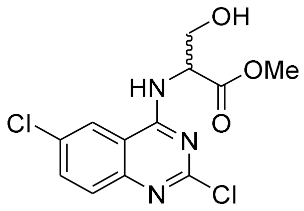

Methyl (2,6-dichloroquinazolin-4-yl)-serinate (**43**) was prepared using General Procedure A, with 2,4,6-trichloroquinazoline (**31**) (500 mg, 2.1 mmol) and *DL*-serine methyl ester hydrochloride (500 mg, 3.2 mmol). Methyl (2,6-dichloroquinazolin-4-yl)-serinate (**13**) (572 mg, 1.8 mmol, 85%) was obtained as a white solid.

IR $ν_{max}$/cm$^{-1}$(solid): 3230 (O-H) and 1736 (C=O).

$^1$H NMR (400 MHz, DMSO-*d*$_6$) δ 9.01 (d, *J* = 7.1 Hz, 1H), 8.72 (d, *J* = 2.3 Hz, 1H), 7.93 (dd, *J* = 8.9, 2.3 Hz, 1H), 7.74 (d, *J* = 8.9 Hz, 1H), 4.89 (dt, *J* = 7.2, 5.7 Hz, 1H), 3.96 (d, *J* = 5.7 Hz, 2H), 3.73 (s, 3H).

$^{13}$C NMR (101 MHz, DMSO-*d*$_6$) δ 170.8, 160.9, 157.1, 149.5, 134.8, 130.9, 129.3, 123.4, 114.7, 61.0, 57.5, 52.6.

LRMS: *m/z*(%)= 316.1 (100), 318.1 (66) [M + H]$^+$; HRMS: calcd. $C_{12}H_{12}O_3N_3{}^{35}Cl_2$ [M + H]$^+$: 316.0250 ;

observed: 316.0251.

**Methyl (2-allylamino-6-chloroquinazolin-4-yl)-serinate (44)**

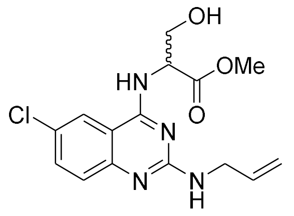


Methyl (2-allylamino-6-chloroquinazolin-4-yl)-serinate (**44**) was prepared using General Procedure B, with methyl (2,6-dichloroquinazolin-4-yl)-serinate (**43**) (300 mg, 0.95 mmol). Purification using flash column chromatography (0-10% MeOH in DCM) gave methyl (2- allylamino-6-chloroquinazolin-4-yl)-serinate (**44**) (110 mg, 0.33 mmol, 34%) as a white solid.

IR $\nu_{max}/cm^{-1}$(solid): 3356 (O-H) and 1735 (C=O).

$^1$H NMR (400 MHz, CDCl3) δ 7.44 (d, *J* = 2.3 Hz, 1H), 7.40 (dd, *J* = 8.9, 2.2 Hz, 1H), 7.28 (d, *J* = 9.0 Hz, 1H), 6.63 (s, 1H), 5.92 (ddt, *J* = 17.1, 10.5, 5.3 Hz, 2H), 5.22 (dd, *J* = 17.1, 1.6 Hz, 1H), 5.10 (dd, *J* = 10.2, 1.5 Hz, 1H), 4.99 – 4.89 (m, 1H), 4.23 (dd, *J* = 11.4, 3.2 Hz, 1H), 4.11 (dd, *J* = 11.4, 3.9 Hz, 1H), 4.03 (s, 2H), 3.83 (s, 3H).

$^{13}$C NMR (101 MHz, $CDCl_3$) δ 171.8, 159.0, 135.5, 133.7, 126.4, 120.9, 115.8, 111.3, 77.5, 77.2, 76.8, 62.8, 56.6, 53.0, 44.1.

LRMS: *m/z*(%)= 337.1 (100), 339.1 (35) $[M + H]^+$; HRMS: calcd. $C_{15}H_{18}O_3N_4{}^{35}Cl$ $[M + H]^+$: 337.1062 ; observed: 337.1063.

**2-(2-Allylamino-6-chloroquinazolin-4-yl)amino-*N*,3-dihydroxypropanamide (14)**

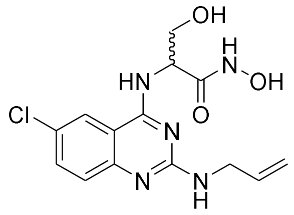


2-(2-Allylamino-6-chloroquinazolin-4-yl)amino-*N*,3-dihydroxypropanamide (**14**) was prepared using General Procedure D, with methyl (2-allylamino-6-chloroquinazolin-4-yl)- serinate (**44**) (85 mg, 0.25 mmol). The product was purified using flash column chromatography (5−20% MeOH in DCM) to give 2-(2-Allylamino-6-chloroquinazolin-4- yl)amino-*N*,3-dihydroxypropanamide (**14**) (47 mg, 0.14 mmol, 56%) as a white solid.

IR $\nu_{max}/cm^{-1}$(solid): 3242 (O-H) and 1654 (C=O).

$^1$H NMR (500 MHz, DMSO-$d_6$) δ 10.56 (s, 1H), 8.40 – 8.31 (m, 1H), 7.54 (d, *J* = 8.9 Hz, 0H), 7.30 (d, *J* = 8.9 Hz, 1H), 6.93 (s, 1H), 6.06 – 5.83 (m, 1H), 5.20 (dd, *J* = 17.2, 1.8 Hz, 1H), 5.06 (dd, *J* = 10.3, 1.7 Hz, 1H), 4.67 (q, *J* = 6.6 Hz, 1H), 4.05 – 3.96 (m, 3H), 3.79 (t, *J* = 5.2 Hz, 2H).

$^{13}$C NMR (126 MHz, DMSO-$d_6$) δ 166.9, 159.2, 158.2, 136.1, 132.9, 126.2, 124.2, 123.1, 115.1, 111.8, 61.2, 55.4, 43.1.

LRMS: *m/z*(%)= 338.1 (100), 340.1 (34) $[M + H]^+$; HRMS: calcd. C H O N $^{35}$Cl $[M + H]^+$: 338.1016 ; observed: 338.1014.

**Scheme S-7. Synthesis of compounds 15-19**

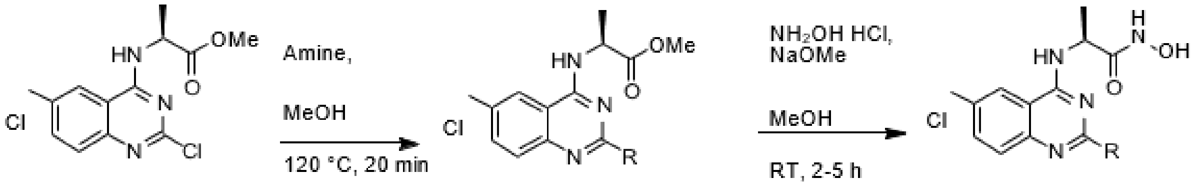


**41** | **45, 46, 47, 48, 49** | **15, 16, 17, 18, 19**

| # | R |
|---|---|
| 45, 15 | 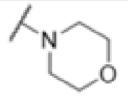 |
| 46, 16 | 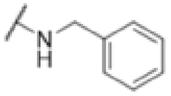 |
| 47, 17 | 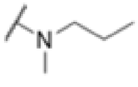 |
| 48, 18 | 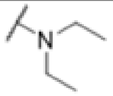 |
| 49, 19 | 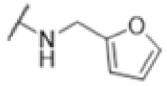 |

## Methyl (6-chloro-2-morpholinoquinazolin-4-yl)-*L*-alaninate (45)

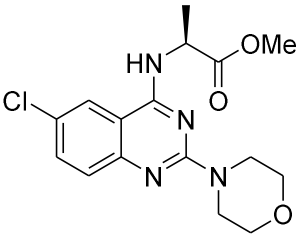

Morpholine (0.5 mL, 5.7 mmol) was added to a solution of methyl (2,6-dichloroquinazolin-4- yl-*L*-alaninate) (**41**) (400 mg, 1.33 mmol) in MeOH (4.5 mL). The reaction mixture was stirred for 20 min at 120 °C in a microwave reactor. The mixture was acidified to pH 2 with 4 M HCl in 1,4-dioxane and concentrated *in vacuo*. The product was purified using flash column chromatography (10−50% EtOAc in cyclohexane) to give methyl (6-chloro-2- morpholinoquinazolin-4-yl)-*L*-alaninate (**45**) (352 mg, 1.10 mmol, 82%) as a yellow solid.

IR $\nu_{max}$/cm$^{-1}$(thin film):1730 (C=O).

$^{1}H$ NMR (400 MHz, $CDCl_3$) δ 7.49 (d, *J* = 2.3 Hz, 1H), 7.39 (dd, *J* = 8.9, 2.3 Hz, 1H), 7.30 (d, *J* = 8.8 Hz, 1H), 6.21 (d, *J* = 6.5 Hz, 1H), 4.86 – 4.64 (m, 1H), 3.83 (dd, *J* = 6.5, 4.2 Hz, 4H), 3.80 (s, 3H), 3.76 (ddd, *J* = 5.7, 3.3, 1.2 Hz, 4H), 1.58 (d, *J* = 7.2 Hz, 3H).

$^{13}C$ NMR (101 MHz, $CDCl_3$) δ 174.8, 158.7, 158.4, 150.9, 133.4, 127.6, 126.1, 120.6, 110.9, 67.2, 52.6, 50.1, 44.4, 17.8.

LRMS: *m/z*(%)= 351.2 (100), 353.2 (33) $[M + H]^+$; HRMS: calcd. $C_{16}H_{20}O_3N_4{}^{35}Cl$ $[M + H]^+$: 351.1218 ; observed: 351.1219.

**(*S*)-2-(6-Chloro-2-morpholinoquinazolin-4-yl)amino-*N*-hydroxypropanamide (15)**

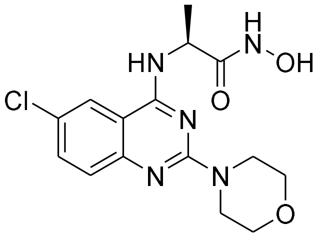


(*S*)-2-(6-Chloro-2-morpholinoquinazolin-4-yl)amino-*N*-hydroxypropanamide (**15**) was prepared using General Procedure D, with methyl (6-chloro-2-morpholinoquinazolin-4-yl)-*L*- alaninate (**45**) (250 mg, 0.71 mmol). The product was purified using flash column chromatography (5−20% MeOH in DCM) to give (*S*)-2-(6-chloro-2-morpholinoquinazolin-4- yl)amino-*N*-hydroxypropanamide (**15**) (224 mg, 0.64 mmol, 90%) as a white solid.

IR $\nu_{max}/cm^{-1}$(solid): 3208 (O-H) and 1644 (C=O).

$^{1}H$ NMR (400 MHz, DMSO-$d_6$) δ 10.66 (d, *J* = 1.7 Hz, 1H), 8.76 (d, *J* = 1.7 Hz, 1H), 8.35 (d, *J* = 2.4 Hz, 1H), 8.02 (d, *J* = 6.6 Hz, 1H), 7.51 (dd, *J* = 8.9, 2.4 Hz, 1H), 7.28 (d, *J* = 8.9 Hz, 1H), 4.52 (p, *J* = 6.9 Hz, 1H), 3.72 (dd, *J* = 5.9, 3.7 Hz, 4H), 3.63 (t, *J* = 4.7 Hz, 4H), 1.42 (d, *J* = 7.2 Hz, 3H).

$^{13}C$ NMR (101 MHz, DMSO-$d_6$) δ 169.7, 158.7, 158.5, 150.4, 132.6, 126.9, 124.2, 122.8, 111.3, 66.2, 48.6, 48.4, 44.0, 17.9.

LRMS: *m/z*(%)= 352.2 (100), 354.2 (34) $[M + H]^+$; HRMS: calcd. $C_{15}H_{19}O_3N_5{}^{35}Cl$ $[M + H]^+$: 352.11706 ; observed: 352.11740.

[a]$\frac{25}{D}$ 25 = −6.9 (c = 0.9 in MeOH).

**Methyl (6-chloro-2-benzylaminoquinazolin-4-yl)-*L*-alaninate (46)**

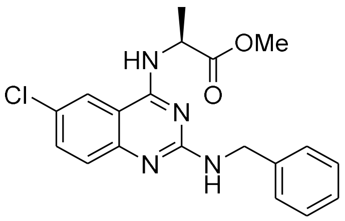


Benzylamine (0.50 mL, 4.60 mmol) was added to a solution of methyl (2,6- dichloroquinazolin-4-yl-*L*-alaninate) (**41**) (400 mg, 1.33 mmol) in MeOH (3 mL). The reaction mixture was stirred for 20 min at 120 °C in a microwave reactor. The mixture was then acidified to pH 2 with 4 M HCl in 1,4-dioxane and concentrated *in vacuo*. The product was purified using flash column chromatography (10−50% EtOAc in

cyclohexane) to give methyl (6-chloro-2-benzylaminoquinazolin-4-yl)-*L*-alaninate (**46**) (237 mg, 0.68 mmol, 51%) as a white solid.

IR $\nu_{max}/cm^{-1}$(thin film):1718 (C=O).

$^{1}$H NMR (400 MHz, $CDCl_3$) δ 7.54 (d, *J* = 2.3 Hz, 1H), 7.44 (dd, *J* = 8.9, 2.3 Hz, 1H), 7.39 – 7.29 (m, 5H), 7.29 – 7.20 (m, 1H), 6.22 (d, *J* = 6.8 Hz, 1H), 5.35 (s, 1H), 4.82 (p, *J* = 7.0 Hz, 1H), 4.67 (d, *J* = 5.8 Hz, 2H), 3.75 (s, 3H), 1.53 (d, *J* = 7.2 Hz, 3H).

$^{13}$C NMR (101 MHz, $CDCl_3$) δ 174.4, 159.2, 158.6, 150.7, 139.8, 133.3, 128.5, 127.5, 127.1, 126.0, 120.5, 52.5, 49.6, 45.5, 18.0.

LRMS: *m/z*(%)= 371.1 (100), 373.2 (33) $[M + H]^+$; HRMS: calcd. $C_{19}H_{20}O_2N_4{}^{35}Cl$ $[M + H]^+$: 371.1269 ; observed: 371.1270.

**(*S*)-2-(6-Chloro-2-benzylaminoquinazolin-4-yl)amino-*N*-hydroxypropanamide (16)**

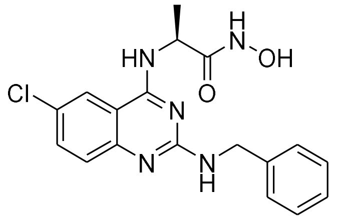

(*S*)-2-(6-Chloro-2-benzylaminoquinazolin-4-yl)amino-*N*-hydroxypropanamide (**16**) was prepared using General Procedure D with methyl (6-chloro-2-benzylaminoquinazolin-4-yl)-*L*- alaninate (**46**) (200 mg, 0.54 mmol). Purification using flash column chromatography (5-20% MeOH in DCM) gave (*S*)-2-(6-chloro-2-benzylaminoquinazolin-4-yl)amino-*N*- hydroxypropanamide (**16**) (187 mg, 0.50 mmol, 93%) as a white solid.

IR $\nu_{max}/cm^{-1}$(solid): 3218 (O-H) and 1644 (C=O).

$^{1}$H NMR (500 MHz, DMSO-$d_6$) δ 10.88 (s, 1H), 9.38 (s, 1H), 8.62 (s, 1H), 8.57 (s, 1H), 7.82 (s, 1H), 7.50 (d, *J* = 8.9 Hz, 1H), 7.42 (d, *J* = 7.5 Hz, 2H), 7.36 (t, *J* = 7.5 Hz, 2H), 7.28 (t, *J* = 7.3 Hz, 1H), 4.74 (t, *J* = 7.1 Hz, 2H), 4.59 (d, *J* = 16.4 Hz, 1H), 1.47 (d, *J* = 7.2 Hz, 4H).

$^{13}$C NMR (126 MHz, DMSO-$d_6$) δ 168.6, 163.6, 159.4, 157.6, 153.8, 139.1, 135.4, 129.0, 128.3, 127.7, 124.6, 120.1, 111.3, 79.7, 50.0, 44.7, 18.0.

LRMS: *m/z*(%)= 372.2 (100), 374.2 (28) $[M + H]^+$; HRMS: calcd. $C_{18}H_{19}O_2N_5{}^{35}Cl$ $[M + H]^+$: 372.12218; observed: 372.12231.

$[a]^{25}_{D}$ = −15.2 (c = 0.8 in MeOH).

**Methyl (6-chloro-2-methyl(propyl)aminoquinazolin-4-yl)-*L*-alaninate (47)**

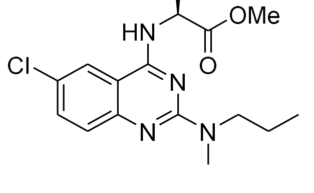

*N*-Methylpropylamine (0.29 mL, 3.00 mmol) was added to a solution of methyl (2,6- dichloroquinazolin-4-yl-*L*-alaninate) (**41**) (300 mg, 1.00 mmol) in MeOH (3 mL). The reaction mixture was stirred for 20 min at 120 °C in a microwave reactor. The reaction mixture was acidified with 4 M HCl, then concentrated *in*

*vacuo*. The product was purified using flash column chromatography (20-60% EtOAc in cyclohexane) to give methyl (6-chloro-2- methyl(propyl)aminoquinazolin-4-yl)-*L*-alaninate (**47**) (247 mg, 0.73 mmol, 73%) as a white solid.

IR $\nu_{max}$/cm$^{-1}$(thin film): 1732 (C=O).

$^1$H NMR (400 MHz, $CDCl_3$) δ 7.48 (d, *J* = 2.3 Hz, 1H), 7.37 (dd, *J* = 9.0, 2.3 Hz, 1H), 7.32 (d, *J* = 8.9 Hz, 1H), 6.01 (d, *J* = 6.6 Hz, 1H), 4.78 (p, *J* = 7.1 Hz, 1H), 3.79 (s, 3H), 3.59 (tq, *J* = 13.6, 7.0 Hz, 2H), 3.17 (s, 3H), 1.69 – 1.59 (m, 2H), 1.58 (d, *J* = 7.2 Hz, 3H), 0.93 (t, *J* = 7.4 Hz, 3H).

$^{13}$C NMR (101 MHz, $CDCl_3$) δ 175.0, 159.4, 158.4, 151.7, 133.4, 127.8, 125.4, 120.8, 110.7, 52.8, 51.4, 50.3, 35.6, 21.4, 18.4, 11.8.

LRMS: *m/z*(%)= 337.2 (100), 339.2 (33) [M + H]$^+$; HRMS: calcd. $C_{16}H_{22}O_2N_4{}^{35}Cl$ [M + H]$^+$: 337.1426 ; observed: 337.1425.

**(*S*)-2-(6-chloro-2-methyl(propyl)aminoquinazolin-4-yl)amino-*N*-hydroxypropanamide formate (17)**

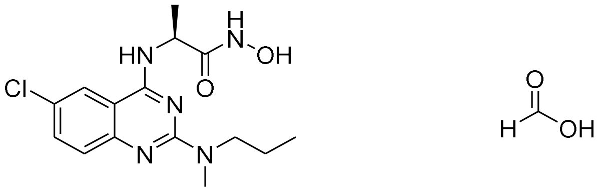

(*S*)-2-(6-chloro-2-methyl(propyl)aminoquinazolin-4-yl)amino-*N*-hydroxypropanamide formate (**17**) was prepared using General Procedure D, with methyl (6-chloro-2- methyl(propyl)aminoquinazolin-4-yl)-*L*-alaninate (**47**) (150 mg, 0.45 mmol). Purification using flash column chromatography (5-20% MeOH in DCM + 0.5% (v/v) Formic acid) gave (*S*)-2-(6-chloro-2-methyl(propyl)aminoquinazolin-4-yl)amino-*N*- hydroxypropanamide formate (**17**) (127 mg, 0.38 mmol, 83%) as a white solid.

$^1$H NMR (400 MHz, DMSO-$d_6$) δ 10.87 (s, 1H), 9.06 (s, 1H), 8.58 (d, *J* = 2.3 Hz, 1H), 7.96 (d, *J* = 8.9 Hz, 1H), 7.72 (dd, *J* = 8.9, 2.3 Hz, 1H), 4.73 – 4.56 (m, 2H), 3.68 – 3.63 (m, 10H), 3.22 (s, 4H), 1.66 – 1.49 (m, 2H), 1.47 (d, *J* = 7.2 Hz, 3H), 0.87 (t, *J* = 7.3 Hz, 4H).

$^{13}$C NMR (101 MHz, DMSO-$d_6$) δ 168.7, 157.9, 157.2, 153.3, 134.2, 123.8, 121.4, 110.6, 49.5, 36.2, 20.2, 17.7, 11.0.

LRMS: *m/z*(%)= 338.2 (100), 340.2 (35) [M + H]$^+$; HRMS: calcd. $C_{15}H_{21}O_2N_5{}^{35}Cl$ [M + H]$^+$: 338.13783 observed: 338.13782.

$[a]\frac{25}{D}$ = −6.9 (c = 1.1 in MeOH).

**Methyl (6-chloro-2-diethylaminoquinazolin-4-yl)-*L*-alaninate (48)**

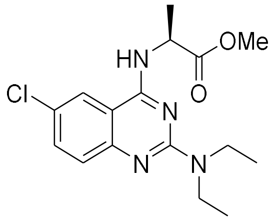

Diethylamine (0.5 mL, 4.8 mmol) was added to a solution of methyl (2,6-dichloroquinazolin- 4-yl-*L*-alaninate) (**41**) (300 mg, 1.00 mmol) in MeOH (3 mL). The reaction mixture was stirred for 20 min at 120

°C in a microwave reactor. The mixture was acidified to pH 2 with 4 M HCl in 1,4-dioxane and then concentrated *in vacuo*. Purification using flash column chromatography (10−50% EtOAc in cyclohexane) gave methyl (6-chloro-2- (diethylamino)quinazolin-4-yl)-*L*-alaninate (**48**) (208 mg, 0.62 mmol, 62%) as a yellow solid.

IR $\nu_{max}$/cm$^{-1}$(thin film):1731 (C=O).

$^1$H NMR (400 MHz, $CDCl_3$) δ 7.49 (d, *J* = 2.3 Hz, 1H), 7.40 (dd, *J* = 9.0, 2.3 Hz, 1H), 7.33 (d, *J* = 9.0 Hz, 1H), 5.85 (d, *J* = 6.5 Hz, 1H), 4.81 (p, *J* = 7.0 Hz, 1H), 3.78 (s, 3H), 3.65 (qd, *J* = 7.0, 4.6 Hz, 4H), 1.58 (d, *J* = 7.2 Hz, 3H), 1.19 (t, *J* = 7.0 Hz, 6H).

$^{13}$C NMR (101 MHz, $CDCl_3$) δ 174.5, 158.2, 151.6, 133.1, 127.6, 125.0, 120.4, 110.5, 52.5, 49.9, 41.7, 18.2, 13.7.

LRMS: *m/z*(%)= 337.2 (100), 339.2 (35) [M + H]$^+$; HRMS: calcd. $C_{16}H_{22}O_2N_4{}^{35}Cl$ [M + H]$^+$: 337.1426 ; observed: 337.1426.

**(*S*)-2-(6-Chloro-2-diethylaminoquinazolin-4-yl)amino-*N*-hydroxypropanamide (18)**

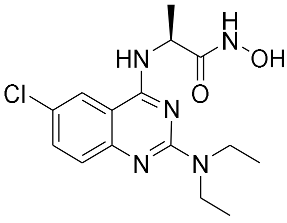

(**S**)-2-(6-Chloro-2-diethylaminoquinazolin-4-yl)amino-*N*-hydroxypropanamide (**18**) was prepared using General Procedure D, with methyl (6-chloro-2-diethylaminoquinazolin-4-yl)- *L*-alaninate (**48**) (150 mg, 0.45 mmol). The product was purified using flash column chromatography (5−20% MeOH in DCM) to give (*S*)-2-(6-chloro-2-(diethylamino)quinazolin- 4-yl)amino-*N*-hydroxypropanamide (**18**) (133 mg, 0.39 mmol, 88%) as a white solid.

IR $\nu_{max}$/cm$^{-1}$(solid): 3221 (O-H) and 1637 (C=O).

$^1$H NMR (400 MHz, DMSO-*d*$_6$) δ 10.82 (s, 1H), 9.31 (brs, 1H), 8.89 (brs, 1H), 8.61 (d, *J* = 2.2 Hz, 1H), 7.86 (dd, *J* = 8.9, 2.2 Hz, 1H), 7.76 (d, *J* = 9.0 Hz, 1H), 4.62 (app. p, *J* = 7.0 Hz, 1H), 3.67 (app. d, *J* = 7.2 Hz, 4H), 1.49 (d, *J* = 7.2 Hz, 3H), 1.20 (t, *J* = 7.0 Hz, 6H).

$^{13}$C NMR (101 MHz, DMSO-*d*$_6$) δ 168.6, 158.3, 151.1, 139.3, 135.1, 128.7, 124.5, 120.1, 111.0, 50.2, 18.1, 13.4.

LRMS: *m/z*(%)= 338.2 (100), 340.2 (27) [M + H]$^+$; HRMS: calcd. $C_{15}H_{21}O_2N_5{}^{35}Cl$ [M + H]$^+$: 338.13783; observed: 338.13791.

$[a]\frac{25}{D}$ = +3.9 (c = 0.6 in MeOH).

**Methyl (6-chloro-2-(furan-2-ylmethyl)aminoquinazolin-4-yl-*L*-alaninate (49)**

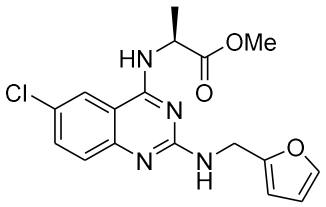

Furfurylamine (0.3 mL, 3.40 mmol) was added to a solution of methyl (2,6- dichloroquinazolin-4-yl-*L*-

alaninate) (**41**) (300 mg, 1.00 mmol) in MeOH (3 mL). The reaction mixture was stirred for 20 min at 120 °C in a microwave reactor. The mixture was acidified to pH 2 with 4 M HCl in 1,4-dioxane and concentrated *in vacuo*. Purification using flash column chromatography (20−60% EtOAc in cyclohexane) gave methyl (6-chloro-2-(furan-2- ylmethyl)aminoquinazolin-4-yl)-*L*-alaninate (**49**) (124 mg, 0.34 mmol, 34%) as an orange solid.

IR $v_{max}$/cm$^{-1}$(thin film):1731 (C=O).

$^1$H NMR (400 MHz, $CDCl_3$) δ 7.54 (d, *J* = 2.3 Hz, 1H), 7.46 (dd, *J* = 8.9, 2.2 Hz, 1H), 7.42 – 7.33 (m, 2H), 6.31 (dd, *J* = 3.2, 1.9 Hz, 1H), 6.24 (d, *J* = 2.3 Hz, 1H), 6.13 (d, *J* = 6.7 Hz, 1H), 5.37 (s, 1H), 4.87 (p, *J* = 7.1 Hz, 1H), 4.66 (d, *J* = 5.5 Hz, 2H), 3.79 (s, 3H), 1.57 (d, *J* = 7.1 Hz, 3H).

$^{13}$C NMR (101 MHz, $CDCl_3$) δ 174.4, 159.0, 158.7, 142.0, 133.5, 126.4, 120.7, 110.5, 106.8, 52.7, 49.7, 38.8, 18.2.

DIPEA
1 h

LRMS: *m/z*(%)= 361.1 (100), 363.2 (36) [M + H]$^+$; HRMS: calcd. $C_{17}H_{18}O_3N_4{}^{35}Cl$ [M + H]$^+$: 361.1062 observed: 361.1063.

NH$_2$OH HCl,
NaOMe

**(*S*)-2-(6-Chloro-2-(furan-2-ylmethyl)aminoquinazolin-4-yl)amino-*N*-hydroxypropanamide (19)**

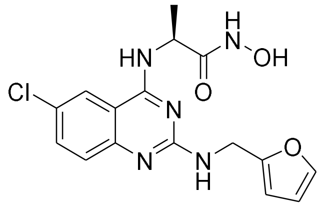

(*S*)-2-(6-Chloro-2-(furan-2-ylmethyl)aminoquinazolin-4-yl)amino-*N*-hydroxypropanamide (**19**) was prepared using General Procedure D, with methyl (6-chloro-2-(furan-2- ylmethyl)aminoquinazolin-4-yl)-*L*-alaninate (**49**) (100 mg, 0.28 mmol). Purification using flash column chromatography (5−20% MeOH in DCM) gave (*S*)-2-(6-chloro-2-(furan-2- ylmethyl)aminoquinazolin-4-yl)amino-*N*-hydroxypropanamide (**19**) (42 mg, 0.12 mmol, 42%) as a brown solid.

IR $v_{max}$/cm$^{-1}$(solid): 3212 (O-H) and 1644 (C=O).

$^1$H NMR (500 MHz, DMSO-*d*$_6$) δ 10.89 (s, 1H), 9.57 (d, *J* = 6.6 Hz, 1H), 8.96 (s, 1H), 8.66 (s, 1H), 8.63 (s, 1H), 7.85 (d, *J* = 8.3 Hz, 1H), 7.62 (s, 1H), 7.51 (d, *J* = 8.5 Hz, 1H), 6.45 (s, 1H), 6.42 (s, 1H), 4.80 – 4.71 (m, 2H), 4.61 (dd, *J* = 15.7, 5.0 Hz, 1H), 1.48 (d, *J* = 7.3 Hz, 3H).

$^{13}$C NMR (126 MHz, DMSO-*d*$_6$) δ 167.9, 158.9, 152.5, 150.9, 142.7, 138.0, 135.3, 128.2, 124.3, 119.0, 110.8, 110.6, 108.2, 49.7, 37.4, 17.5.

LRMS: *m/z*(%)= 362.2 (100), 363.2 (40) [M + H]$^+$; HRMS: calcd. $C_{16}H_{17}O_3N_5{}^{35}Cl$ [M + H]$^+$: 362.10144 ; observed: 362.10138.

$[a]^{25}_{D}$ = −27.6 (c = 0.3 in MeOH).

**Scheme S-8. Synthesis of compound 20**

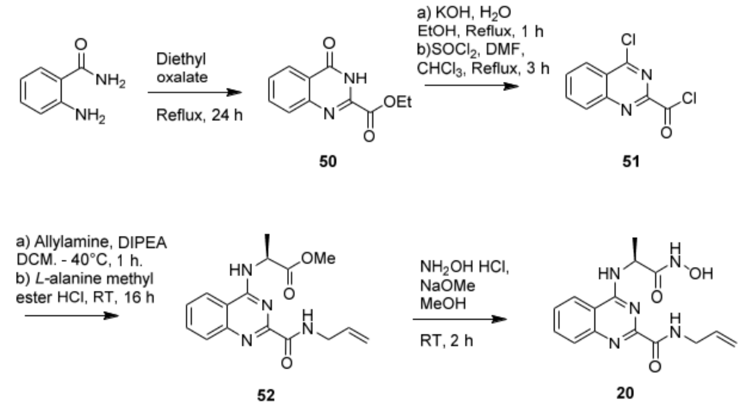


**Ethyl 4-oxo-3,4-dihydroquinazoline-2-carboxylate (50)**

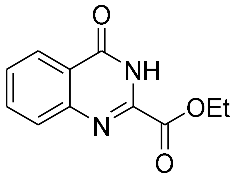

A mixture of 2-aminobenzamide (6 g, 44 mmol) and diethyl oxalate (24 mL, 176 mmol) was stirred under reflux for 24 h. The reaction mixture was then concentrated *in vacuo*. The product was recrystallized in EtOH to give ethyl 4-oxo-3,4-dihydroquinazoline-2-carboxylate (**50**) (6.3 g, 29 mmol, 66%) as a white solid.

$^1$H NMR (400 MHz, $CDCl_3$) δ 10.40 (s, 1H), 8.36 (ddd, *J* = 7.9, 1.6, 0.6 Hz, 1H), 7.96 (ddd, *J* = 8.2, 1.2, 0.6 Hz, 1H), 7.84 (ddd, *J* = 8.2, 7.2, 1.6 Hz, 1H), 7.62 (ddd, *J* = 7.9, 7.2, 1.2 Hz, 1H), 4.58 (q, *J* = 7.1 Hz, 2H), 1.49 (t, *J* = 7.1 Hz, 3H).

$^{13}$C NMR (101 MHz, $CDCl_3$) δ 161.0, 160.6, 147.6, 141.6, 135.0, 129.3, 129.3, 126.8, 123.2, 64.2, 14.2.

LRMS: *m/z*(%)= 219.2 (100) $[M + H]^+$.

**4-Chloroquinazoline-2-carbonyl chloride (51) and Methyl (2-(allylcarbamoyl)quinazolin-4- yl)-L-alaninate (52)**

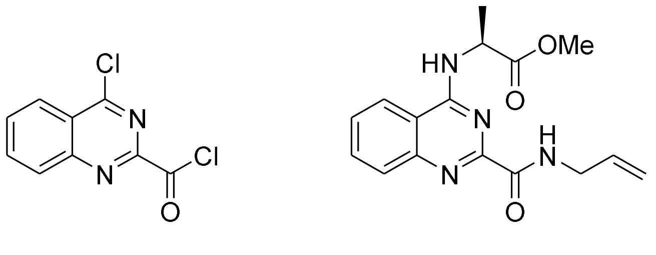

**51** **52**

NaOH (1.65 g, 41 mmol) was added to suspension of ethyl 4-oxo-3,4-dihydroquinazoline-2- carboxylate (**50**) (1.8 g, 8.3 mmol) in a mixture of $H_2O$/EtOH (1:1, 80 mL). The reaction mixture was stirred under reflux

for 1 h. The mixture was then cooled, acidified to pH 2 with 2 M $HCl_{(aq)}$ and left in an ice bath for 1 h to crystallize. The resultant precipitate was collected by filtration and dried to give intermediate 4-oxo-3,4-dihydroquinazoline-2- carboxylic acid. The intermediate was added to a solution of $SOCl_2$ (1.2 mL, 16 mmol) and DMF (5 drops) in $CHCl_3$ (15 mL). The reaction mixture was stirred under reflux for 3 h, and was then concentrated *in vacuo* to give 4-chloroquinazoline-2-carbonyl chloride (**51**) (1.7 g, 7.5 mmol, 91% over 2 steps) as a yellow solid. The crude product was used without further purification for the subsequent reaction.

Allylamine (300 µL, 4.10 mmol) and DIPEA (2 mL, 11 mmol) were added dropwise to a solution of crude 4-chloroquinazoline-2-carbonyl chloride (**51**) (650 mg, 2.90 mmol) in anhydrous DCM (10 mL) at −40 °C. The reaction mixture was stirred at −40 °C under an inert atmosphere for 1 h. After 1 h, *L*-alanine methyl ester hydrochloride (800 mg, 5.8 mmol) was added, the reaction mixture was stirred at room temperature for a further 16 h. The reaction mixture was then quenched with $H_2O$ (25 mL) and extracted with EtOAc (3 × 25 mL). The combined organic layers were washed with brine (50 mL), dried over $Na_2SO_4$ and concentrated *in vacuo*. The product was purified using flash column chromatography (50−100% EtOAc in cyclohexane) to give impure methyl (4-allylaminoquinazoline-2- carbonyl)-*L*-alaninate (**52**) (total mass 618 mg; based on $^1$HNMR analysis, ~50% of the mixture was the desired product, 1.00 mmol, 34%) as a yellow solid. The product was contaminated with *N*-allyl-4-(allylamino)quinazoline-2-carboxamide, and crude mixture used in the next step without further purification.

LRMS: *m/z*(%)= 315.2 (100) $[M + H]^+$

**(*S*)-*N*-Allyl-4-(1-hydroxyamino-1-oxopropan-2-yl)aminoquinazoline-2-carboxamide (20)**

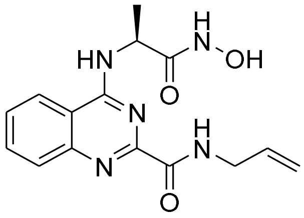


(*S*)-*N*-Allyl-4-(1-hydroxyamino-1-oxopropan-2-yl)aminoquinazoline-2-carboxamide (**20**) was prepared using General Procedure D, with methyl (2-(allylcarbamoyl)quinazolin-4-yl)-L- alaninate (**52**) (250 mg, 0.40 mmol, ~50% wt.). Purification using RP HPLC (MeCN in $H_2O$ + 0.1% (v/v) TFA) followed by lyophilization gave (*S*)-*N*-allyl-4-(1-hydroxyamino-1-oxopropan- 2-yl)aminoquinazoline-2-carboxamide (**20**) (47 mg, 0.15 mmol, 38%) as a white solid.

IR $\nu_{max}/cm^{-1}$(solid): 3226 (O-H) and 1648 (C=O).

$^1$H NMR (400 MHz, DMSO-$d_6$) δ 10.85 (s, 1H), 9.85 (d, *J* = 7.4 Hz, 1H), 9.26 (t, *J* = 6.2 Hz, 1H), 8.62 (dd, *J* = 8.5, 1.3 Hz, 1H), 8.08 (dd, *J* = 8.5, 1.4 Hz, 1H), 8.03 (ddd, *J* = 8.3, 6.9, 1.2 Hz, 1H), 7.79 (ddd, *J* = 8.3, 6.9, 1.4 Hz, 1H), 5.93 (ddt, *J* = 17.2, 10.3, 5.2 Hz, 1H), 5.32 (t, *J* = 7.2 Hz, 1H), 5.24 (dd, *J* = 17.2, 1.7 Hz, 1H), 5.15 (dd, *J* = 10.3, 1.6 Hz, 1H), 4.01 (tt, *J* = 5.2, 1.8 Hz, 2H), 1.52 (d, *J* = 7.1 Hz, 3H).

$^{13}$C NMR (101 MHz, DMSO-$d_6$) δ 168.0, 160.2, 159.5, 150.5, 135.5, 134.2, 128.4, 124.3, 122.8, 115.8, 113.6, 48.9, 41.7, 17.3.

LRMS: *m/z*(%)= 316.2 (100) $[M + H]^+$; HRMS: calcd. $C_{15}H_{18}O_3N_5$ $[M + H]^+$: 316.14042; observed: 316.14044.

$[a]^{25}_{D}$ = +33.3 (c = 0.8 in MeOH).

**Scheme. S-9**

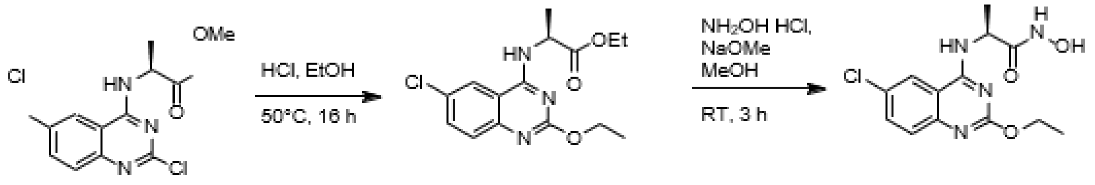


41 53 21

**Ethyl (6-chloro-2-ethoxyquinazolin-4-yl)-*L*-alaninate (53)**

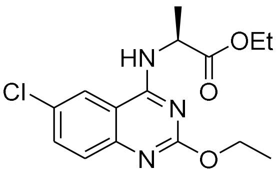


4 M HCl in 1,4-dioxane (2 mL) was added to a solution of methyl (2,6-dichloroquinazolin-4- yl-*L*-alaninate) (**41**) (200 mg, 0.67 mmol) in EtOH (2 mL). The reaction mixture was stirred at 50 °C for 16 h. The mixture was then quenched with sat. $NaHCO_{3(aq)}$ (25 mL) and extracted with DCM (3 × 25 ml). The combined organic layers were washed with brine (30 mL), dried over $Na_2SO_4$, then concentrated *in vacuo* to give ethyl (6-chloro-2-ethoxyquinazolin-4-yl)-*L*- alaninate (**53**) (182 mg, 0.56 mmol, 84% yield).

IR $\nu_{max}$/cm$^{-1}$(thin film): 1722 (C=O).

$^{1}$H NMR (400 MHz, $CDCl_3$) δ 7.63 (dd, *J* = 2.2, 0.7 Hz, 1H), 7.51 (dd, *J* = 8.9, 2.0 Hz, 1H), 7.47 (dd, *J* = 8.9, 0.7 Hz, 1H), 6.55 (d, *J* = 7.0 Hz, 1H), 5.01 (p, *J* = 7.2 Hz, 1H), 4.44 (qt, *J* = 7.0, 3.5 Hz, 2H), 4.29 (qt, *J* = 7.1, 3.5 Hz, 2H), 1.59 (d, *J* = 7.1 Hz, 3H), 1.43 (t, *J* = 7.1 Hz, 3H), 1.33 (t, *J* = 7.1 Hz, 3H).

$^{13}$C NMR (101 MHz, $CDCl_3$) δ 174.0, 162.3, 160.0, 150.2, 133.5, 128.4, 128.3, 120.5, 112.6, 62.8, 61.8, 49.5, 18.2, 14.7, 14.2.

LRMS: *m/z*(%)= 324.2 (100), 326.2 (34) [M + H]$^+$; HRMS: calcd. $C_{15}H_{19}O_3N_3{}^{35}Cl$ [M + H]$^+$: 324.1109 ; observed: 324.1110.

**(*S*)-2-(6-Chloro-2-ethoxyquinazolin-4-yl)amino-*N*-hydroxypropanamide (21)**

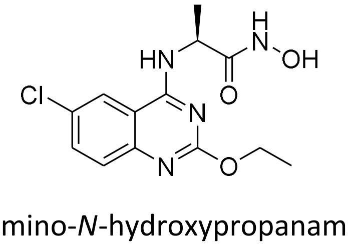


(***S***)-2-(6-Chloro-2-ethoxyquinazolin-4-yl)amino-*N*-hydroxypropanamide was prepared using General Procedure D, with ethyl (6-chloro-2-ethoxyquinazolin-4-yl)-*L*-alaninate (**53**) (150 mg, 0.48 mmol).

Purification using flash column chromatography (2−20% MeOH in DCM) gave (*S*)-2-(6-chloro-2-ethoxyquinazolin-4-yl)amino-*N*-hydroxypropanamide (**21**) (123 mg, 0.40 mmol, 82%) as a white solid.

IR $\nu_{max}/cm^{-1}$(solid): 3223 (O-H) and 1666 (C=O).

$^{1}H$ NMR (400 MHz, DMSO-$d_6$) δ 10.83 (s, 1H), 9.44 (s, 1H), 8.68 (d, *J* = 2.3 Hz, 1H), 7.87 (dd, *J* = 8.9, 2.3 Hz, 1H), 7.54 (d, *J* = 8.9 Hz, 1H), 5.02 – 4.69 (m, 1H), 4.51 (q, *J* = 7.1 Hz, 2H), 1.49 (d, *J* = 7.2 Hz, 3H), 1.36 (t, *J* = 7.1 Hz, 3H).

$^{13}C$ NMR (101 MHz, DMSO-$d_6$) δ 168.1, 160.7, 159.1, 135.0, 128.7, 123.9, 122.9, 112.1, 64.6, 49.5, 17.6, 14.2.

LRMS: *m/z*(%)= 311.1 (100), 313.1 (31) [M + H]$^+$; HRMS: calcd. $C_{13}H_{16}O_3N_4{}^{35}Cl$ [M + H]$^+$: 311.09054; observed: 311.09055.

$[a]_D^{25}$ = +29.2 (c = 0.6 in MeOH).

**Scheme. S-10**

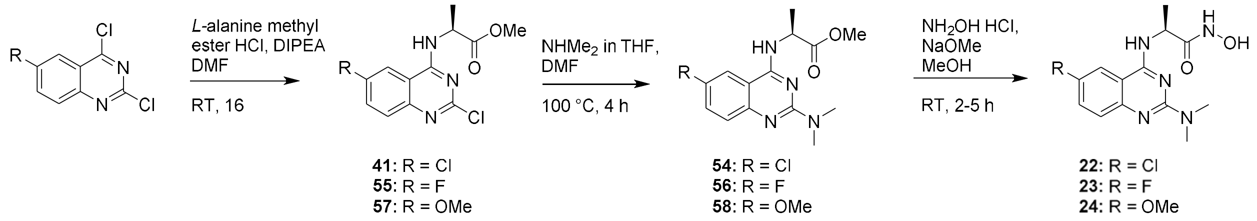


**Methyl (6-chloro-2-dimethylaminoquinazolin-4-yl)-*L*-alaninate (54)**

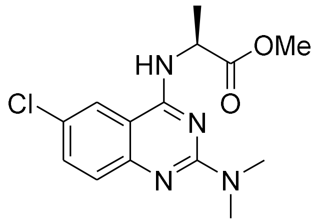

2 M $NHMe_2$ in THF (1.0 mL, 2.0 mmol) was added to a solution of methyl (2,6- dichloroquinazolin-4-yl-*L*-alaninate) (**41**) (200 mg, 0.67 mmol) in anhydrous DMF (3 mL). The reaction mixture was stirred for 4 h at 100 °C under an inert atmosphere and was then concentrated in vacuo. The product was purified using flash column chromatography (40−80% EtOAc in cyclohexane) to give methyl (6-chloro-2-dimethylaminoquinazolin-4-yl)-*L*- alaninate (**54**) (201 mg, 0.65 mmol, 98%) as a yellow solid.

IR $\nu_{max}/cm^{-1}$(thin film):1729 (C=O).

$^{1}H$ NMR (400 MHz, $CDCl_3$) δ 7.45 (d, *J* = 2.2 Hz, 1H), 7.35 (dd, *J* = 9.0, 2.3 Hz, 1H), 7.28 (dd, *J* = 8.9, 0.5 Hz, 1H), 6.19 (d, *J* = 6.5 Hz, 1H), 4.83 – 4.74 (m, 1H), 3.81 (s, 3H), 3.18 (s, 6H), 1.59 (t, *J* = 7.3 Hz, 3H).

$^{13}C$ NMR (101 MHz, $CDCl_3$) δ 175.4, 159.7, 158.4, 151.5, 133.4, 127.6, 125.5, 120.9, 110.6, 52.9, 50.3, 37.3, 18.2.

LRMS: *m/z*(%)= 309.1 (100), 311.1 (34) [M + H]$^+$; HRMS: calcd. $C_{14}H_{18}O_2N_4{}^{35}Cl$ [M + H]$^+$: 309.1113; observed: 309.1111.

**(*S*)-2-(6-Chloro-2-dimethylaminoquinazolin-4-yl)amino-*N*-hydroxypropanamide (22)**

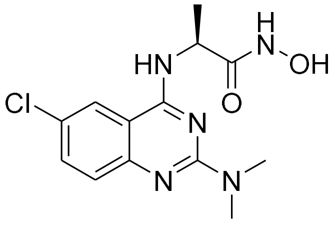

(*S*)-2-(6-Chloro-2-dimethylaminoquinazolin-4-yl)amino-*N*-hydroxypropanamide (**22**) was prepared using General Procedure D, with methyl (6-chloro-2-dimethylaminoquinazolin-4- yl)-*L*-alaninate (**54**) (100 mg, 0.32 mmol). Purification using RP HPLC (MeCN in $H_2O$ + 0.1% (v/v) TFA) and lyophilization gave (*S*)-2-(6-chloro-2-dimethylaminoquinazolin-4-yl)amino-*N*- hydroxypropanamide (**22**) (30 mg, 0.10 mmol, 30%) as a white solid.

IR $ν_{max}/cm^{-1}$(solid): 3234 (O-H) and 1645 (C=O).

$^1$H NMR (400 MHz, MeOD) δ 8.24 (d, *J* = 2.2 Hz, 1H), 7.71 (dd, *J* = 8.8, 2.0 Hz, 1H), 7.53 (d, *J* = 8.9 Hz, 1H), 4.67 (q, *J* = 7.2 Hz, 1H), 3.33 (s, 6H), 1.64 (d, *J* = 7.3 Hz, 3H).

13C NMR (101 MHz, MeOD) δ 171.7, 159.7, 153.5, 139.4, 136.4, 131.2, 124.7, 120.1, 111.7, 51.5, 38.7, 17.9.

LRMS: *m/z*(%)= 310.1 (100), 312.1 (33) $[M + H]^+$; HRMS: calcd. $C_{13}H_{17}O_2N_5{}^{35}Cl$ $[M + H]^+$: 310.1065 observed: 310.1066.

$[a]\frac{25}{D}$ = +5.7 (c = 1.2 in MeOH).

**Methyl (2-chloro-6-fluoroquinazolin-4-yl)-*L*-alaninate (55)**

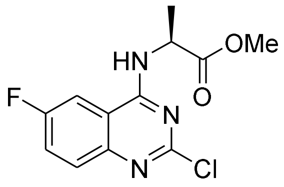

Anhydrous DIPEA (1.2 mL, 6.9 mmol) was added dropwise to a solution of 2,4-dichloro-6- fluoroquinazoline (500 mg, 2.3 mmol) and *L*-alanine methyl ester hydrochloride (480 mg, 3.4 mmol) in anhydrous DMF (5 mL) at 0 °C. The reaction mixture was stirred at room temperature under an inert atmosphere for 16 h and then diluted with cold $H_2O$ (75 mL).
The precipitate was collected by filtration and dried under vacuum to give methyl (2-chloro- 6-fluoroquinazolin-4-yl)-*L*-alaninate (**55**) (620 mg, 2.2 mmol, 94%) as a pale yellow solid.

IR $ν_{max}/cm^{-1}$(thin film): 1722 (C=O).

$^1$H NMR (400 MHz, $CDCl_3$) δ 7.65 (dd, *J* = 9.2, 5.1 Hz, 1H), 7.43 (ddd, *J* = 9.1, 8.1, 2.7 Hz, 1H), 7.33 (dd, *J* = 8.7, 2.7 Hz, 1H), 6.81 (d, *J* = 7.0 Hz, 1H), 5.01 (p, *J* = 7.2 Hz, 1H), 3.88 (s, 3H), 1.61 (d, *J* = 7.2 Hz, 3H).

$^{13}$C NMR (101 MHz, $CDCl_3$) δ 174.3, 161.0, 159.5 (d, *J* = 4.6 Hz), 158.5, 156.5, 147.3, 129.9 (d,

$J$ = 8.4 Hz), 122.9 (d, $J$ = 24.6 Hz), 113.2 (d, $J$ = 8.3 Hz), 105.5 (d, $J$ = 23.5 Hz), 52.8, 49.6, 17.7.

$^{19}$F NMR (377 MHz, CDCl3) δ -111.5.

LRMS: *m/z*(%)= 284.1 (100), 286.1 (34) [M + H]$^+$; HRMS: calcd. $C_{12}H_{12}O_2N_3{}^{35}ClF$ [M + H]$^+$: 284.0597; observed: 284.0596.

**Methyl (2-dimethylamino-6-fluoroquinazolin-4-yl)-*L*-alaninate (56)**

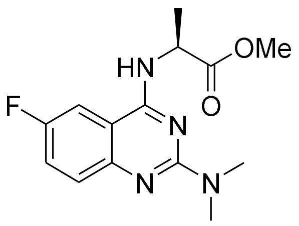


2 M $NHMe_2$ in THF (1.0 mL, 2.0 mmol) was added to a solution of methyl (2-chloro-6- fluoroquinazolin-4-yl)-*L*-alaninate (**55**) (200 mg, 0.70 mmol) in anhydrous DMF (3 mL). The reaction mixture was stirred at 100°C under an inert atmosphere for 4 h, then concentrated in vacuo. The product was purified using flash column chromatography (40−100% EtOAc in cyclohexane) to give methyl (2-dimethylamino-6-fluoroquinazolin-4-yl)-*L*-alaninate (**56**) (192 mg, 0.66 mmol, 93%) as a white solid.

IR $\nu_{max}$/cm$^{-1}$(thin film): 1731 (C=O).

$^1$H NMR (400 MHz, $CDCl_3$) δ 7.42 (dd, $J$ = 9.2, 5.2 Hz, 1H), 7.26 (ddd, $J$ = 9.2, 8.3, 2.8 Hz, 1H), 7.18 (dd, $J$ = 9.0, 2.8 Hz, 1H), 5.95 (d, $J$ = 6.4 Hz, 1H), 4.93 – 4.72 (m, 1H), 3.80 (s, 3H), 3.20 (s, 6H), 1.60 (d, $J$ = 7.1 Hz, 3H).

$^{13}$C NMR (101 MHz, $CDCl_3$) δ 175.1, 159.6, 158.9 (d, $J$ = 4.0 Hz), 158.4, 156.0, 149.9, 128.1 (d, $J$ = 7.8 Hz), 122.2 (d, $J$ = 24.1 Hz), 109.7 (d, $J$ = 7.9 Hz), 105.8 (d, $J$ = 22.8 Hz), 52.8, 50.3, 37.3, 18.3.

$^{19}$F NMR (376 MHz, $CDCl_3$) δ -121.1.

LRMS: *m/z*(%)= 293.1 (100) [M + H]$^+$; HRMS: calcd. $C_{14}H_{18}O_2N_4F$ [M + H]$^+$: 293.1408; observed: 293.1407.

**(*S*)-2-(2-Dimethylamino-6-fluoroquinazolin-4-yl)amino-*N*-hydroxypropanamide (23)**

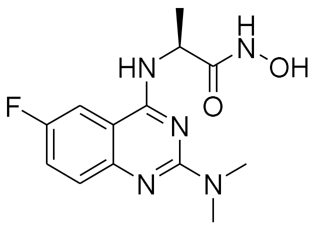


(*S*)-2-(2-Dimethylamino-6-fluoroquinazolin-4-yl)amino-*N*-hydroxypropanamide (**23**) was prepared using General Procedure D, with methyl (2-dimethylamino-6-fluoroquinazolin-4- yl)-*L*-alaninate (**56**) (100 mg, 0.34 mmol). Purification using RP HPLC (MeCN in $H_2O$ + 0.1% (v/v) TFA) and lyophilization gave (*S*)-2-(2-dimethylamino-6-fluoroquinazolin-4-yl)amino-*N*- hydroxypropanamide (**23**) (42 mg, 0.14 mmol, 42%) as a white solid.

IR $\nu_{max}$/cm$^{-1}$(solid): 3201 (O-H) and 1649 (C=O).

$^1$H NMR (400 MHz, MeOD) δ 8.03 (dd, *J* = 9.1, 2.6 Hz, 1H), 7.75 – 7.48 (m, 2H), 4.61 (q, *J* = 7.2 Hz, 1H), 3.29 (s, 6H), 1.60 (d, *J* = 7.3 Hz, 3H).

$^{13}$C NMR (101 MHz, MeOD) δ 171.6, 161.7, 160.3 (d, *J* = 2.8 Hz), 159.2, 153.7, 137.6, 124.7 (d, *J* = 24.7 Hz), 120.6 (d, *J* = 8.2 Hz), 111.7 (d, *J* = 8.5 Hz), 110.7 (d, *J* = 25.3 Hz), 51.6, 38.6, 17.9.

$^{19}$F NMR (376 MHz, MeOD) δ -76.9.

LRMS: *m/z*(%)= 294.1 (100), [M + H]$^+$; HRMS: calcd. $C_{13}H_{17}O_2N_5F$ [M + H]$^+$: 294.1361; observed: 294.1360.

$[a]_D^{25}$ = −26.0 (c = 1.0 in MeOH).

**Methyl (2-chloro-6-methoxyquinazolin-4-yl)-*L*-alaninate (57)**

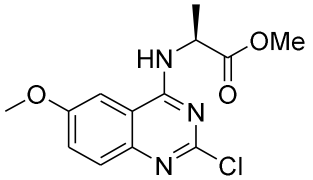

Anhydrous DIPEA (1.2 mL, 6.9 mmol) was added dropwise to a solution of 2,4-dichloro-6-methoxyquinazoline (500 mg, 2.2 mmol) and *L*-alanine methyl ester hydrochloride (480 mg, 3.4 mmol) at 0 °C. The reaction mixture was stirred 16 h at room temperature under an inert atmosphere and then diluted with cold $H_2O$ (75 mL). The precipitate was collected by filtration and dried under vacuum to give methyl (2-chloro-6-methoxyquinazolin-4-yl)-*L*- alaninate (**57**) (470 mg, 1.6 mmol, 73%) as a pale yellow solid.

IR $\nu_{max}$/cm$^{-1}$(solid): 1719 (C=O).

$^1$H NMR (400 MHz, MeOD) δ 7.57 (d, *J* = 2.7 Hz, 1H), 7.49 (d, *J* = 9.1 Hz, 1H), 7.37 (dd, *J* = 9.2, 2.7 Hz, 1H), 4.86 (q, *J* = 7.3 Hz, 1H), 3.91 (s, 3H), 3.77 (s, 3H), 1.62 (d, *J* = 7.4 Hz, 3H).

$^{13}$C NMR (101 MHz, MeOD) δ 175.5, 161.9, 159.5, 156.0, 146.6, 128.5, 126.2, 115.1, 102.9, 56.4, 52.9, 51.4, 17.1.

LRMS: *m/z*(%)= 296.1 (100), 298.1 (32) [M + H]$^+$; HRMS: calcd. $C_{13}H_{15}O_3N_3{}^{35}Cl$ [M + H]$^+$: 296.0796; observed: 296.0798.

**Methyl (2-dimethylamino-6-methoxyquinazolin-4-yl)-*L*-alaninate (58)**

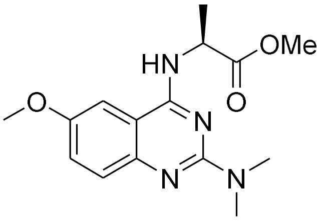

2 M $NHMe_2$ in THF (1.0 mL, 2.0 mmol) was added to a solution of methyl (2-chloro-6- methoxyquinazolin-4-yl)-*L*-alaninate (**57**) (200 mg, 0.68 mmol) in anhydrous DMF (3 mL). The reaction mixture was stirred for 4 h at 100 °C under an inert atmosphere and concentrated *in vacuo*. The product was purified using flash column chromatography (40−100% EtOAc in cyclohexane) to give methyl (2-dimethylamino-6-

methoxyquinazolin-4- yl)-*L*-alaninate (**58**) (183 mg, 0.60 mmol, 89%) as a white solid.

IR $\nu_{max}/cm^{-1}$(thin film): 1731 (C=O).

$^1$H NMR (400 MHz, $CDCl_3$) δ 7.40 (d, *J* = 9.1 Hz, 1H), 7.18 (dd, *J* = 9.1, 2.7 Hz, 1H), 6.85 (d, *J* = 2.8 Hz, 1H), 5.96 (d, *J* = 6.5 Hz, 1H), 4.83 (p, *J* = 7.0 Hz, 1H), 3.82 (s, 3H), 3.77 (s, 3H), 3.18 (s, 6H), 1.59 (d, *J* = 7.2 Hz, 3H).

$^{13}$C NMR (101 MHz, $CDCl_3$) δ 175.3, 159.0, 158.7, 154.3, 148.4, 127.7, 124.0, 109.8, 101.4, 56.1, 52.7, 50.3, 37.4, 18.4.

LRMS: *m/z*(%)= 305.1 (100) $[M + H]^+$; HRMS: calcd. $C_{15}H_{21}O_3N_4$ $[M + H]^+$: 305.1608; observed: 305.1607.

**(*S*)-2-(2-Dimethylamino-6-methoxyquinazolin-4-yl)amino-*N*-hydroxypropanamide (24)**

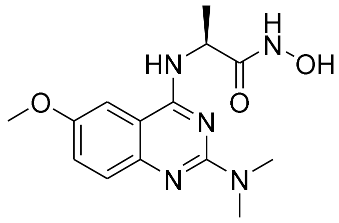


(*S*)-2-(2-Dimethylamino-6-methoxyquinazolin-4-yl)amino-*N*-hydroxypropanamide (**24**) was prepared using General Procedure D, with methyl (2-dimethylamino-6-methoxyquinazolin- 4-yl)-*L*-alaninate (**58**) (100 mg, 0.33 mmol). Purification using RP HPLC (MeCN in $H_2O$ + 0.1% (v/v) TFA) and lyophilization gave (*S*)-2-(2-dimethylamino-6-methoxyquinazolin-4-yl)amino- *N*-hydroxypropanamide (**24**) (33 mg, 0.11 mmol, 33%) as a white solid.

IR $\nu_{max}/cm^{-1}$(solid): 3234 (O-H) and 1644 (C=O).

$^1$H NMR (400 MHz, MeOD) δ 7.37 (s, 1H), 7.29 (d, *J* = 9.0 Hz, 1H), 7.21 (d, *J* = 8.3 Hz, 1H), 4.68 (q, *J* = 6.8 Hz, 1H), 3.77 (s, 3H), 3.30 (s, 6H), 1.69 (d, *J* = 7.2 Hz, 3H).

$^{13}$C NMR (101 MHz, MeOD) δ 172.2, 160.3, 158.0, 152.8, 134.7, 126.4, 119.9, 110.8, 104.6, 56.9, 51.4, 38.3, 18.1.

LRMS: *m/z*(%)= 306.2 (100) $[M + H]^+$; HRMS: calcd. $C_{14}H_{20}O_3N_5$ $[M + H]^+$: 306.1561; observed: 306.1561.

$[a]\frac{25}{D}$ = +103.2 (c = 1.2 in MeOH).

**NMR Spectra**

**2-Benzyloxy-6-bromo-benzo[de]isoquinoline-1,3-dione (25)**

$^1$H NMR

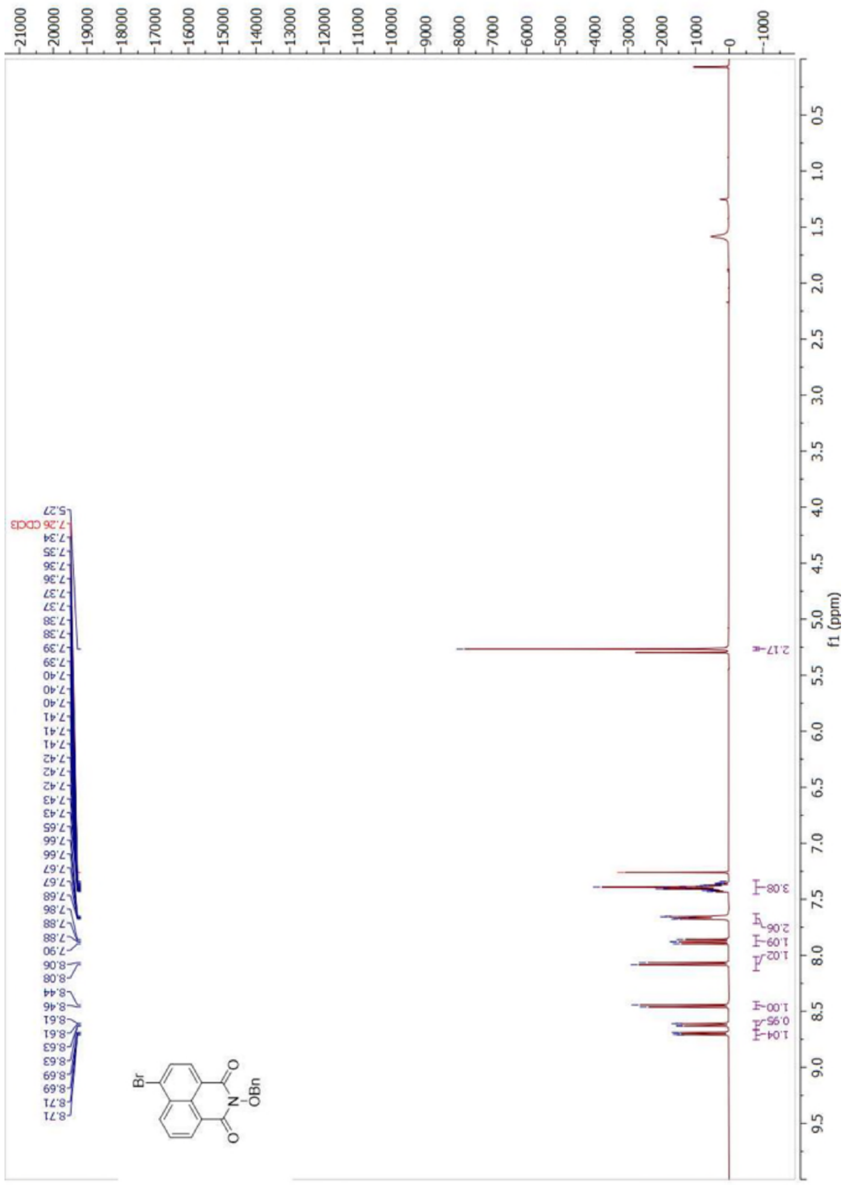

$^{13}C$ NMR

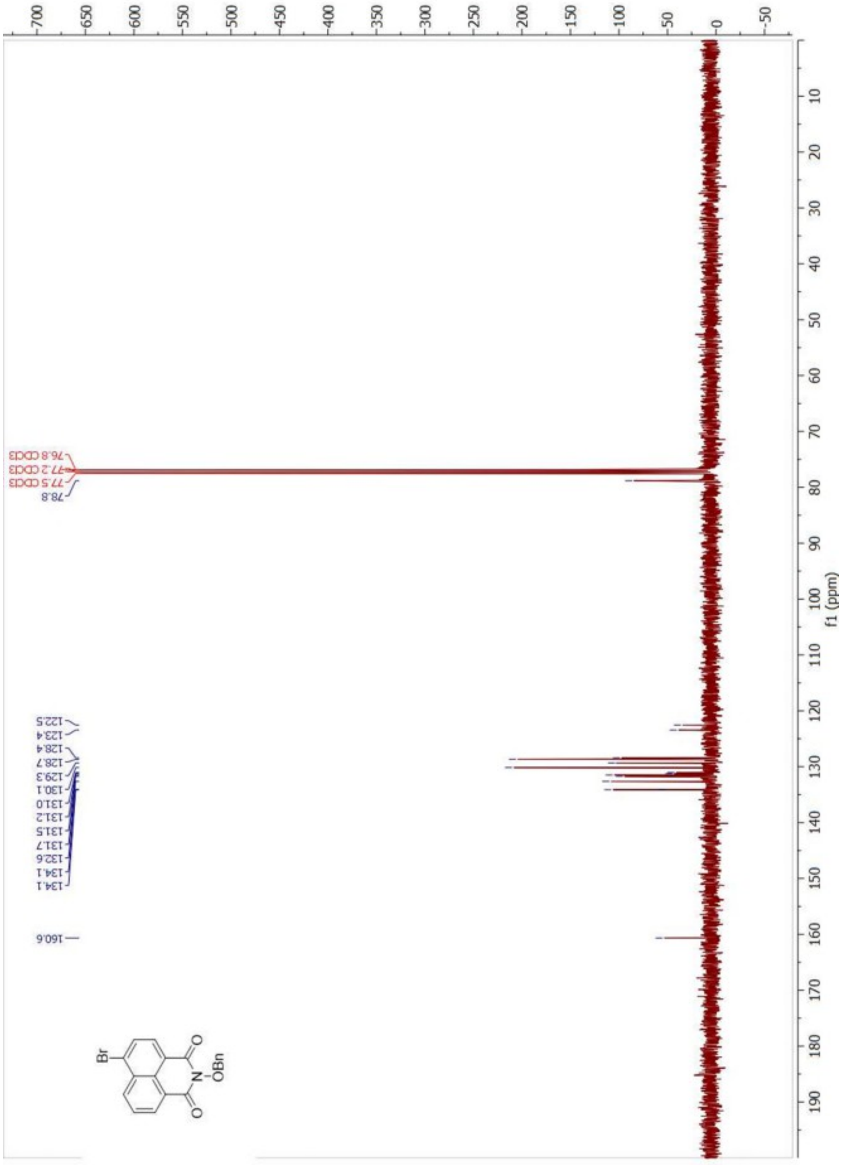

76.8 CDCl3
77.2 CDCl3
77.5 CDCl3
78.8
122.5
123.4
128.4
128.7
129.3
130.1
131.0
131.2
131.5
131.7
132.6
134.1
134.1
160.6
Br
N
O
OBn
f1 (ppm)

**2-Benzyloxy-6-methoxy-benzo[de]isoquinoline-1,3-dione (26)**

$^1$H NMR

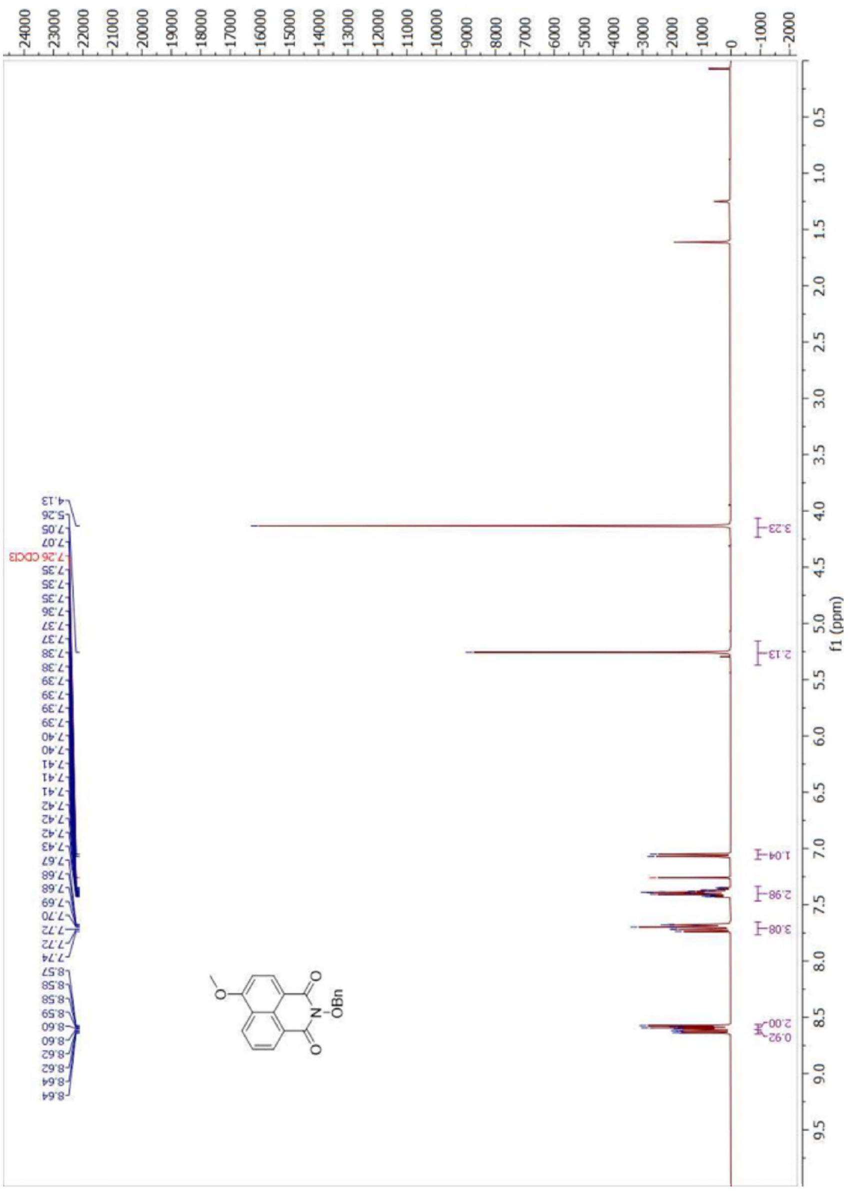

$^{13}C$ NMR

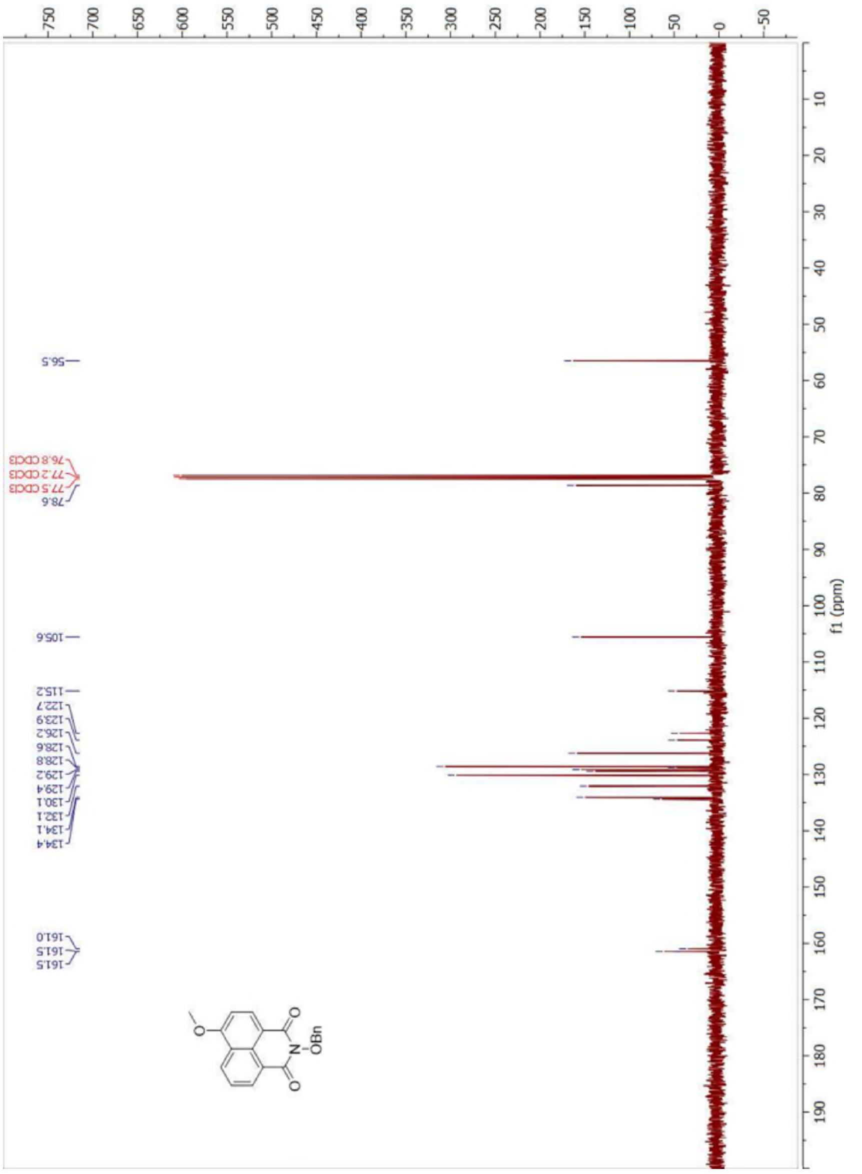

56.5
76.8 CDCl3
77.2 CDCl3
77.5 CDCl3
78.6
105.6
115.2
122.7
123.9
126.2
128.6
128.8
129.2
129.4
130.1
132.1
134.1
134.4
161.0
161.5
161.5
f1 (ppm)
OBn

**2-Hydroxy-6-methoxy-benzo[de]isoquinoline-1,3-dione (1)**

$^1$H NMR

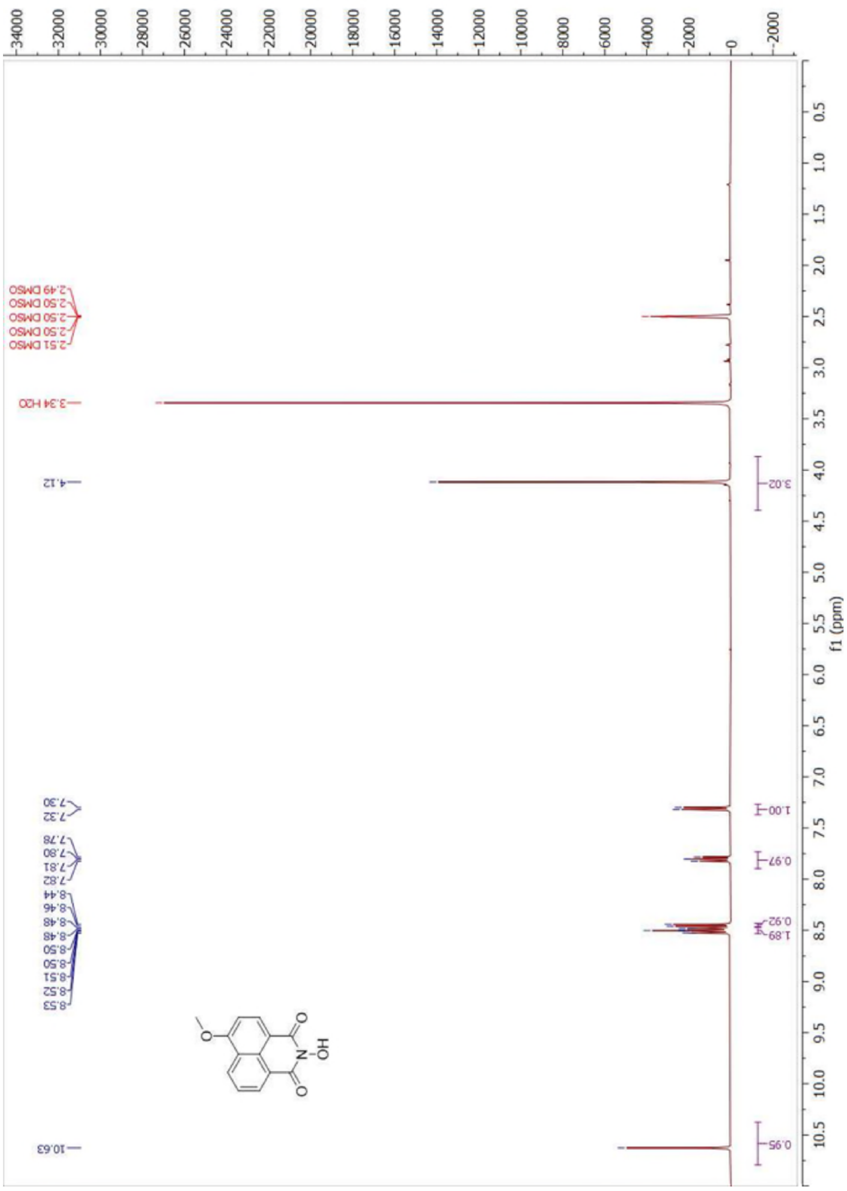

$^{13}C$ NMR

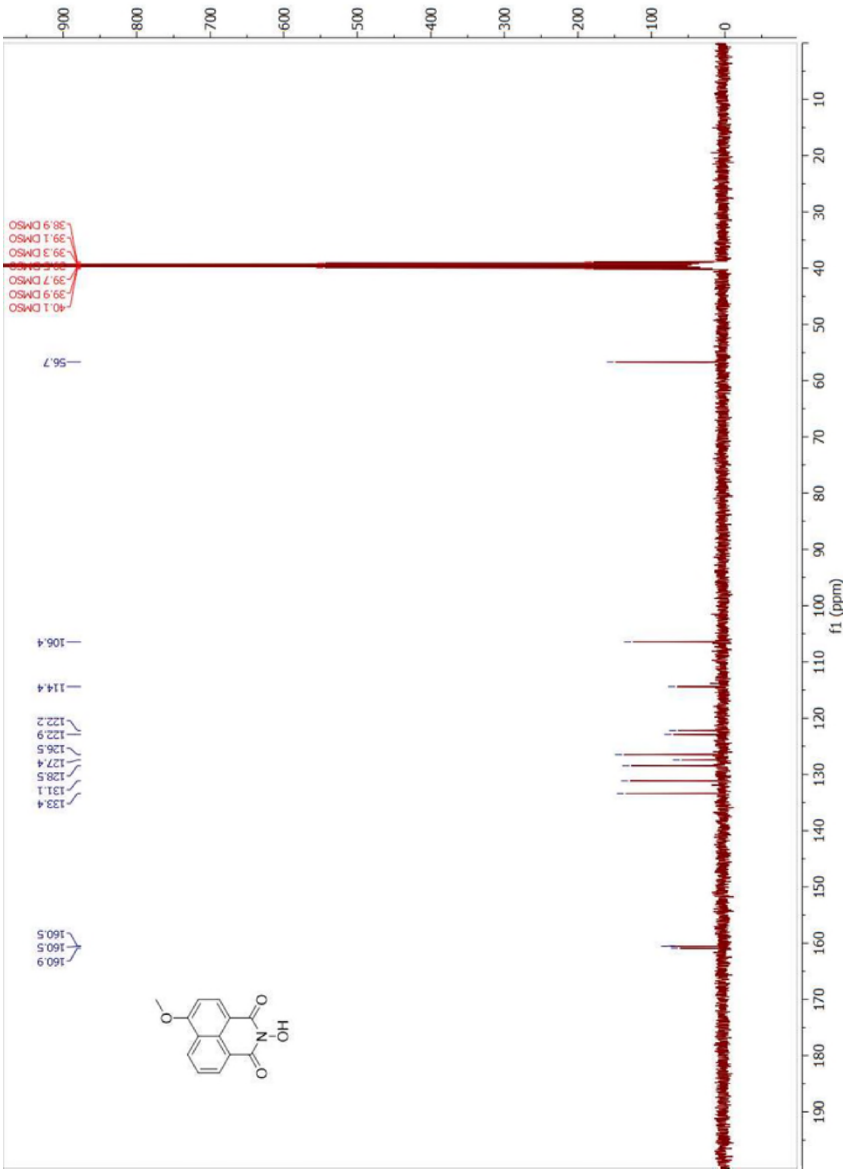

38.9 DMSO
39.1 DMSO
39.3 DMSO
39.7 DMSO
39.9 DMSO
40.1 DMSO
56.7
106.4
114.4
122.2
122.9
126.5
127.4
128.5
131.1
133.4
160.5
160.5
160.9
f1 (ppm)

**4,6-Dichloro-3-nitroquinoline (27)**

$^1$H NMR

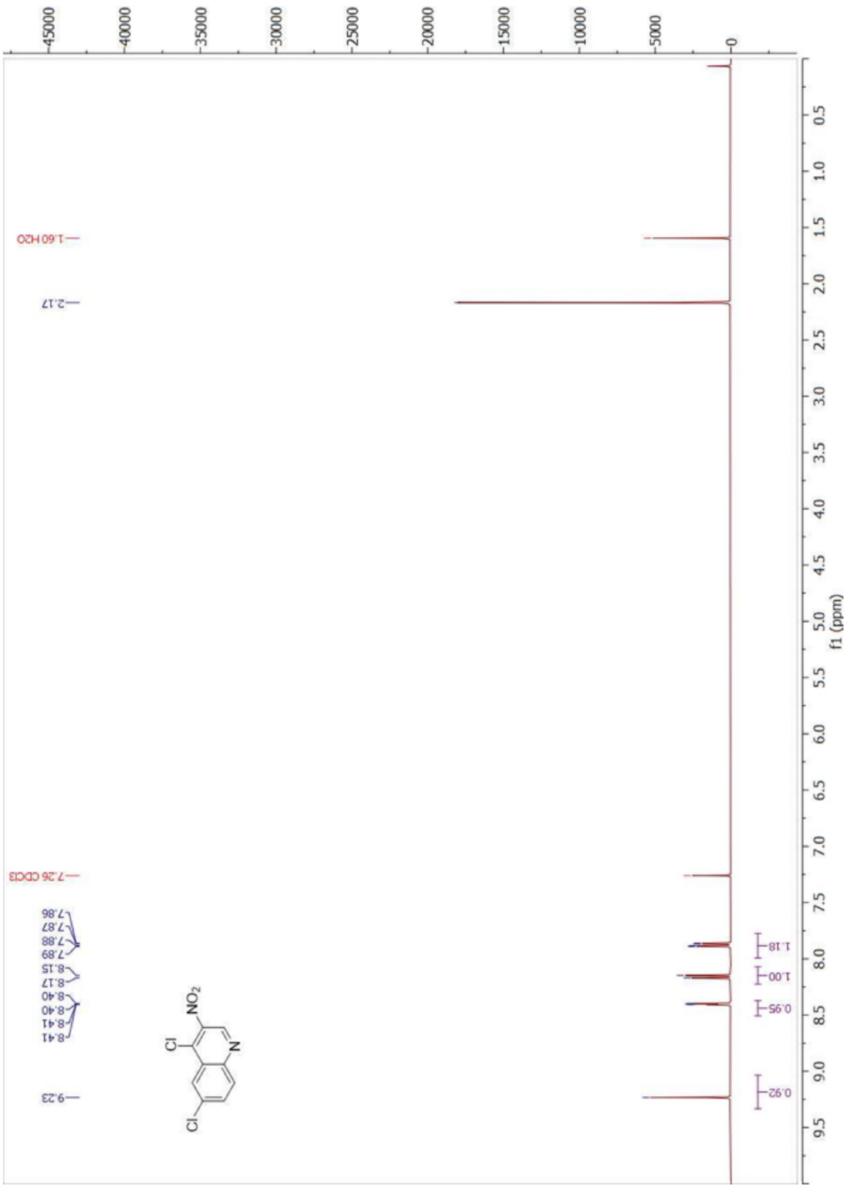

$^{13}C$ NMR

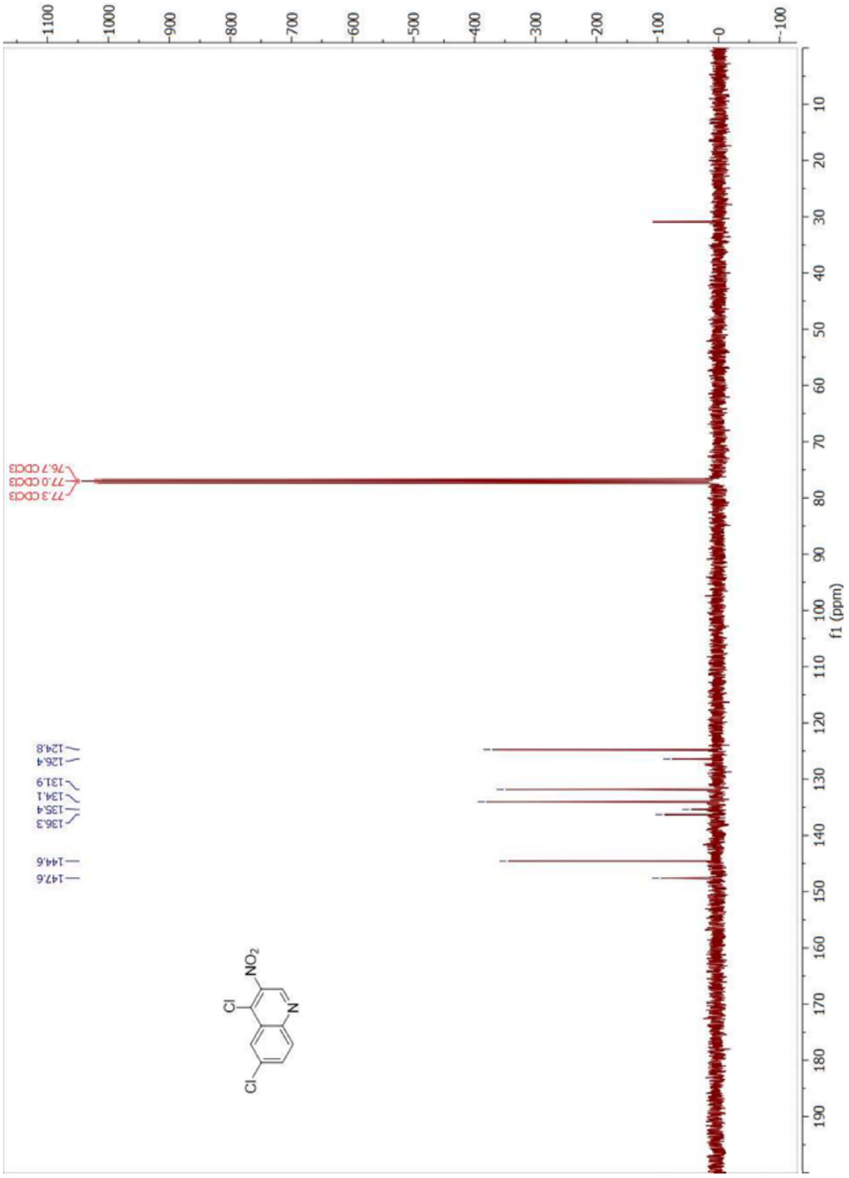
76.7 CDCl3
77.0 CDCl3
77.3 CDCl3
124.8
126.4
131.9
134.1
135.4
136.3
144.6
147.6
f1 (ppm)
NO2
Cl
Cl
N

**4- Azido-6-chloro-3-nitroquinoline (28)**

$^{1}$H NMR

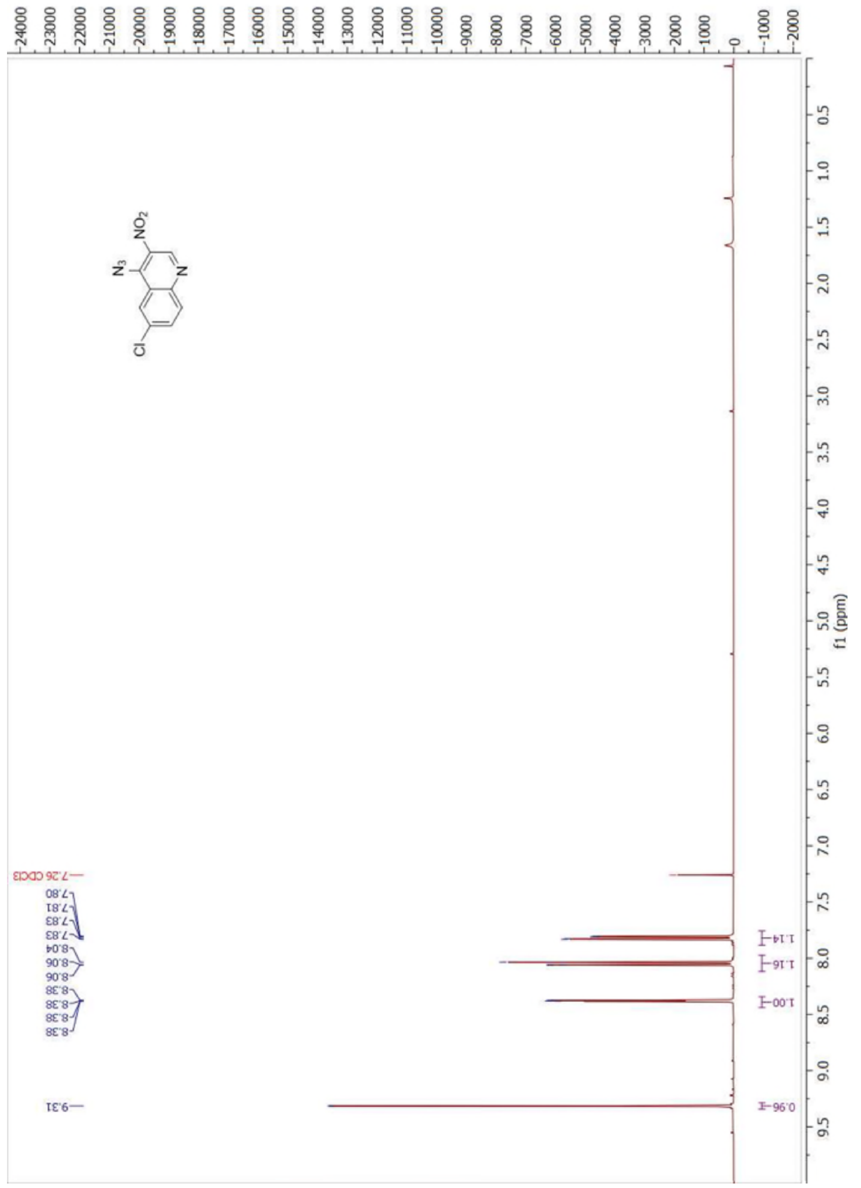

$^{13}C$ NMR

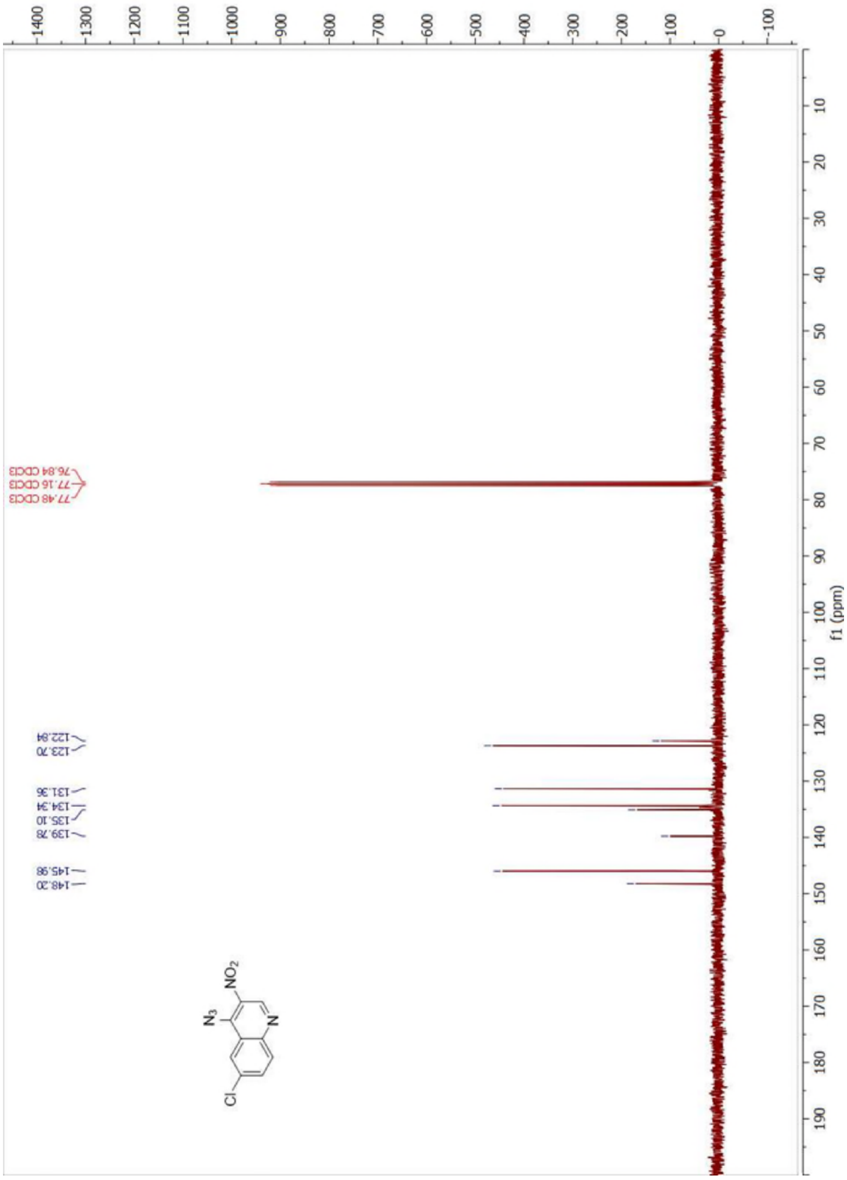

76.84 CDCl3
77.16 CDCl3
77.48 CDCl3
122.84
123.70
131.36
134.34
135.10
139.78
145.98
148.30
N3
NO2
N
Cl
f1 (ppm)

**9-Chloro-1,4-dihydropyrazino[2,3-c]quinoline-2,3-dione (2)**

$^{1}$H NMR

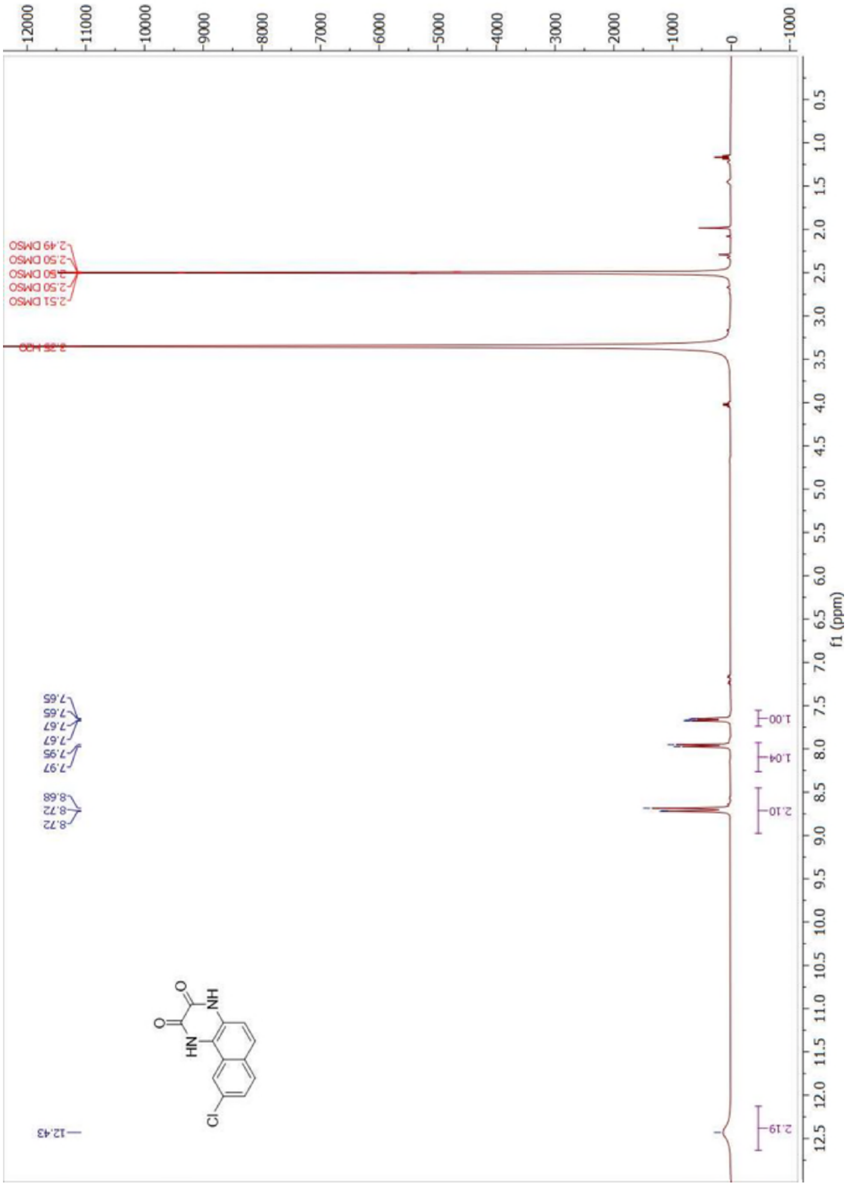

$^{13}C$ NMR

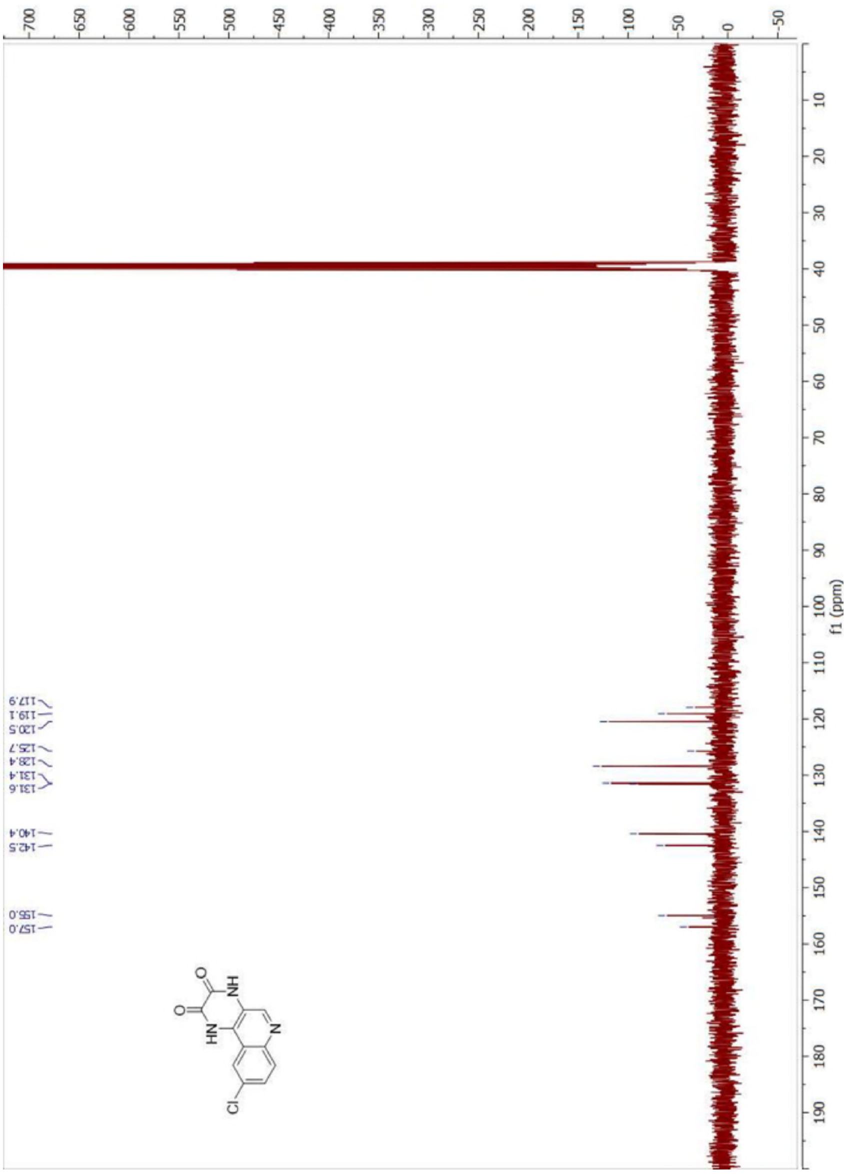

**5- Methoxy-benzo[de]isochromene-1,3-dione (29)**

$^1$H NMR

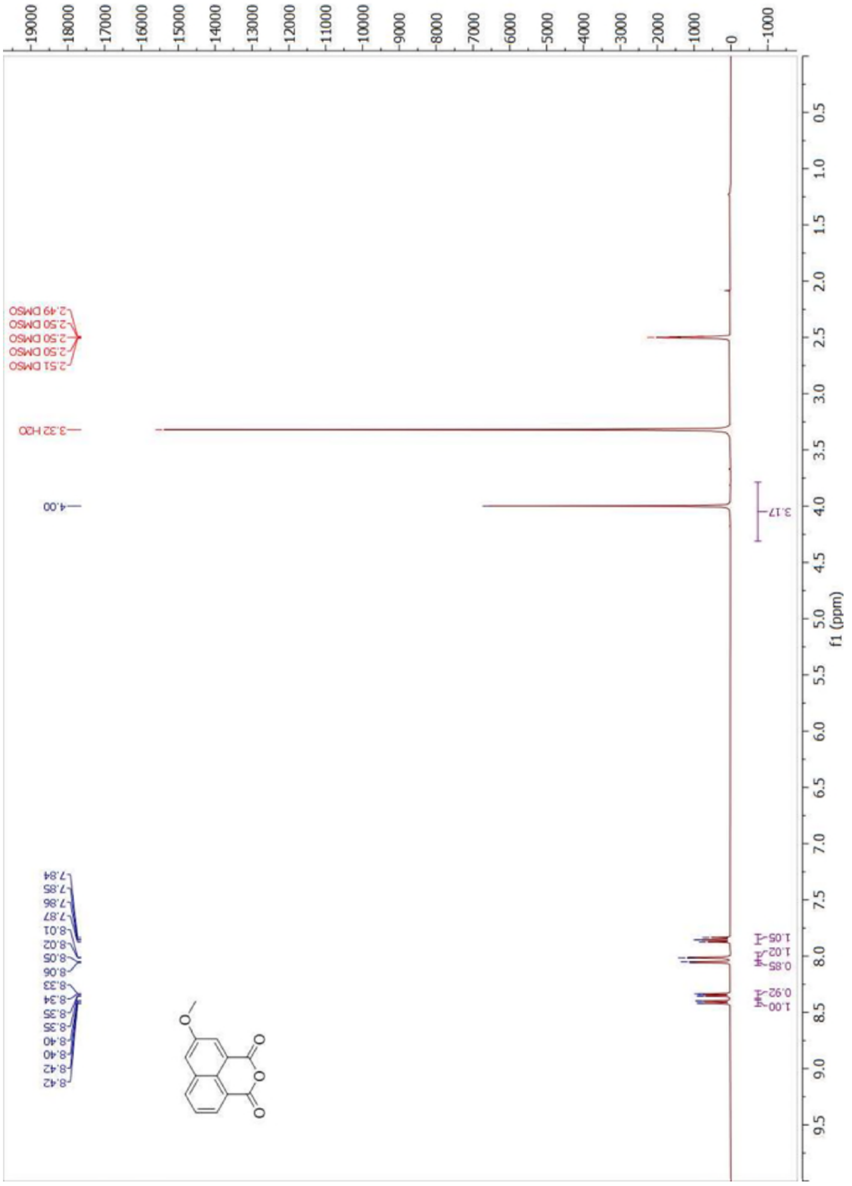

$^{13}C$ NMR

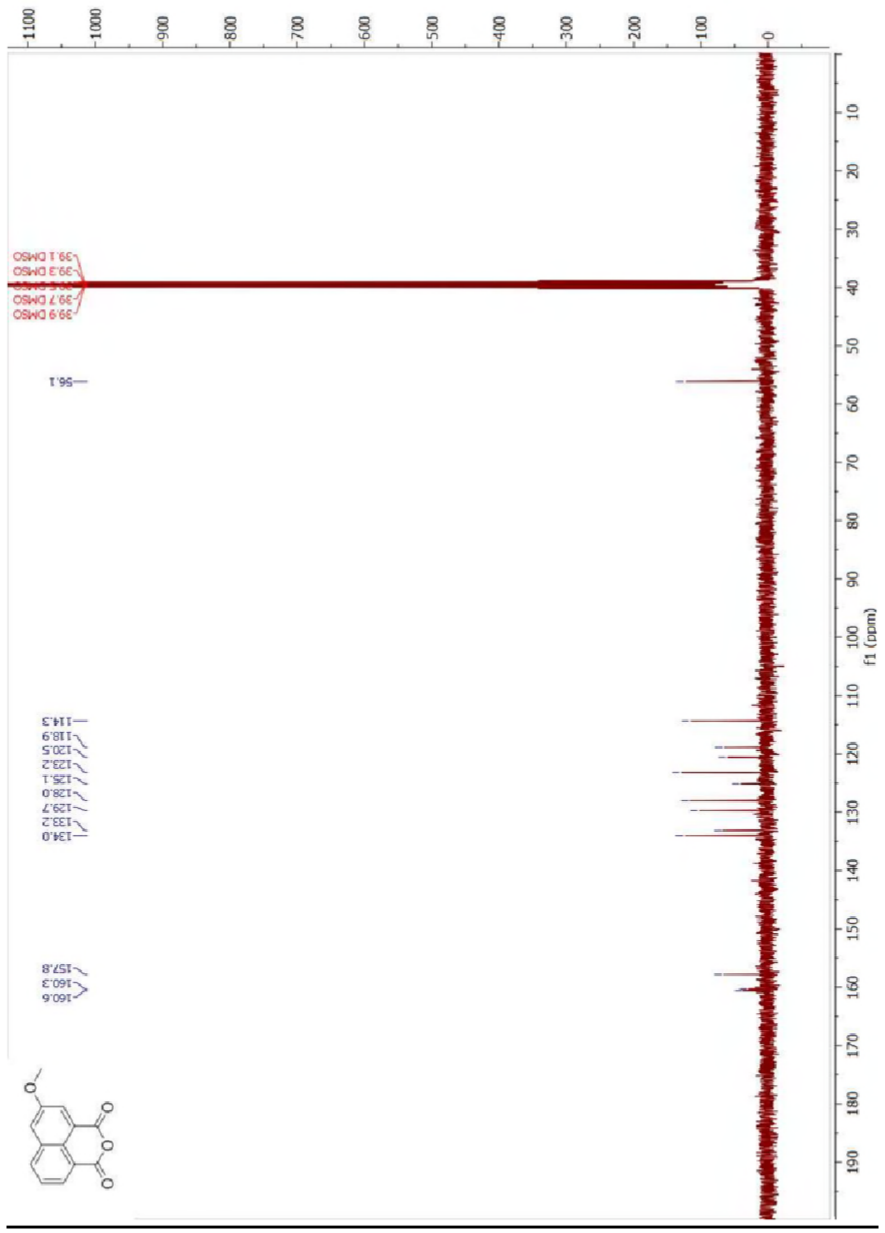
39.1 DMSO
39.3 DMSO
39.7 DMSO
39.9 DMSO
56.1
114.3
118.9
120.5
123.2
125.1
128.0
129.7
133.2
134.0
157.8
160.3
160.6
f1 (ppm)

**2-Hydroxy-5-methoxy-benzo[de]isoquinoline-1,3-dione (3)**

$^{1}H$ NMR

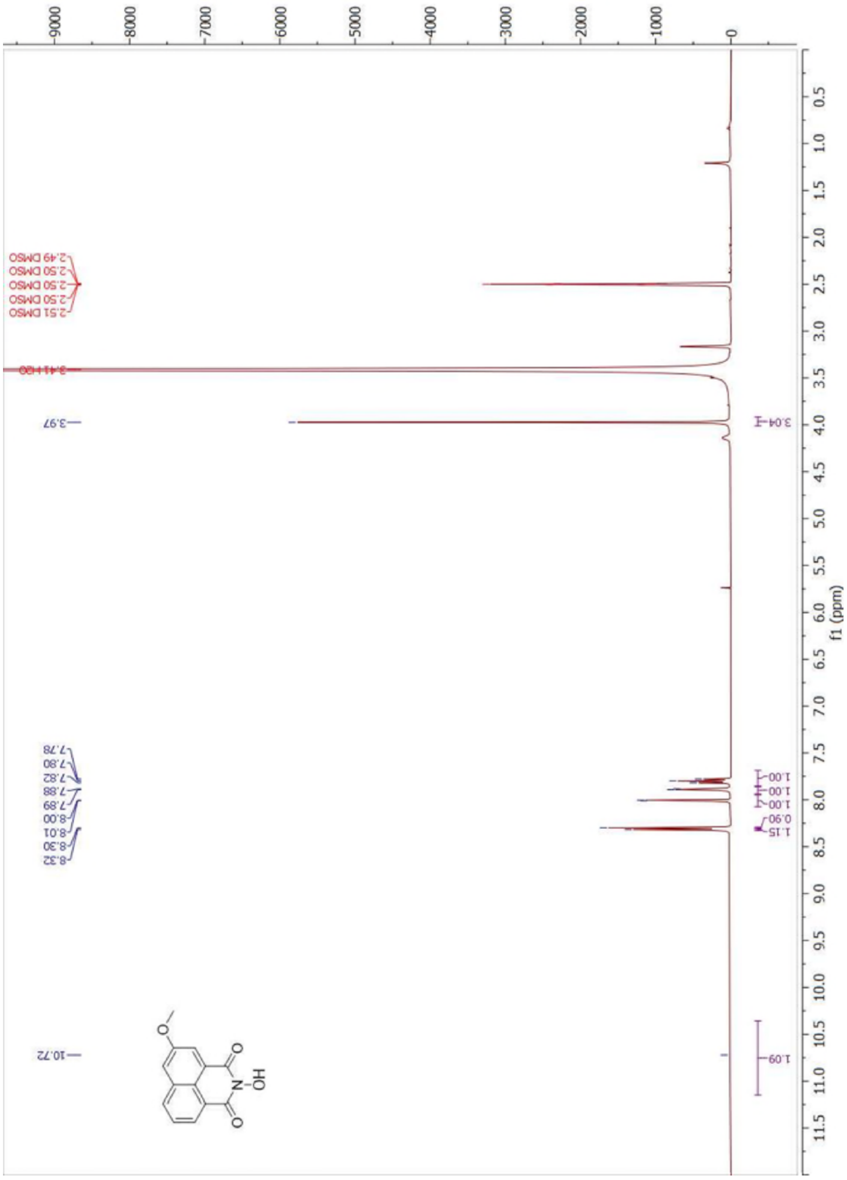

$^{13}C$ NMR

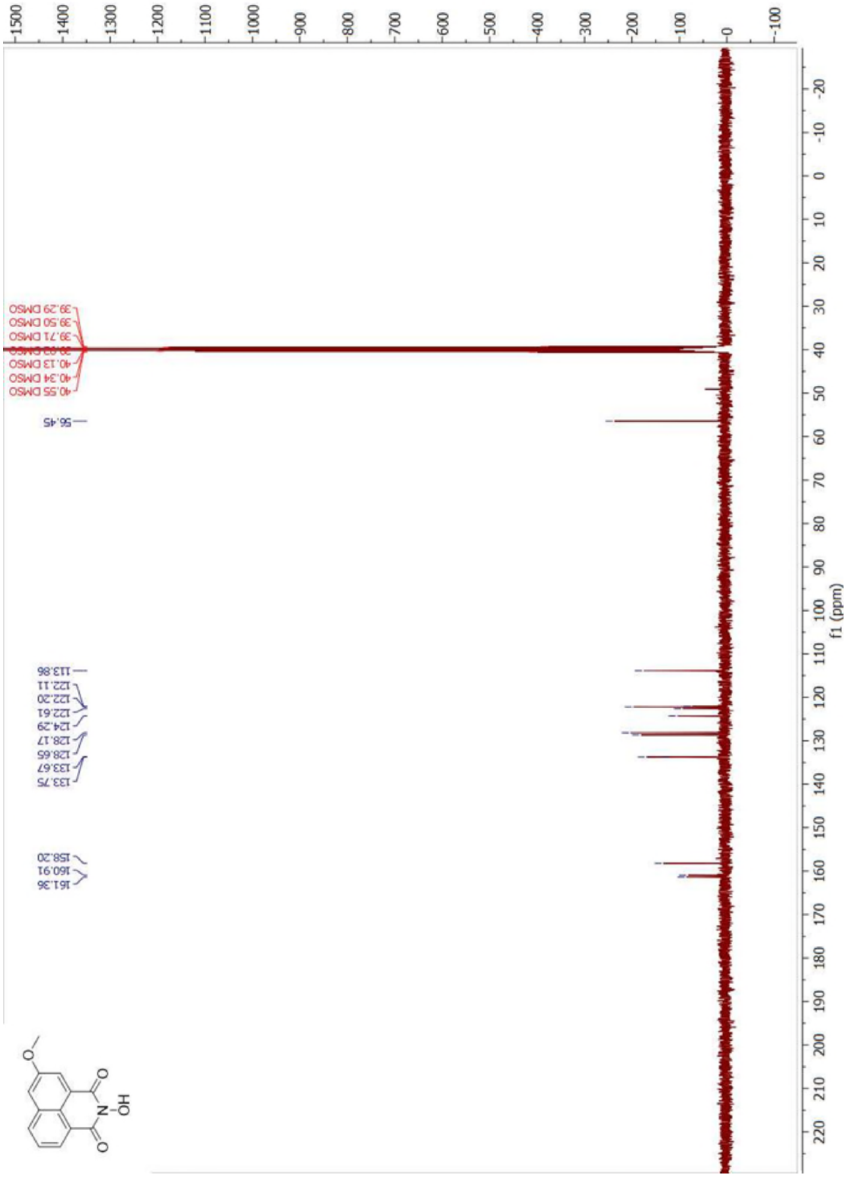
39.29 DMSO
39.50 DMSO
39.71 DMSO
40.13 DMSO
40.34 DMSO
40.55 DMSO
56.45
113.86
122.11
122.20
122.61
124.29
128.17
128.65
133.67
133.75
158.20
160.91
161.36
f1 (ppm)

**6- Chloroquinazoline-2,4-dione (30)**

$^{1}H$ NMR

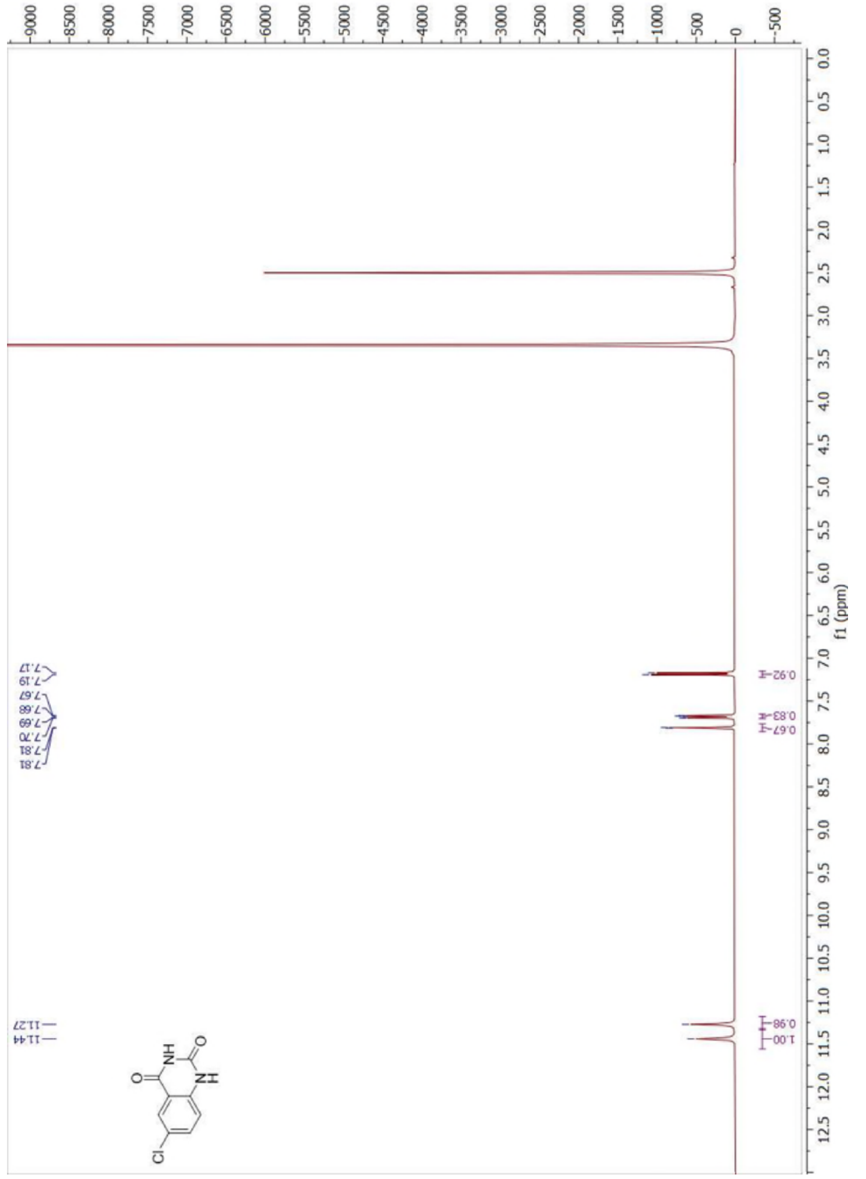

$^{13}C$ NMR

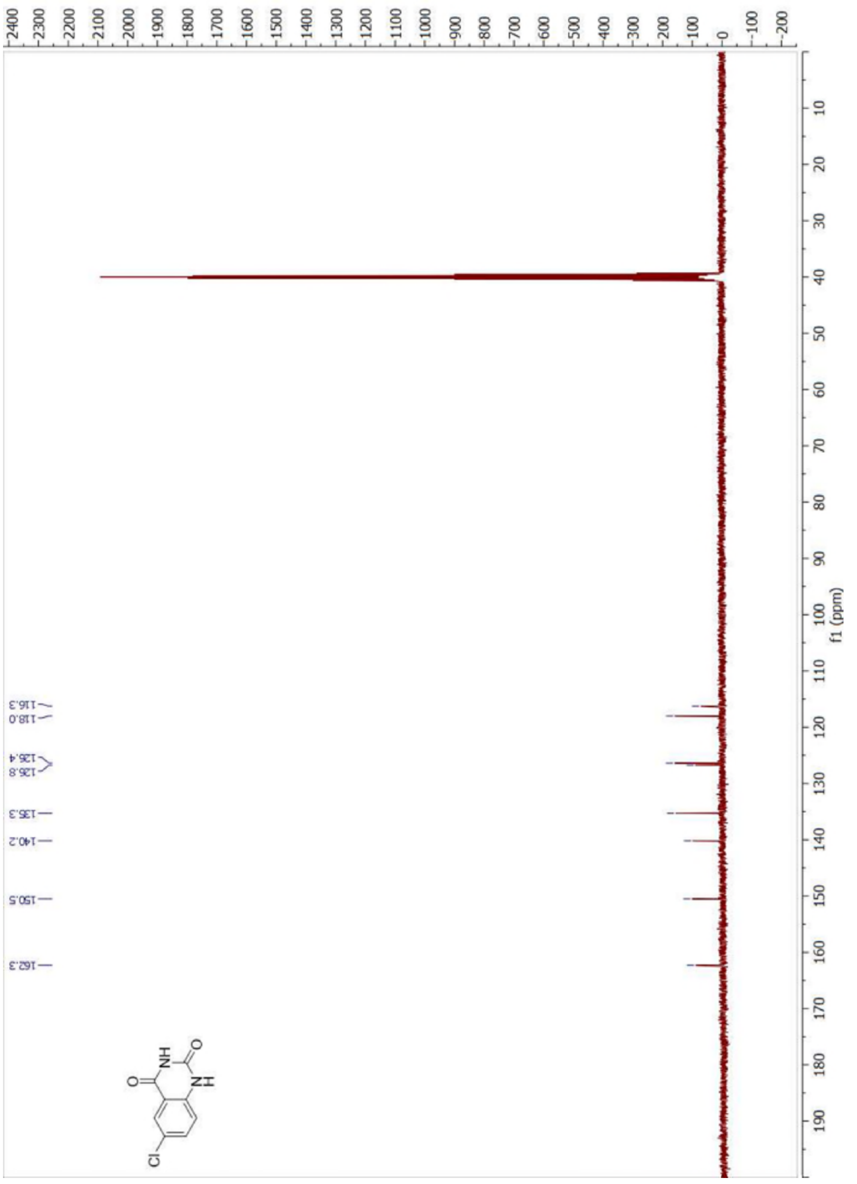

**2,4,6-Trichloroquinazoline (31)**

$^1H$ NMR

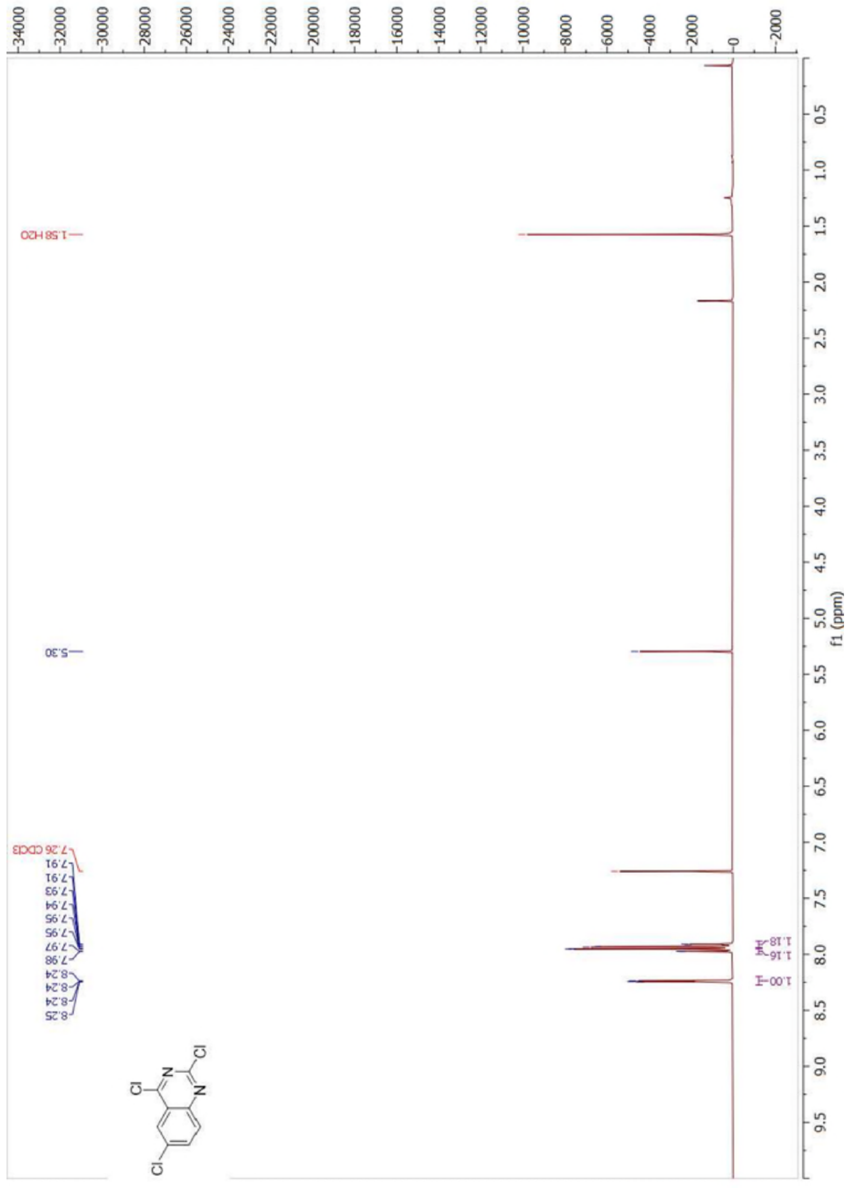

$^{13}C$ NMR

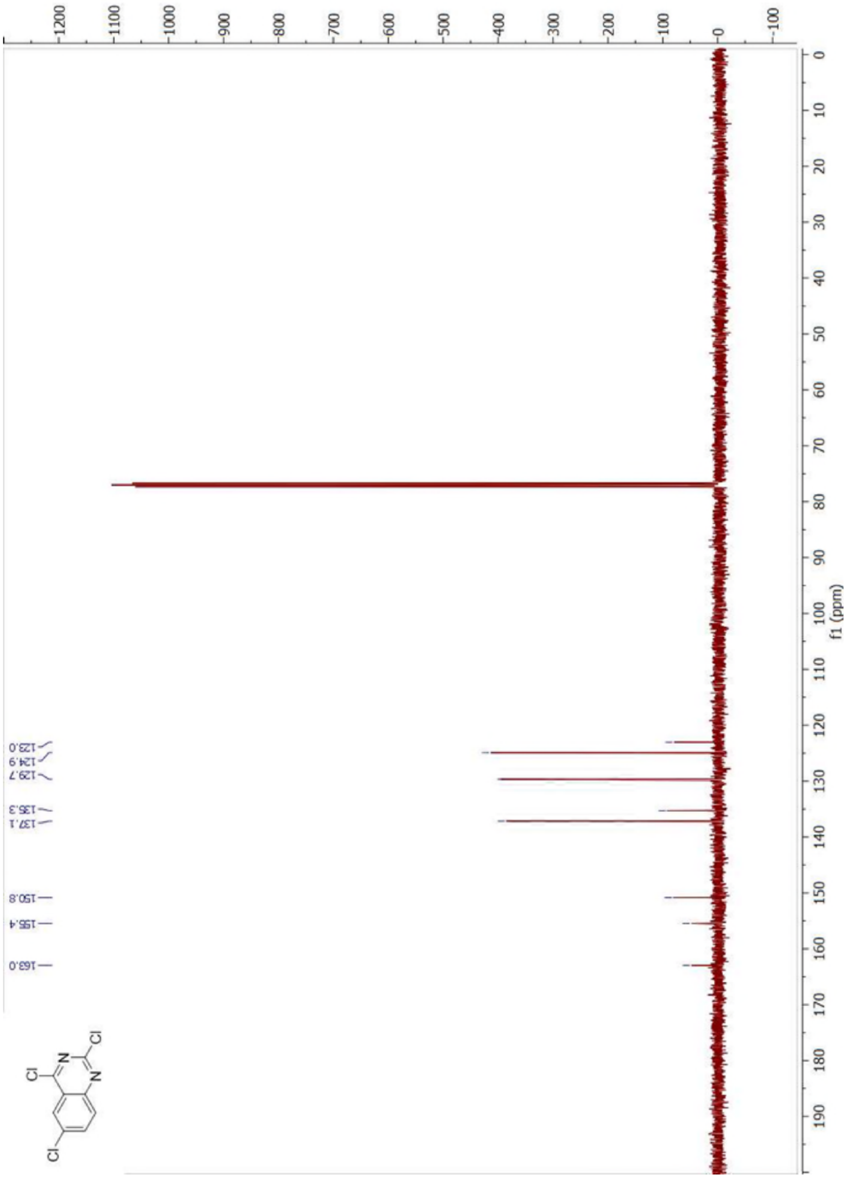

**Methyl (2,6-dichloroquinazolin-4-yl)-*L*-leucinate (32)**

$^1$H NMR

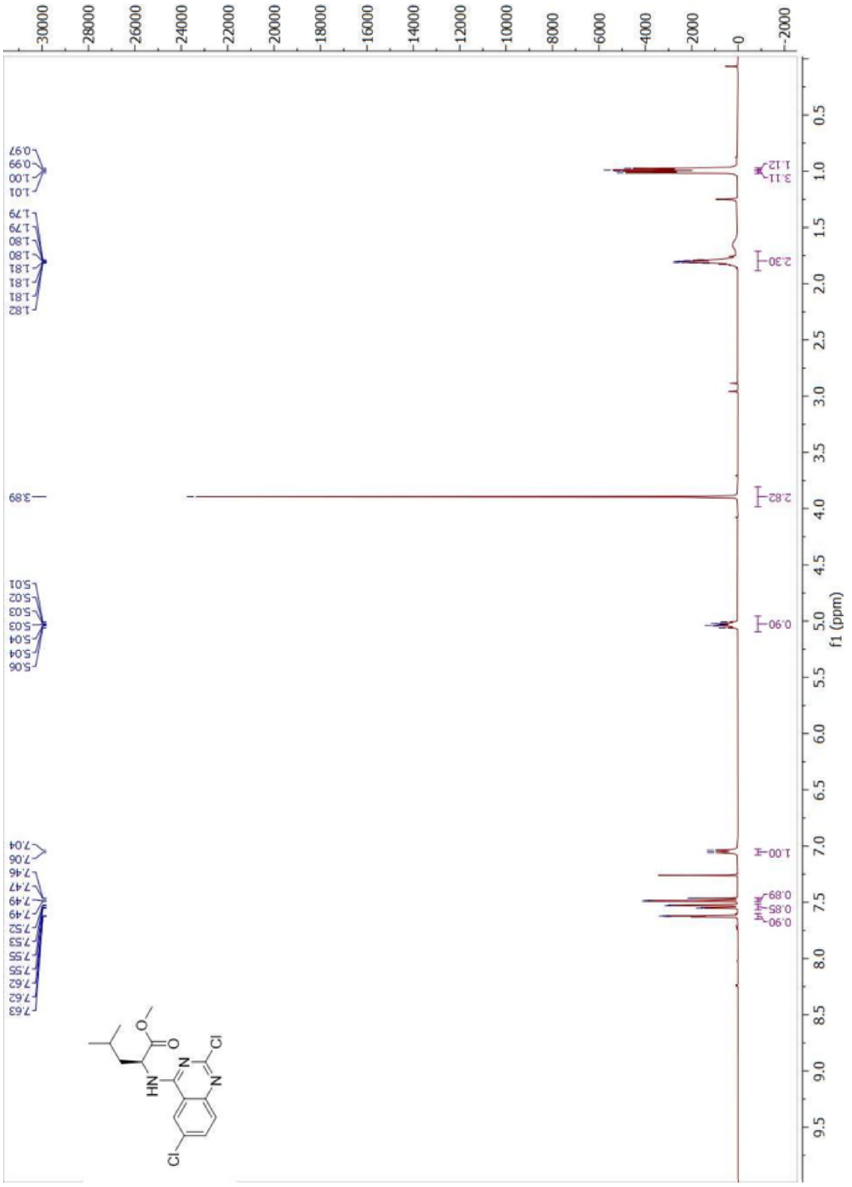

$^{13}C$ NMR

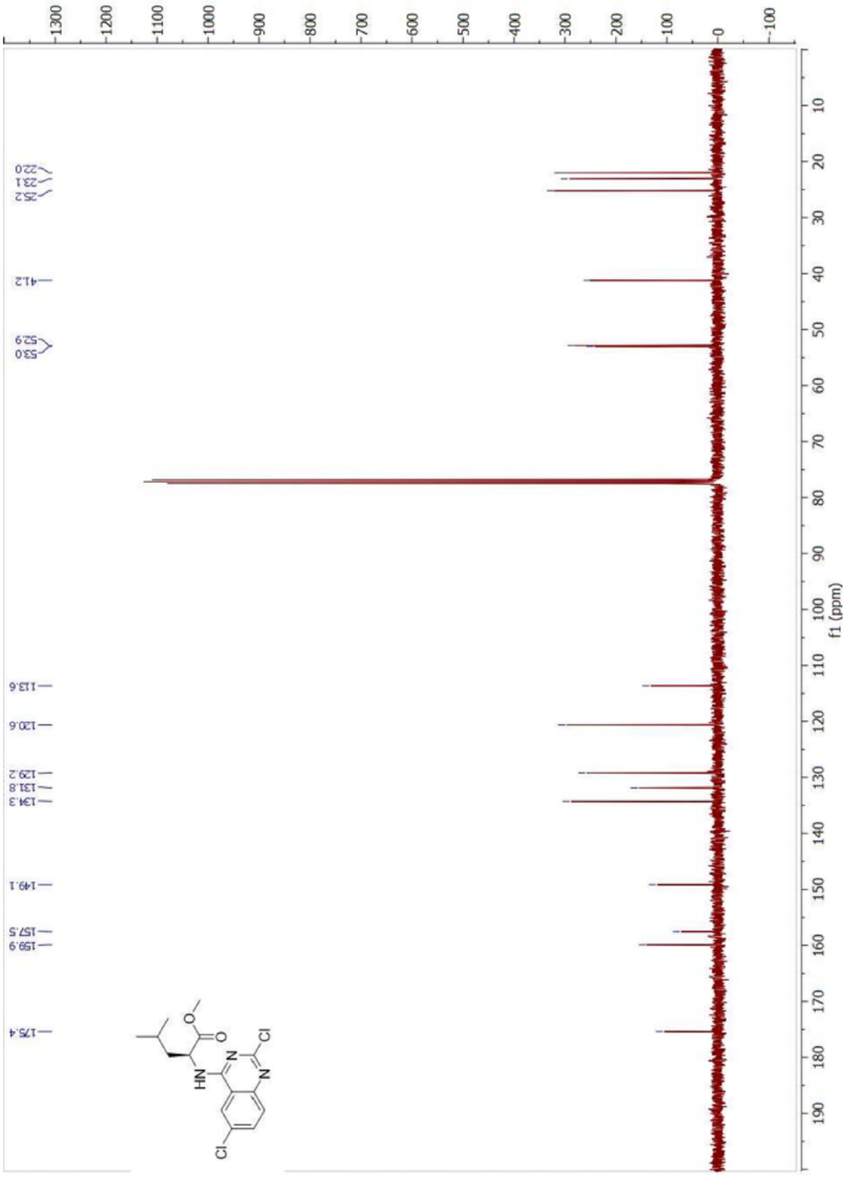
22.0
23.1
25.2
41.2
52.9
53.0
113.6
120.6
129.2
131.8
134.3
149.1
157.5
159.9
175.4
f1 (ppm)
HN
N
Cl
O

**Methyl (2-allylamino-6-chloroquinazolin-4-yl)-*L*-leucinate (7)**

$^1$H NMR

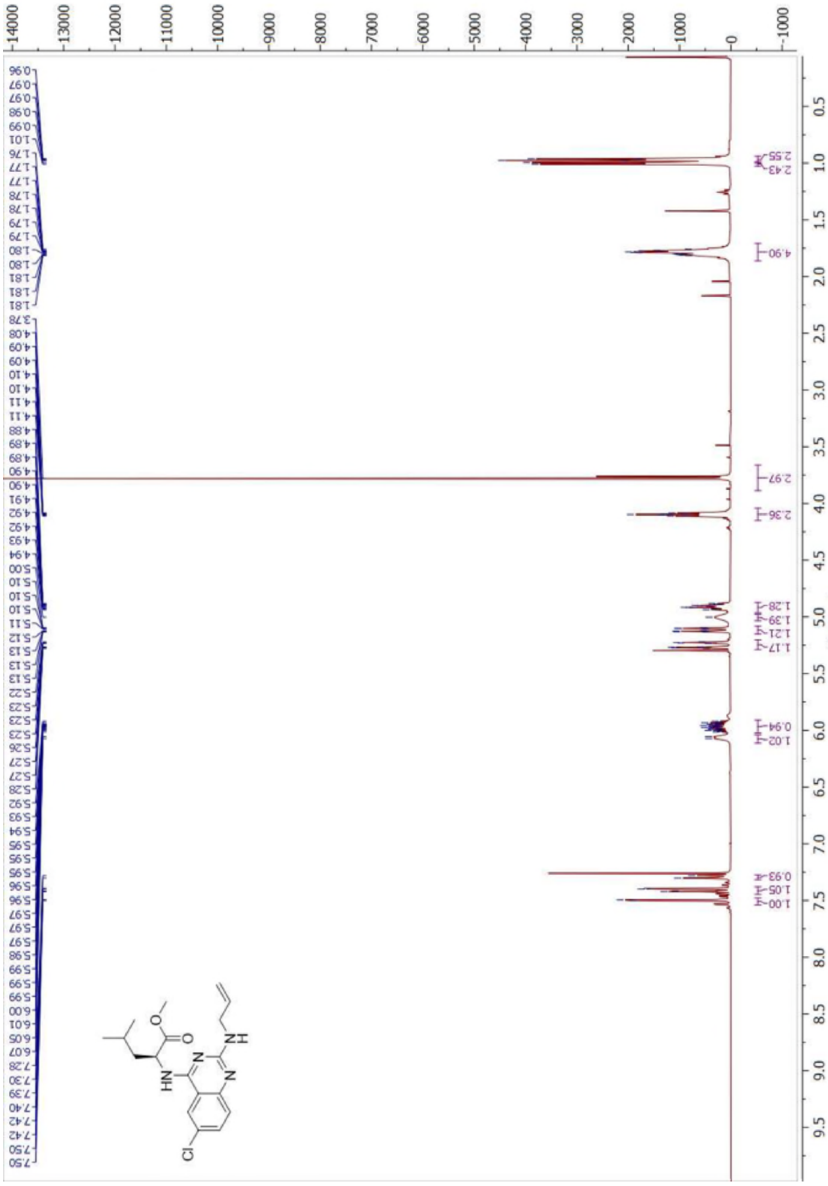

$^{13}C$ NMR

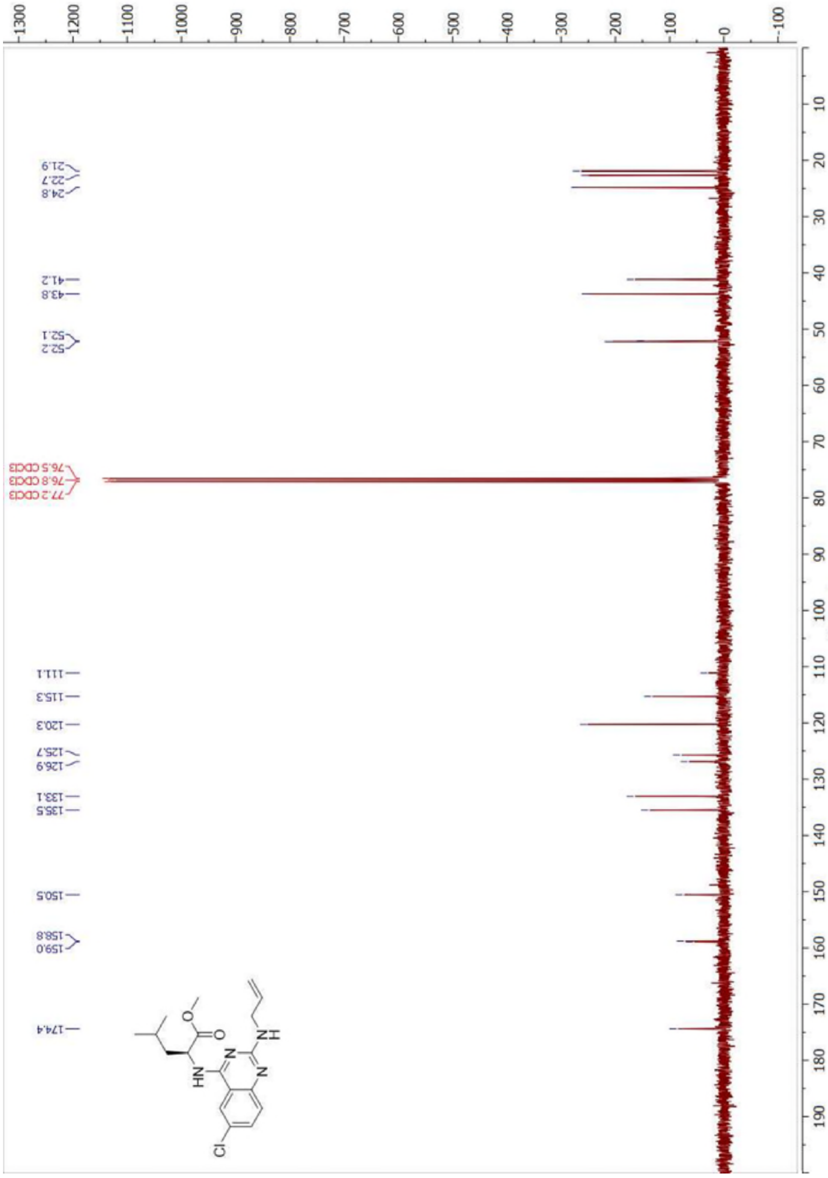

**(2-Allylamino-6-chloroquinazolin-4-yl)-*L*-leucine (6)**

$^{1}$H NMR

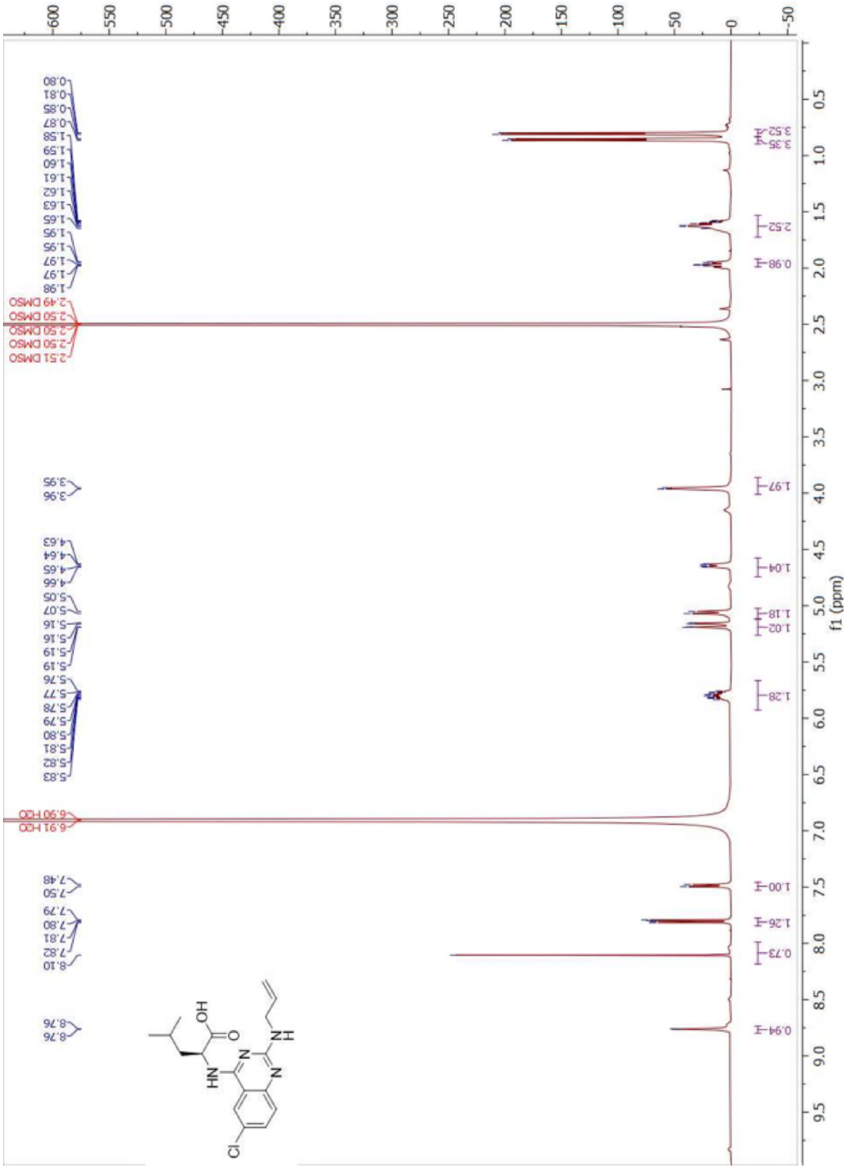

$^{13}C$ NMR

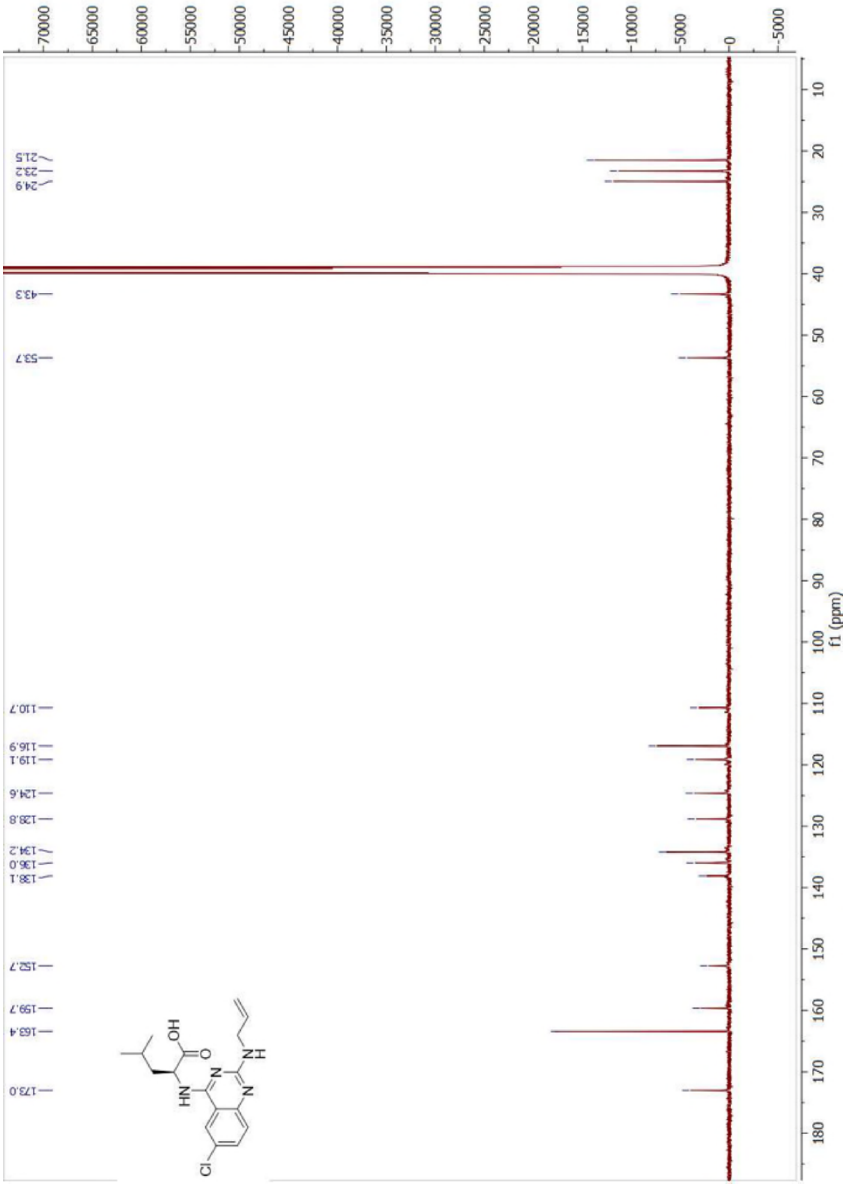

21.5
23.2
24.9
43.3
53.7
110.7
116.9
119.1
124.6
128.8
134.2
136.0
138.1
152.7
159.7
163.4
173.0
f1 (ppm)

**(*S*)-2-(2,6-Dichloroquinazolin-4-yl)amino-4-methylpentanamide (33)**

$^{1}$H NMR

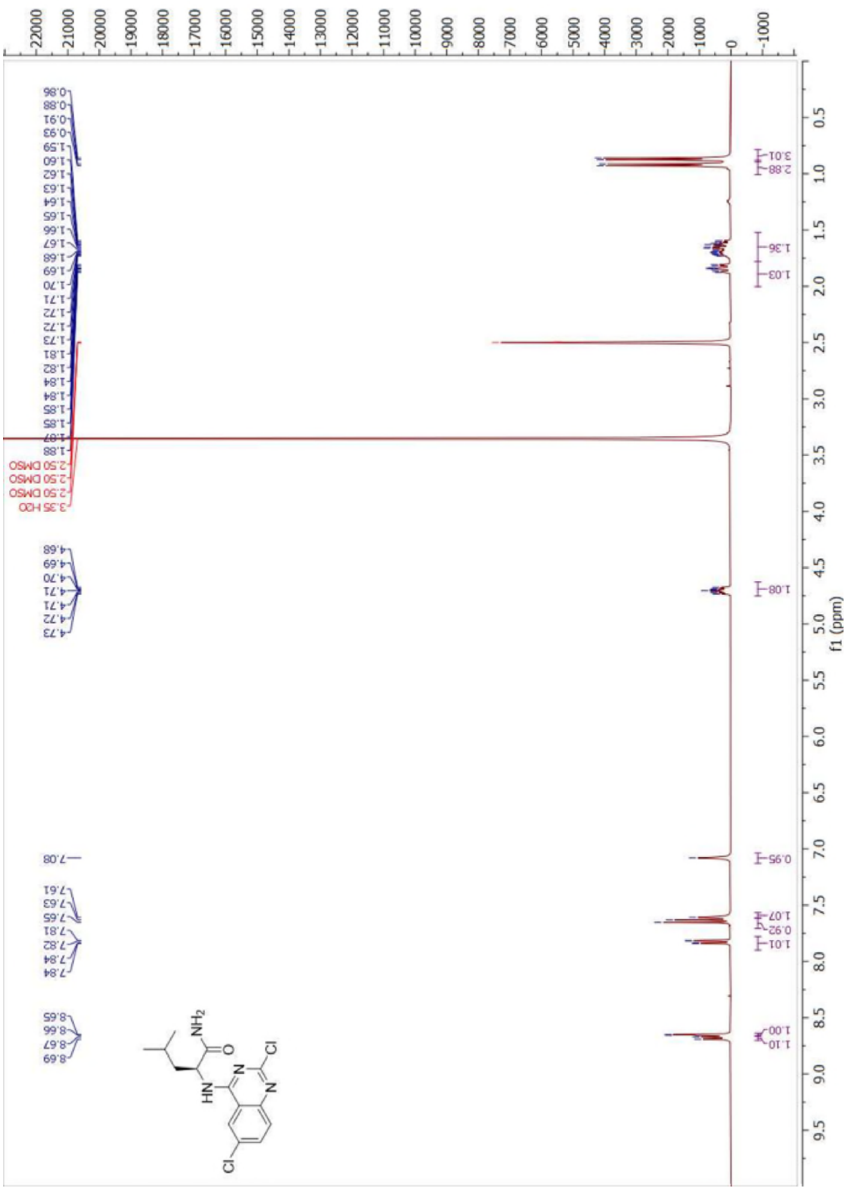

$^{13}C$ NMR

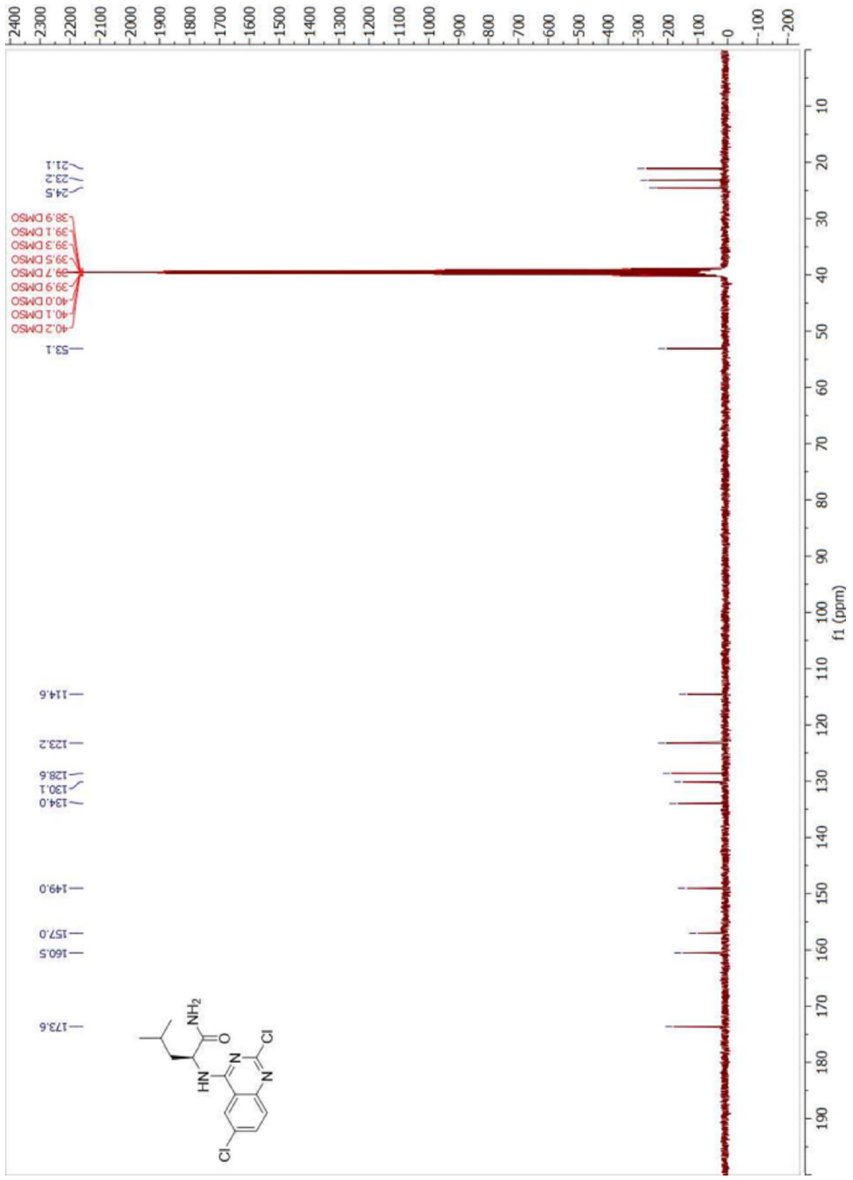

21.1
23.2
24.5
38.9 DMSO
39.1 DMSO
39.3 DMSO
39.5 DMSO
39.7 DMSO
39.9 DMSO
40.0 DMSO
40.1 DMSO
40.2 DMSO
53.1
114.6
123.2
128.6
130.1
134.0
149.0
157.0
160.5
173.6
f1 (ppm)
NH2
O
HN
N
Cl
Cl

**(*S*)-2-(2-Allylamino-6-chloroquinazolin-4-yl)amino-4-methylpentanamide (8)**

$^{1}$H NMR

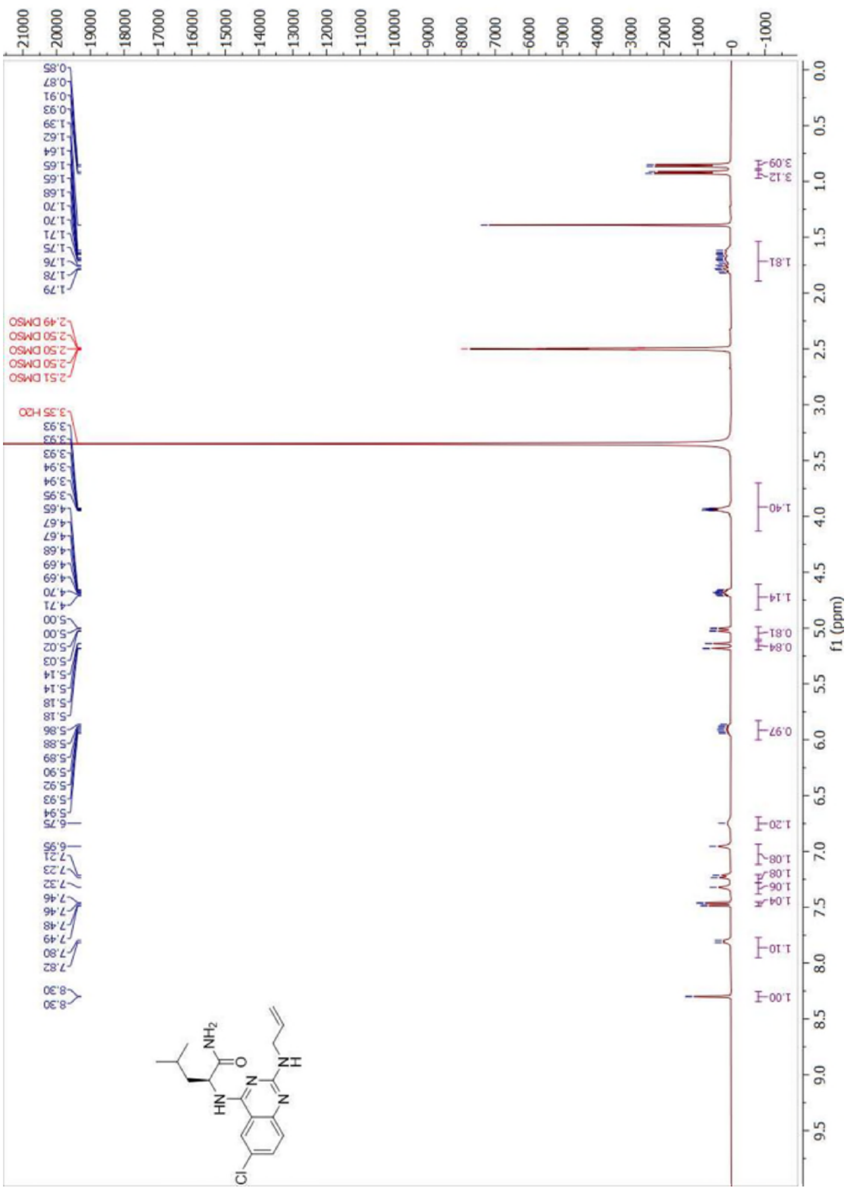

$^{13}C$ NMR

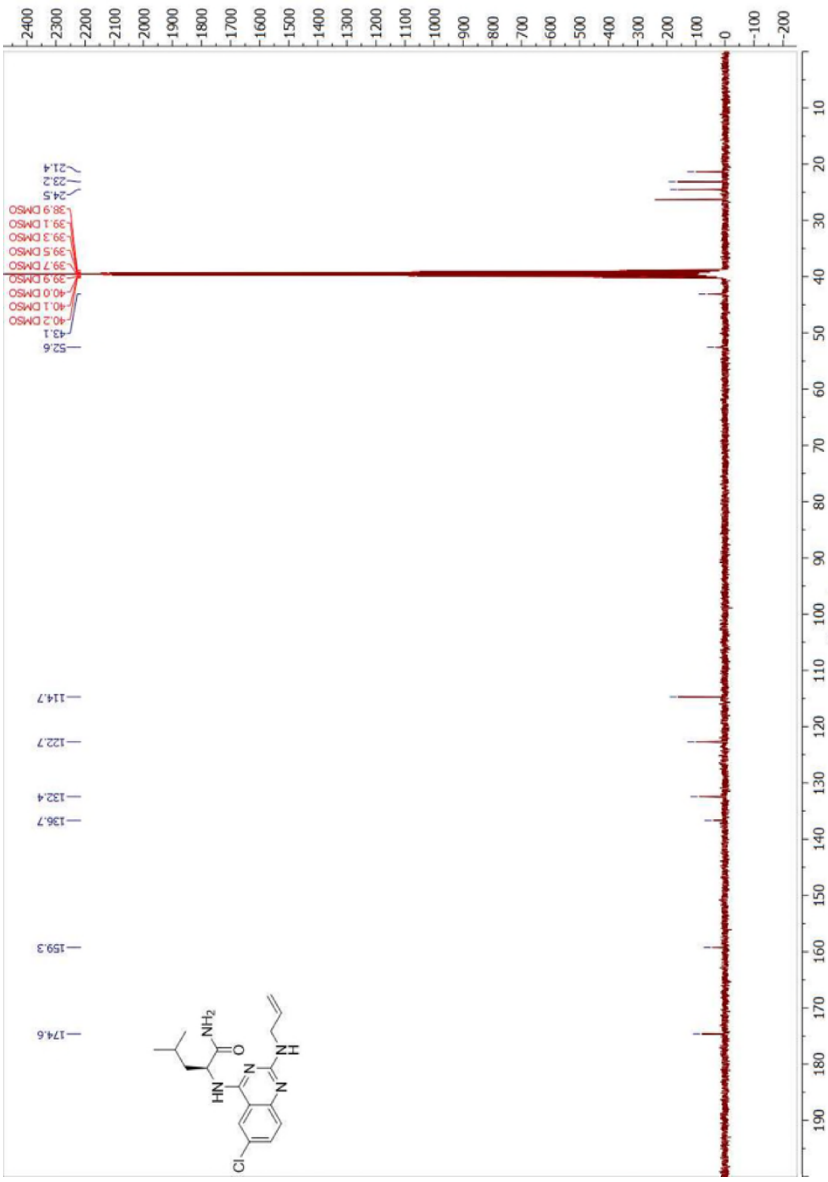

21.4
23.2
24.5
38.9 DMSO
39.1 DMSO
39.3 DMSO
39.5 DMSO
39.7 DMSO
39.9 DMSO
40.0 DMSO
40.1 DMSO
40.2 DMSO
43.1
52.6
114.7
122.7
132.4
136.7
159.3
174.6
f1 (ppm)

**(*S*)-2-(2,6-Dichloroquinazolin-4-yl)amino-4-methylpentan-1-ol (34)**

$^{1}$H NMR

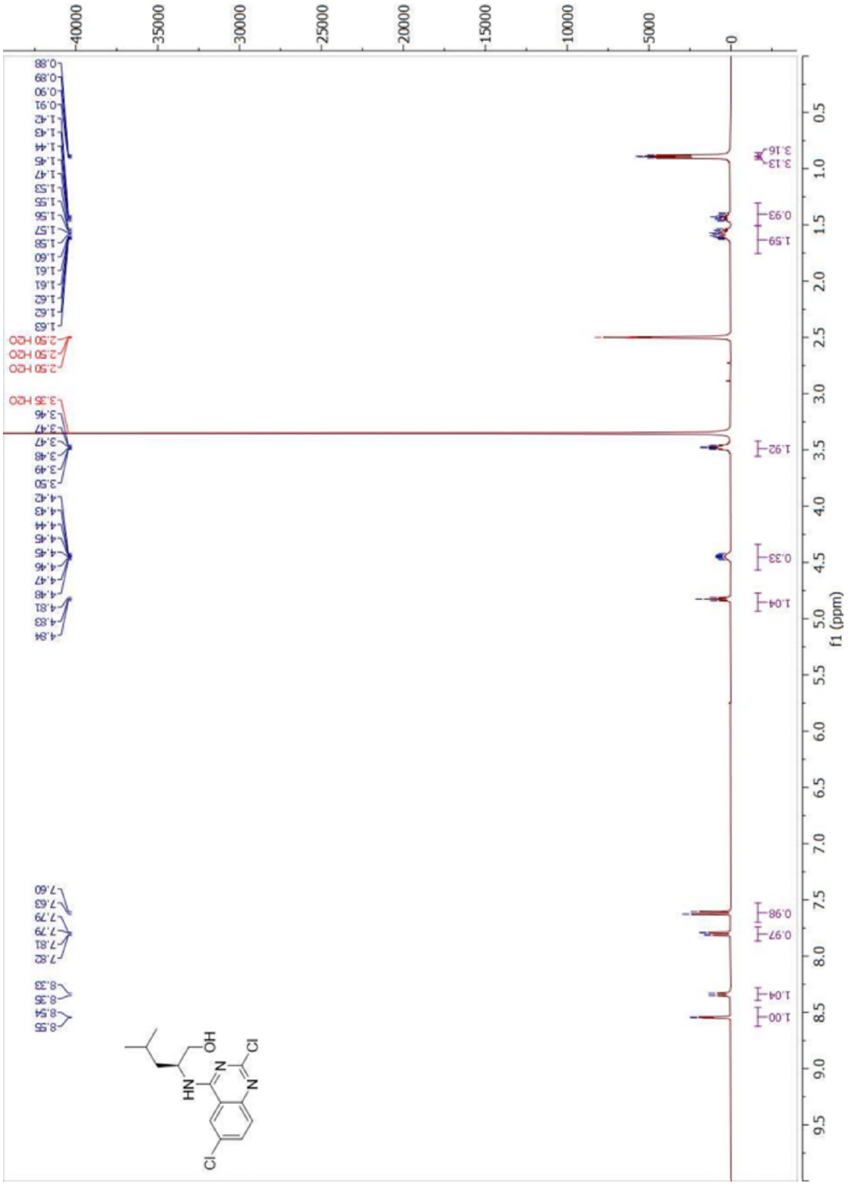

$^{13}C$ NMR

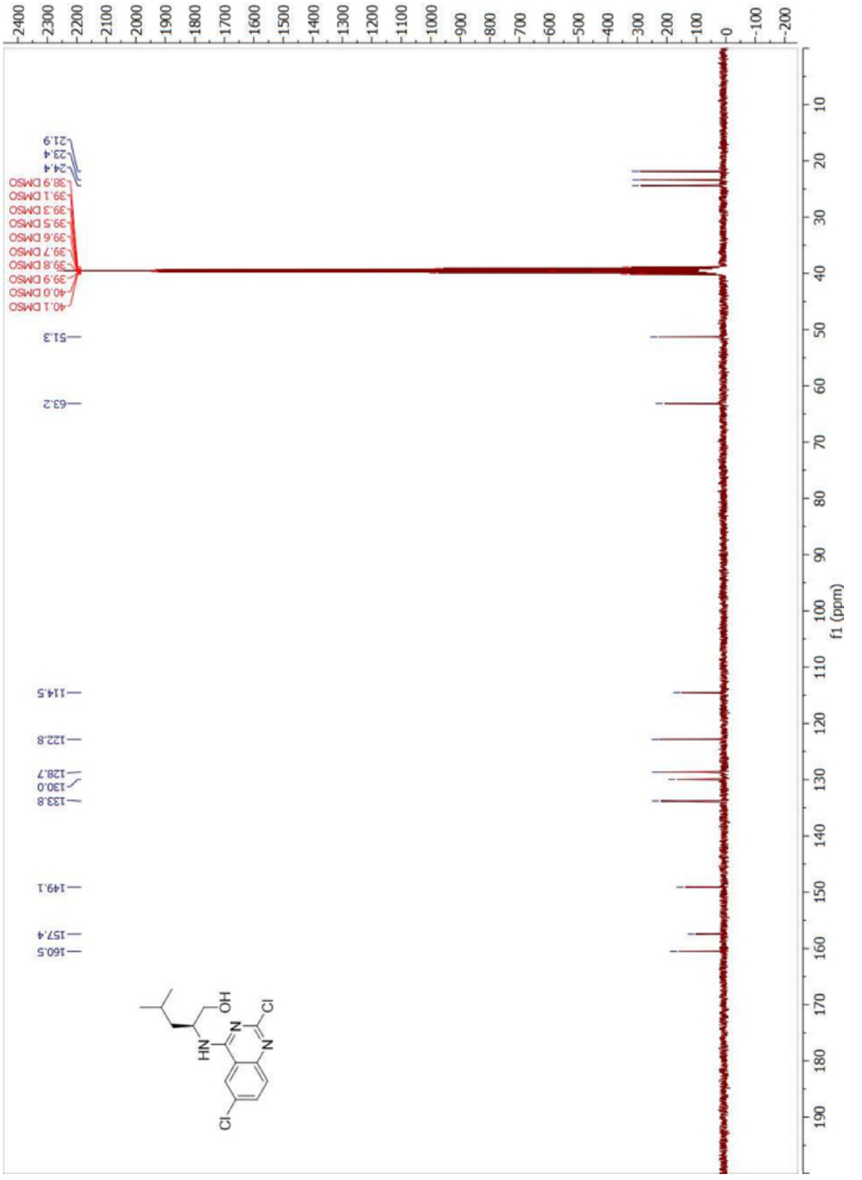

**(*S*)-2-(2-Allylamino-6-chloroquinazolin-4-yl)amino-4-methylpentan-1-ol (9)**

$^{1}$H NMR

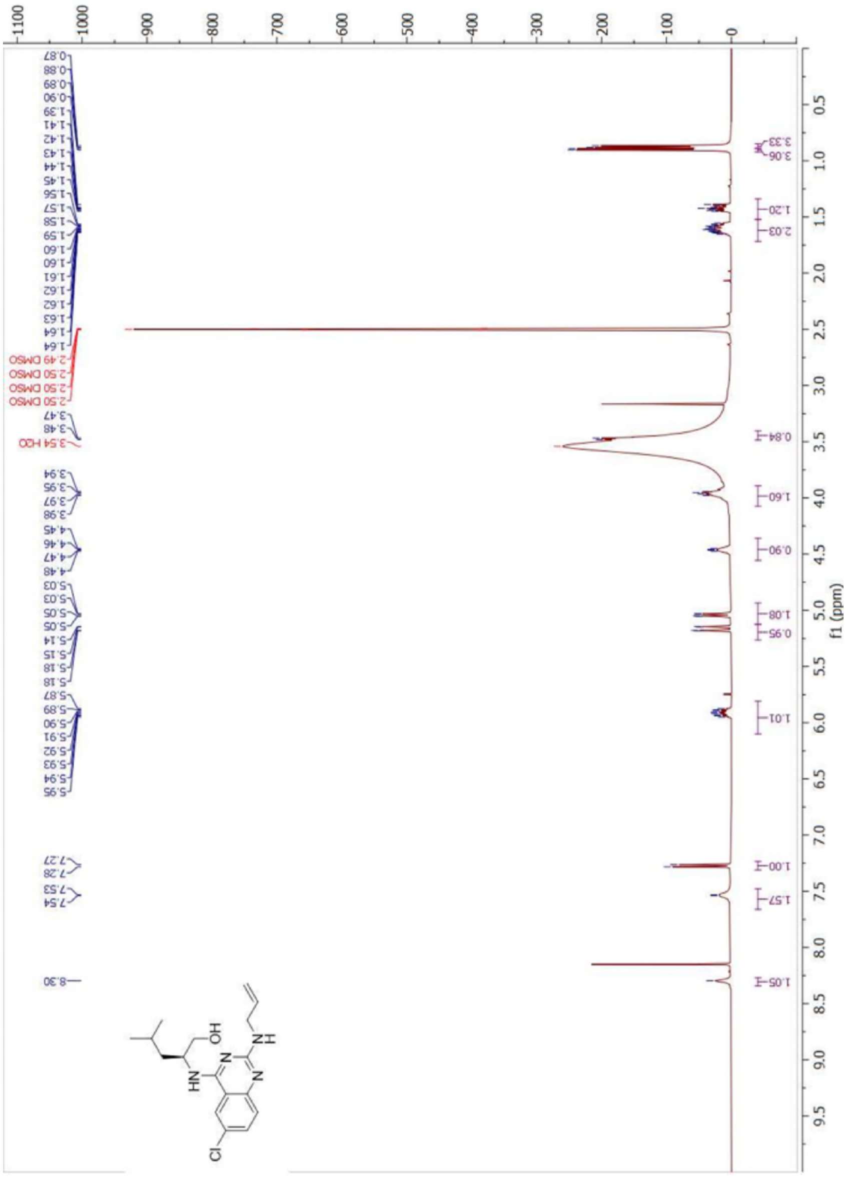

$^{13}C$ NMR

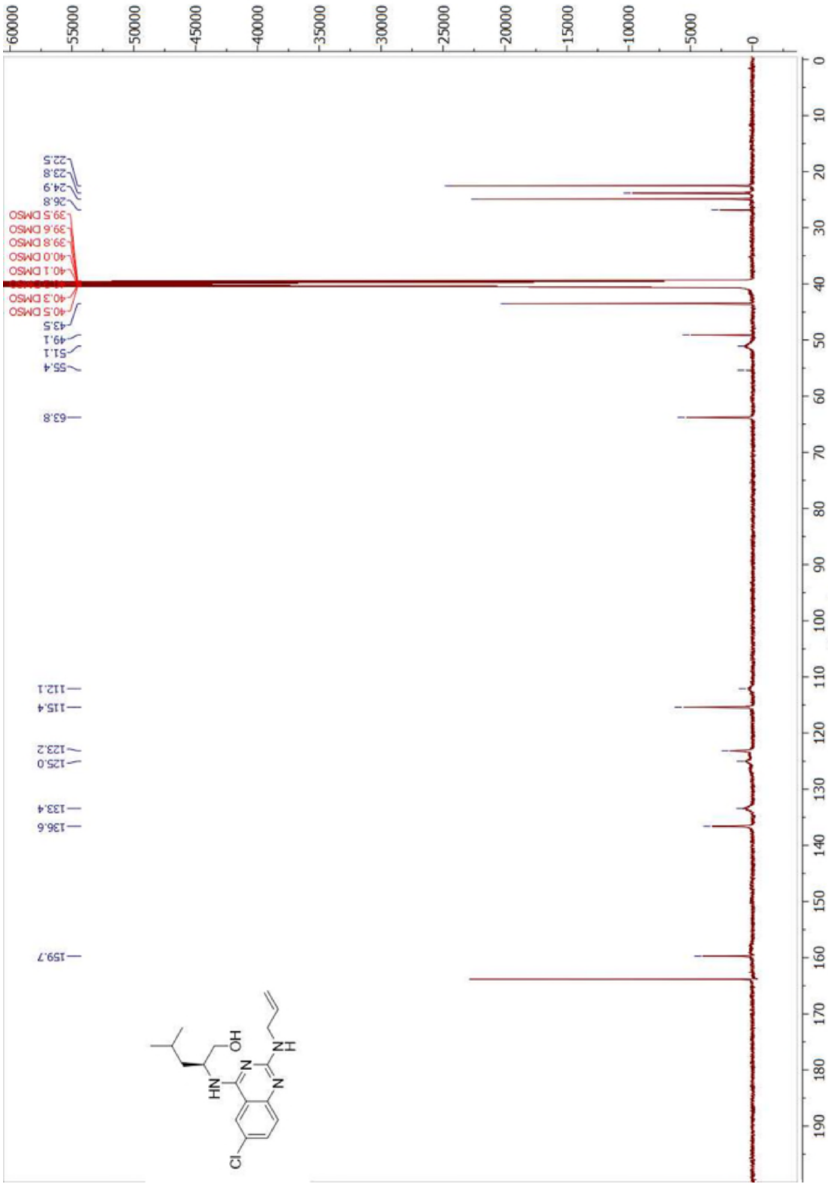

22.5
23.8
24.9
26.8
39.5 DMSO
39.6 DMSO
39.8 DMSO
40.0 DMSO
40.1 DMSO
40.3 DMSO
40.5 DMSO
43.5
49.1
51.1
55.4
63.8
112.1
115.4
123.2
125.0
133.4
136.6
159.7
f1 (ppm)

**Methyl (2,6-dichloroquinazolin-4-yl)leucinate (35)**

$^{1}H$ NMR

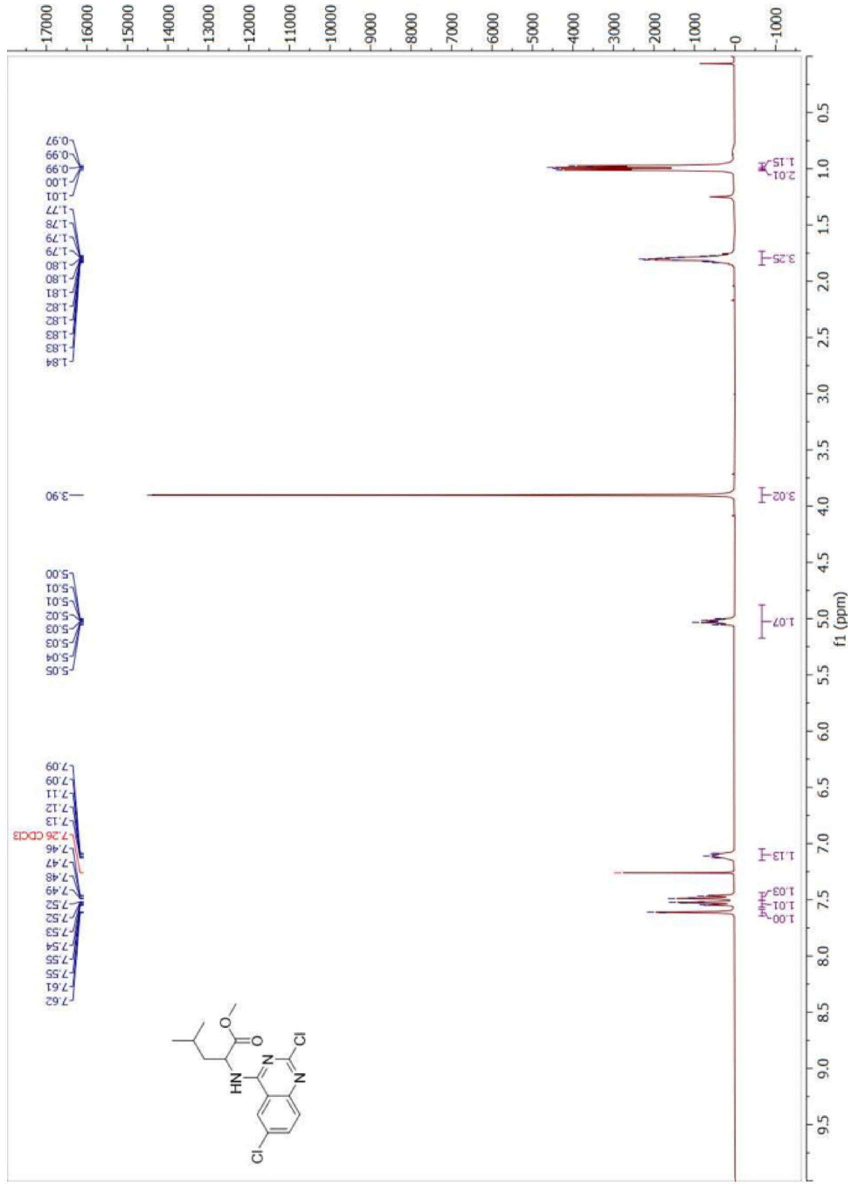

$^{13}C$ NMR

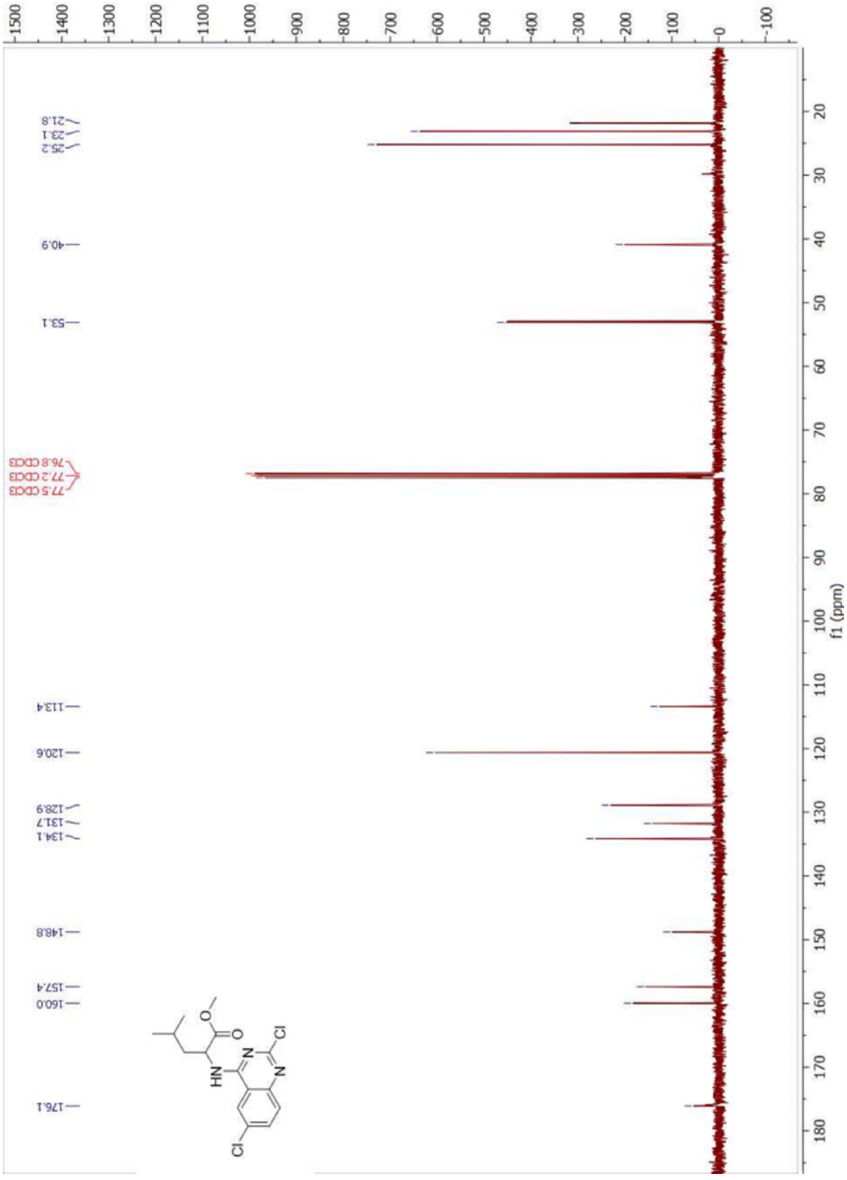

21.8
23.1
25.2
40.9
53.1
76.8 CDCl3
77.2 CDCl3
77.5 CDCl3
113.4
120.6
128.9
131.7
134.1
148.8
157.4
160.0
176.1
f1 (ppm)

**Methyl (2-allylamino-6-chloroquinazolin-4-yl)leucinate (36)**

$^{1}$H NMR

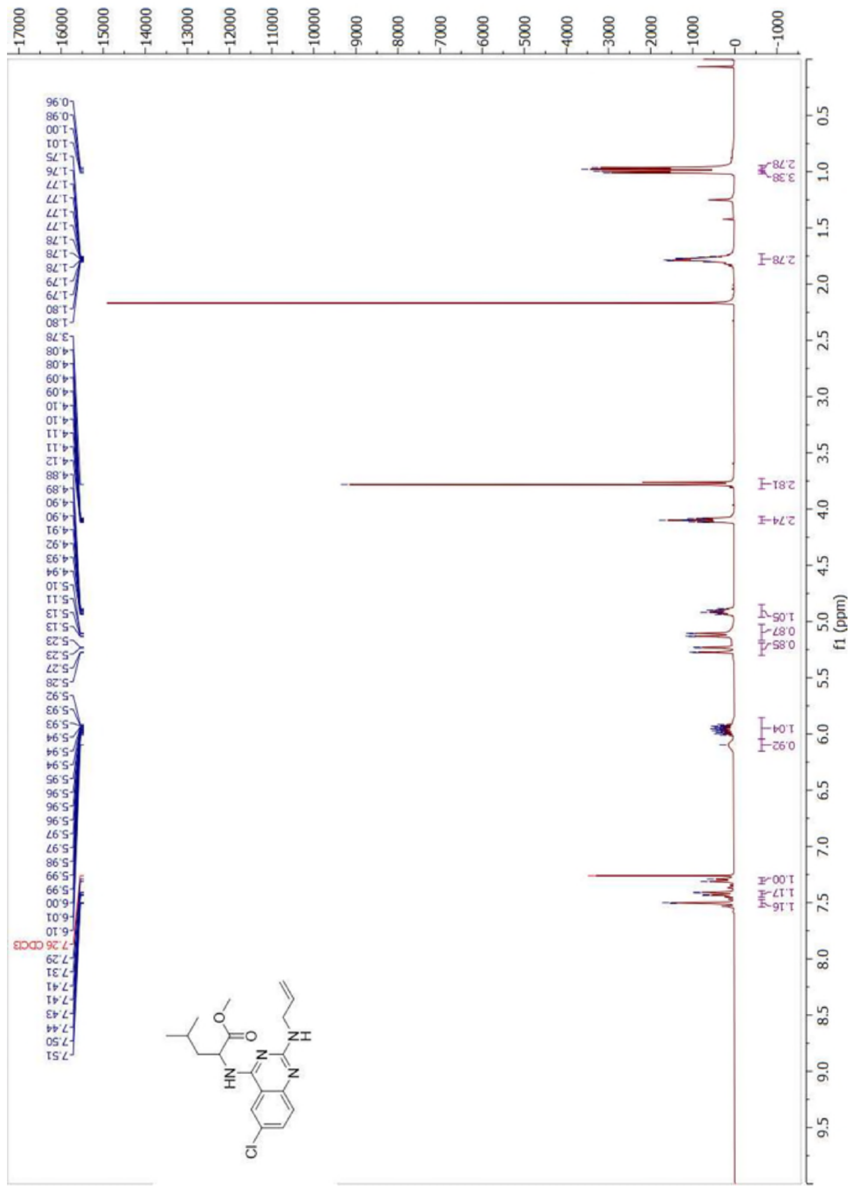

$^{13}C$ NMR

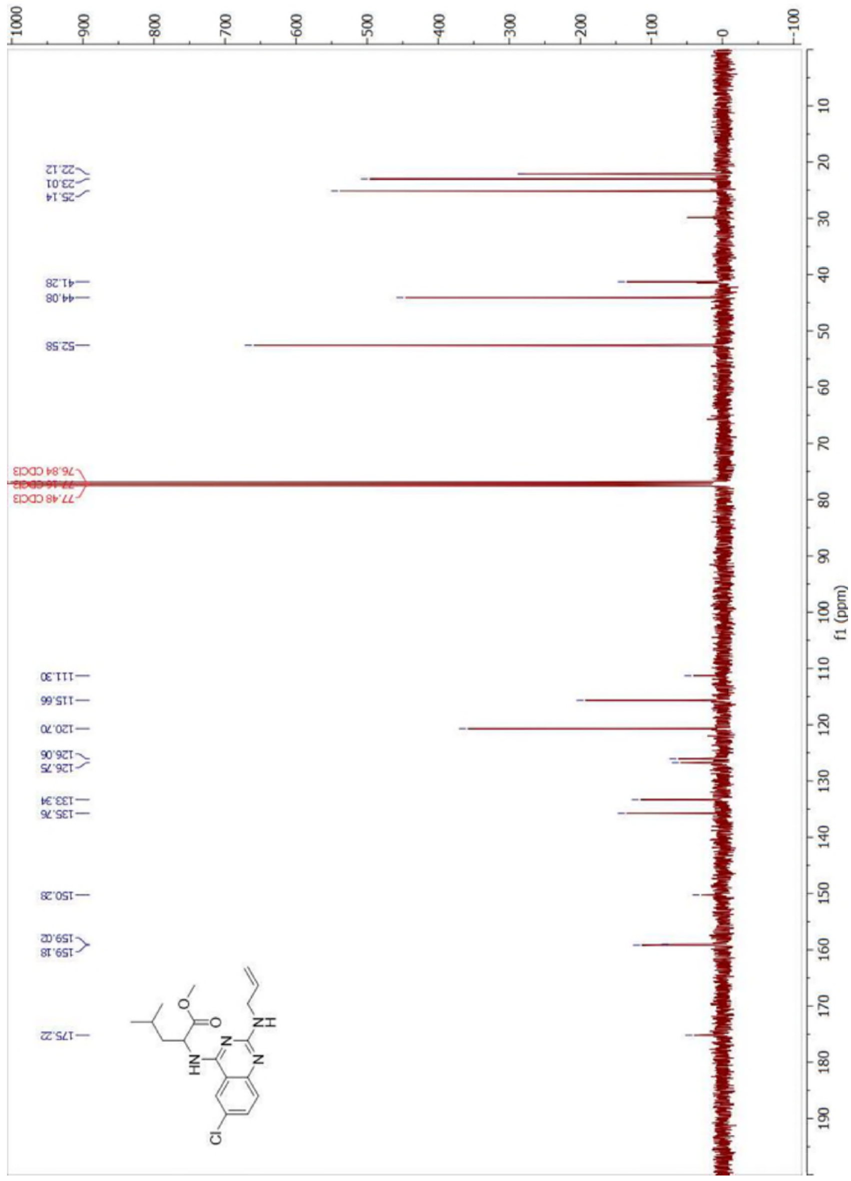

22.12
23.01
25.14
41.28
44.08
52.58
76.84 CDCl3
77.16 CDCl3
77.48 CDCl3
111.30
115.66
120.70
126.06
126.75
133.34
135.76
150.28
159.02
159.18
175.22
f1 (ppm)

**2-(2-allylamino-6-chloroquinazolin-4-yl)amino-*N*-hydroxy-4-methylpentanamide (10)**

$^{1}$H NMR

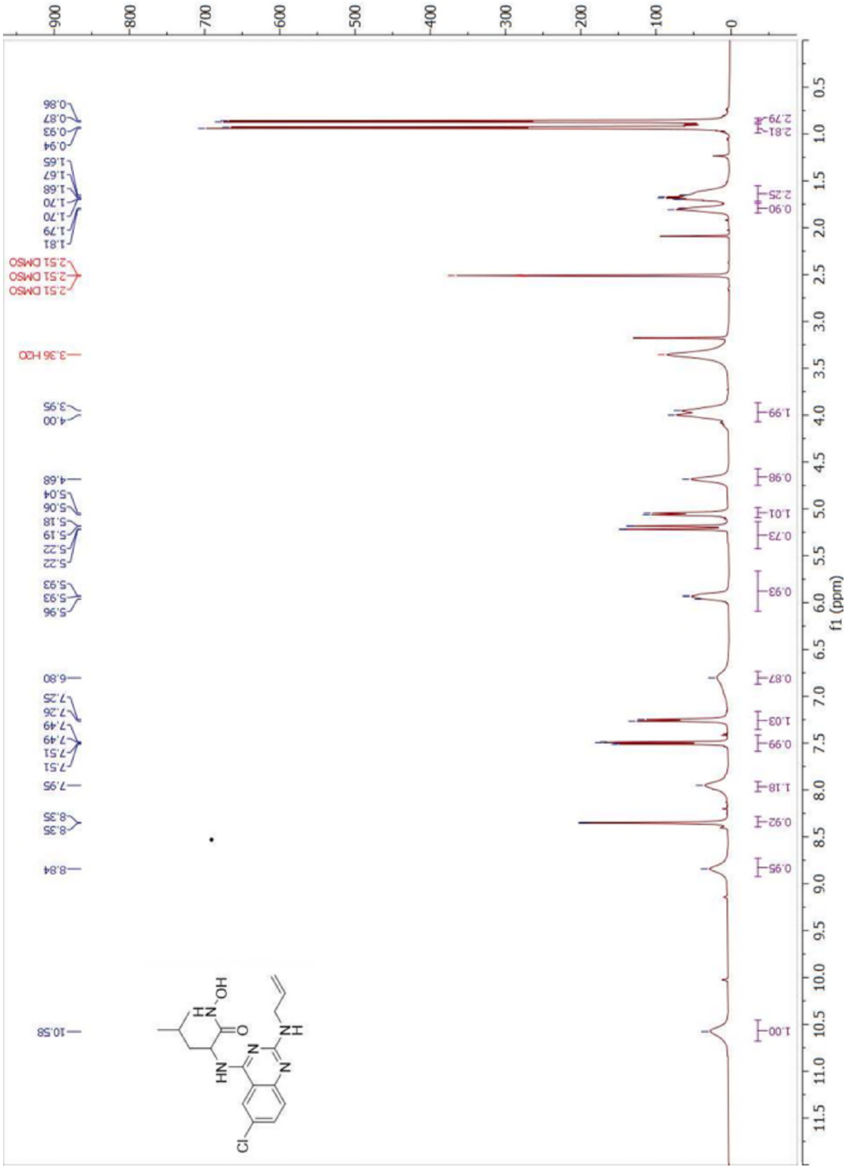

$^{13}C$ NMR

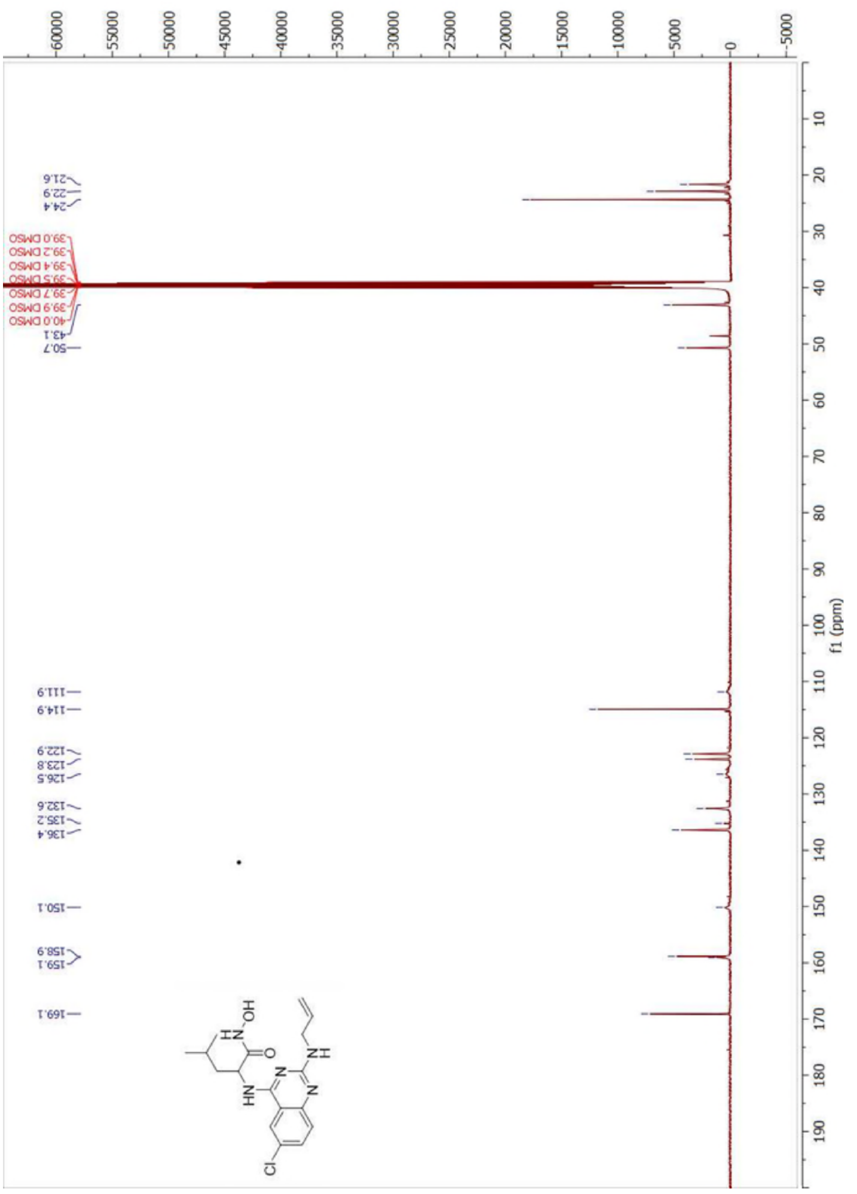
21.6
22.9
24.4
39.0 DMSO
39.2 DMSO
39.4 DMSO
39.5 DMSO
39.7 DMSO
39.9 DMSO
40.0 DMSO
43.1
50.7
111.9
114.9
122.9
123.8
126.5
132.6
135.2
136.4
150.1
158.9
159.1
169.1
f1 (ppm)

**Methyl (2,6-dichloroquinazolin-4-yl-glycinate (37)**

$^1$H NMR

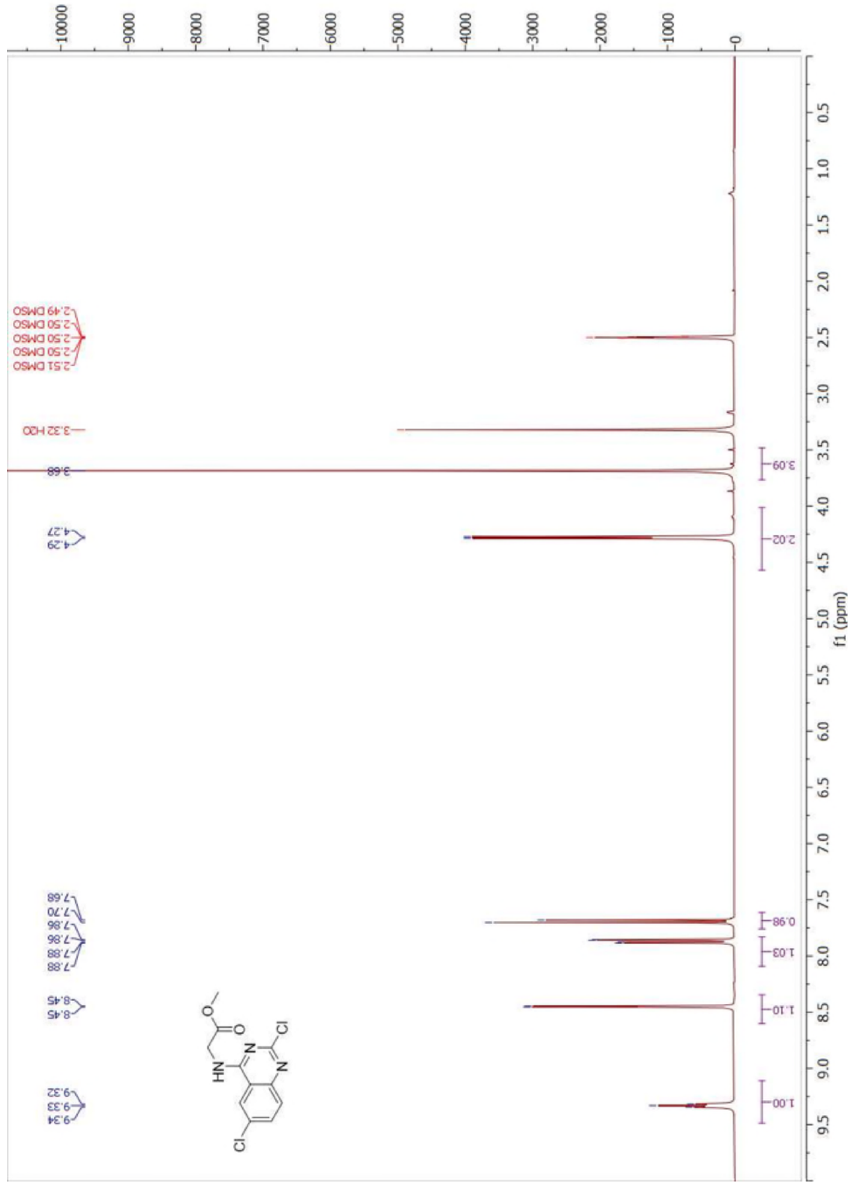

$^{13}C$ NMR

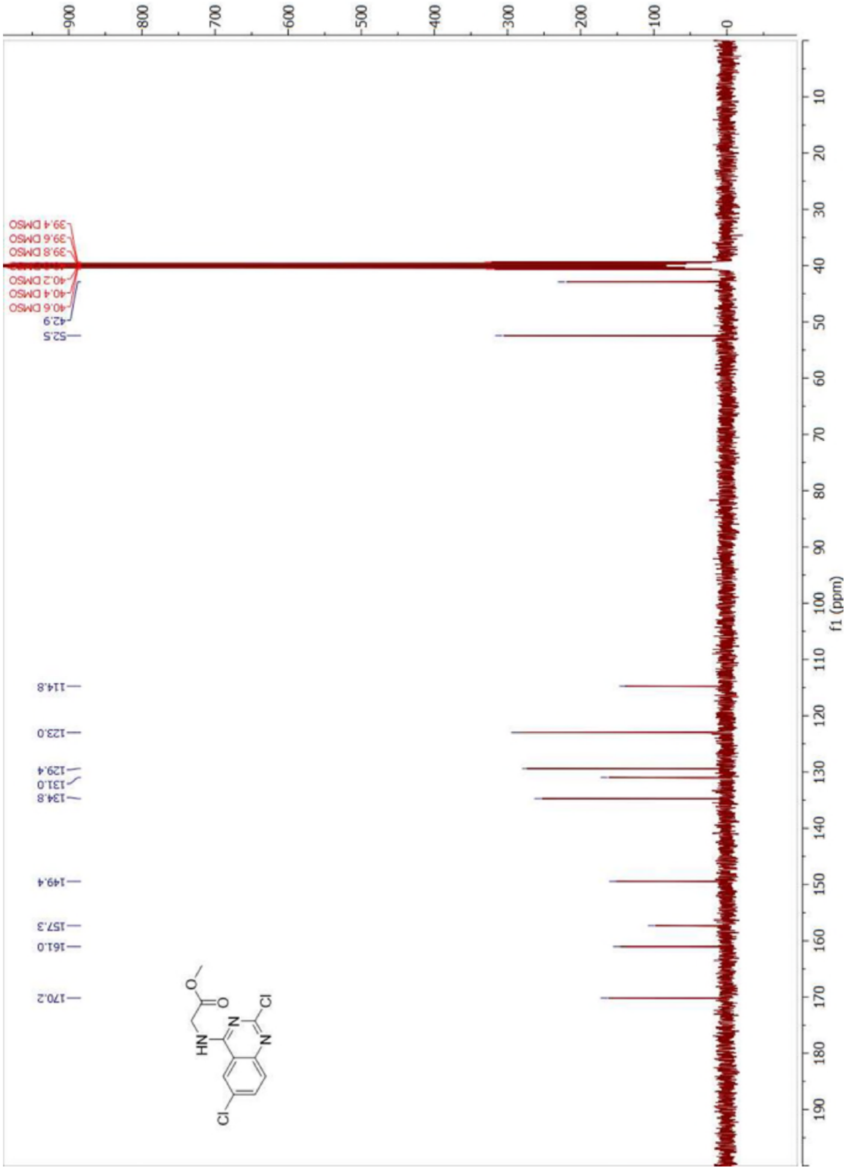
39.4 DMSO
39.6 DMSO
39.8 DMSO
40.2 DMSO
40.4 DMSO
40.6 DMSO
42.9
52.5
114.8
123.0
129.4
131.0
134.8
149.4
157.3
161.0
170.2
f1 (ppm)

**Methyl (2-allylamino-6-chloroquinazolin-4-yl)glycinate (38)**

$^{1}$H NMR

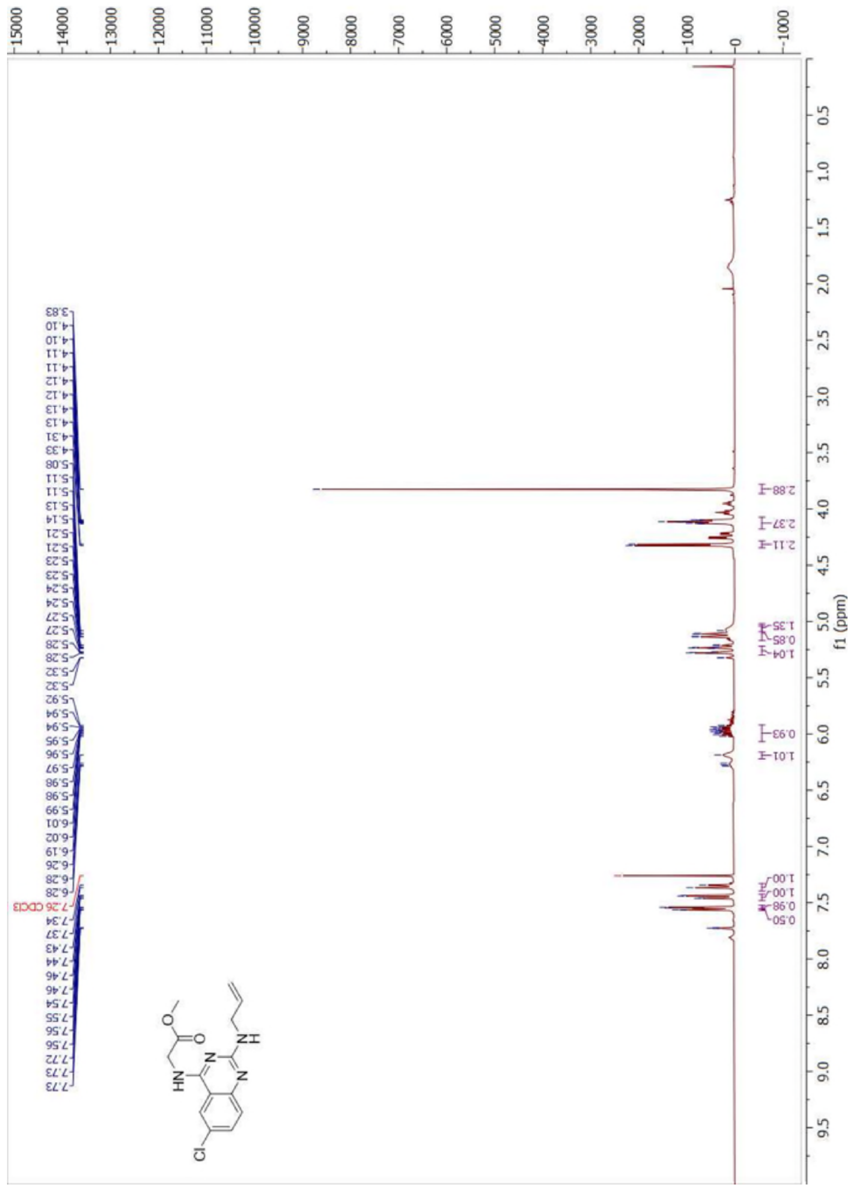

$^{13}C$ NMR

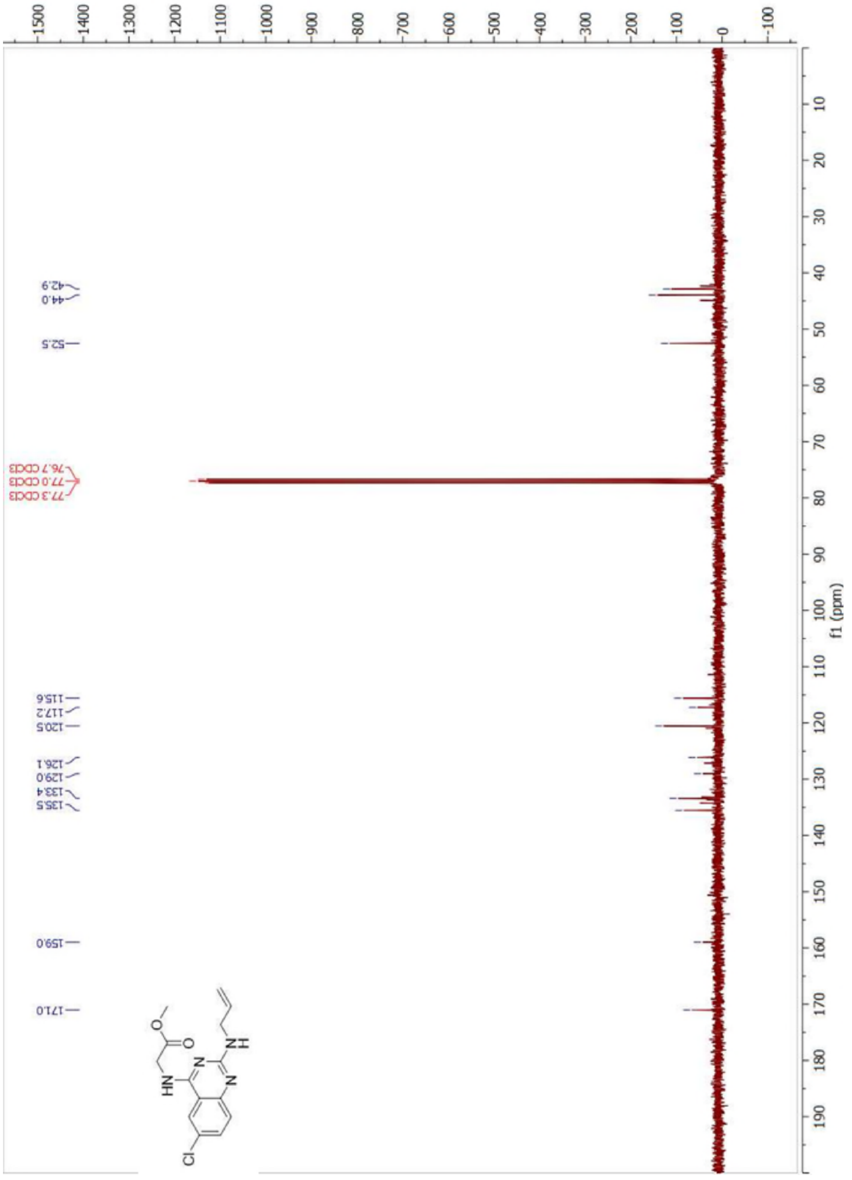

42.9
44.0
52.5
76.7 CDCl3
77.0 CDCl3
77.3 CDCl3
115.6
117.2
120.5
126.1
129.0
133.4
135.5
159.0
171.0
f1 (ppm)

**2-(2-Allylamino-6-chloroquinazolin-4-yl)amino-*N*-hydroxyethanamide (11)**

$^{1}$H NMR

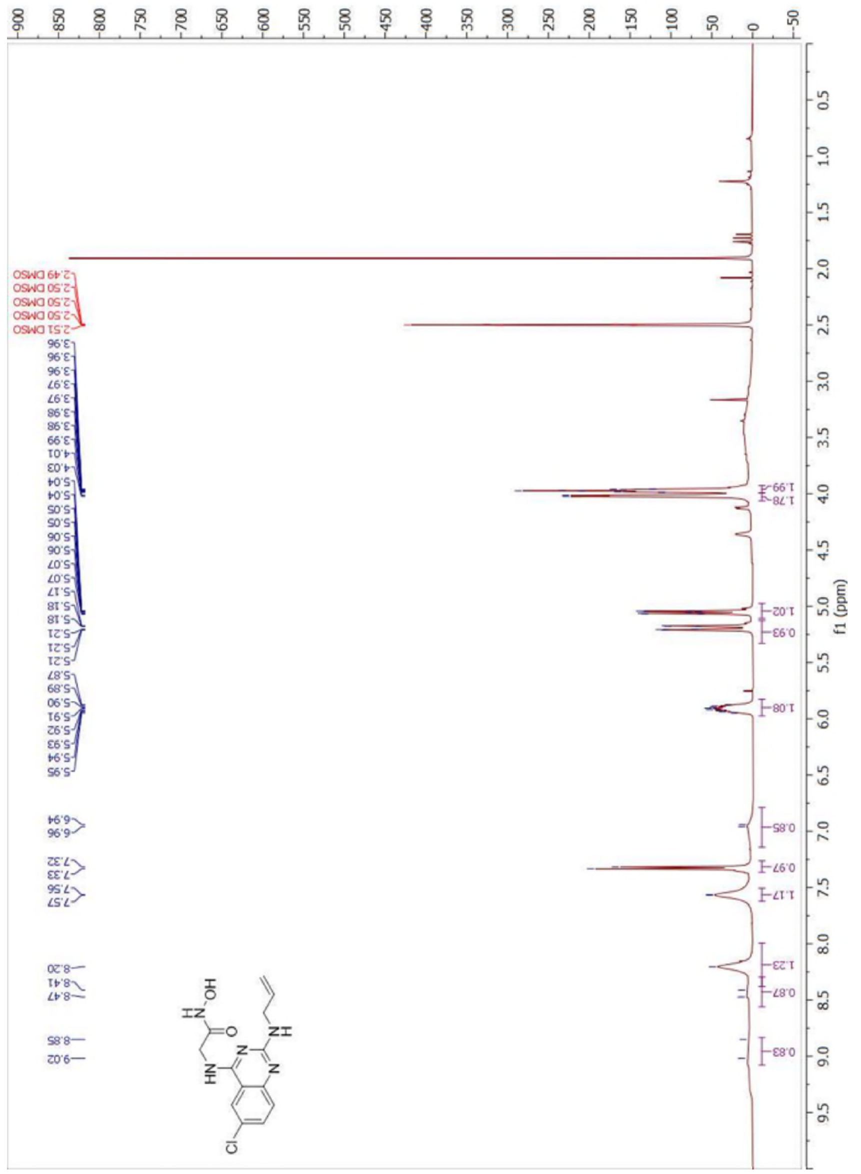

$^{13}C$ NMR

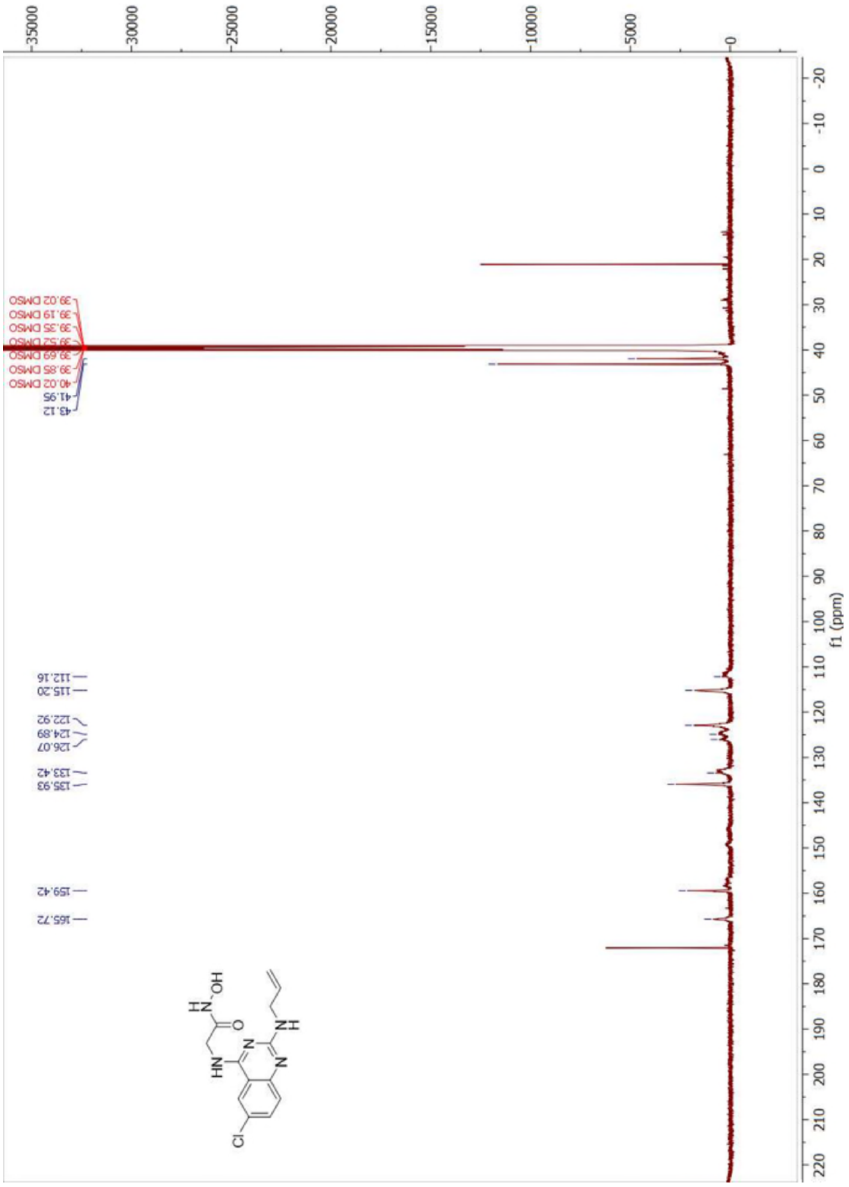
39.02 DMSO
39.19 DMSO
39.35 DMSO
39.52 DMSO
39.69 DMSO
39.85 DMSO
40.02 DMSO
41.95
43.12
112.16
115.20
122.92
124.89
126.07
133.42
135.93
159.42
165.72
f1 (ppm)

**Methyl (2,6-dichloroquinazolin-4-yl)-*D*-alaninate (39)**

$^{1}$H NMR

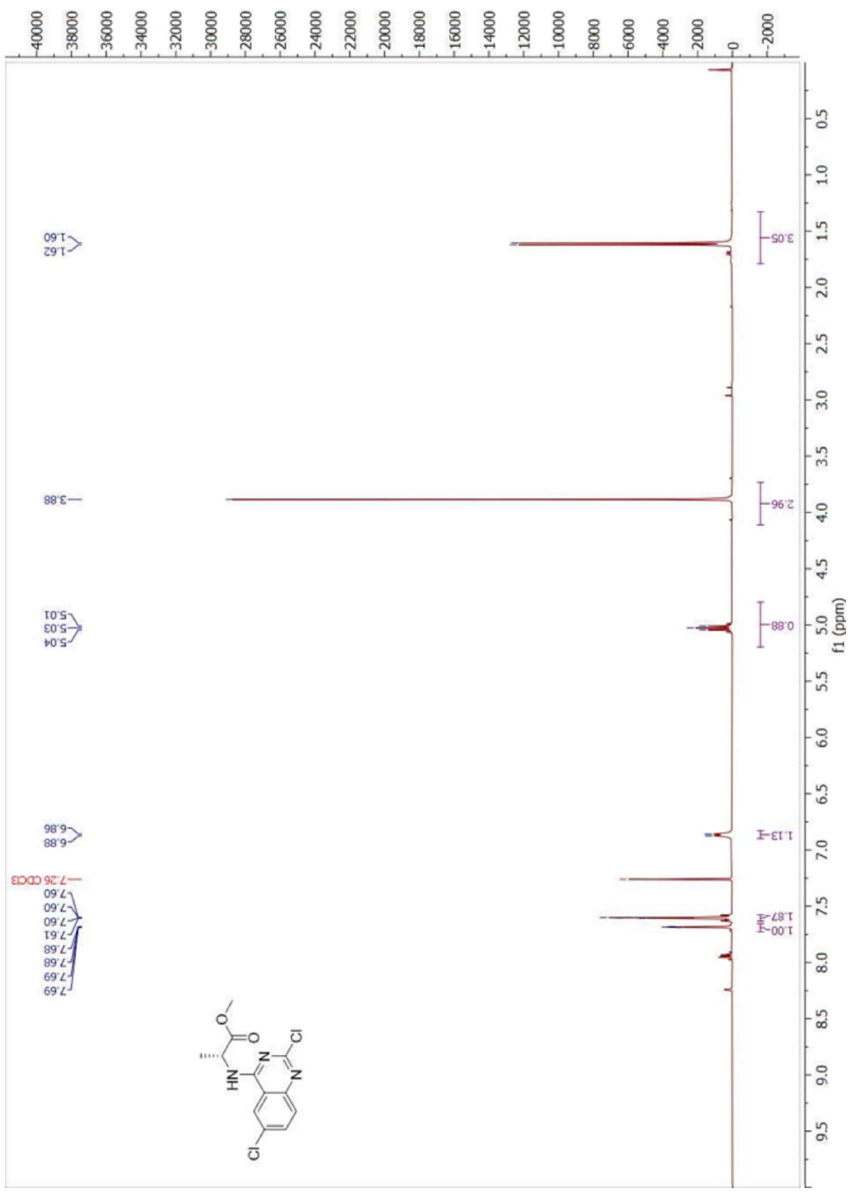

$^{13}C$ NMR

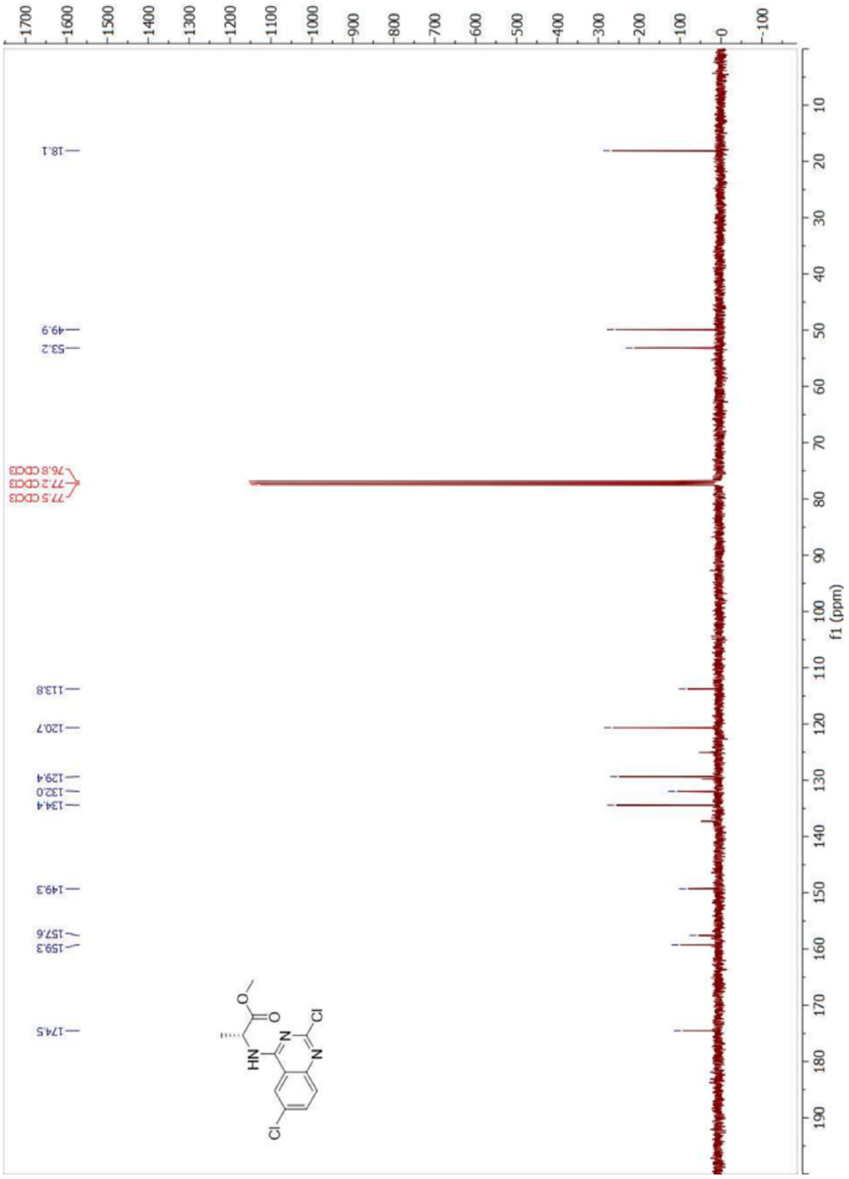
18.1
49.9
53.2
76.8 CDCl3
77.2 CDCl3
77.5 CDCl3
113.8
120.7
129.4
132.0
134.4
149.3
157.6
159.3
174.5
f1 (ppm)

**Methyl (2-allylamino-6-chloroquinazolin-4-yl)-*D*-alaninate (40)**

$^{1}$H NMR

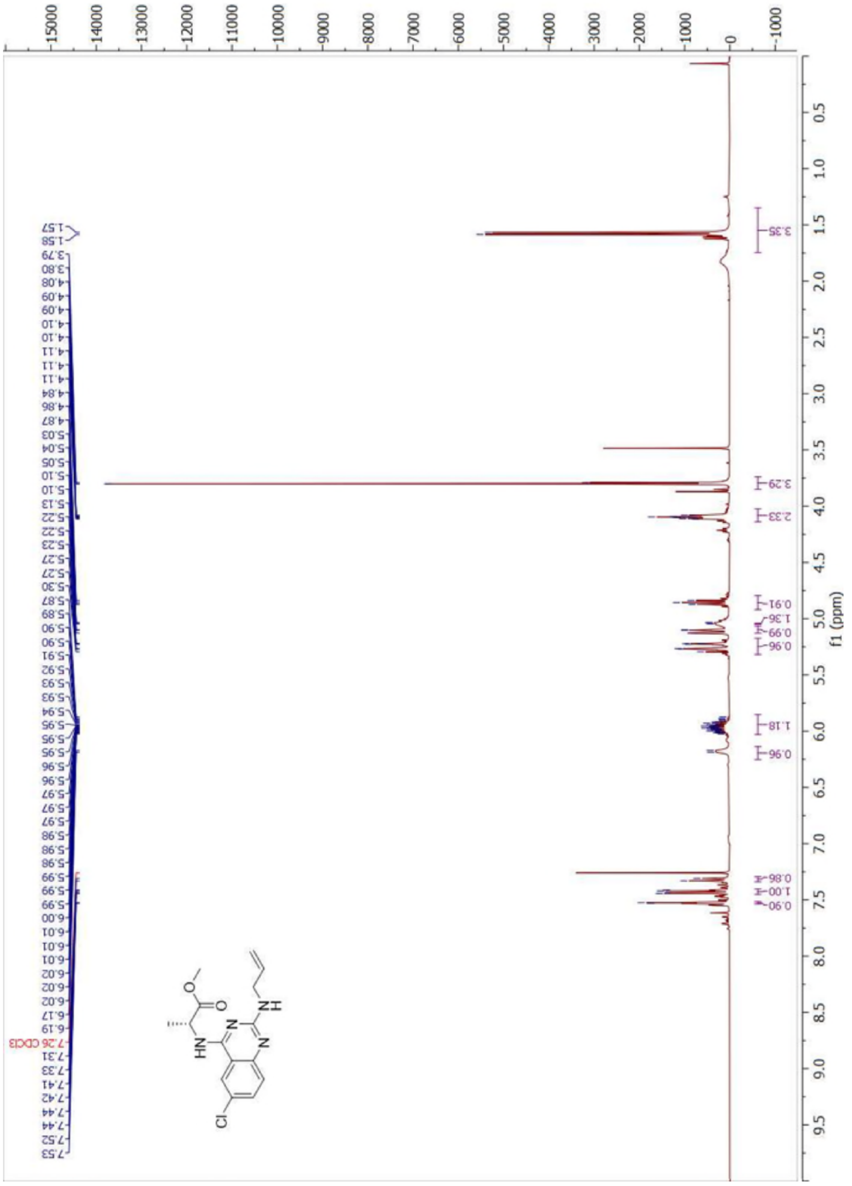

$^{13}C$ NMR

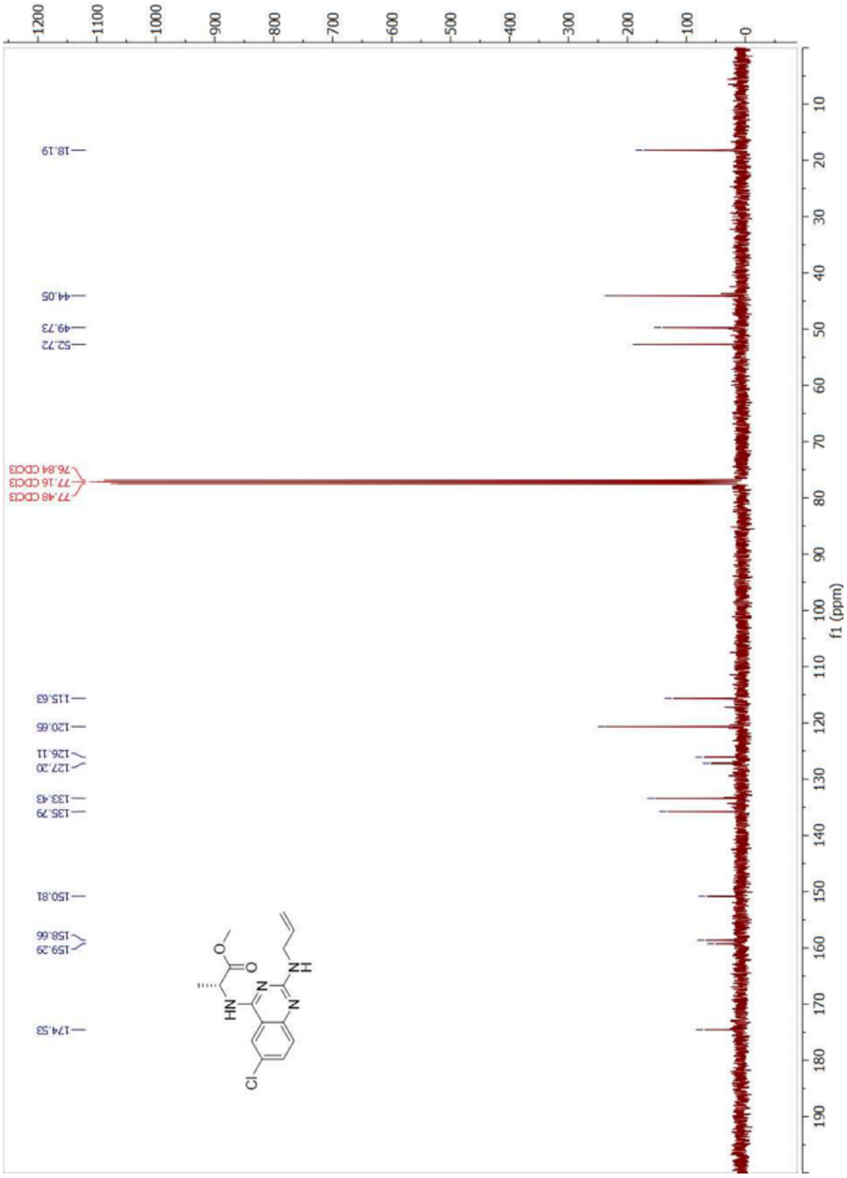
18.19
44.05
49.73
52.72
76.84 CDCl3
77.16 CDCl3
77.48 CDCl3
115.63
120.65
126.11
127.30
133.43
135.79
150.81
158.66
159.29
174.53
f1 (ppm)

## Methyl (2,6-dichloroquinazolin-4-yl-*L*-alaninate) (41)

$^{1}$H NMR

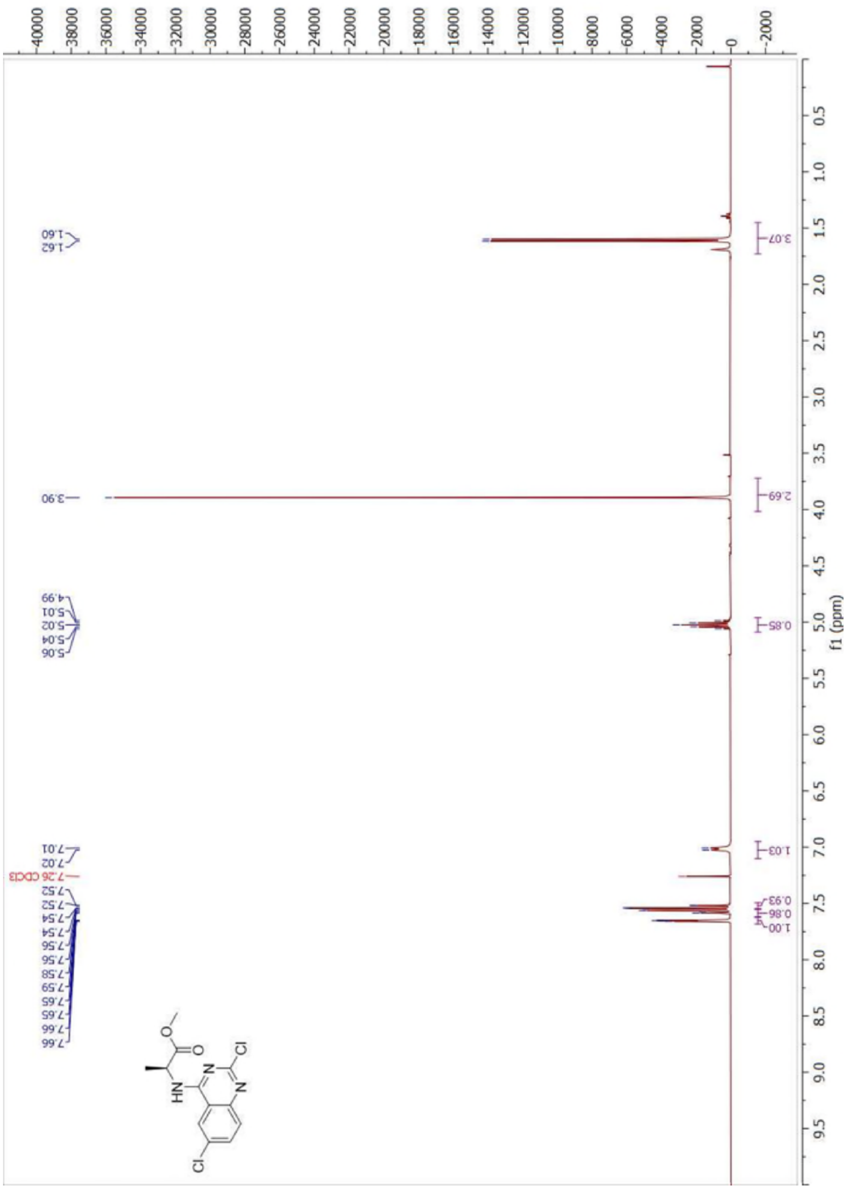

$^{13}C$ NMR

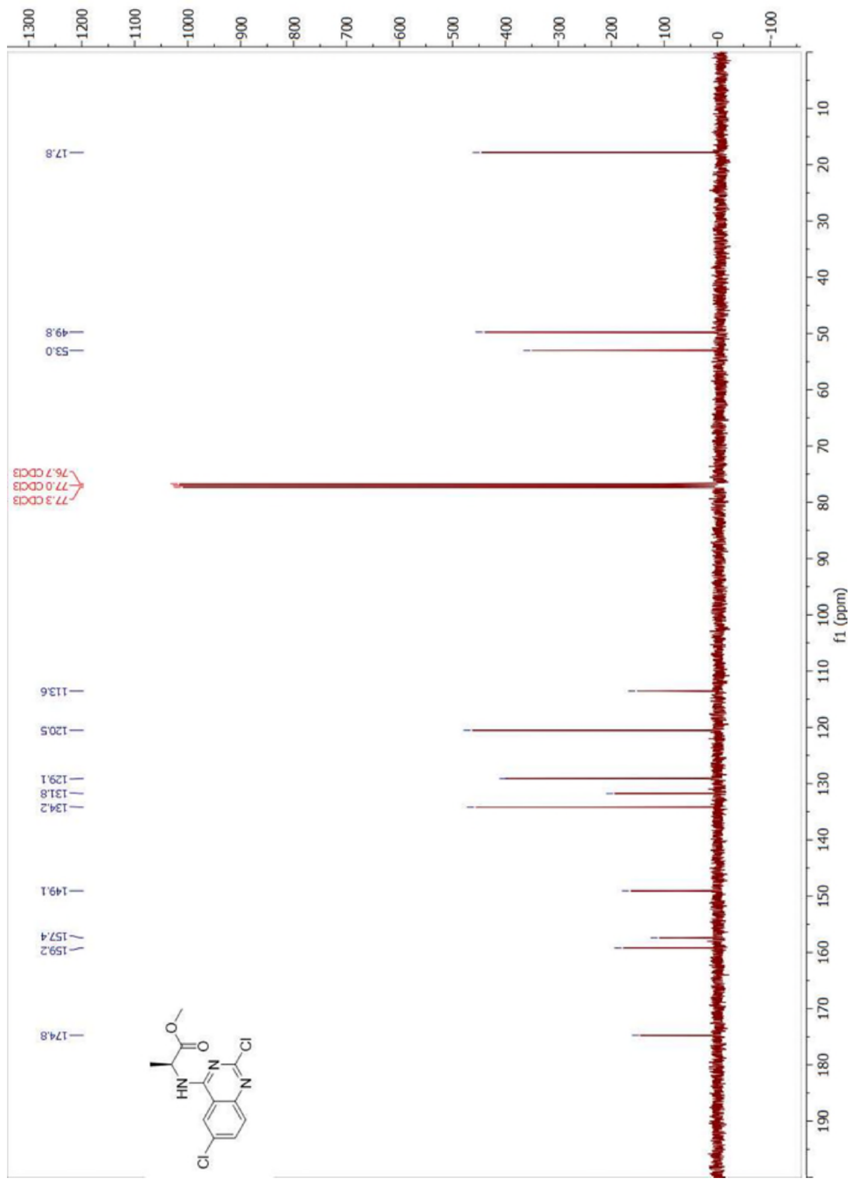

17.8
49.8
53.0
76.7 CDCl3
77.0 CDCl3
77.3 CDCl3
113.6
120.5
129.1
131.8
134.2
149.1
157.4
159.2
174.8
f1 (ppm)

**Methyl (2-allylamino-6-chloroquinazolin-4-yl)-*L*-alaninate (42)**

$^1$H NMR

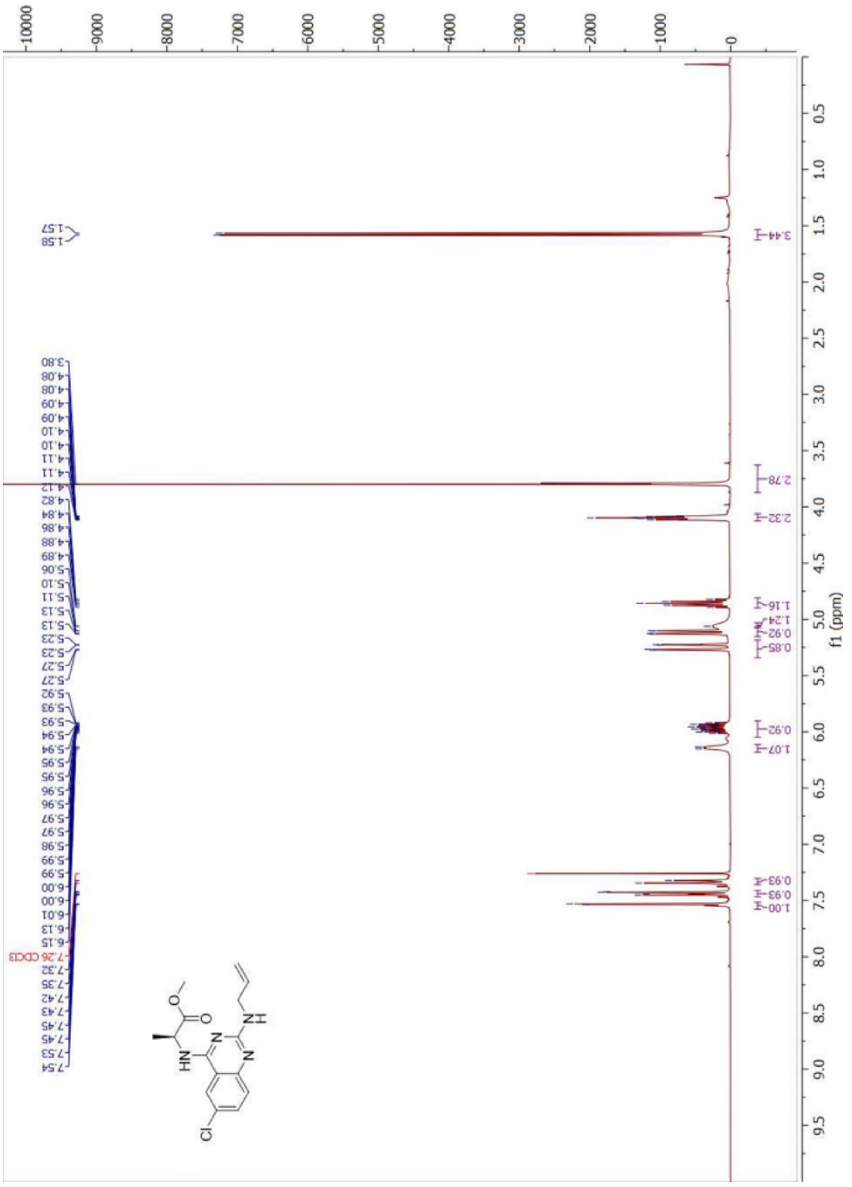

$^{13}C$ NMR

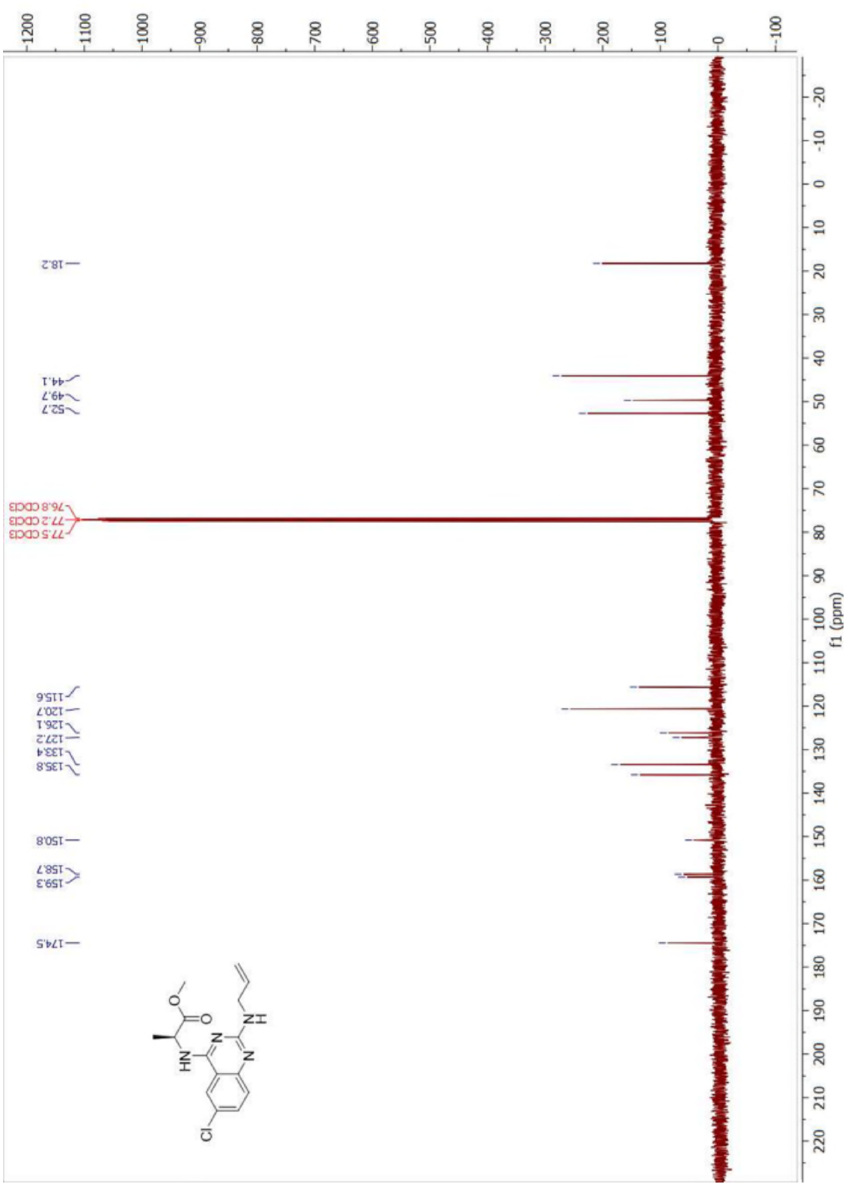

18.2
44.1
49.7
52.7
76.8 CDCl3
77.2 CDCl3
77.5 CDCl3
115.6
120.7
126.1
127.2
133.4
135.8
150.8
158.7
159.3
174.5
f1 (ppm)

**(*S*)-2-(2-Allylamino-6-chloroquinazolin-4-yl)amino-*N*-hydroxypropanamide (13)**

$^1$H NMR

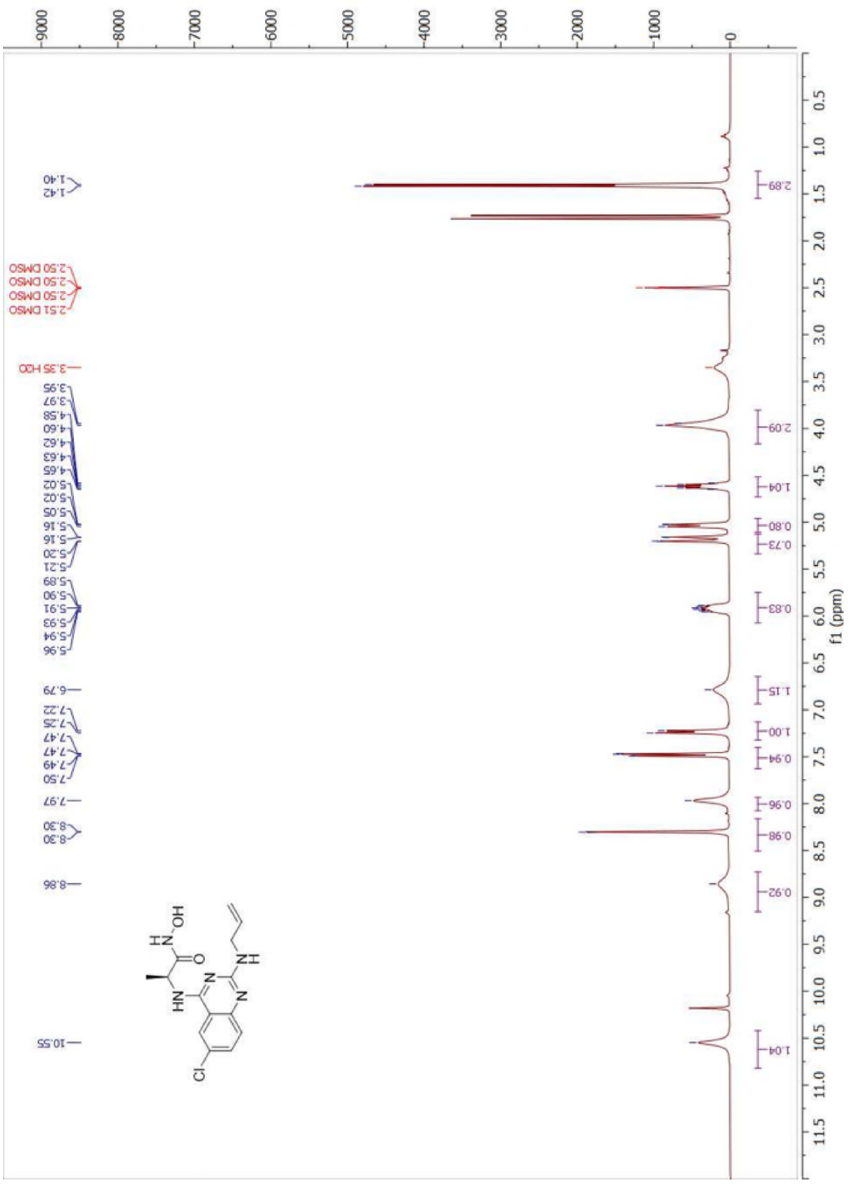

$^{13}$C NMR

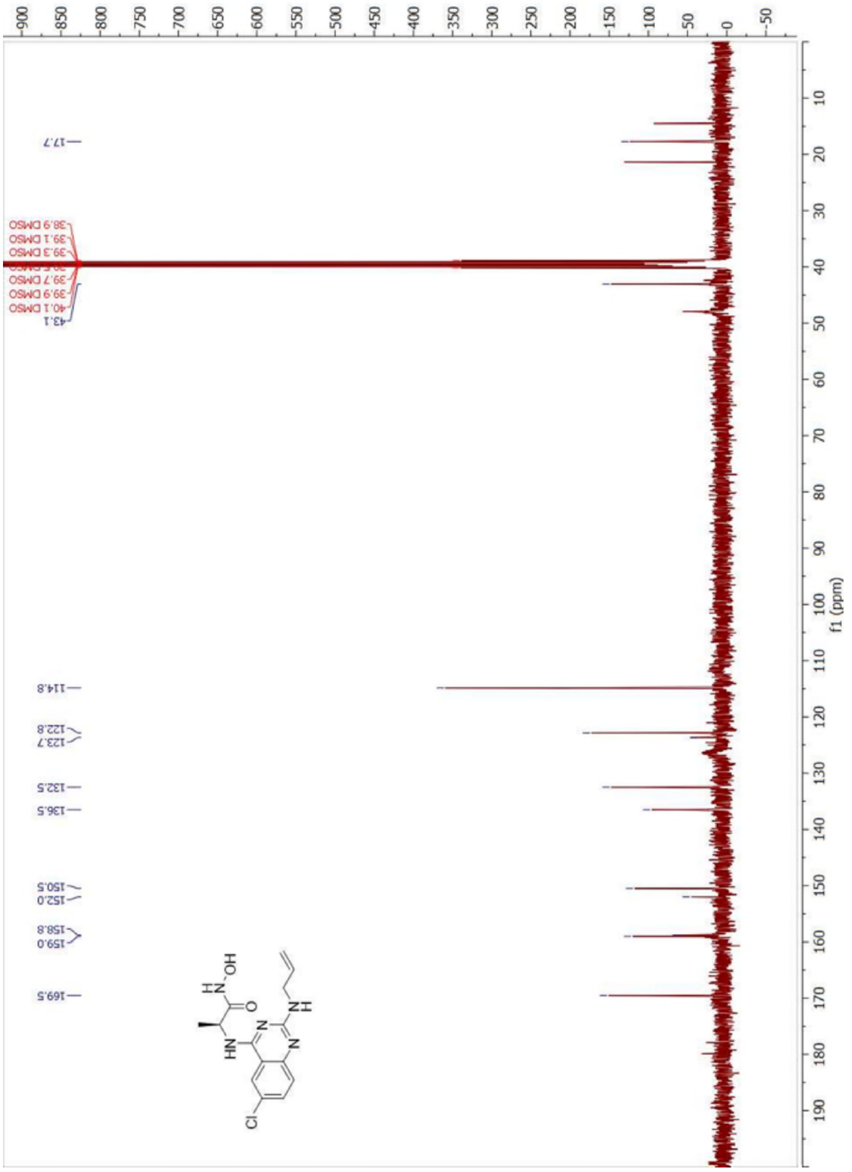

**Methyl (2,6-dichloroquinazolin-4-yl)-serinate (43)**

$^1$H NMR

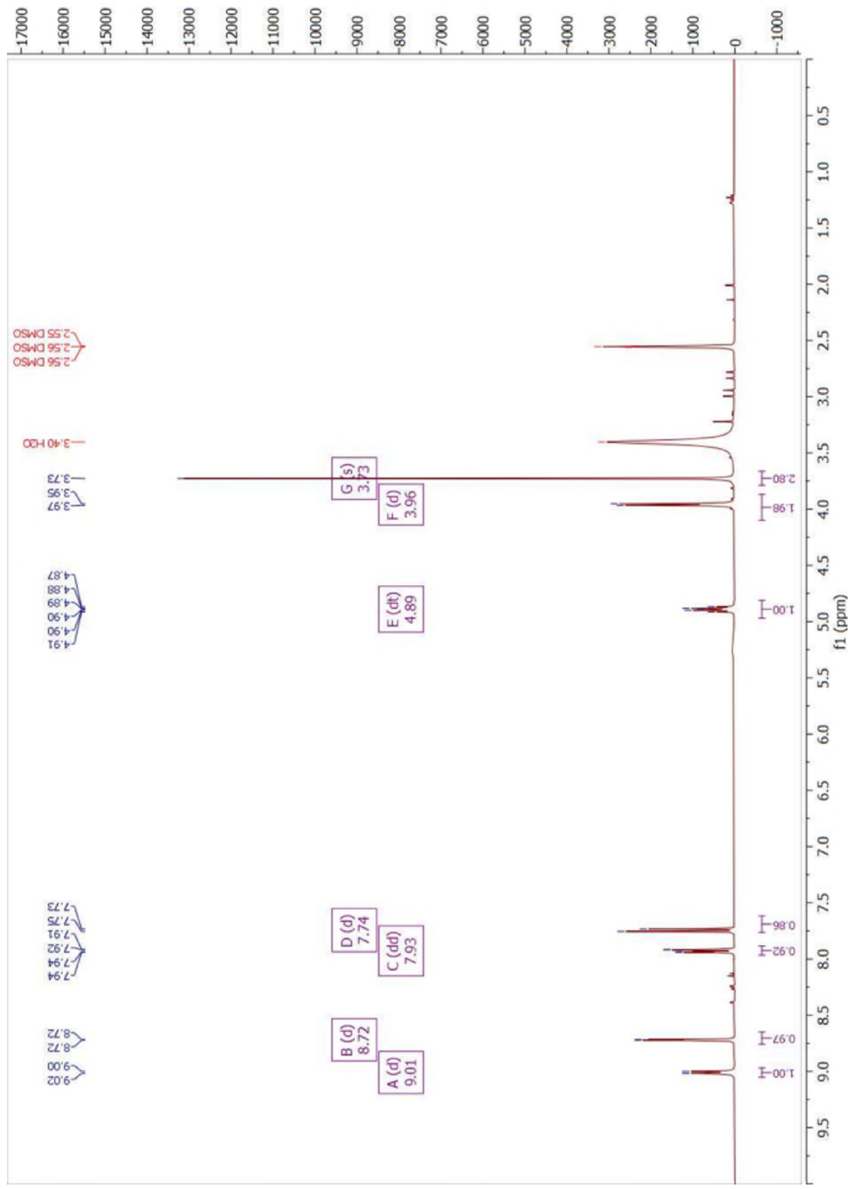

$^{13}C$ NMR

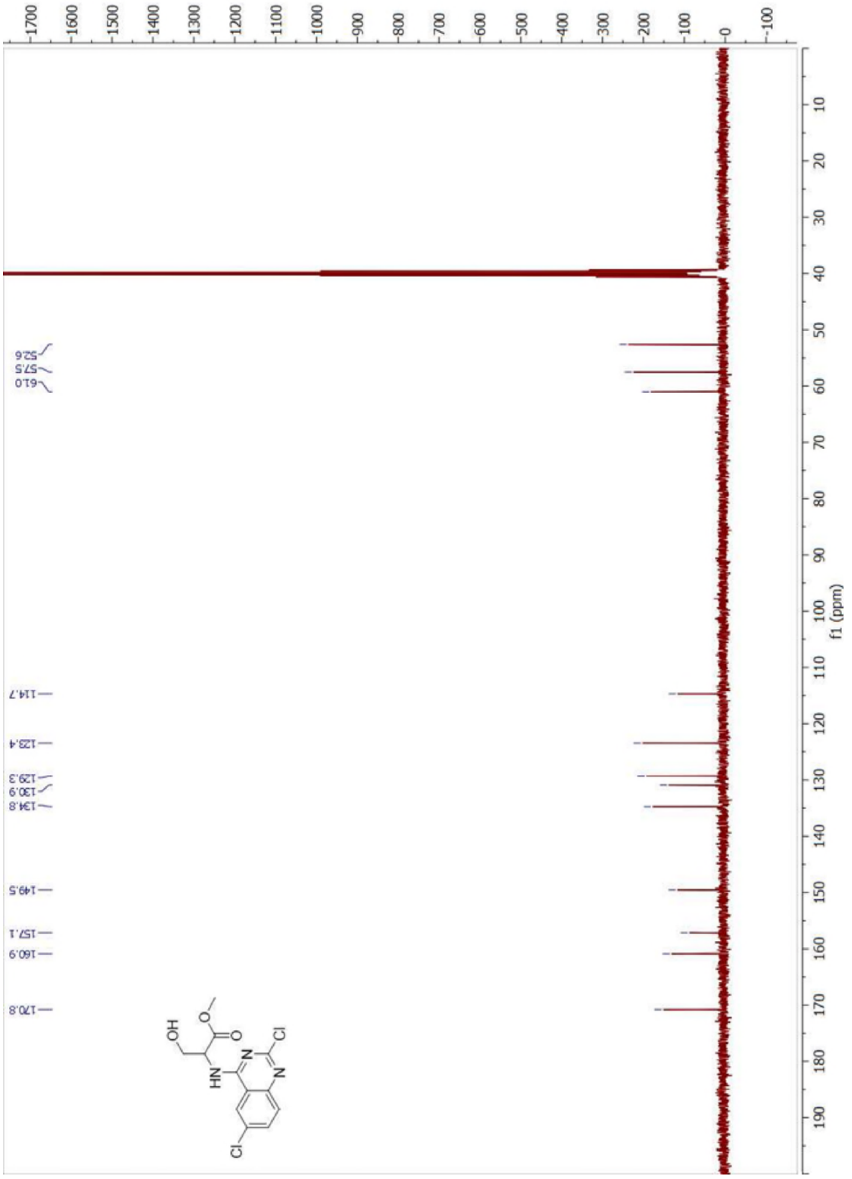

**Methyl (2-allylamino-6-chloroquinazolin-4-yl)-serinate (44)**

$^1$H NMR

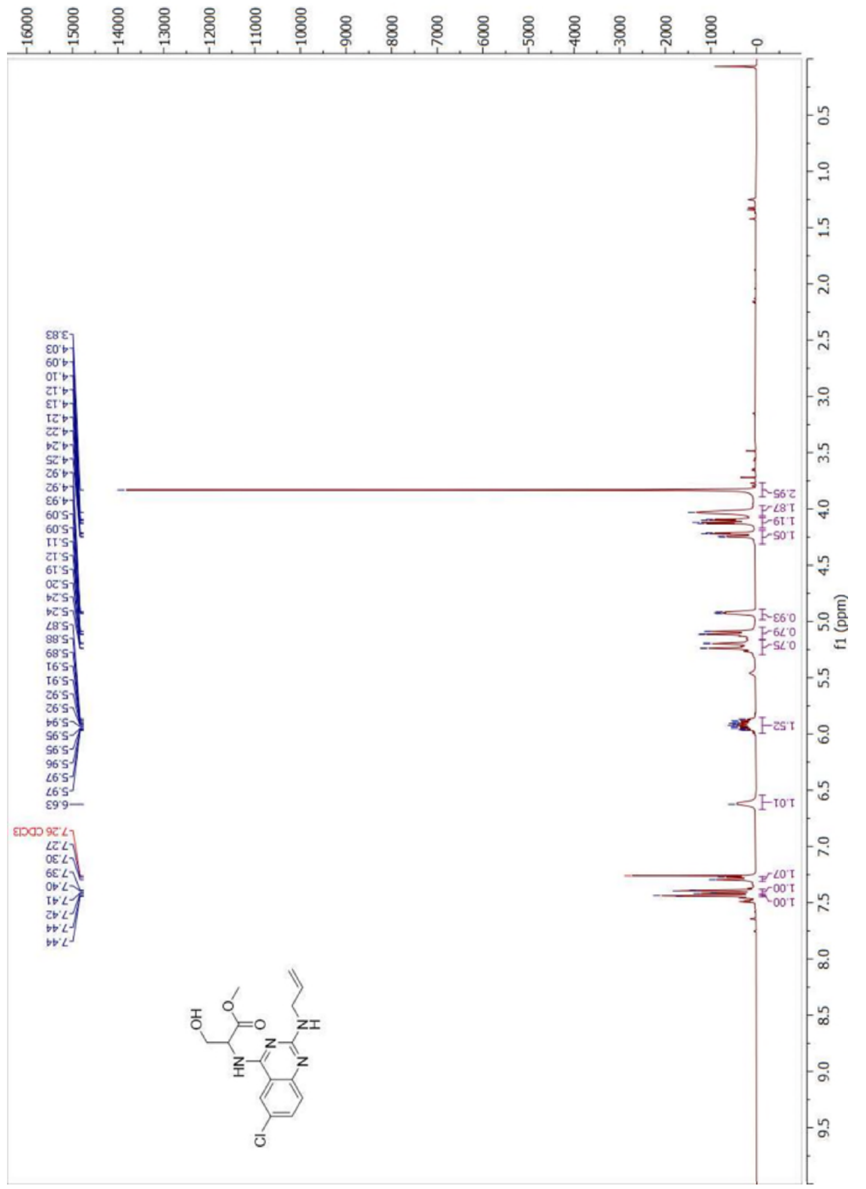

$^{13}$C NMR

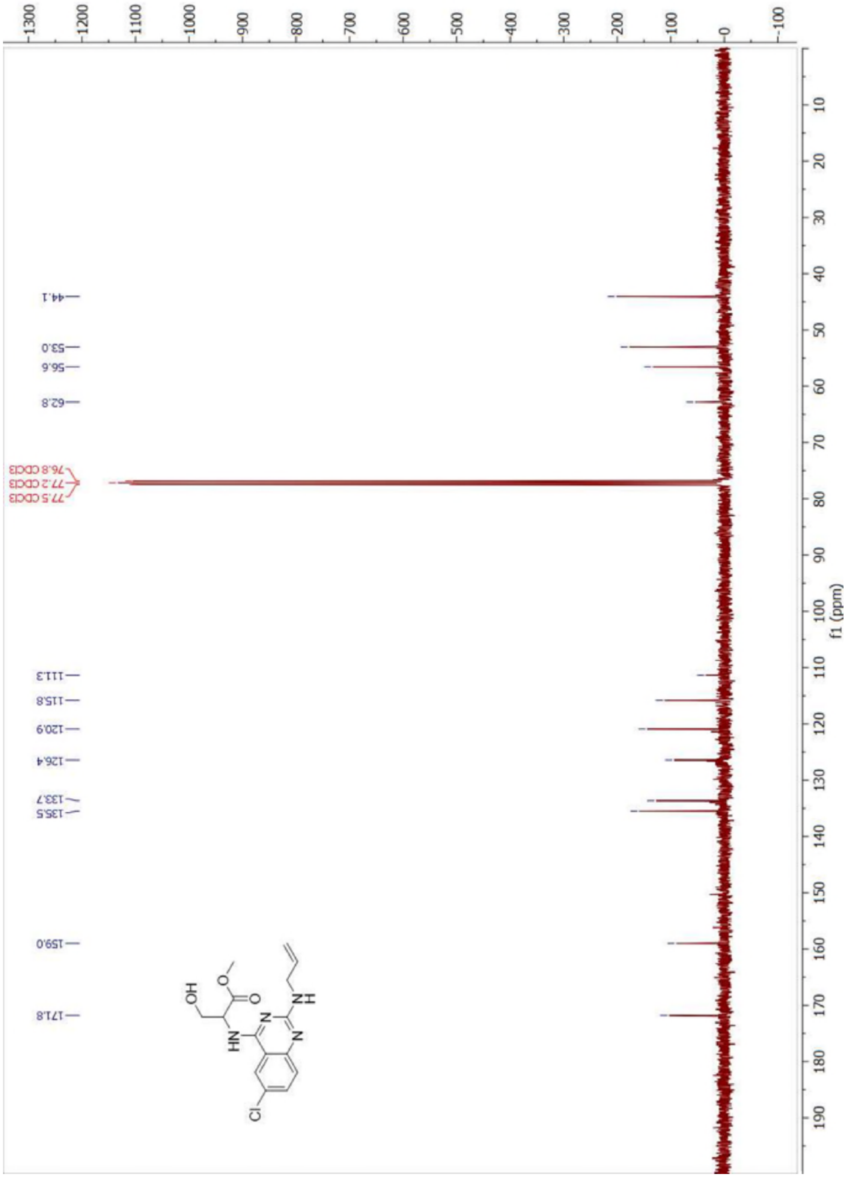
44.1
53.0
56.6
62.8
76.8 CDCl3
77.2 CDCl3
77.5 CDCl3
111.3
115.8
120.9
126.4
133.7
135.5
159.0
171.8
f1 (ppm)

**2-(2-Allylamino-6-chloroquinazolin-4-yl)amino-*N*,3-dihydroxypropanamide (14)**

$^1$H NMR

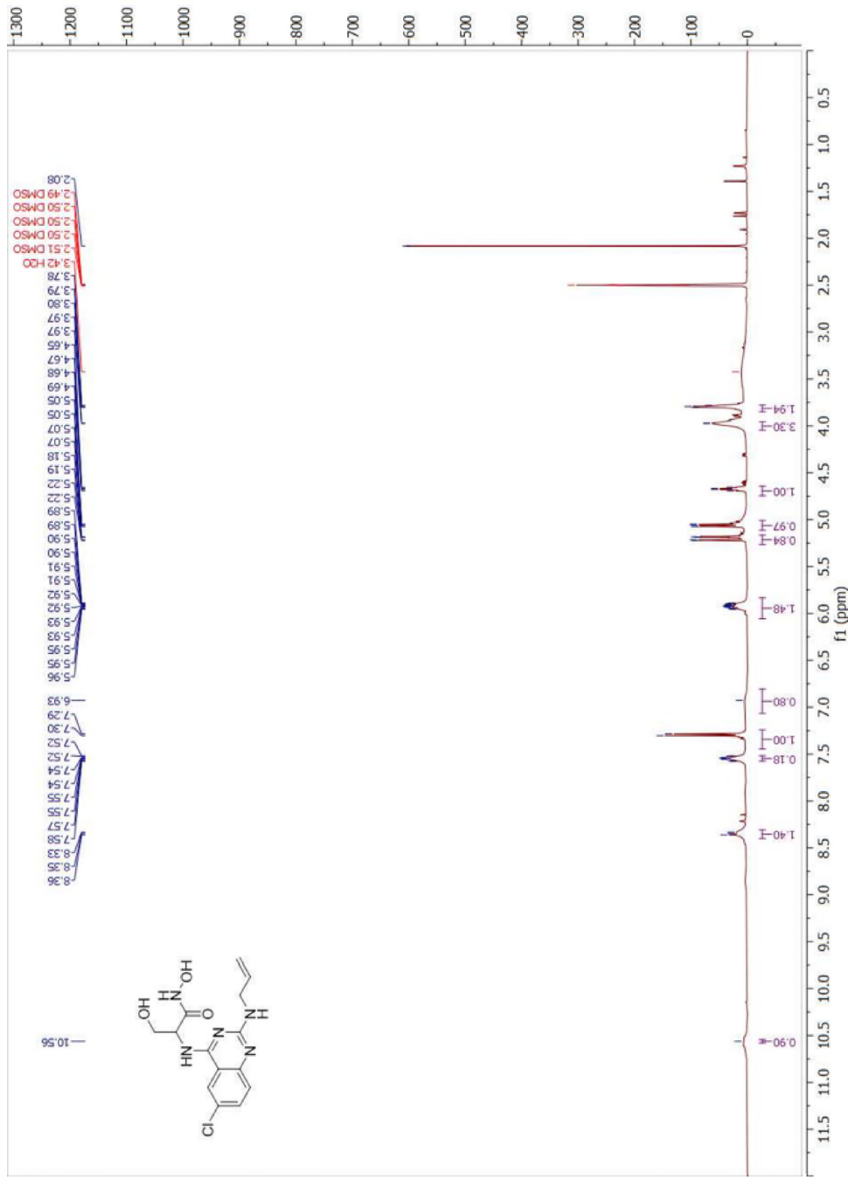

$^{13}C$ NMR

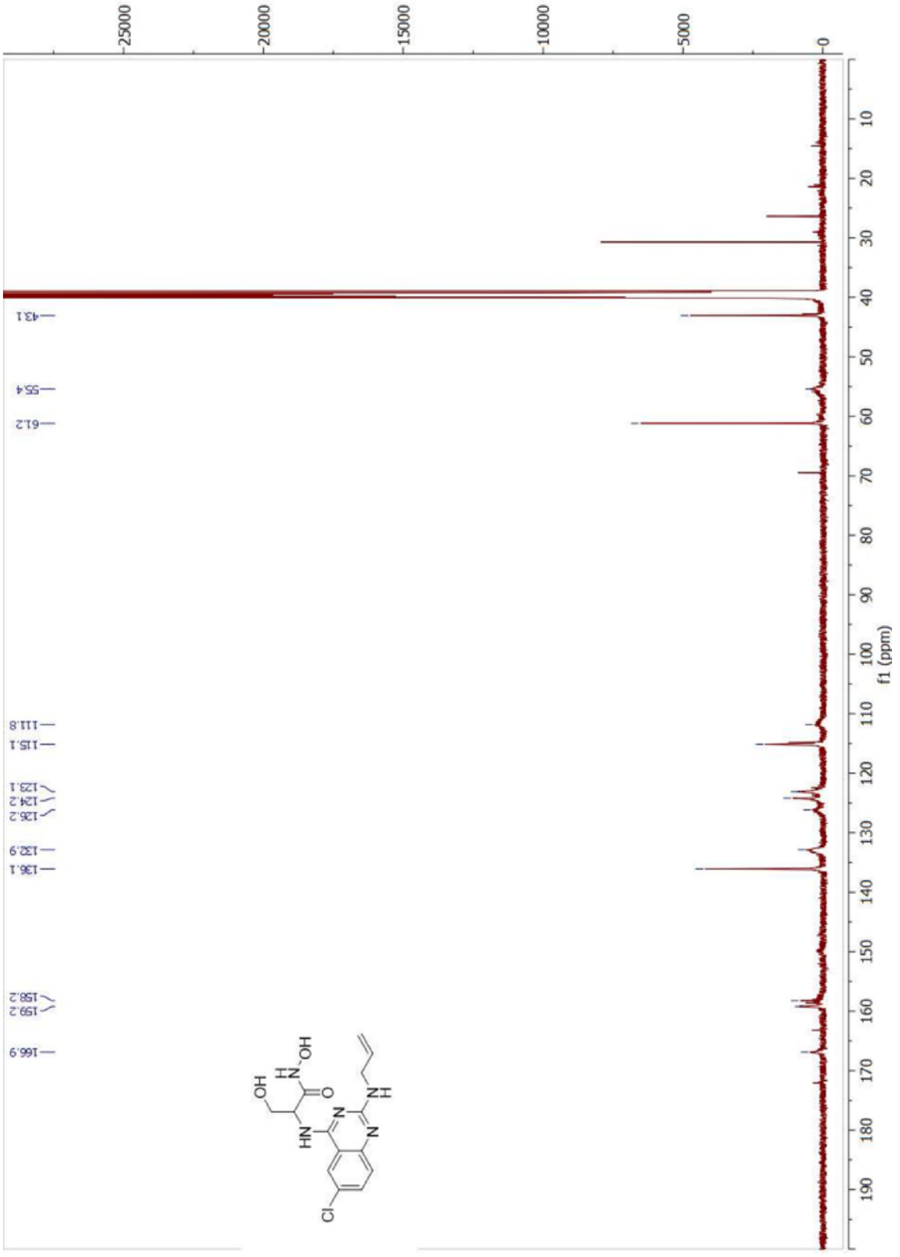
43.1
55.4
61.2
111.8
115.1
123.1
124.2
126.2
132.9
136.1
158.2
159.2
166.9
f1 (ppm)

**Methyl (6-chloro-2-morpholinoquinazolin-4-yl)-*L*-alaninate (45)**

$^{1}$H NMR

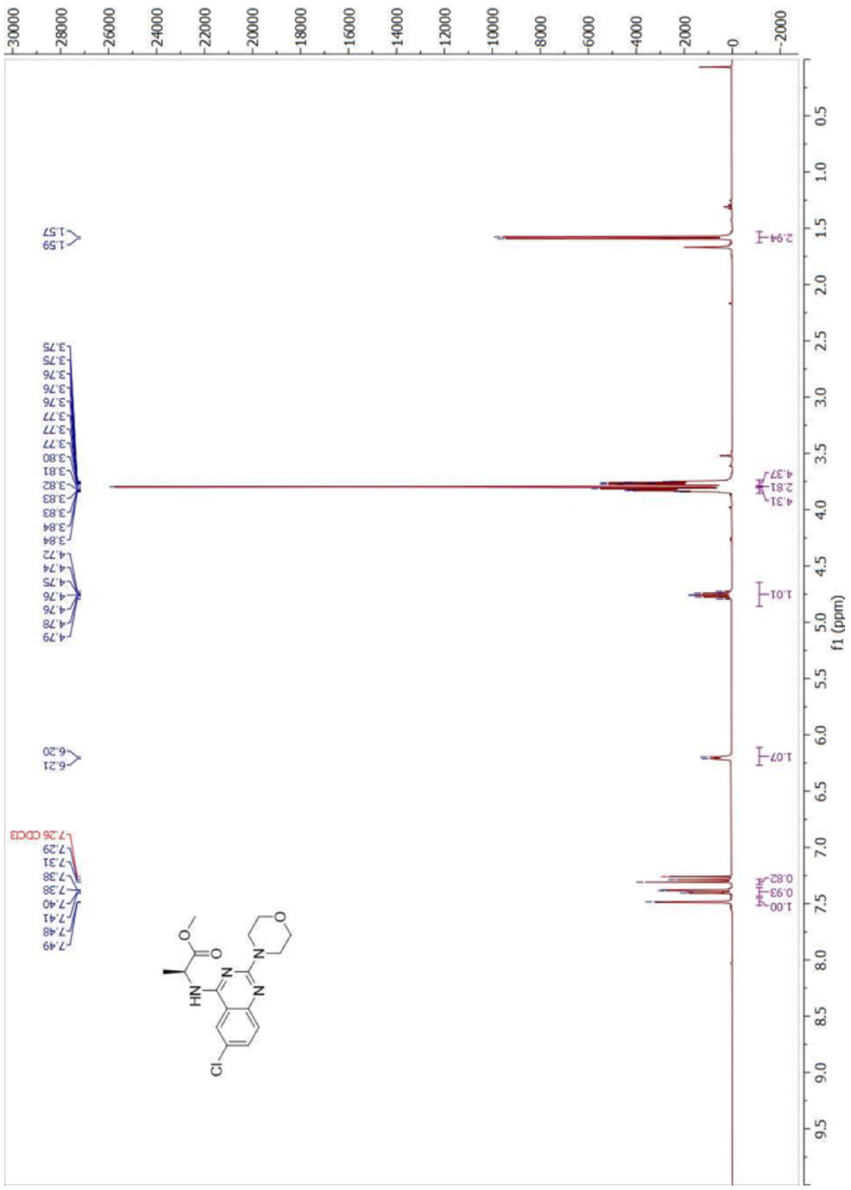

$^{13}C$ NMR

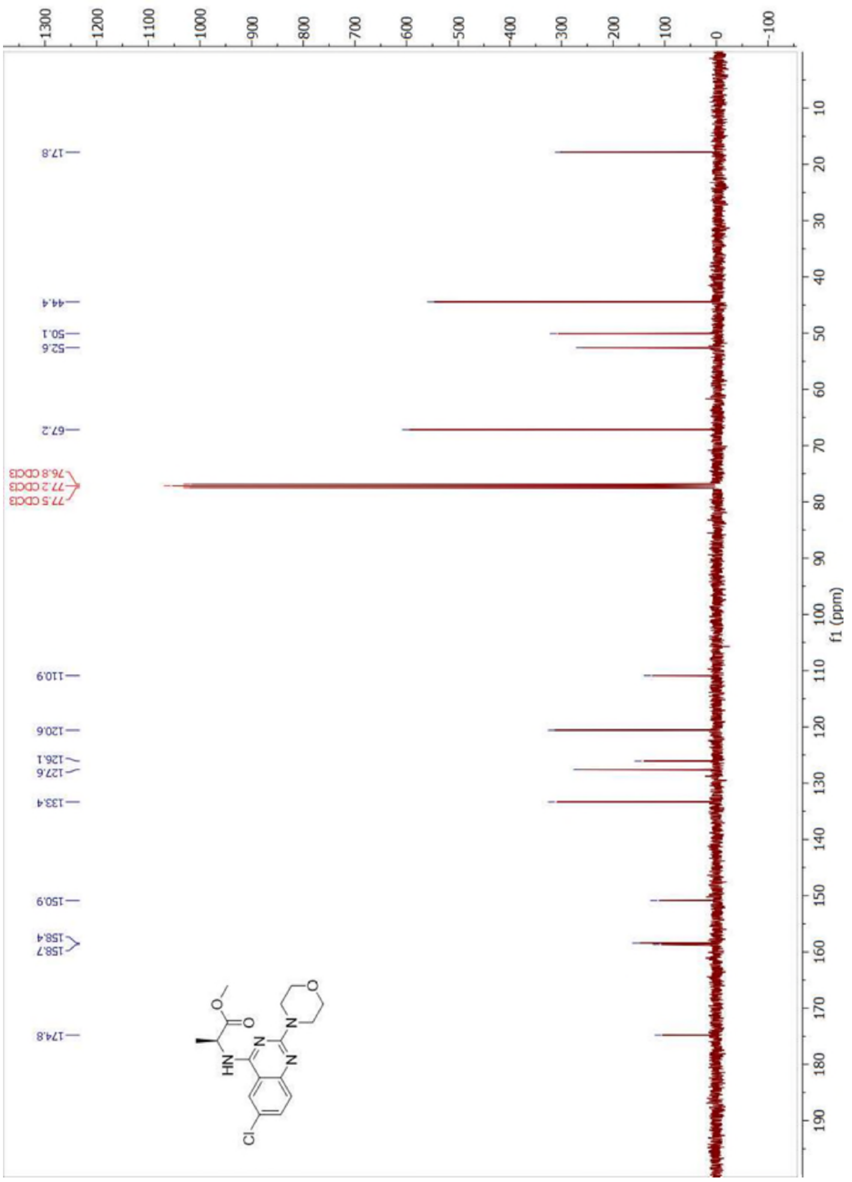
17.8
44.4
50.1
52.6
67.2
76.8 CDCl3
77.2 CDCl3
77.5 CDCl3
110.9
120.6
126.1
127.6
133.4
150.9
158.4
158.7
174.8
f1 (ppm)

**(*S*)-2-(6-Chloro-2-morpholinoquinazolin-4-yl)amino-*N*-hydroxypropanamide (15)**

$^{1}$H NMR

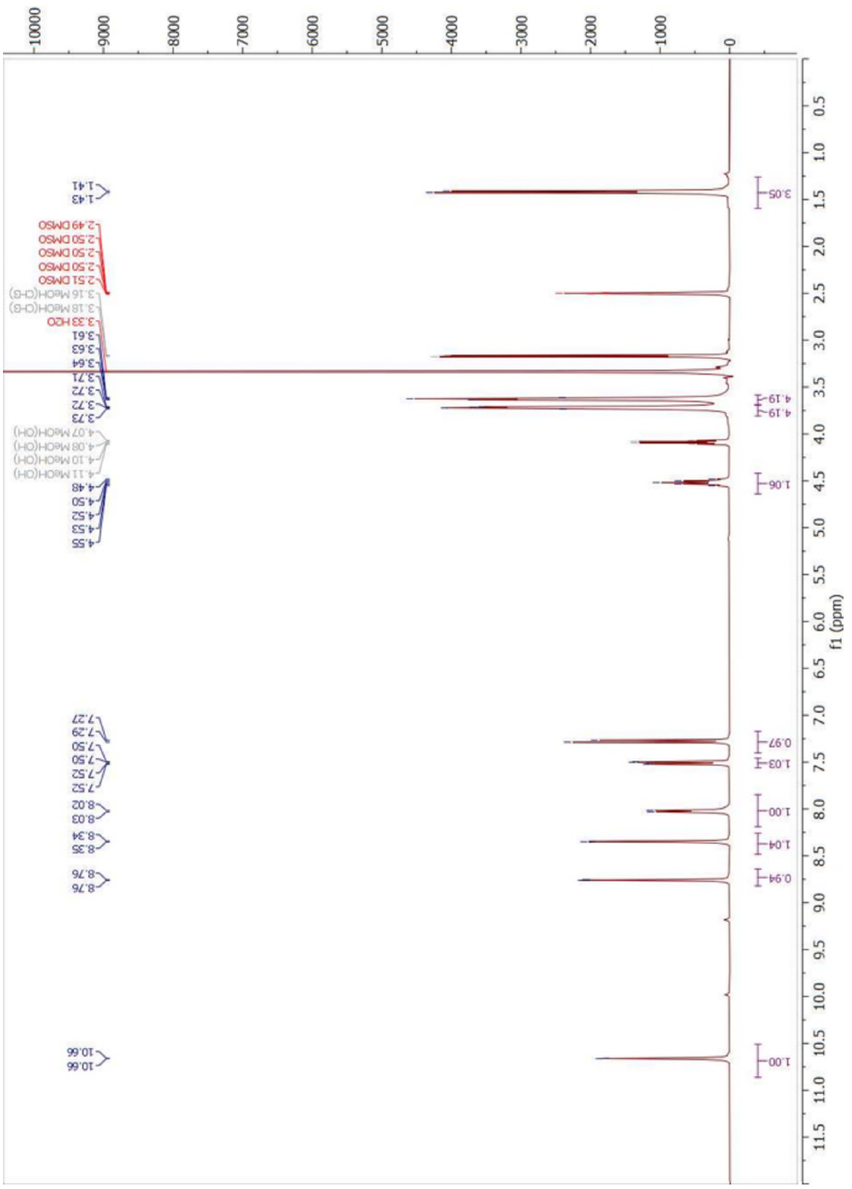

$^{13}$C NMR

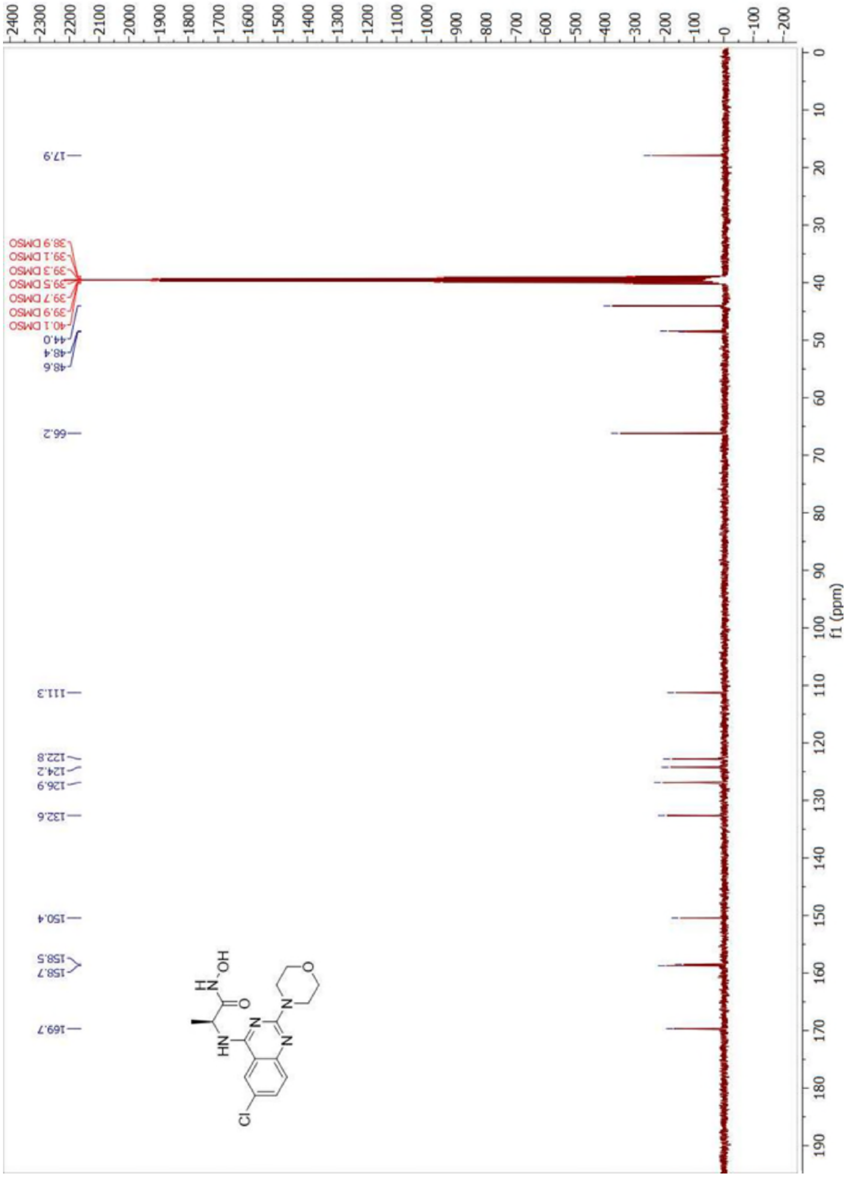

17.9
38.9 DMSO
39.1 DMSO
39.3 DMSO
39.5 DMSO
39.7 DMSO
39.9 DMSO
40.1 DMSO
44.0
48.4
48.6
66.2
111.3
122.8
124.2
126.9
132.6
150.4
158.5
158.7
169.7
f1 (ppm)

**Methyl (6-chloro-2-benzylaminoquinazolin-4-yl)-*L*-alaninate (46)**

$^{13}$C NMR

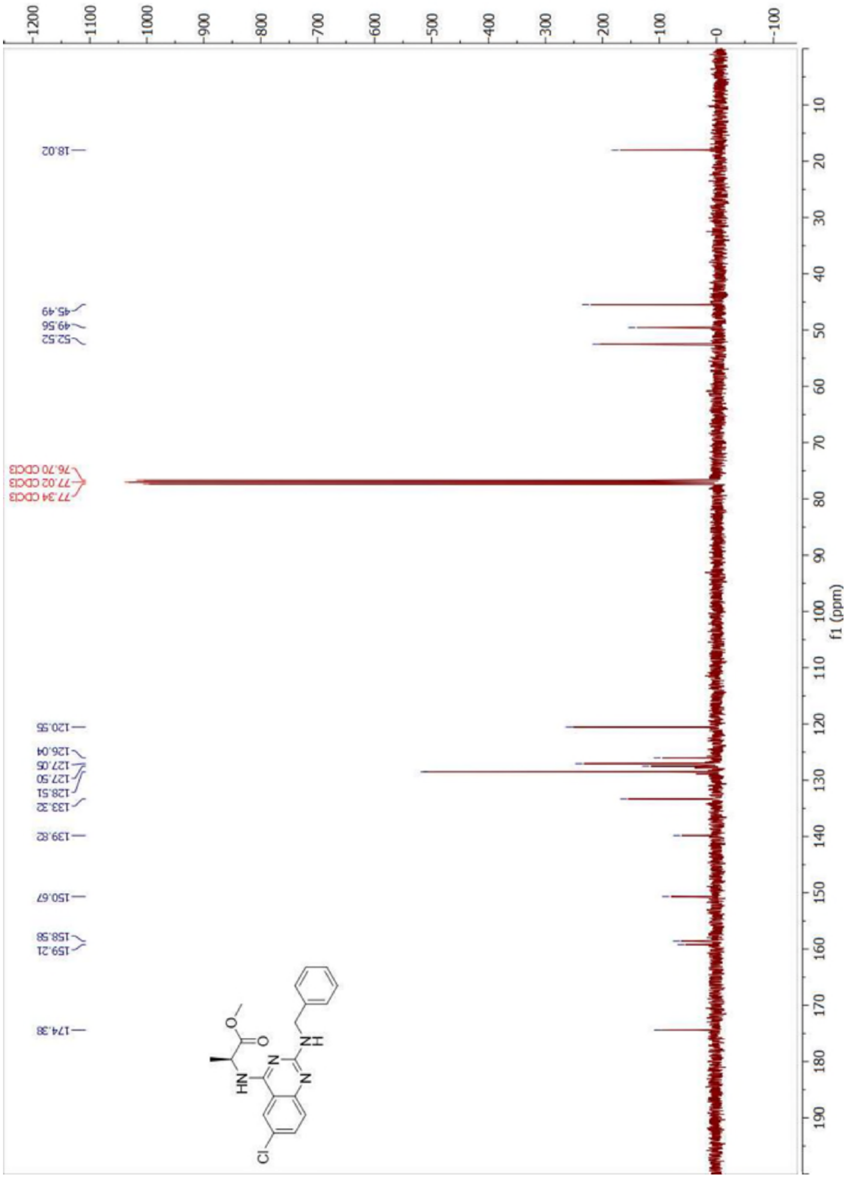

**(*S*)-2-(6-Chloro-2-benzylaminoquinazolin-4-yl)amino-*N*-hydroxypropanamide (16)**

$^{1}$H NMR

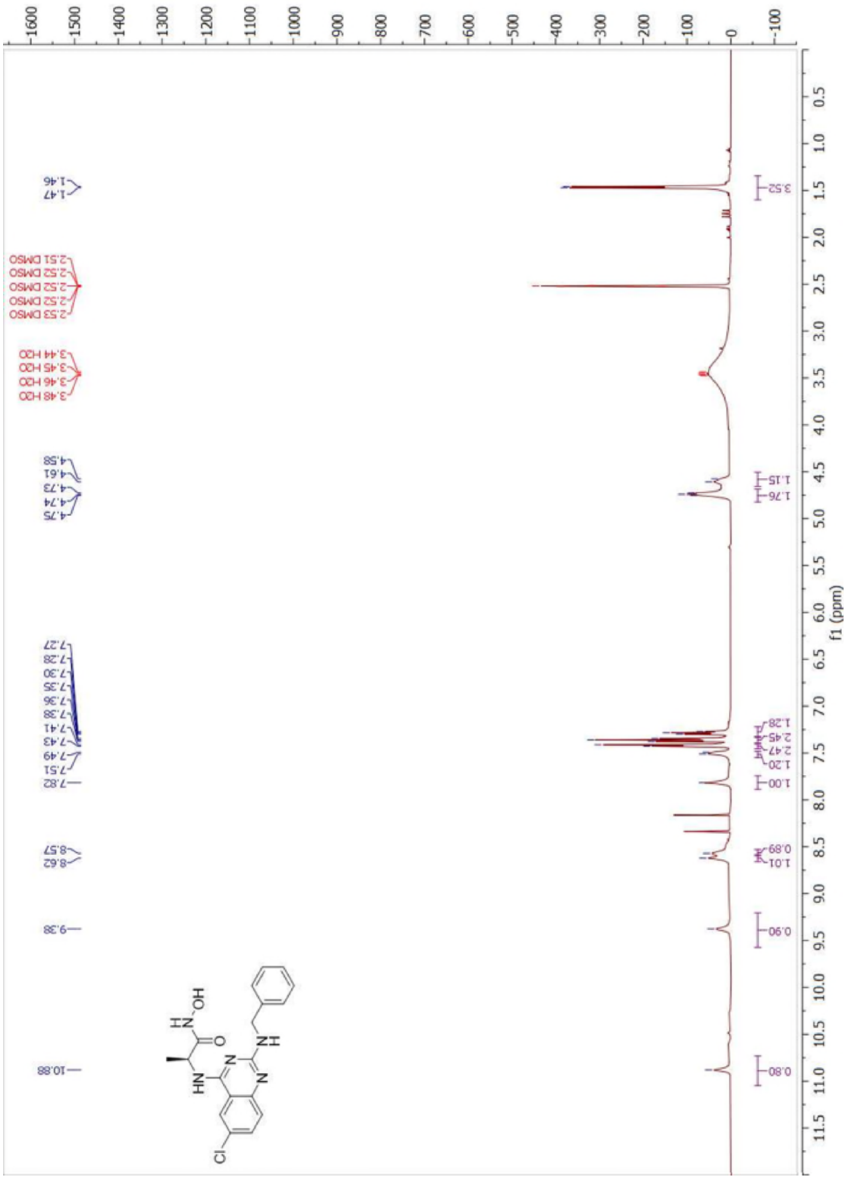

$^{13}C$ NMR

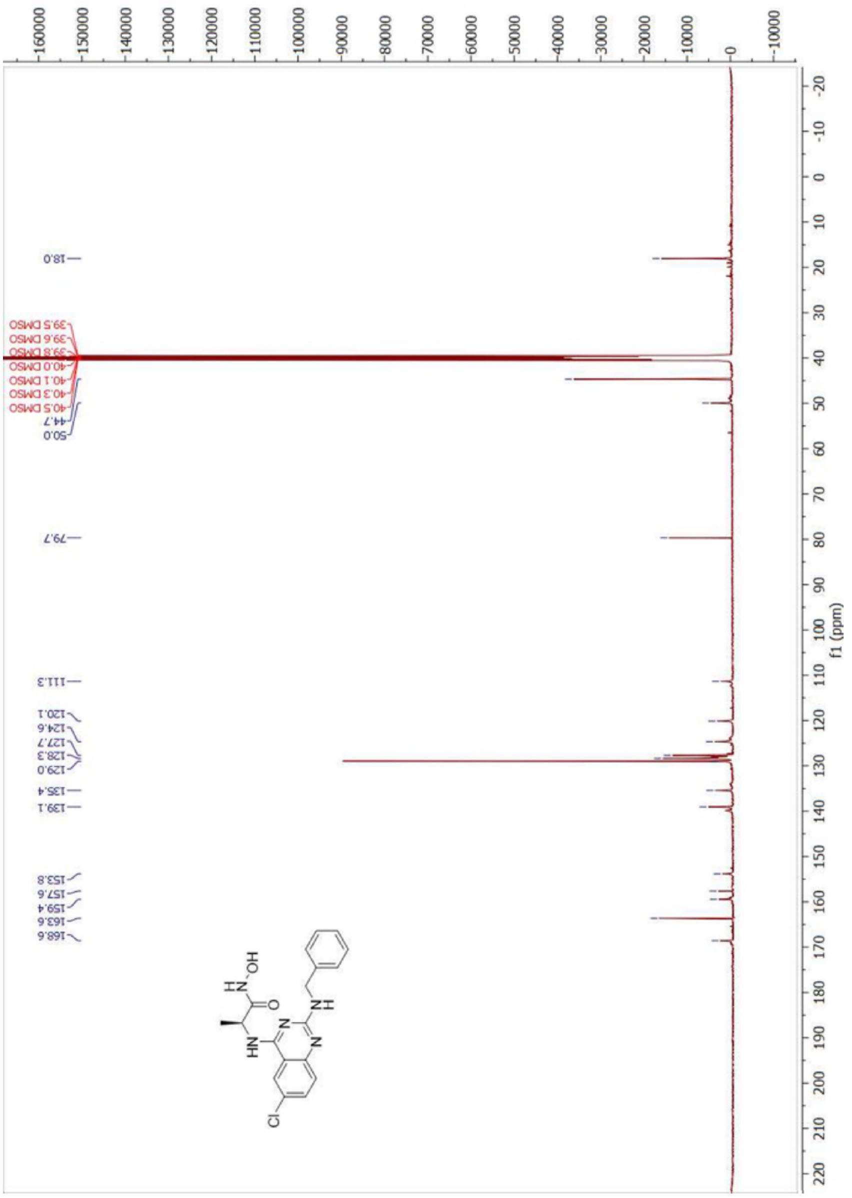

**Methyl (6-chloro-2-methyl(propyl)aminoquinazolin-4-yl)-*L*-alaninate (47)**

$^1$H NMR

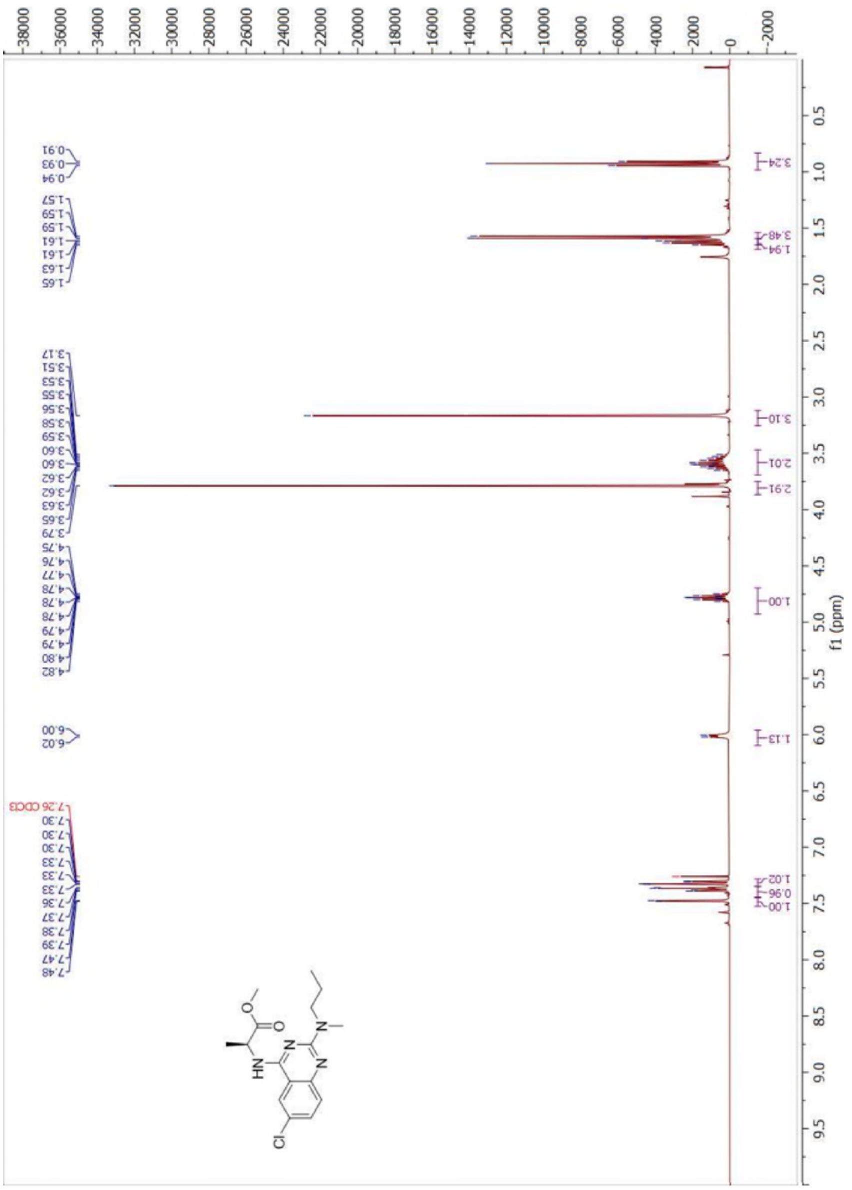

$^{13}$C NMR

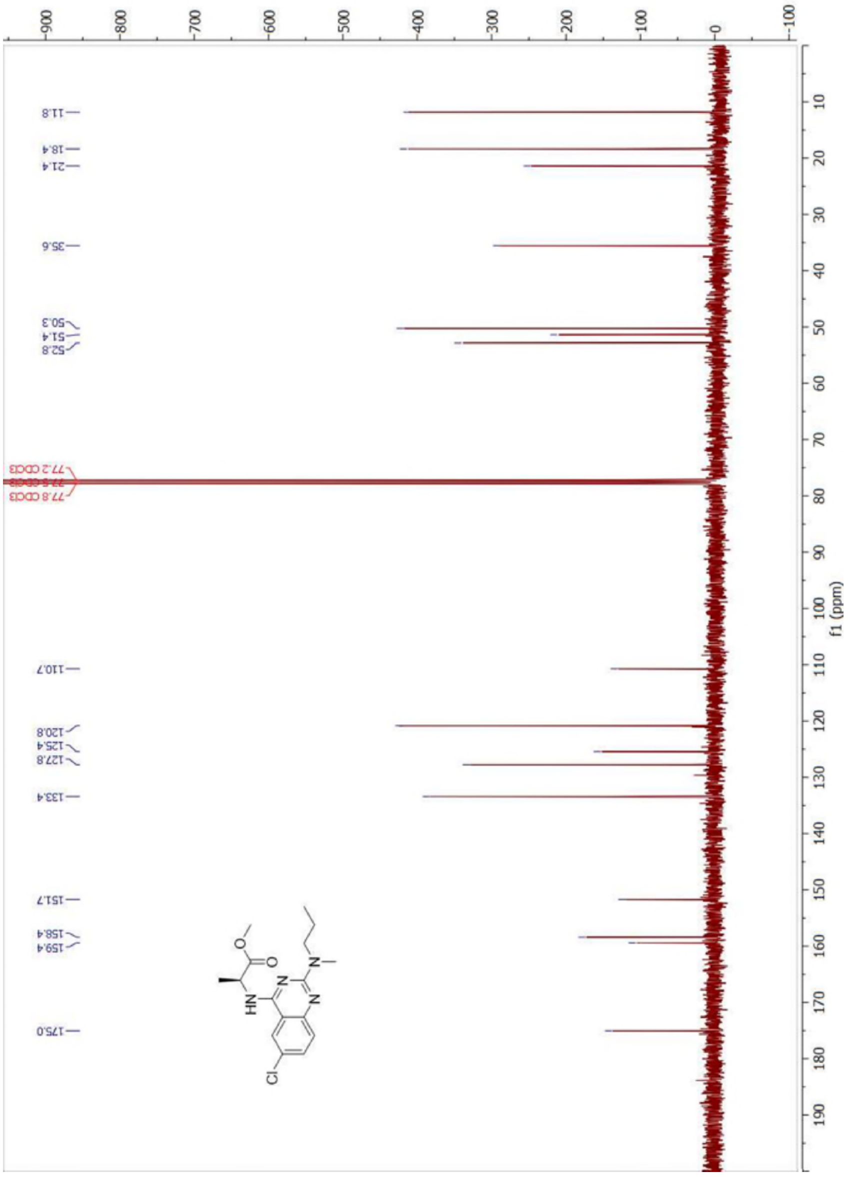
11.8
18.4
21.4
35.6
50.3
51.4
52.8
77.2 CDCl3
77.5 CDCl3
77.8 CDCl3
110.7
120.8
125.4
127.8
133.4
151.7
158.4
159.4
175.0
f1 (ppm)

**(*S*)-2-(6-chloro-2-methyl(propyl)aminoquinazolin-4-yl)amino-*N*-hydroxypropanamide (17)**

$^1$H NMR

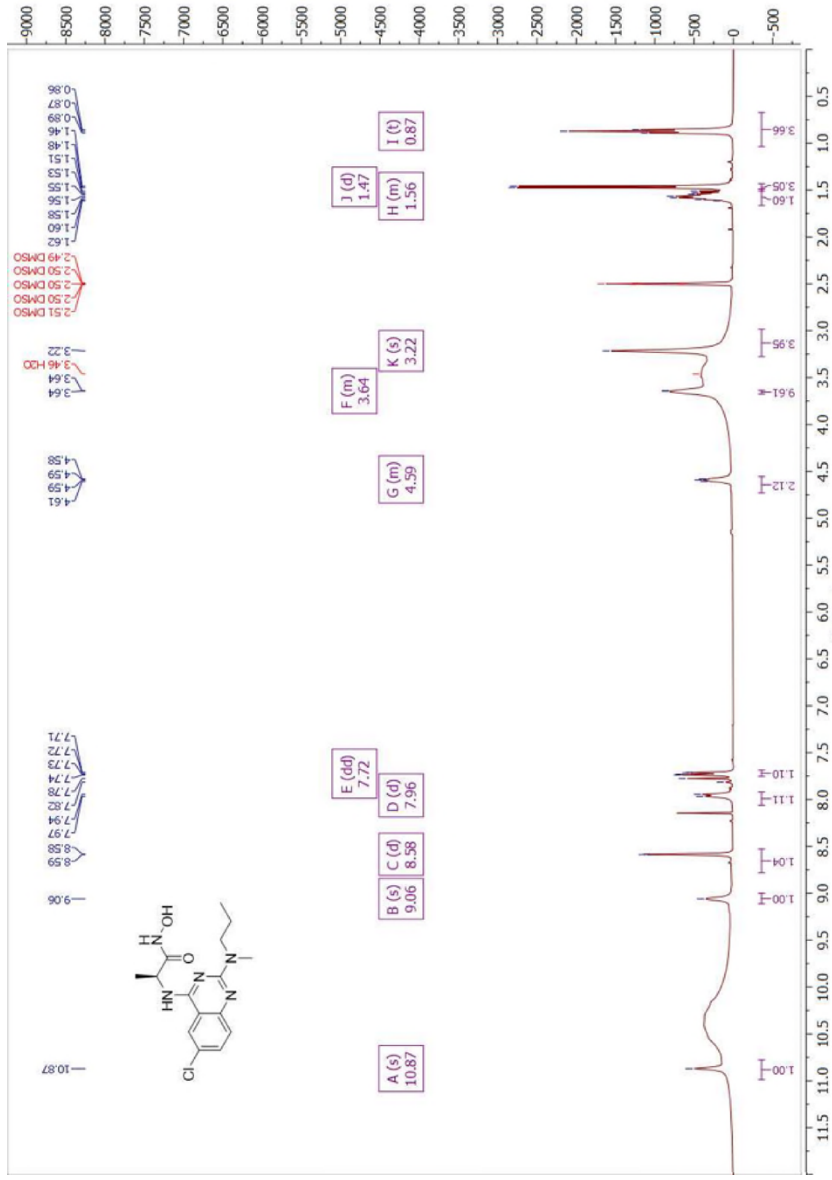

$^{13}C$ NMR

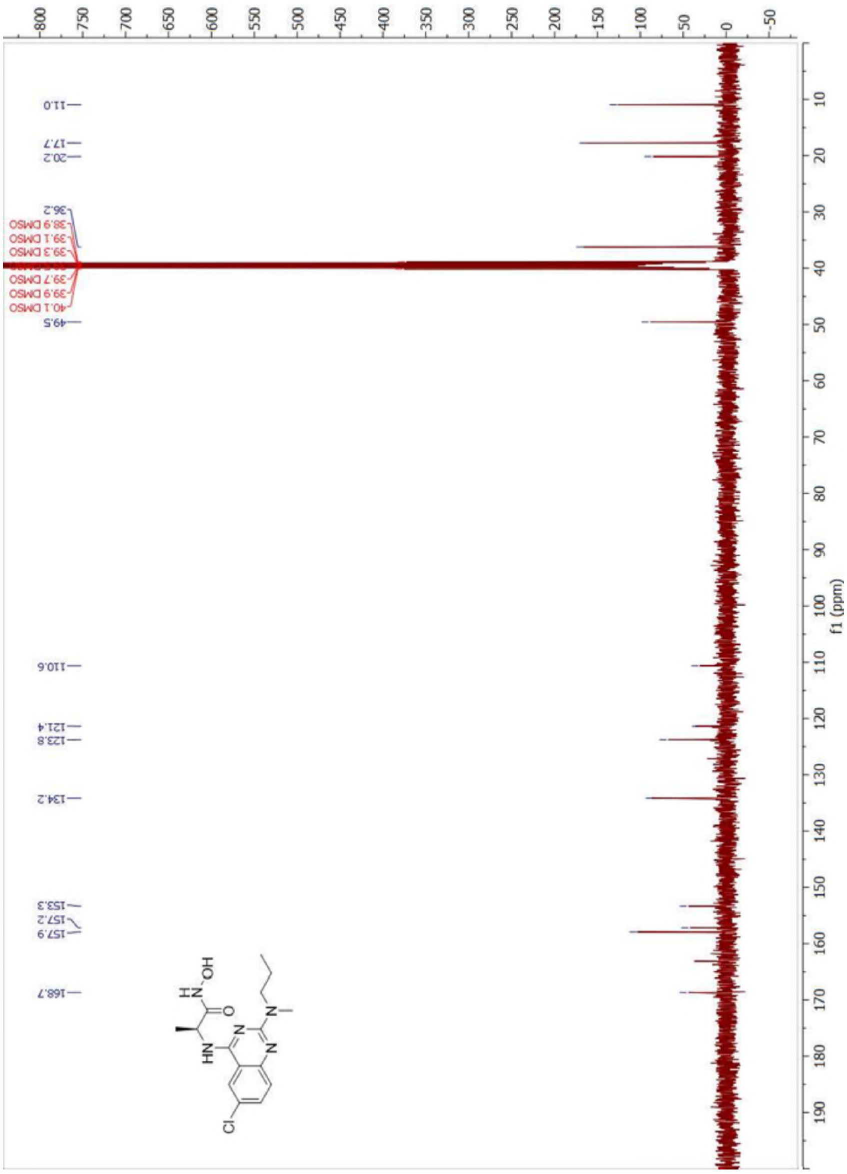

**Methyl (6-chloro-2-diethylaminoquinazolin-4-yl)-*L*-alaninate (48)**

$^{1}$H NMR

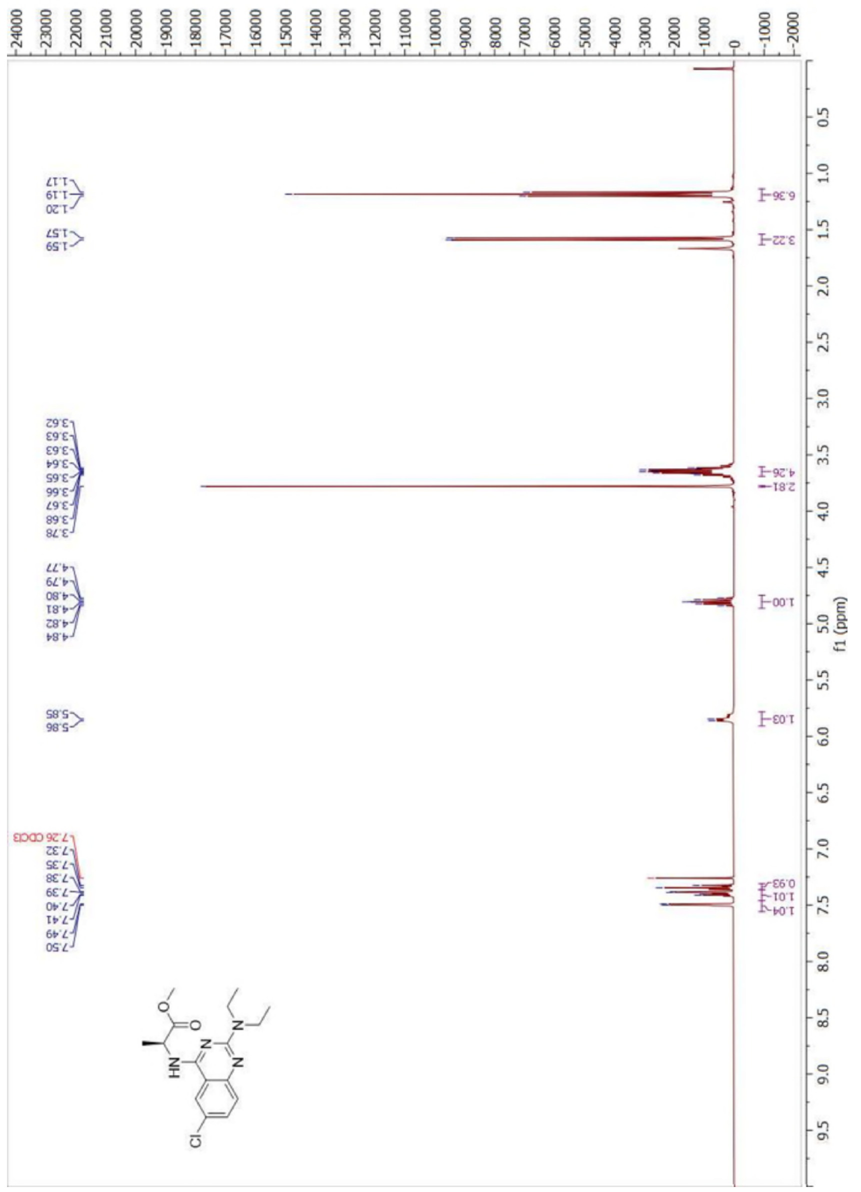

$^{13}C$ NMR

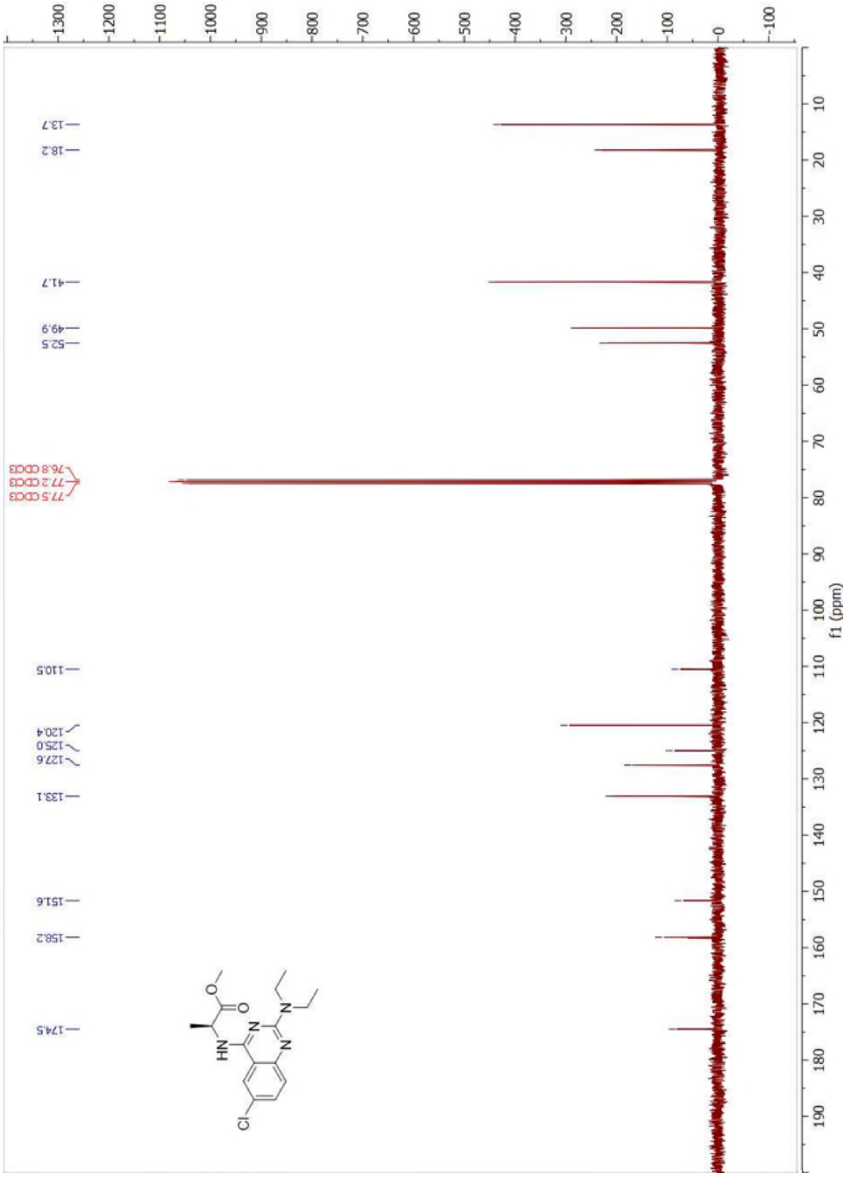

13.7
18.2
41.7
49.9
52.5
76.8 CDCl3
77.2 CDCl3
77.5 CDCl3
110.5
120.4
125.0
127.6
133.1
151.6
158.2
174.5
f1 (ppm)

**Methyl (6-chloro-2-(furan-2-ylmethyl)aminoquinazolin-4-yl-*L*-alaninate (49)**

$^{1}$H NMR

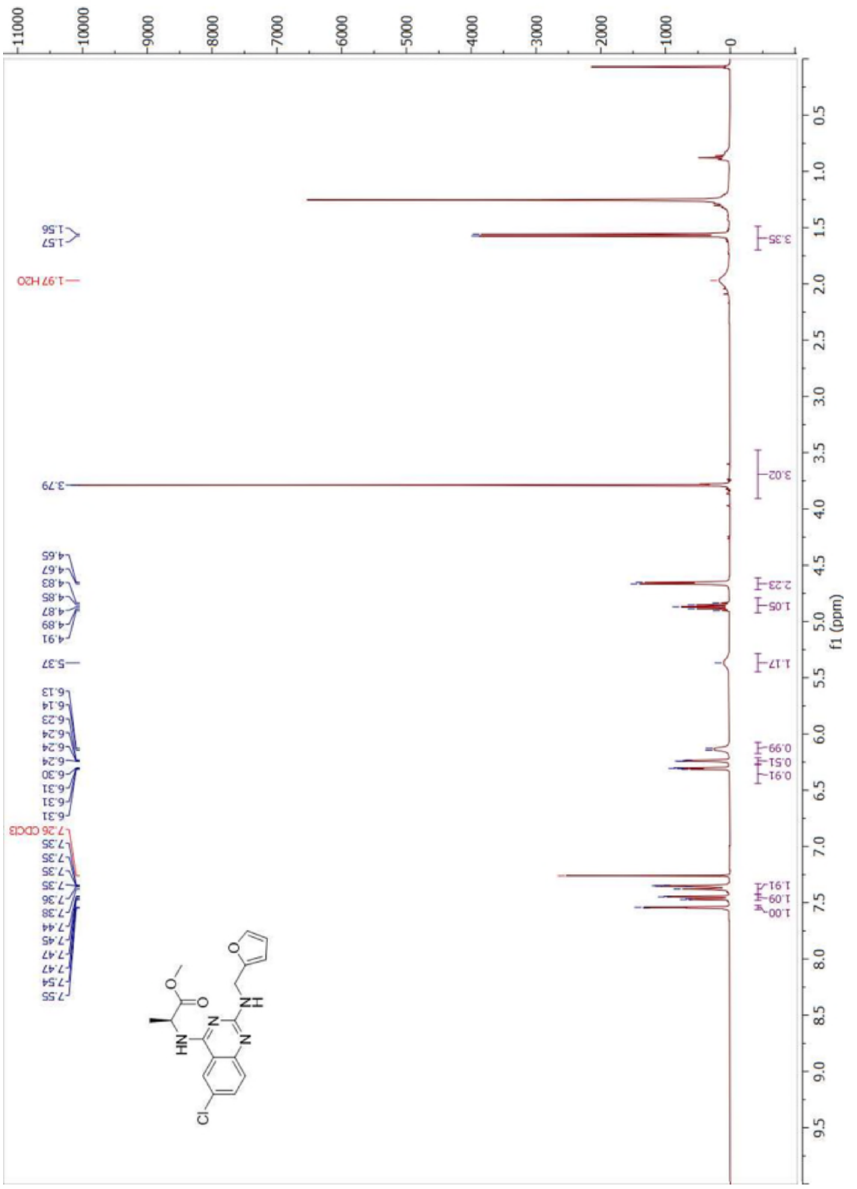

$^{13}C$ NMR

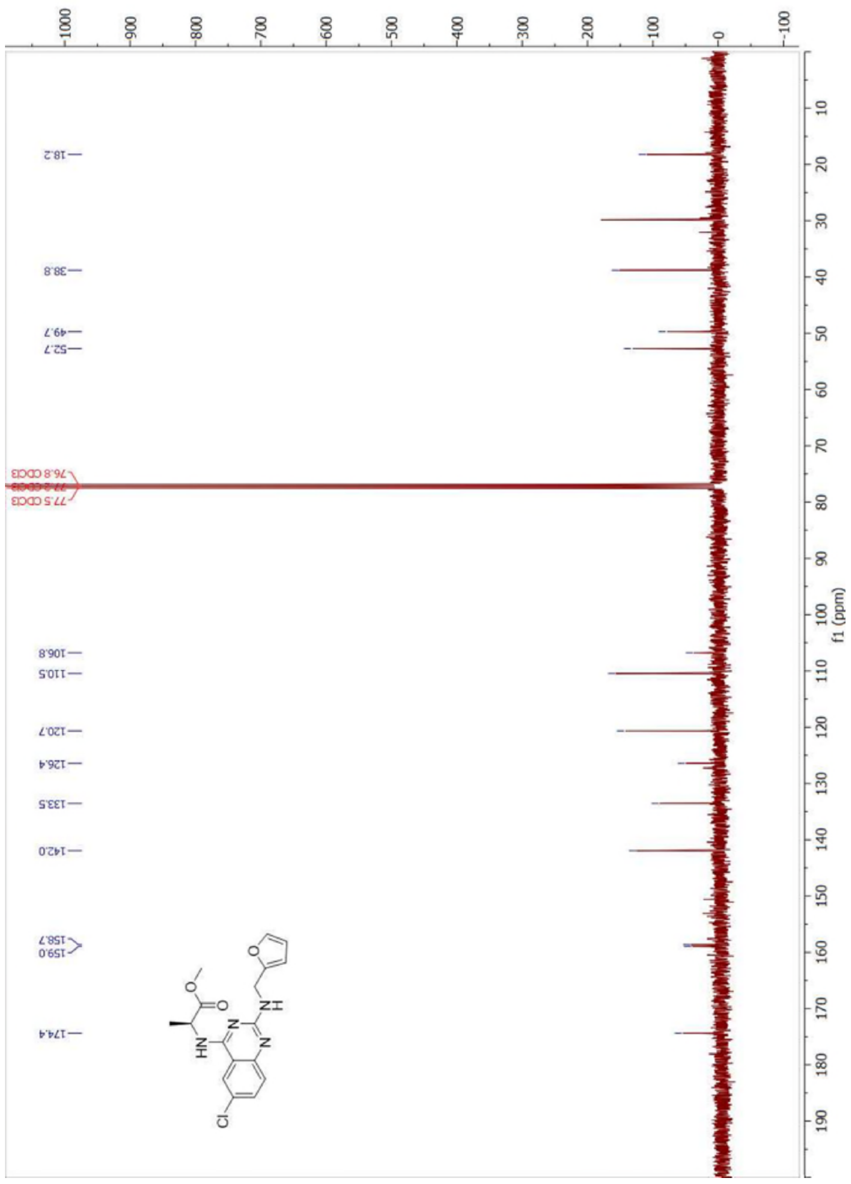

**(*S*)-2-(6-Chloro-2-(furan-2-ylmethyl)aminoquinazolin-4-yl)amino-*N*- hydroxypropanamide (19)**

$^{1}$H NMR

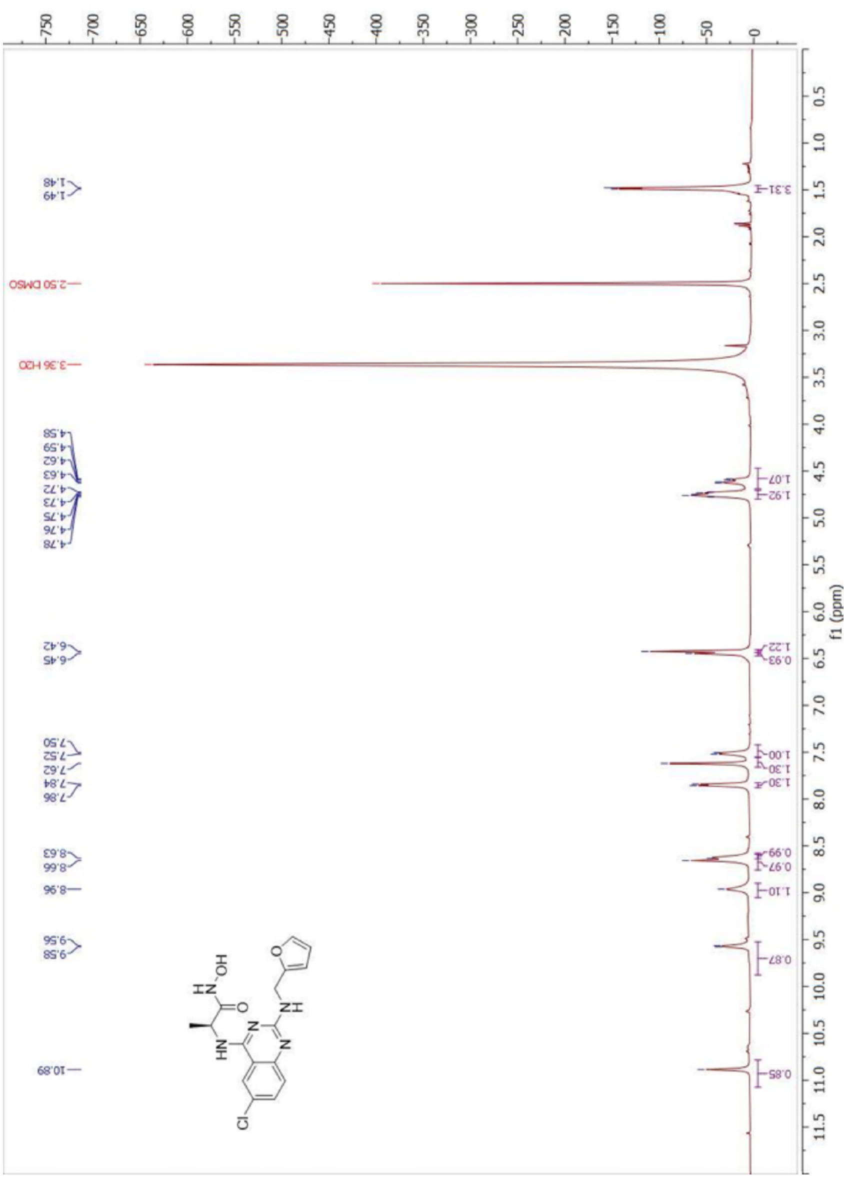

$^{13}C$ NMR

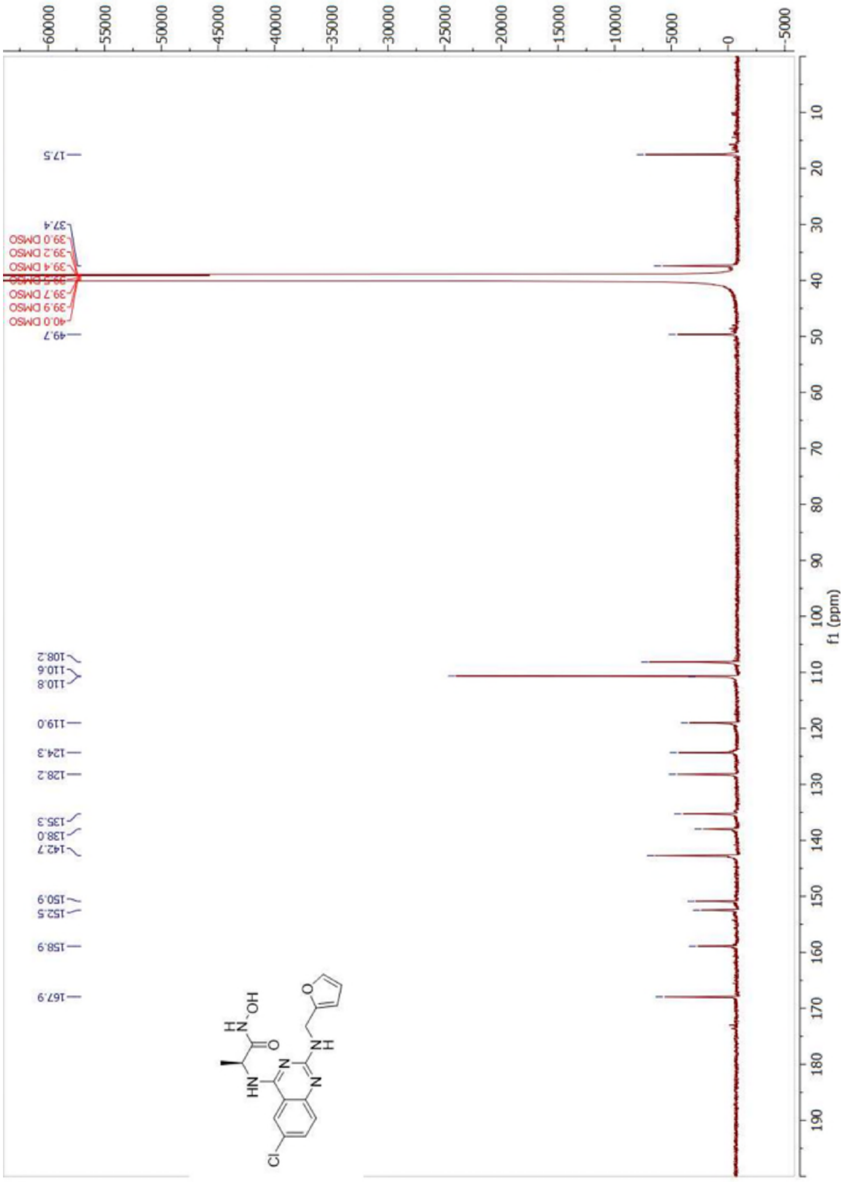

17.5
37.4
39.0 DMSO
39.2 DMSO
39.4 DMSO
39.7 DMSO
39.9 DMSO
40.0 DMSO
49.7
108.2
110.6
110.8
119.0
124.3
128.2
135.3
138.0
142.7
150.9
152.5
158.9
167.9
f1 (ppm)

**Ethyl 4-oxo-3,4-dihydroquinazoline-2-carboxylate (50)**

$^1$H NMR

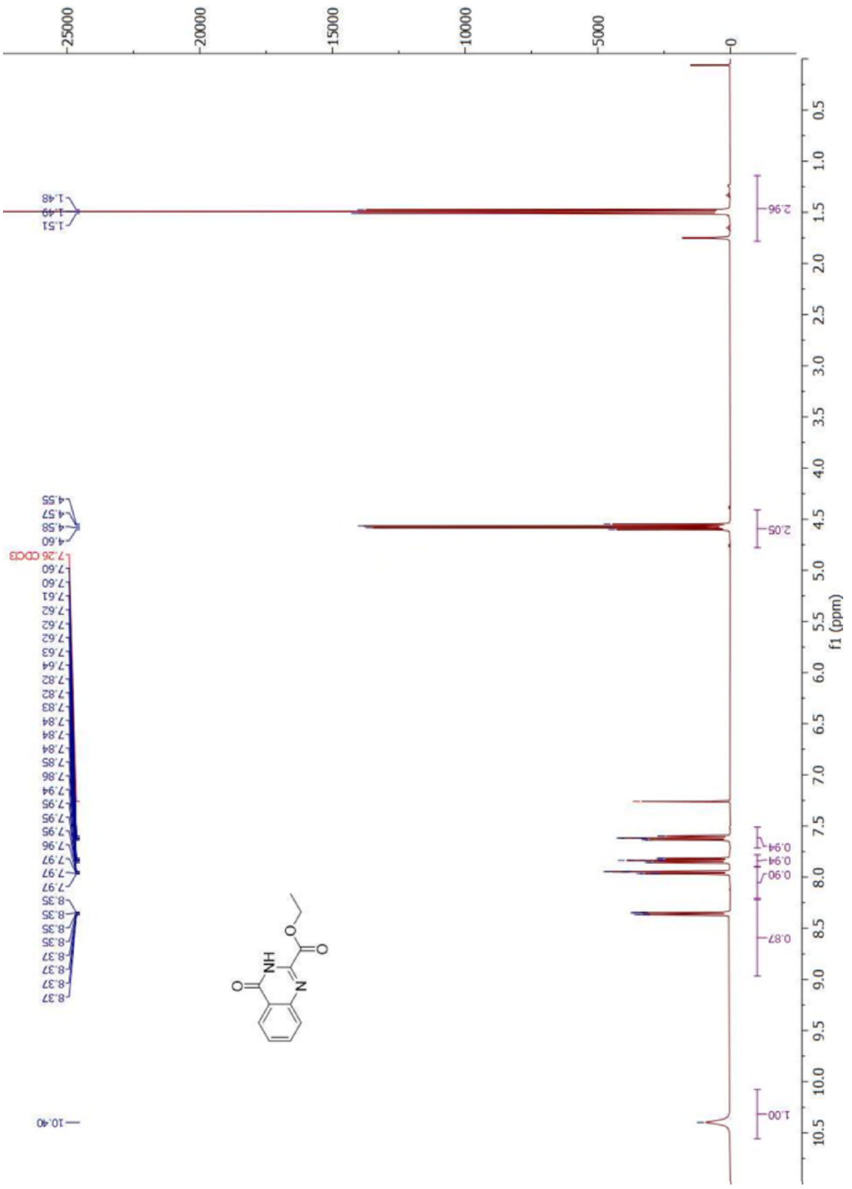

$^{13}C$ NMR

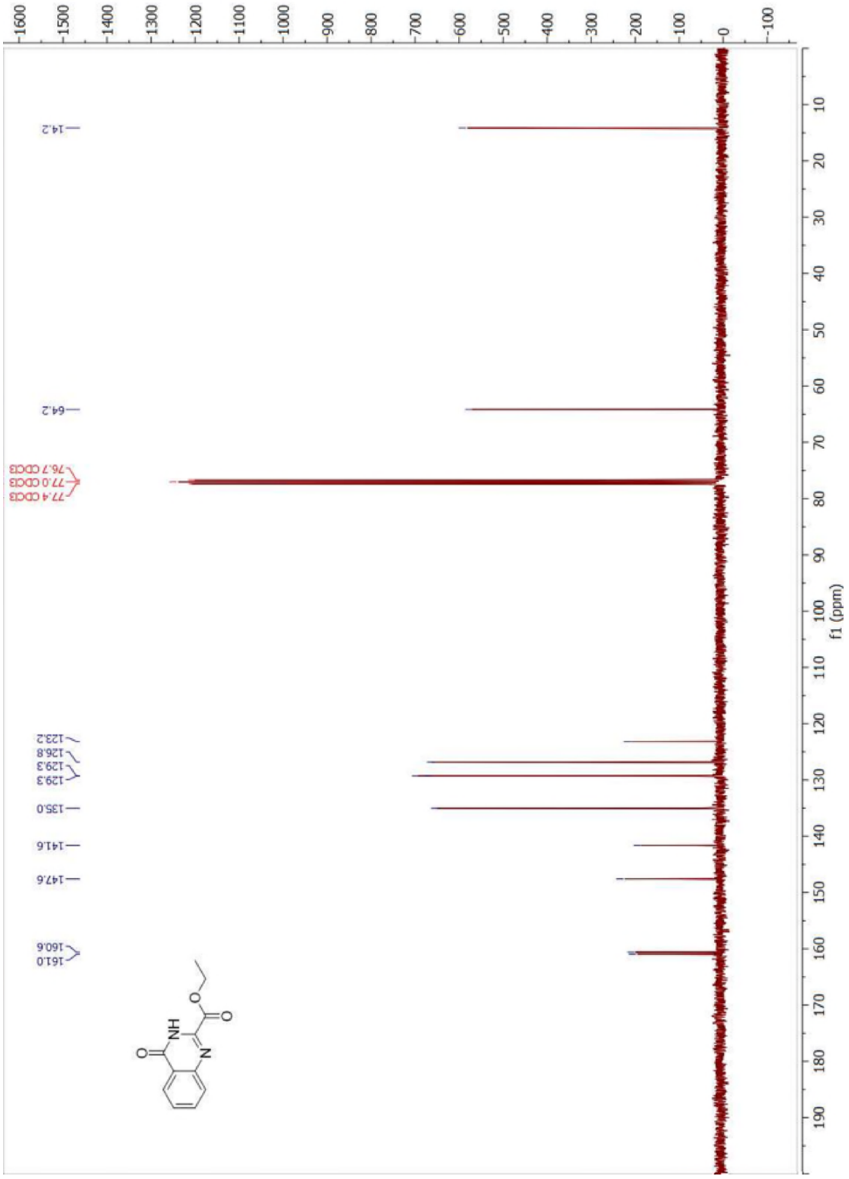

14.2
64.2
76.7 CDCl3
77.0 CDCl3
77.4 CDCl3
123.2
126.8
129.3
129.3
135.0
141.6
147.6
160.6
161.0
f1 (ppm)

**(*S*)-*N*-Allyl-4-(1-hydroxyamino-1-oxopropan-2-yl)aminoquinazoline-2-carboxamide (20)**

$^{1}$H NMR

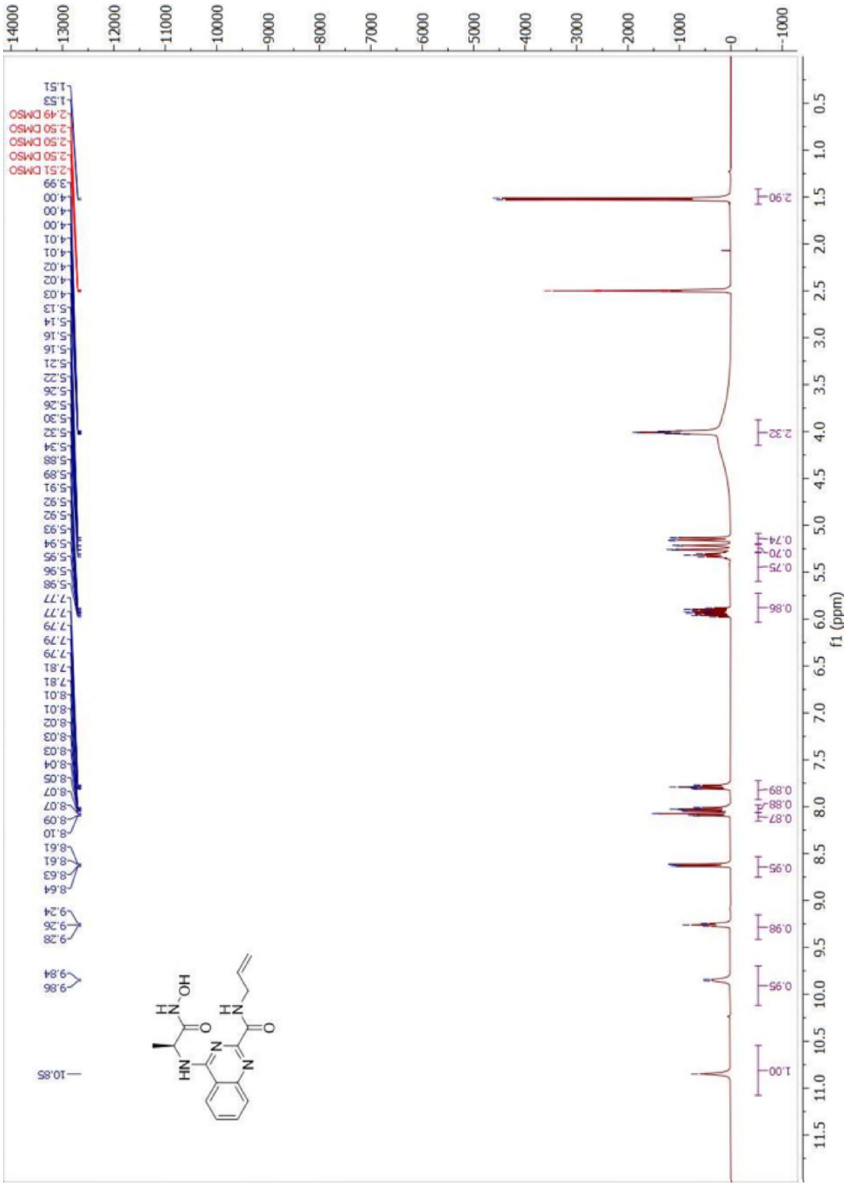

$^{13}C$ NMR

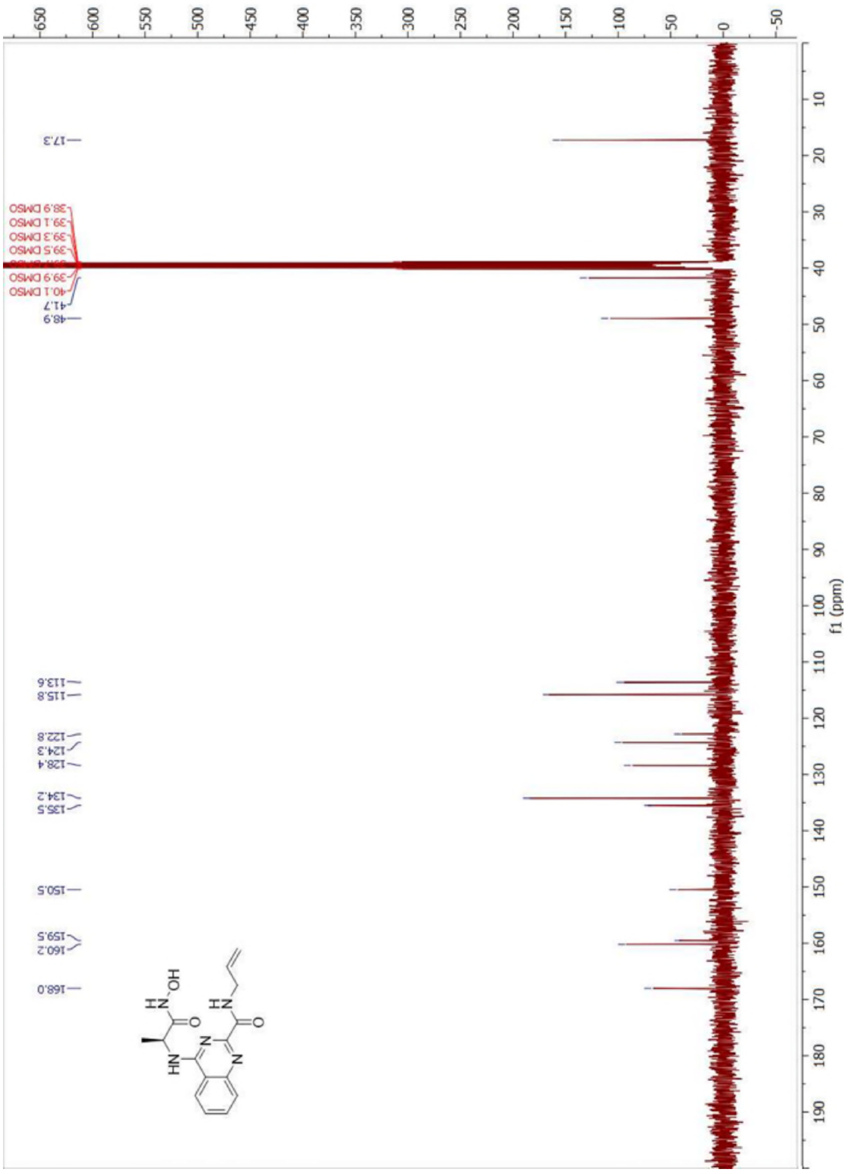

17.3
38.9 DMSO
39.1 DMSO
39.3 DMSO
39.5 DMSO
39.9 DMSO
40.1 DMSO
41.7
48.9
113.6
115.8
122.8
124.3
128.4
134.2
135.5
150.5
159.5
160.2
168.0
f1 (ppm)

**Ethyl (6-chloro-2-ethoxyquinazolin-4-yl)-*L*-alaninate (53)**

$^{1}$H NMR

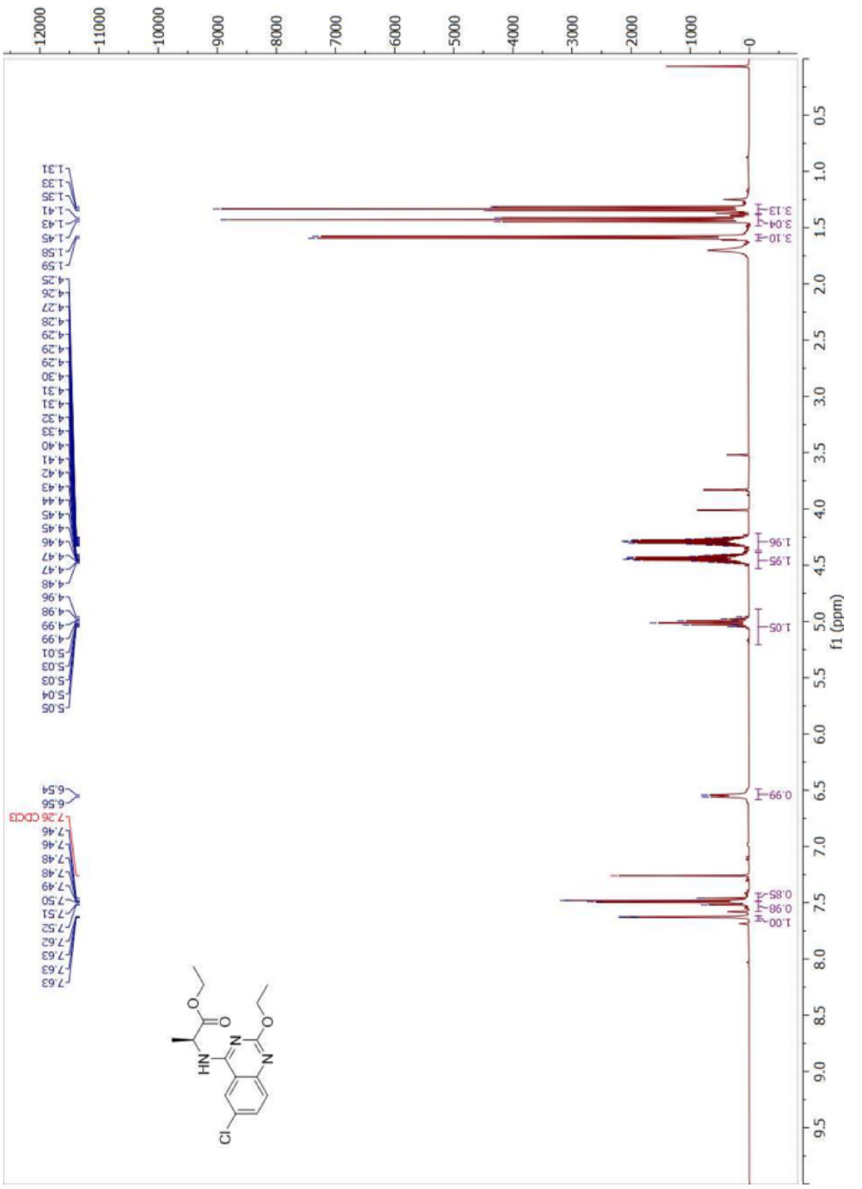

$^{13}C$ NMR

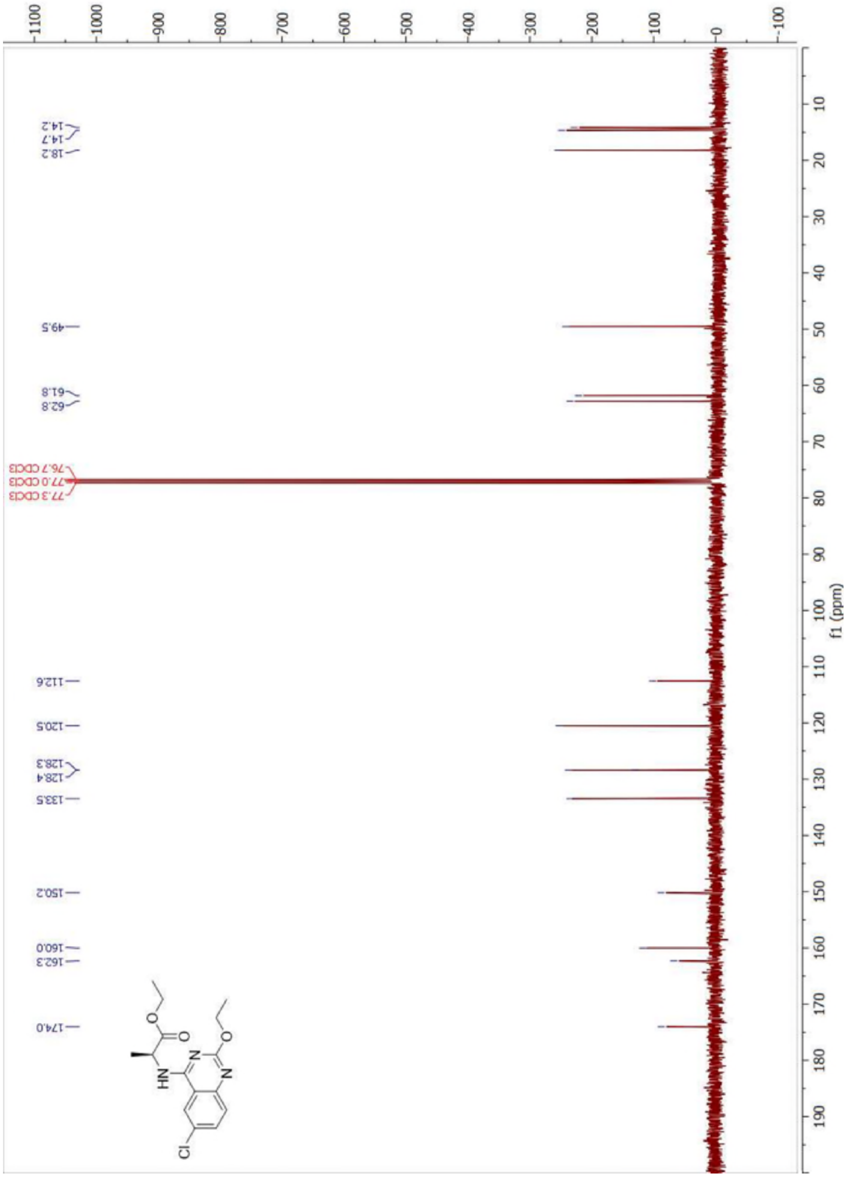
14.2
14.7
18.2
49.5
61.8
62.8
76.7 CDCl3
77.0 CDCl3
77.3 CDCl3
112.6
120.5
128.3
128.4
133.5
150.2
160.0
162.3
174.0
f1 (ppm)

**(*S*)-2-(6-Chloro-2-ethoxyquinazolin-4-yl)amino-*N*-hydroxypropanamide (21)**

$^1$H NMR

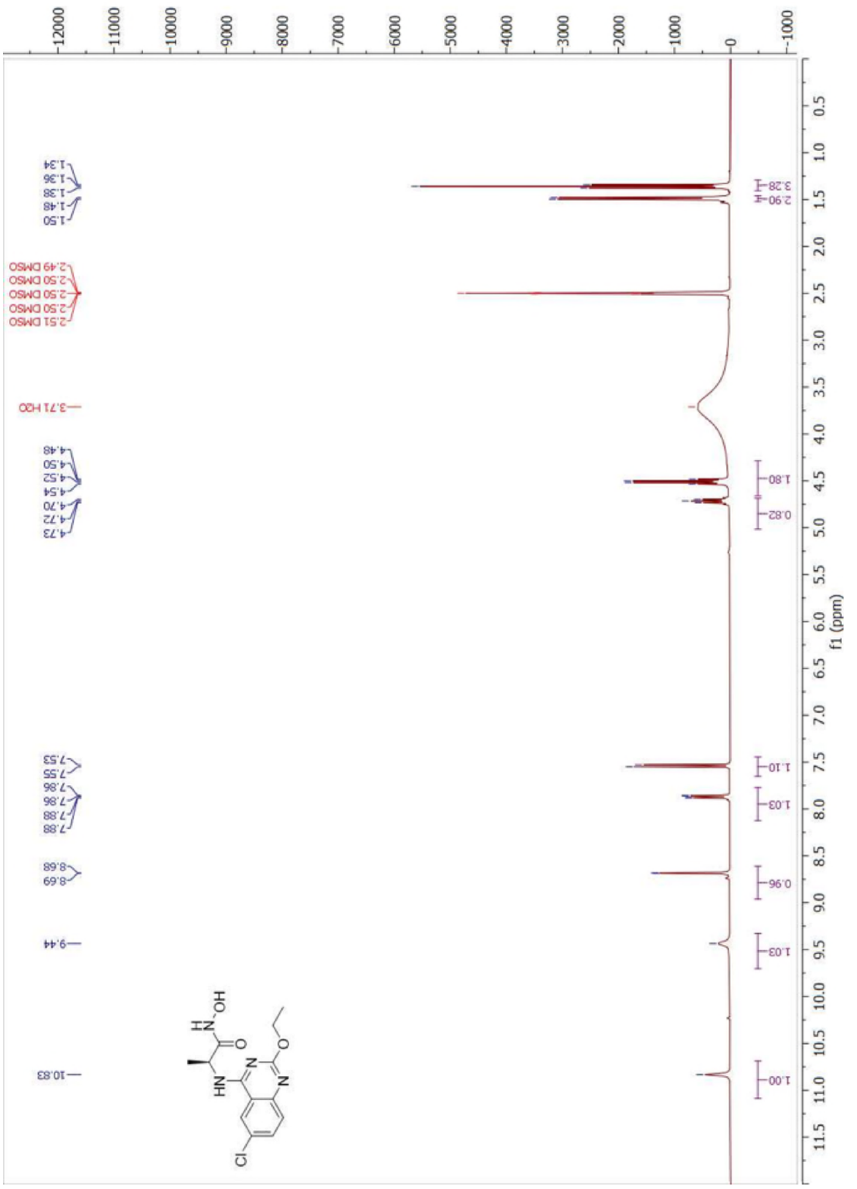

$^{13}C$ NMR

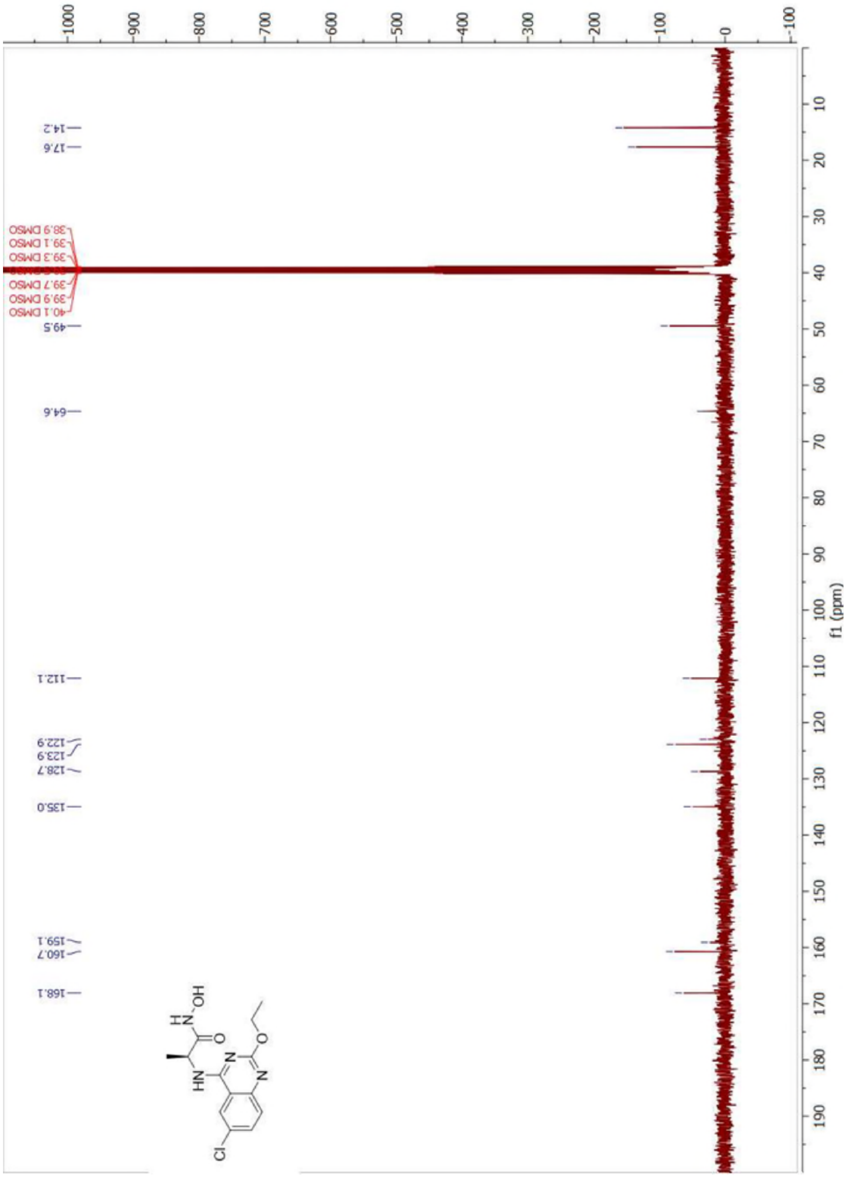
14.2
17.6
38.9 DMSO
39.1 DMSO
39.3 DMSO
39.7 DMSO
39.9 DMSO
40.1 DMSO
49.5
64.6
112.1
122.9
123.9
128.7
135.0
159.1
160.7
168.1
f1 (ppm)

**Methyl (6-chloro-2-dimethylaminoquinazolin-4-yl)-*L*-alaninate (54)**

$^{1}$H NMR

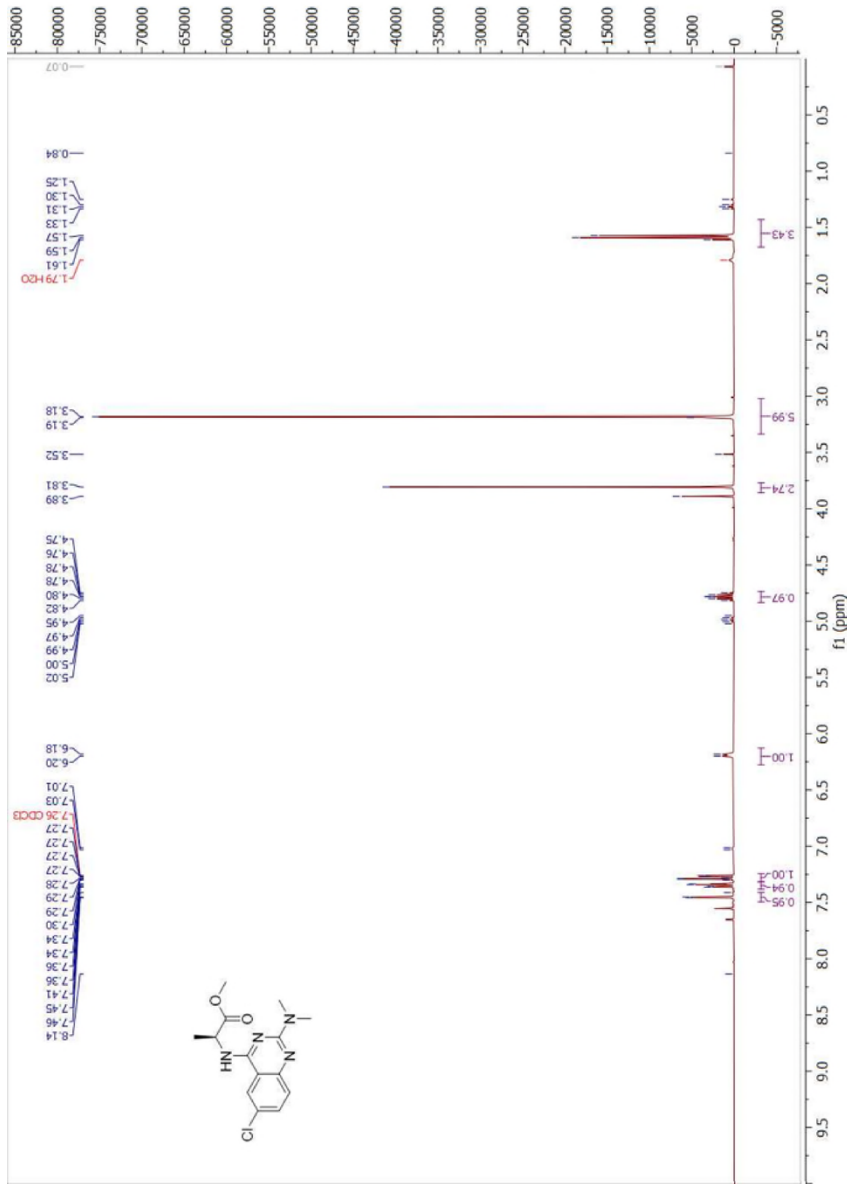

$^{13}C$ NMR

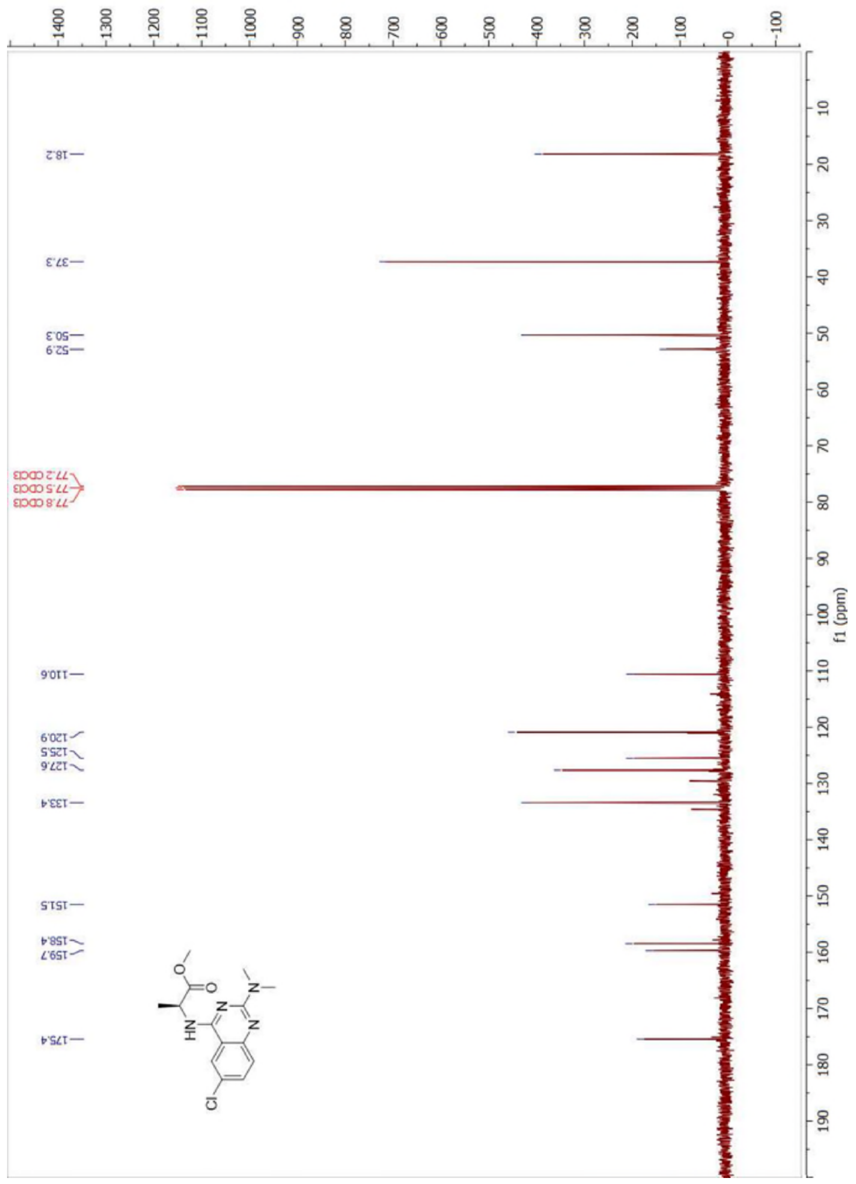

**(*S*)-2-(6-Chloro-2-dimethylaminoquinazolin-4-yl)amino-*N*-hydroxypropanamide (22)**

$^{1}$H NMR

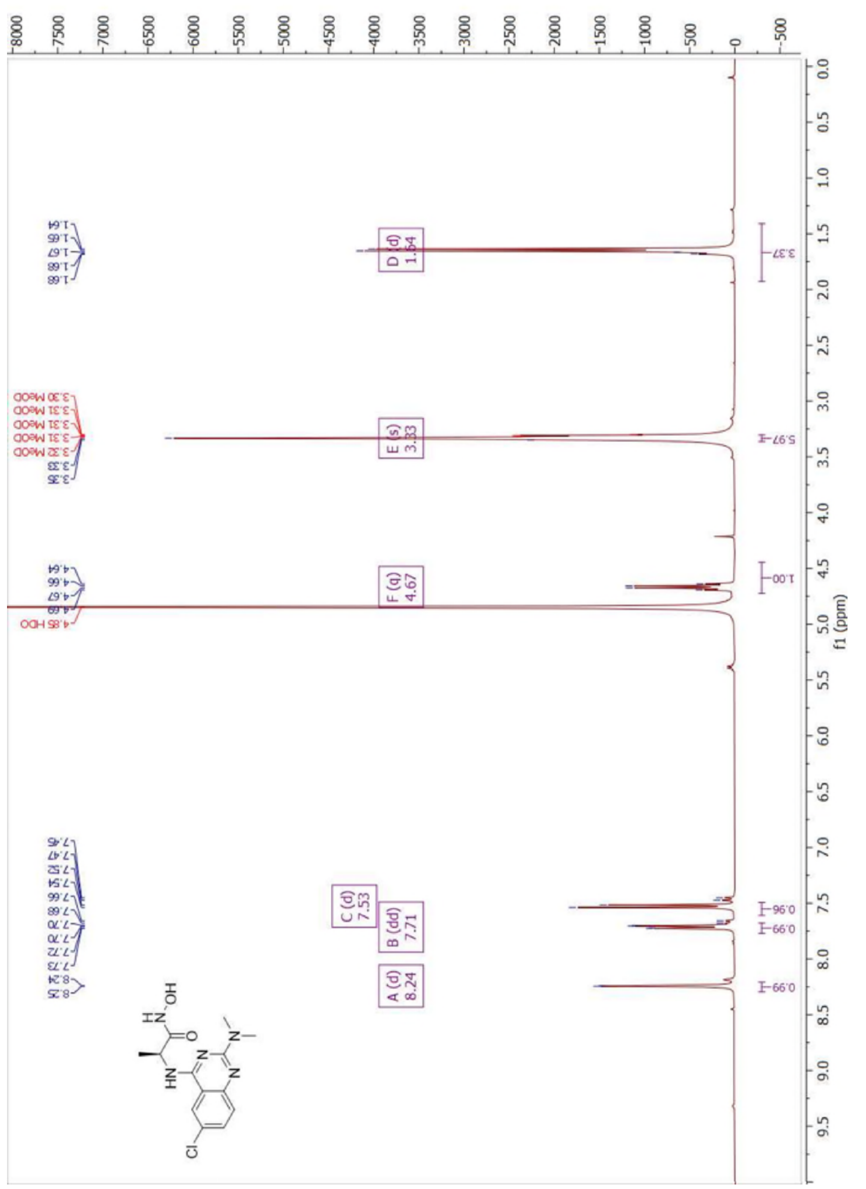

$^{13}C$ NMR

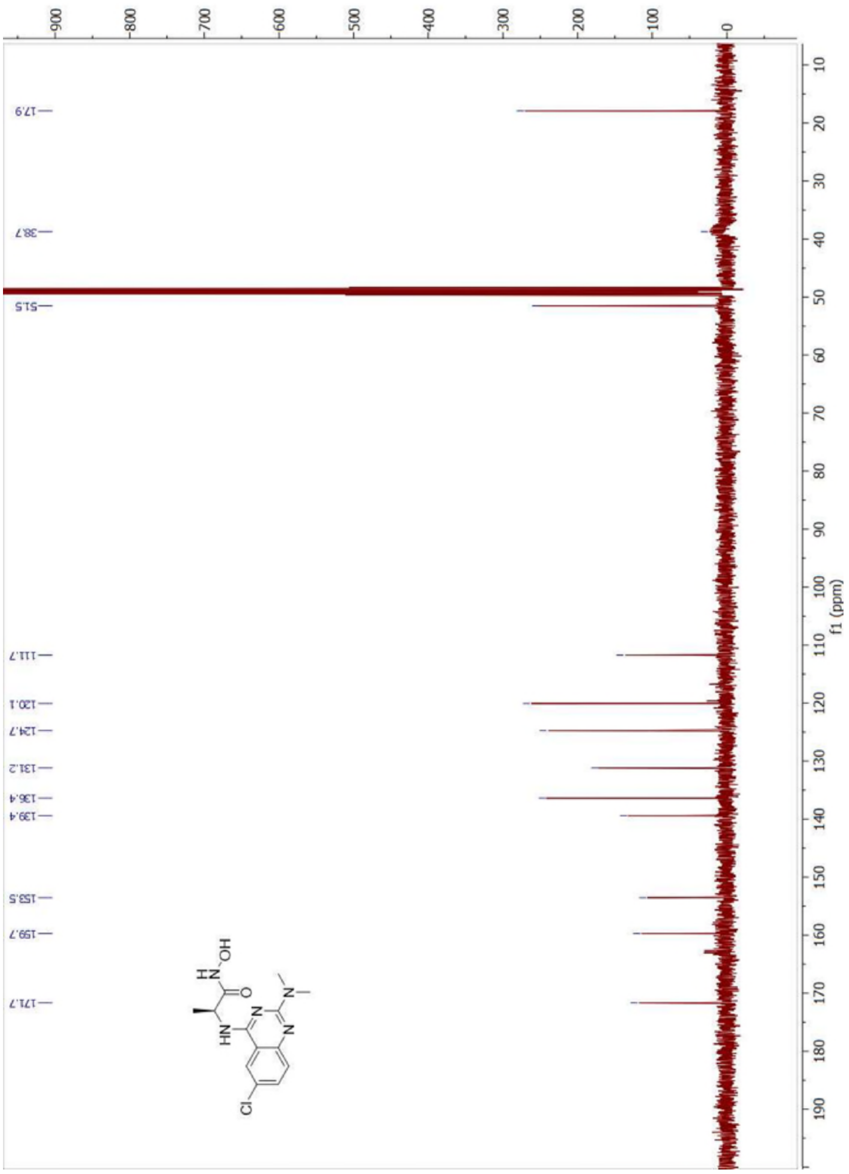
17.9
38.7
51.5
111.7
120.1
124.7
131.2
136.4
139.4
153.5
159.7
171.7
f1 (ppm)

**Methyl (2-chloro-6-fluoroquinazolin-4-yl)-*L*-alaninate (55)**

$^1H$ NMR

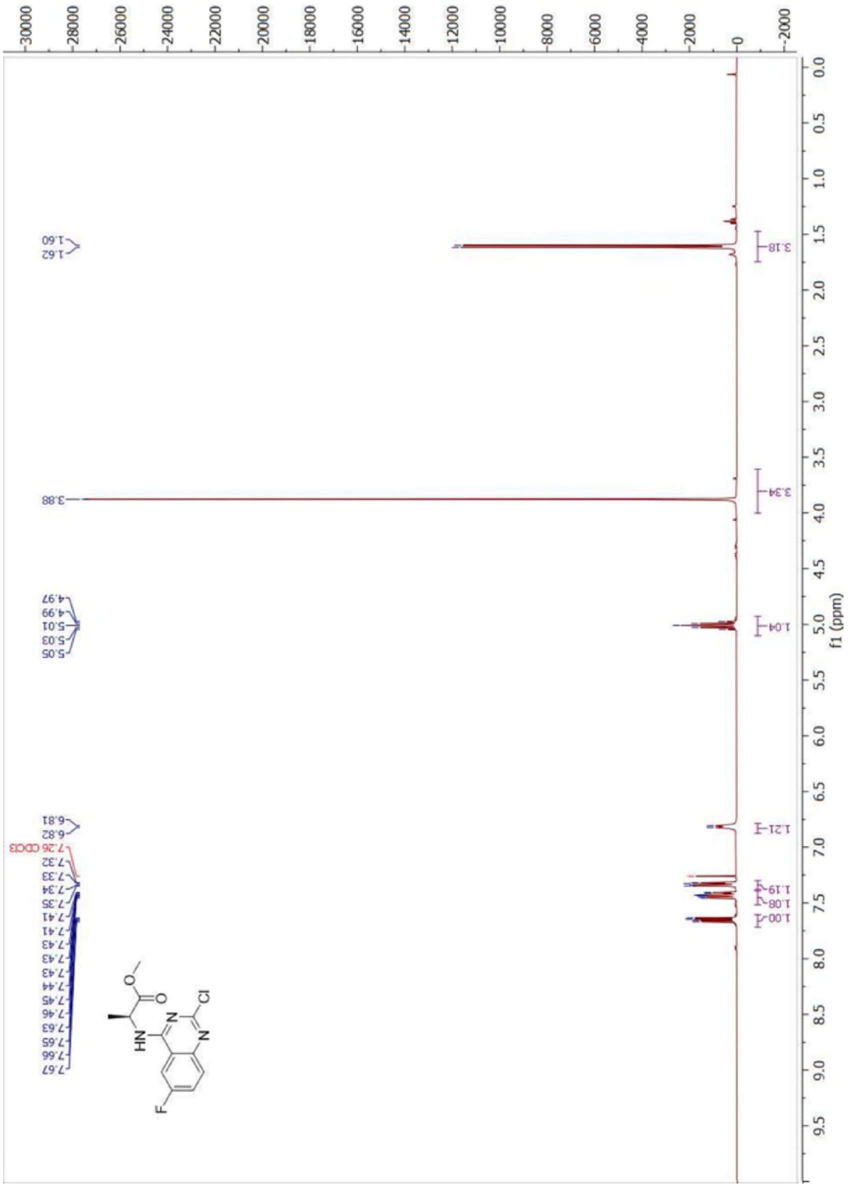

$^{13}$C NMR

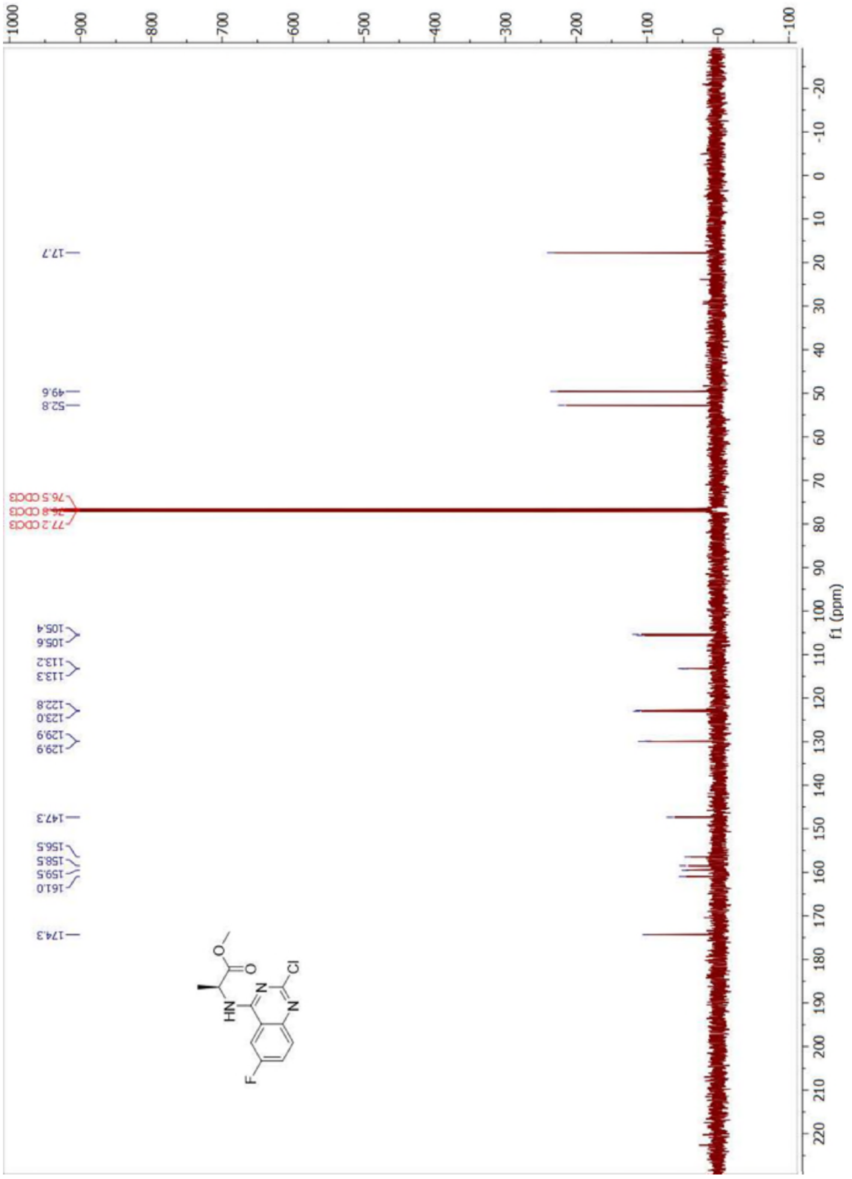

17.7
49.6
52.8
76.5 CDCl3
76.8 CDCl3
77.2 CDCl3
105.4
105.6
113.2
113.3
122.8
123.0
129.9
129.9
147.3
156.5
158.5
159.5
161.0
174.3
f1 (ppm)

$^{19}F$ NMR

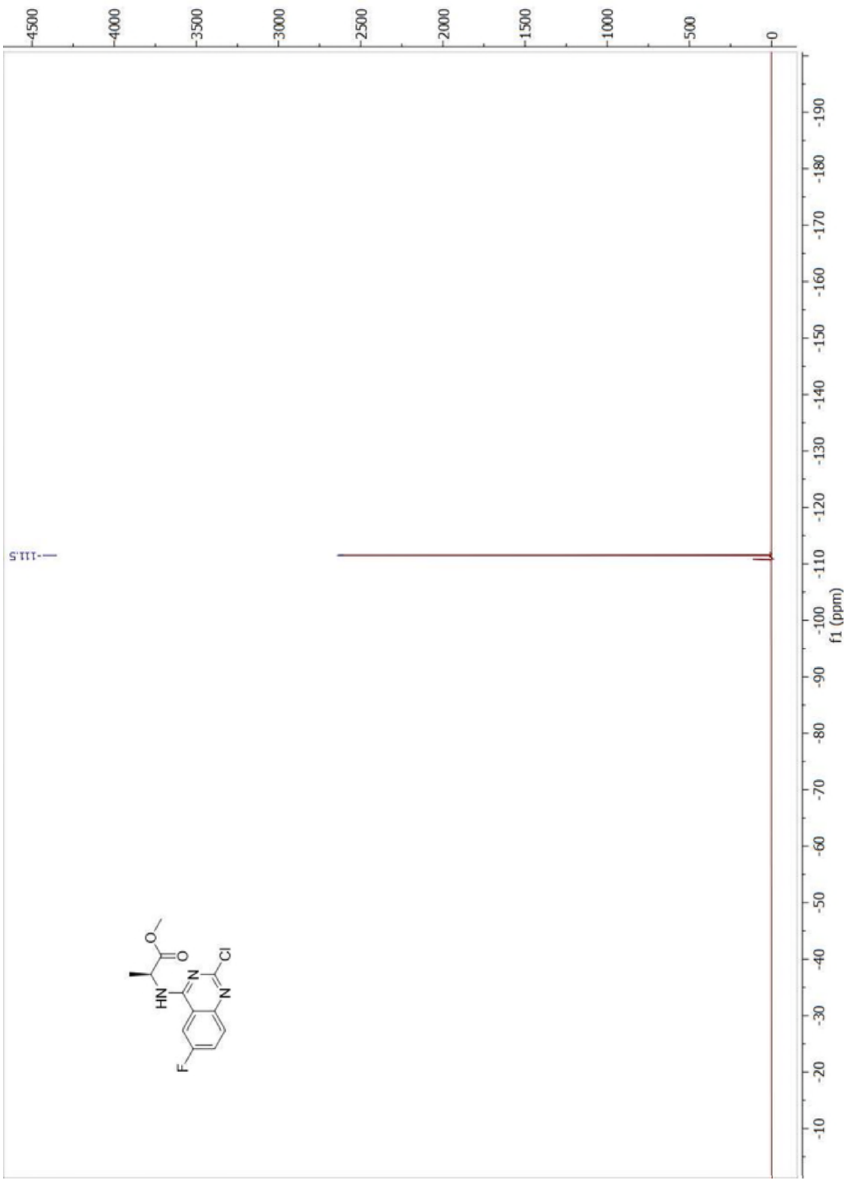

**Methyl (2-dimethylamino-6-fluoroquinazolin-4-yl)-*L*-alaninate (56)**

$^1$H NMR

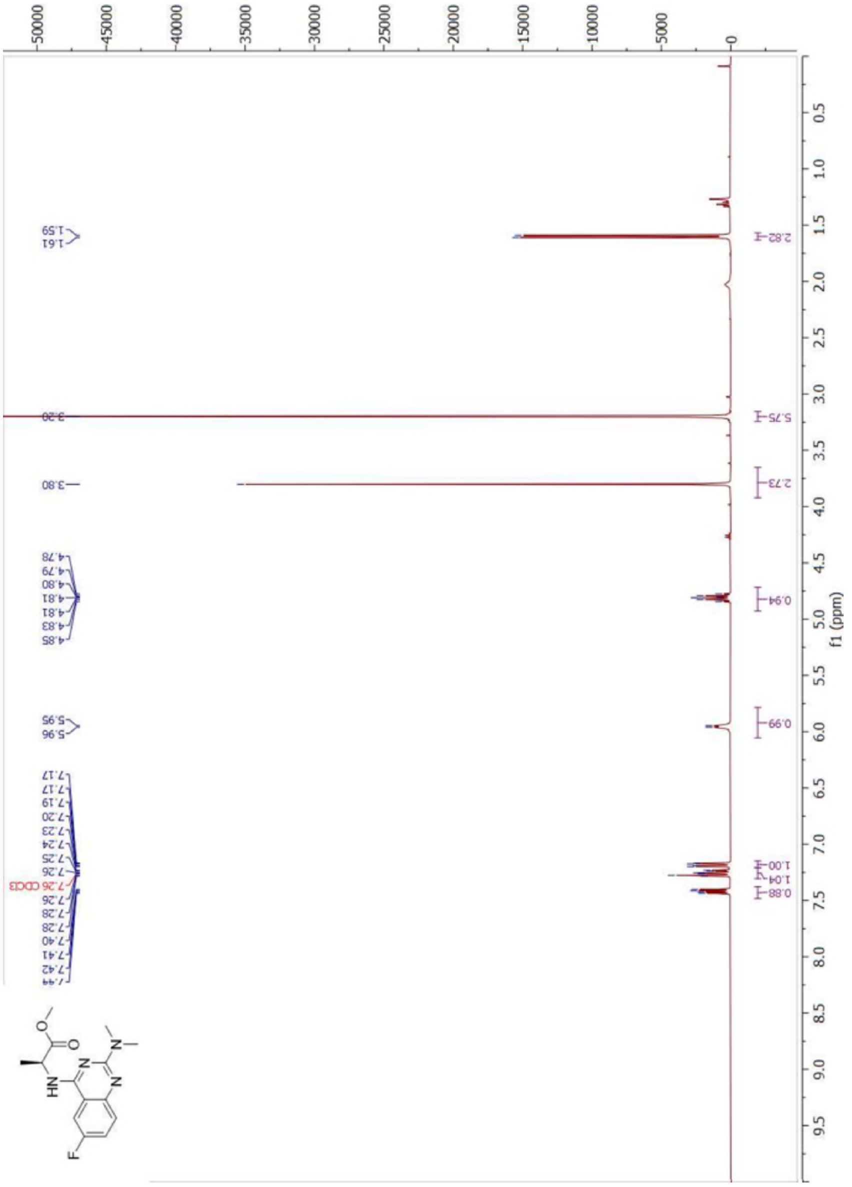

$^{13}C$ NMR

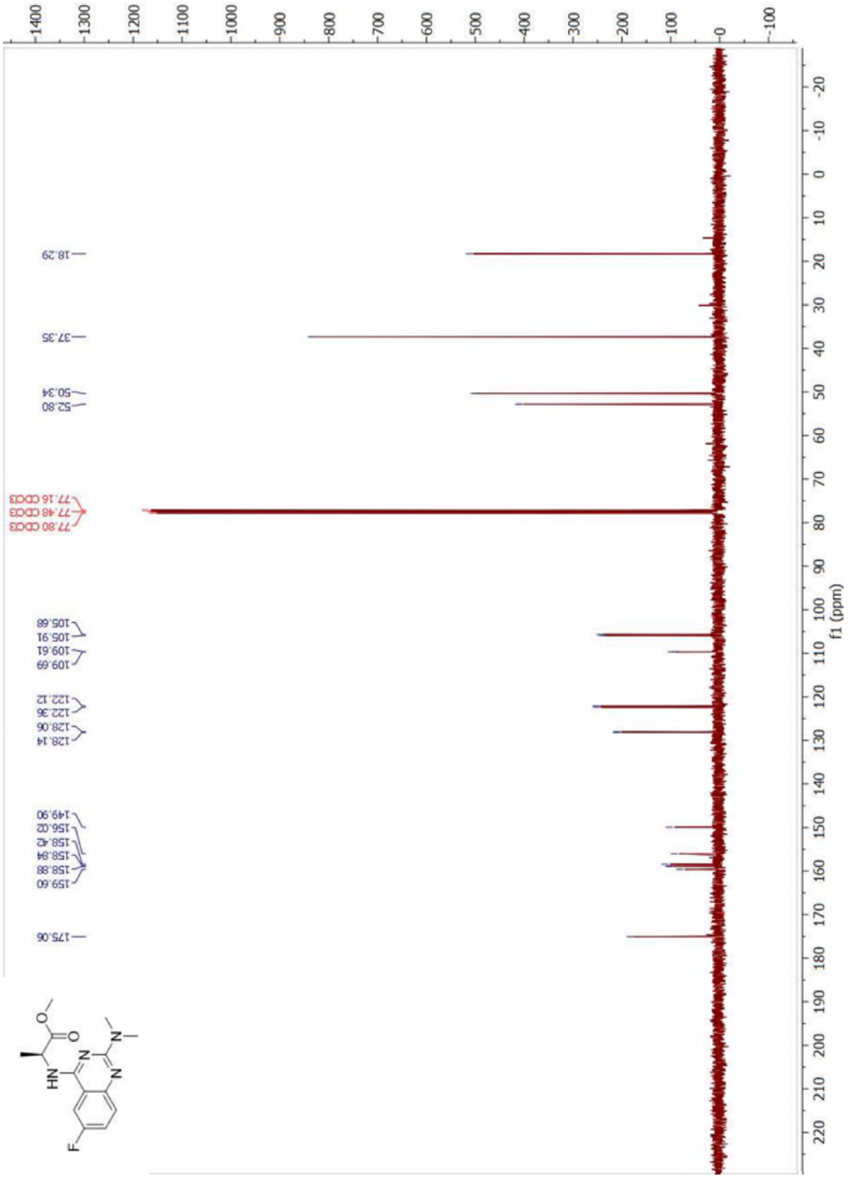
18.29
37.35
50.34
52.80
77.16 CDCl3
77.48 CDCl3
77.80 CDCl3
105.68
105.91
109.61
109.69
122.12
122.36
128.06
128.14
149.90
156.02
158.42
158.84
158.88
159.60
175.06
f1 (ppm)

$^{19}F$ NMR

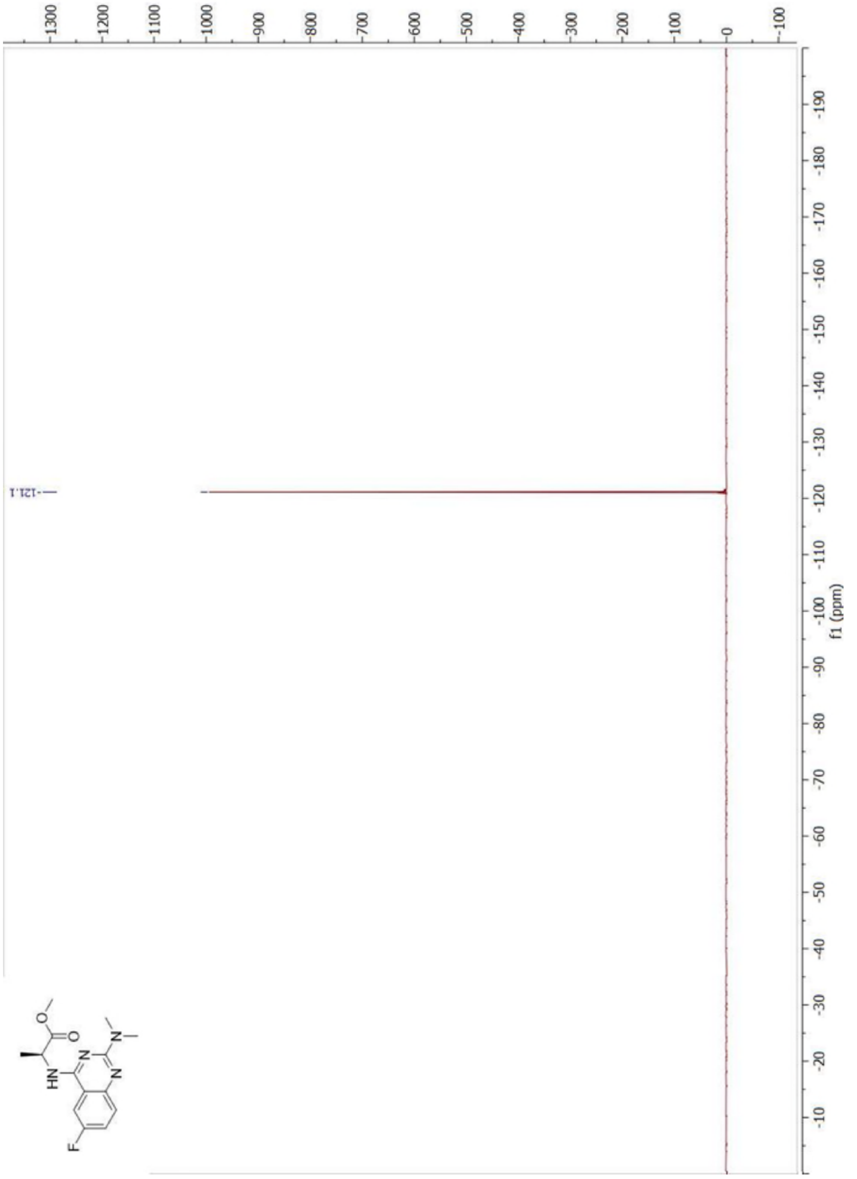

**(*S*)-2-(2-Dimethylamino-6-fluoroquinazolin-4-yl)amino-*N*-hydroxypropanamide (23)**

$^{1}H$ NMR

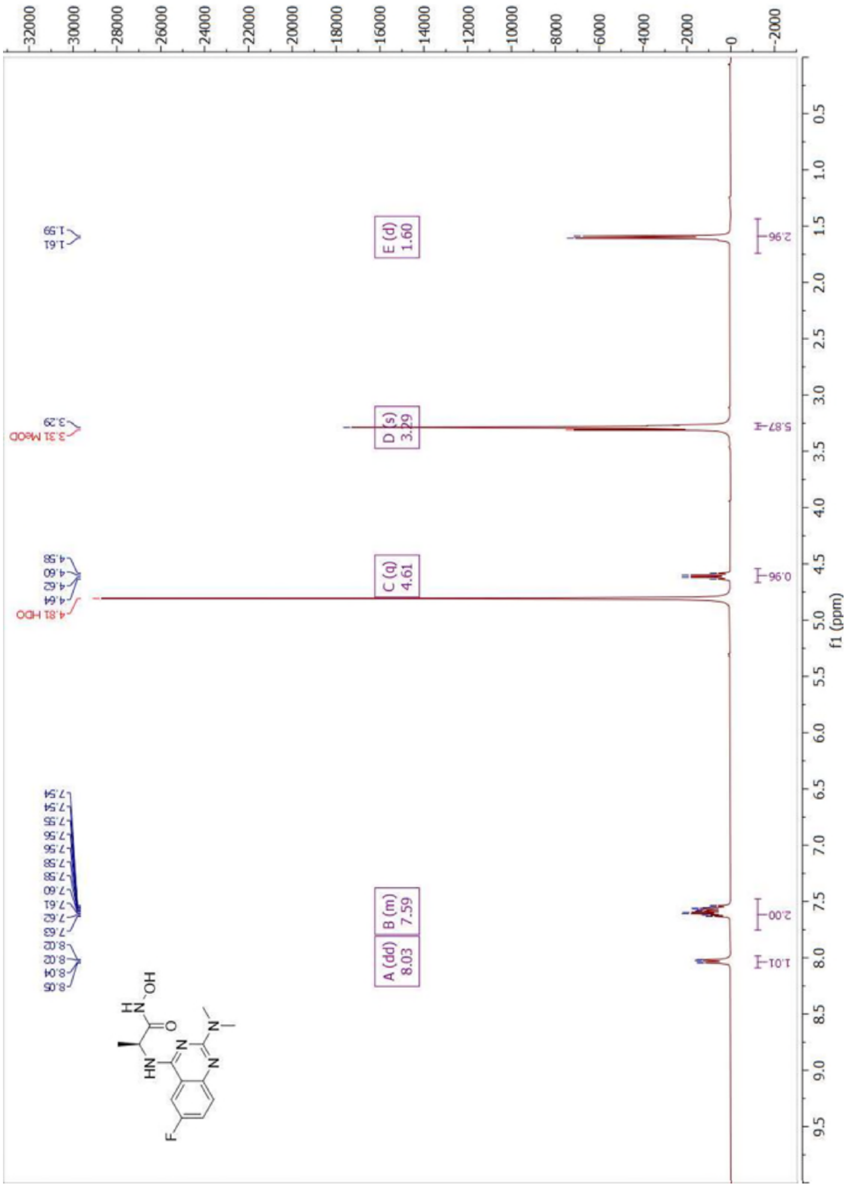

$^{13}C$ NMR

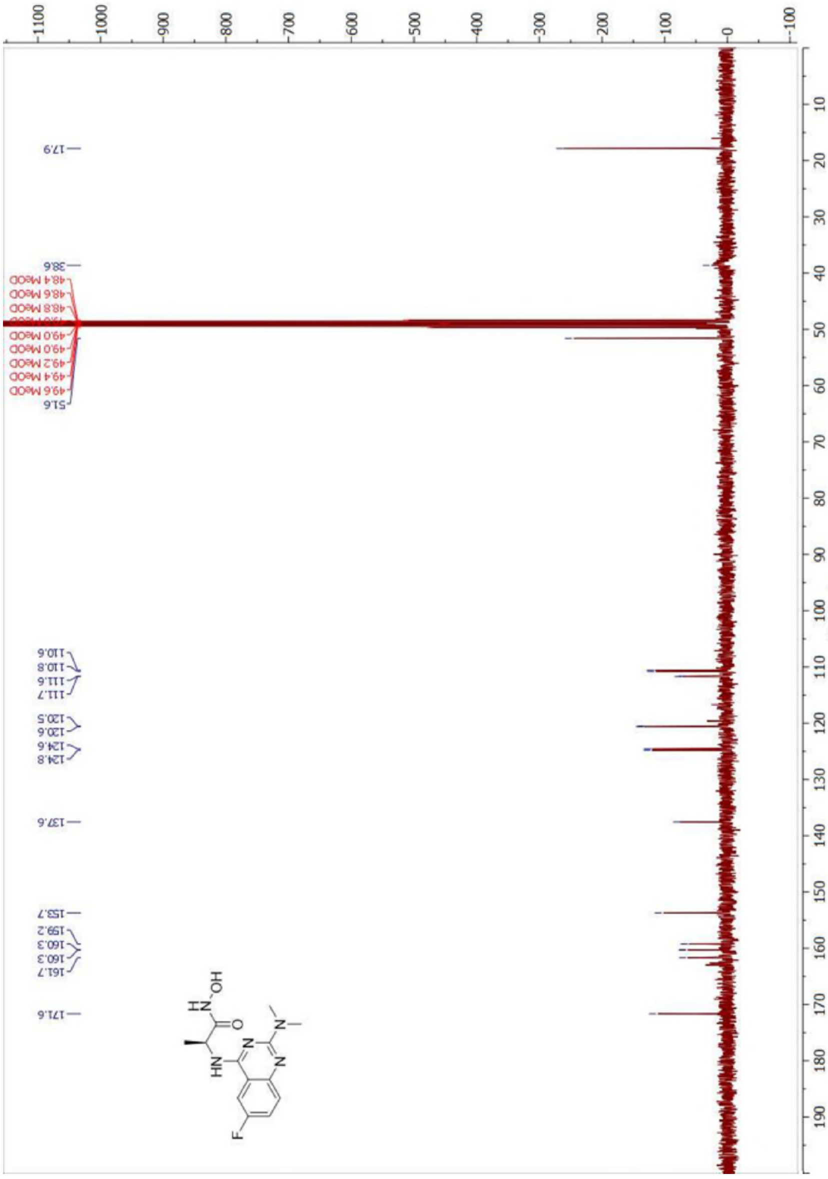
17.9
38.6
48.4 MeOD
48.6 MeOD
48.8 MeOD
49.0 MeOD
49.0 MeOD
49.2 MeOD
49.4 MeOD
49.6 MeOD
51.6
110.6
110.8
111.6
111.7
120.5
120.6
124.6
124.8
137.6
153.7
159.2
160.3
160.3
161.7
171.6
f1 (ppm)

$^{19}F$ NMR

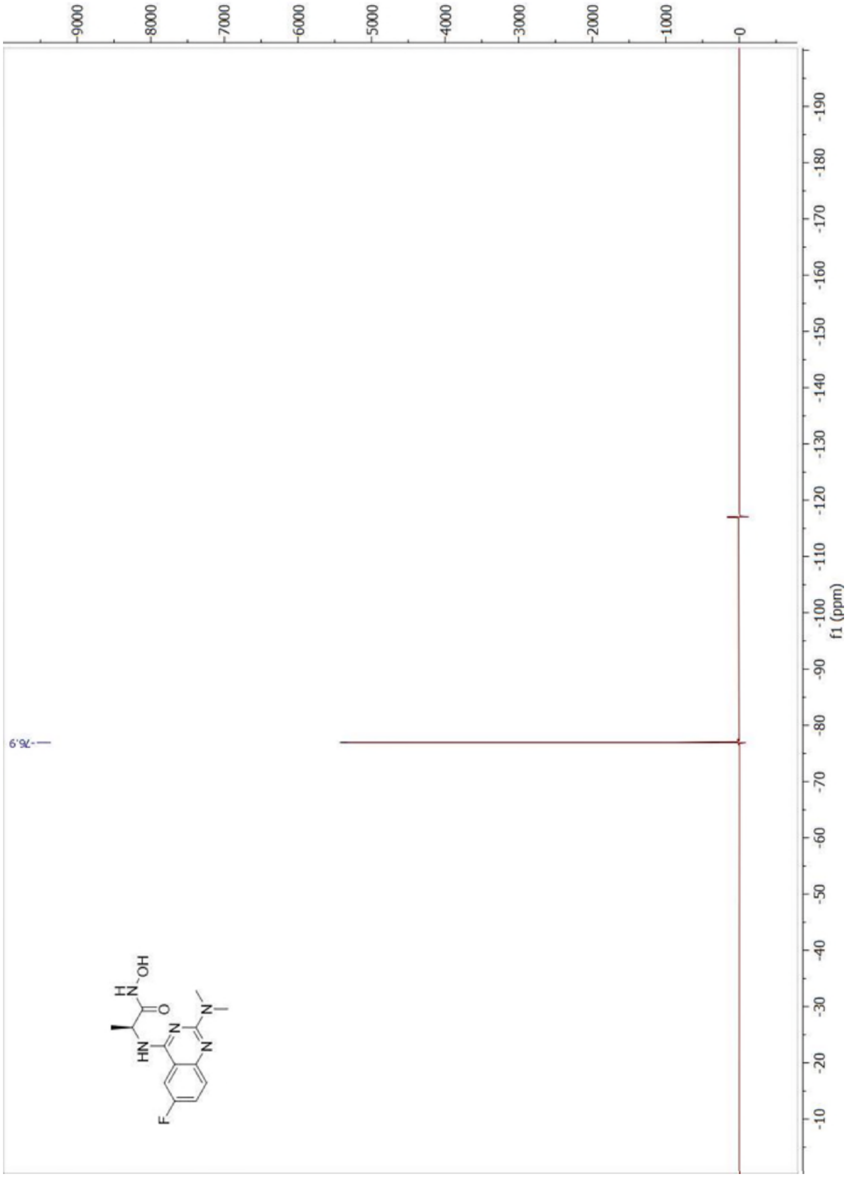

**Methyl (2-chloro-6-methoxyquinazolin-4-yl)-*L*-alaninate (57)**

$^1$H NMR

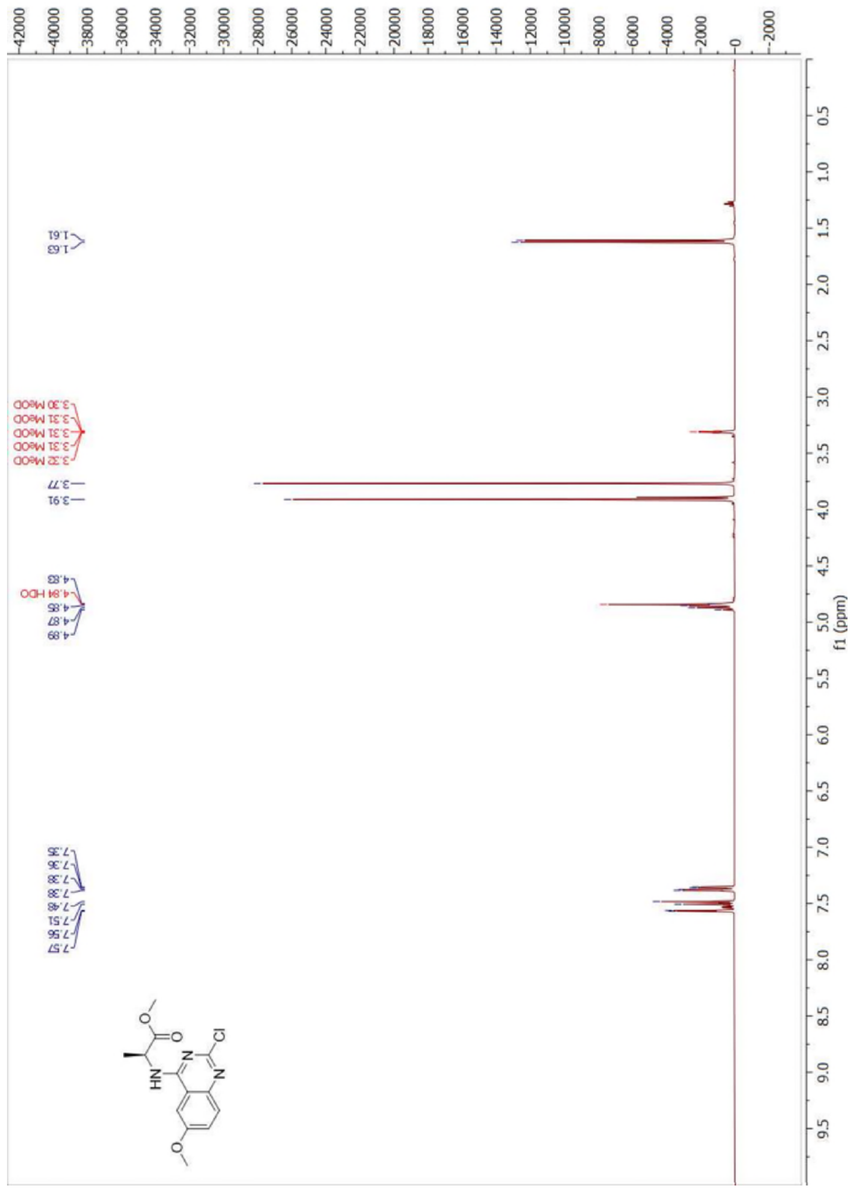

$^{13}C$ NMR

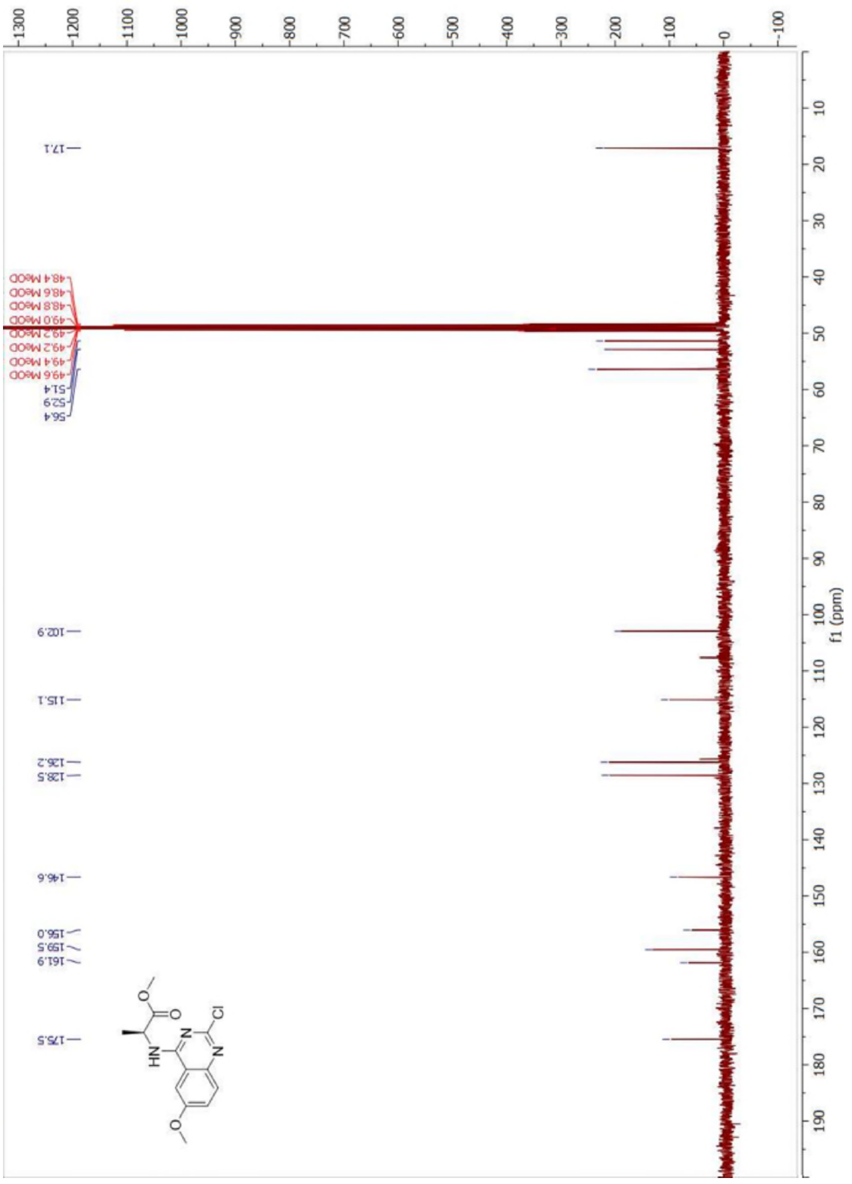

17.1
48.4 MeOD
48.6 MeOD
48.8 MeOD
49.0 MeOD
49.2 MeOD
49.2 MeOD
49.4 MeOD
49.6 MeOD
51.4
52.9
56.4
102.9
115.1
126.2
128.5
146.6
156.0
159.5
161.9
175.5
HN
N
N
Cl
O
O
f1 (ppm)

**Methyl (2-dimethylamino-6-methoxyquinazolin-4-yl)-*L*-alaninate (58)**

$^1$H NMR

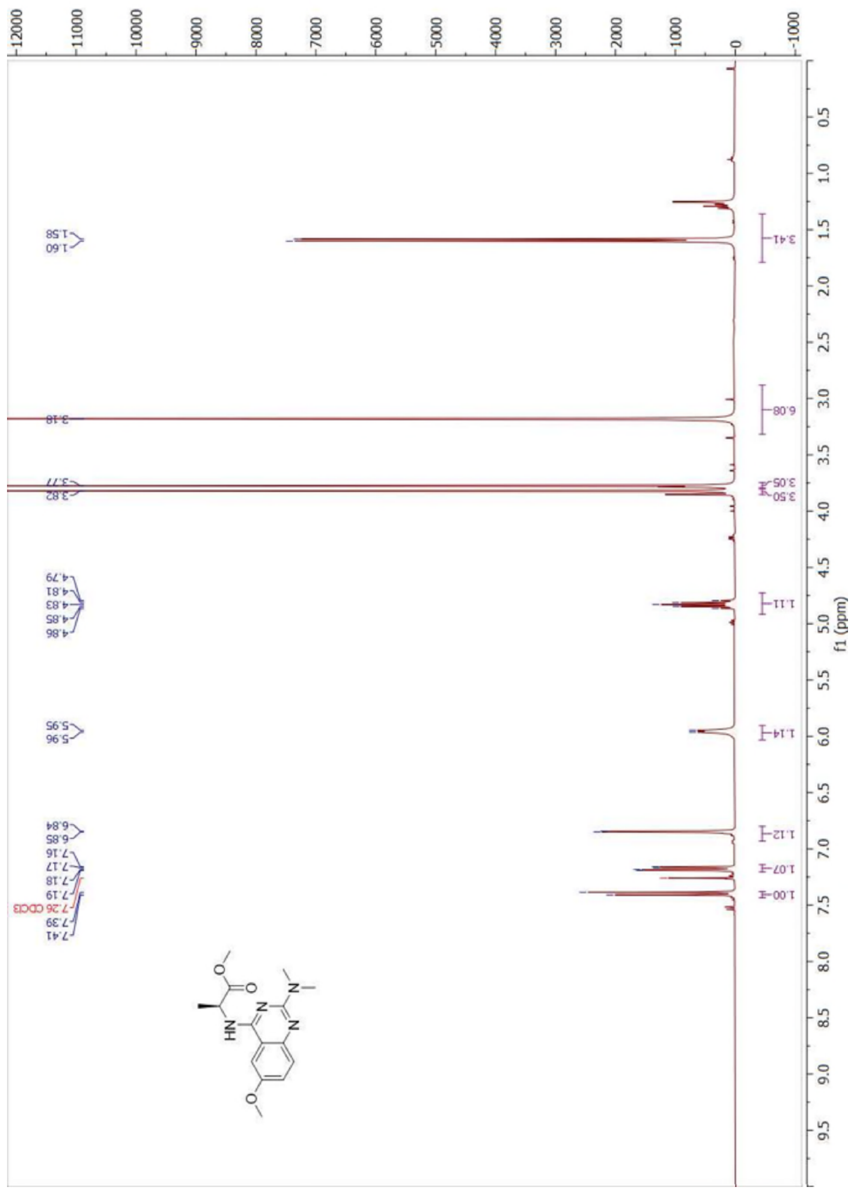

$^{13}C$ NMR

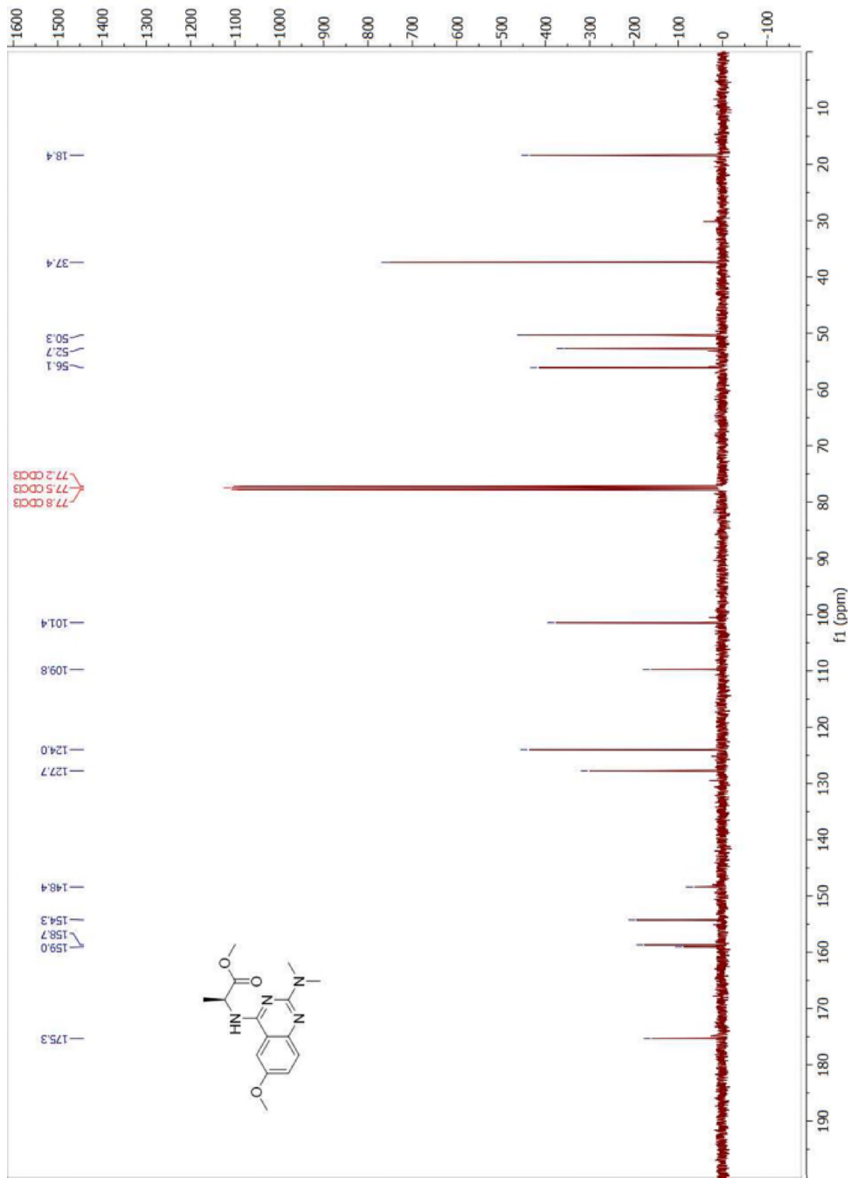

**(*S*)-2-(2-Dimethylamino-6-methoxyquinazolin-4-yl)amino-*N*-hydroxypropanamide (24)**

$^1$H NMR

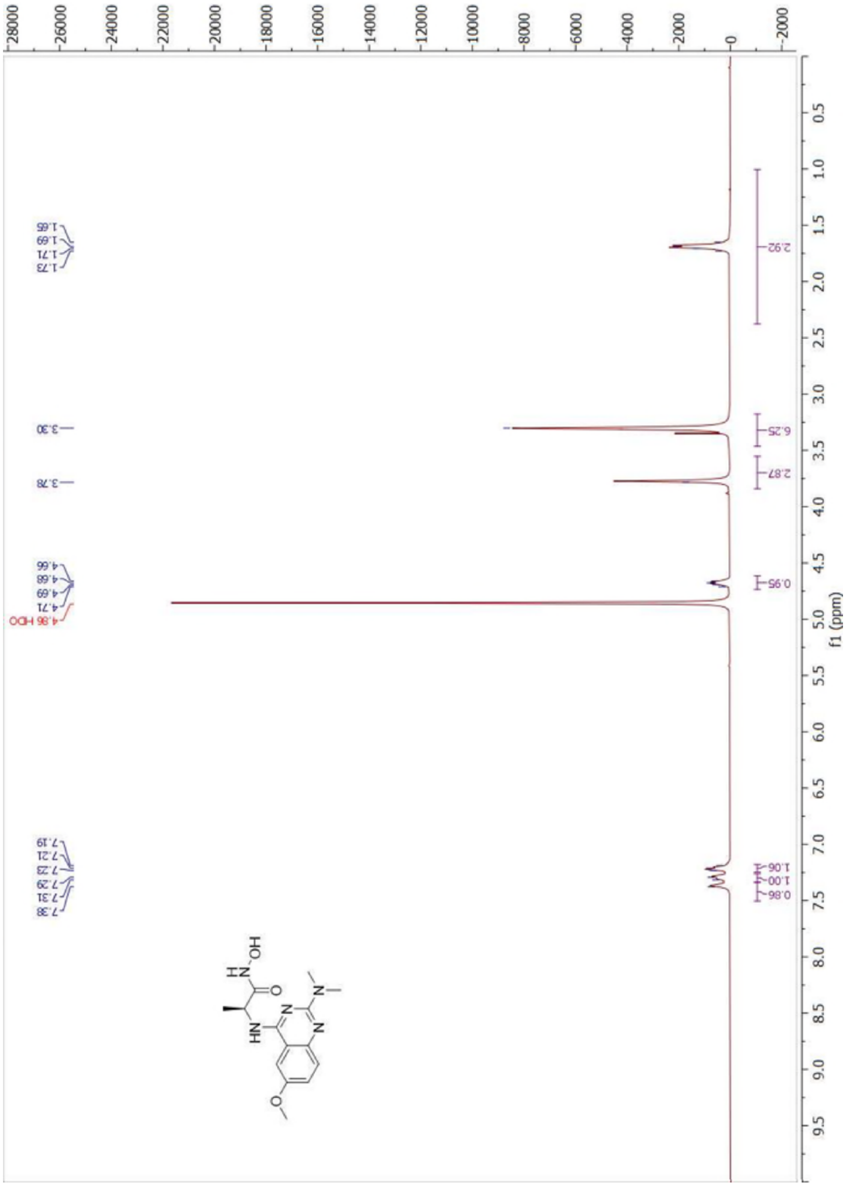

$^{13}C$ NMR

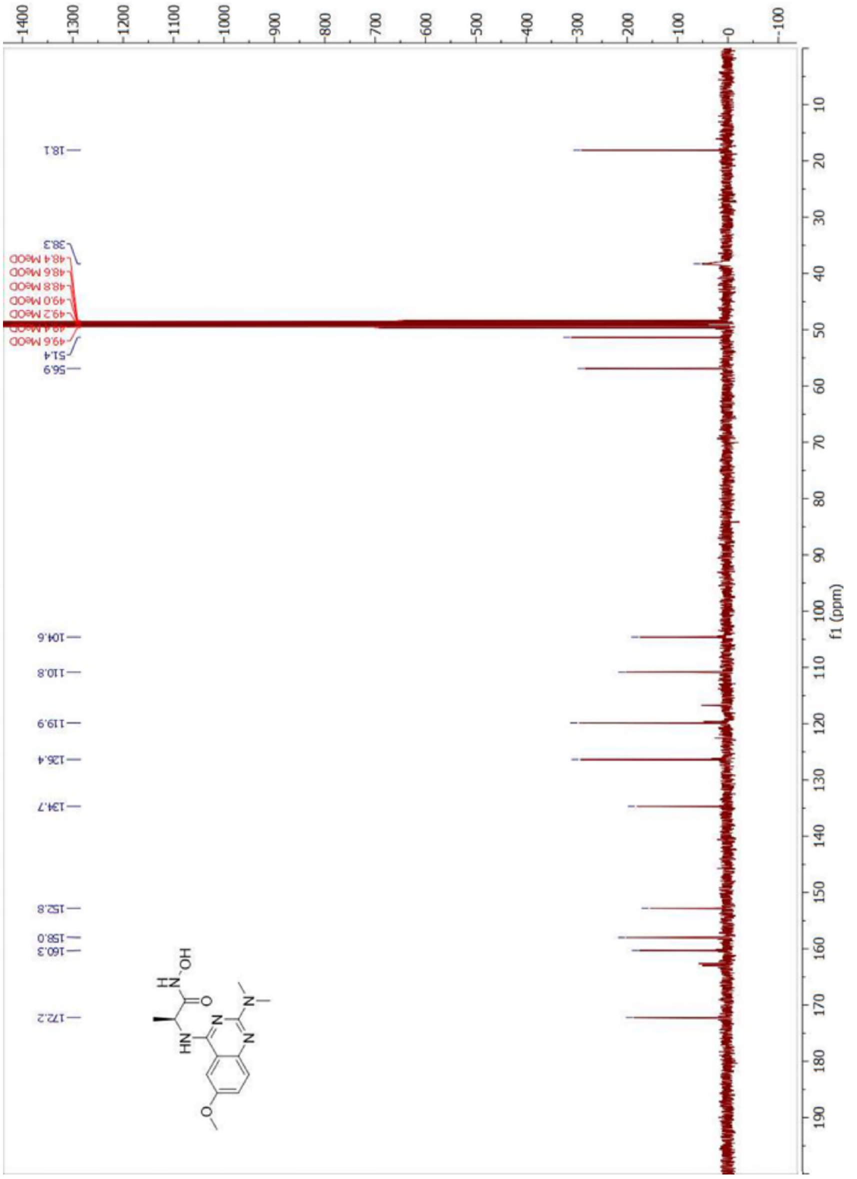

18.1
38.3
48.4 MeOD
48.6 MeOD
48.8 MeOD
49.0 MeOD
49.2 MeOD
49.4 MeOD
49.6 MeOD
51.4
56.9
104.6
110.8
119.9
126.4
134.7
152.8
158.0
160.3
172.2
f1 (ppm)
